# X‑ray Crystallography-Guided Design and Synthesis of Cyclopentyl Heteroaryl Carboxylic Acid-Based Inhibitors of the SARS-CoV‑2 Nsp3 Macrodomain (Mac1)

**DOI:** 10.1021/acs.jmedchem.6c00236

**Published:** 2026-07-13

**Authors:** Xinyu Wang, William T. W. Butler, James R. Donald, Yuran Wang, Alice L. Shaw, Marion Schuller, Daren Fearon, Jasmin C. Aschenbrenner, Peter G. Marples, Grant Watt, Yang Lu, Simon C. C. Lucas, Silvia Bonomo, Jennifer E. Nelson, Ivan Ahel, Frank von Delft, Peter O’Brien

**Affiliations:** † Department of Chemistry, 8748 University of York, York YO10 5DD, U.K.; ‡ Sir William Dunn School of Pathology, University of Oxford, South Parks Road, Oxford OX1 3RE, U.K.; § Diamond Light Source Ltd, 120796 Harwell Science and Innovation Campus, Didcot Oxfordshire, U.K.; ∥ Research Complex at Harwell, Harwell Science and Innovation Campus, Didcot Oxfordshire, U.K.; ⊥ Hit Discovery, Discovery Sciences, R&D, AstraZeneca, 1 Francis Crick Ave, Cambridge CB2 0AA, U.K.; # Centre for Medicines Discovery, University of Oxford, NDM Research Building, Oxford, Oxfordshire OX3 7FZ, U.K.; ∇ Department of Biochemistry, University of Johannesburg, Johannesburg 2092, South Africa

## Abstract

Mac1 is a conserved macrodomain enzyme in the nonstructural protein 3 (Nsp3) of SARS-CoV-2 and is part of the viral replication machinery. Mac1 is a target for small-molecule inhibitors that could ultimately enable new COVID-19 therapeutics to be developed. Here, we report the structure-guided design, synthesis, and Mac1 inhibition profiling of 25 analogues derived from a hit identified through crystallographic fragment screening. The heteroaryl group and scaffold (*cis*- and *trans*-cyclopentane and cyclopentene) were varied. Two new approaches to *trans-*cyclopentanes were developed: MacMillan’s Ir/Ni-mediated photoredox cross-coupling of alcohols and Barluenga–Valdés’ metal-free cross-coupling of sulfonyl hydrazones and boronic acids. X-ray crystal structures of 19 compounds bound to Mac1 were determined to guide the design and to rationalize the observed SAR. A new family of Mac1 inhibitors with benzothiazole or amino benzothiazoles was discovered and characterized, with IC_50_ values of 6–8 μM and ligand efficiency values of up to 0.40.

## Graphical abstract

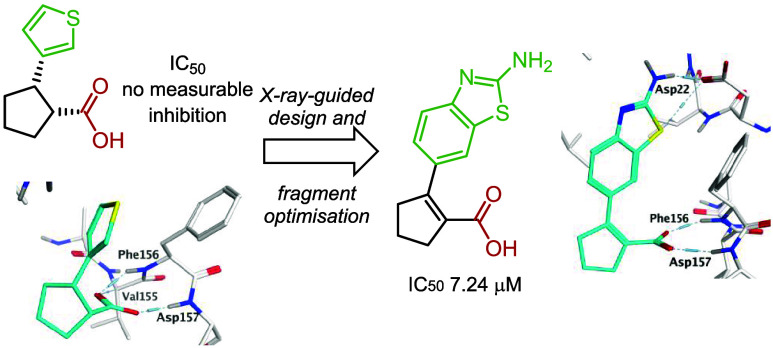

## Introduction

On 11th March 2020, the World Health Organization (WHO) declared coronavirus disease 2019 (COVID-19) a global pandemic.^1^ Since that time, the ensuing COVID-19 pandemic has had an unprecedented impact on almost everyone around the world and, with just over seven million deaths,^2^ highlights the scale and potentially devastating impact of viral diseases. The causative agent of COVID-19 is severe acute respiratory syndrome coronavirus 2 (SARS-CoV-2), which was discovered in December 2019.^3^ To combat the SARS-CoV-2 virus, from early 2020 onward, industrial and academic laboratories from around the world initiated a significant effort to explore strategies for the prevention and treatment of COVID-19. This included efforts led by the Diamond XChem facility, which were coordinated by von Delft and colleagues. Thus, high-throughput crystallographic fragment screening^4,5^ was deployed in campaigns against nine SARS-CoV-2 proteins including the main protease (M^Pro^),^6^ the nonstructural protein 3 macrodomain (Nsp3, Mac1),^7^ the nonstructural protein 13 helicase (Nsp13),^8^ the nonstructural protein 14 exonuclease/methyltransferase,^9^ and the nonstructural protein 15 endoribonuclease.^10^ This paper focuses on the structure-based optimization of an X-ray fragment hit against Mac1 to deliver a new family of Mac1 inhibitors. Of note, the initial X-ray fragment, which originated from the York 3-D Fragment Library,^11–13^ was part of the subset of 3-D fragments which were designed to be in new chemical space and synthetically enabled for rapid follow-up synthetic work.^12^ As a result, the initial stages of the fragment optimization campaign were significantly expedited.

Mac1 is a conserved macrodomain enzyme in the Nsp3 of SARS-CoV-2 and is part of the viral replication machinery. Macrodomains are a diverse protein family that is involved in both the recognition and turnover of ADP-ribose (ADPr).^14^ ADP-ribosylation is a post-translational modification^15,16^ used by the immune system to trigger the release of proinflammatory cytokines from macrophages, which suppresses viral replication.^17^ Viral macrodomains interfere with this pathway by cleaving ADPr and halting the immune signaling and inflammatory response.^18–20^ It has been shown that mutations of residues that make up the ADPr binding site led to virus attenuation, lowering of viral load, and a stronger immune response following infection compared to the wild-type virus, thereby rendering the virus nonlethal.^21^ Hence, Mac1 is important for replication and pathogenicity in hosts for coronaviruses. As a result, Mac1 can be considered a target for small-molecule inhibitors that could enable new therapeutics to be developed to treat COVID-19.^22–24^

The overall structure of ADPr bound to Mac1, together with a view of the active site residues and interactions, is shown in Figure 1A (PDB: 6W02).^25^ The active site comprises the catalytic site and the adenosine site (made up of the adenine and oxyanion subsites). The distal ribose and diphosphate groups of ADPr are bound into the catalytic site. ADPr is conjugated to the host protein via the anomeric carbon of the distal ribose (marked with an asterisk), and this is where macrodomain-mediated cleavage occurs. Most of the small-molecule inhibitors of Mac1 bind within the adenine and oxyanion subsites. The key interactions of ADPr with Mac1 in the adenine subsite are hydrogen bonds of the adenine to the carboxylic acid of Asp22 and to the backbone NH of Ile23. In a different structure of an ADPr-Mac1 complex, there was evidence of a weak π–π interaction between the adenine and Phe156 (PDB: 6WOJ).^19^

**Figure 1:**
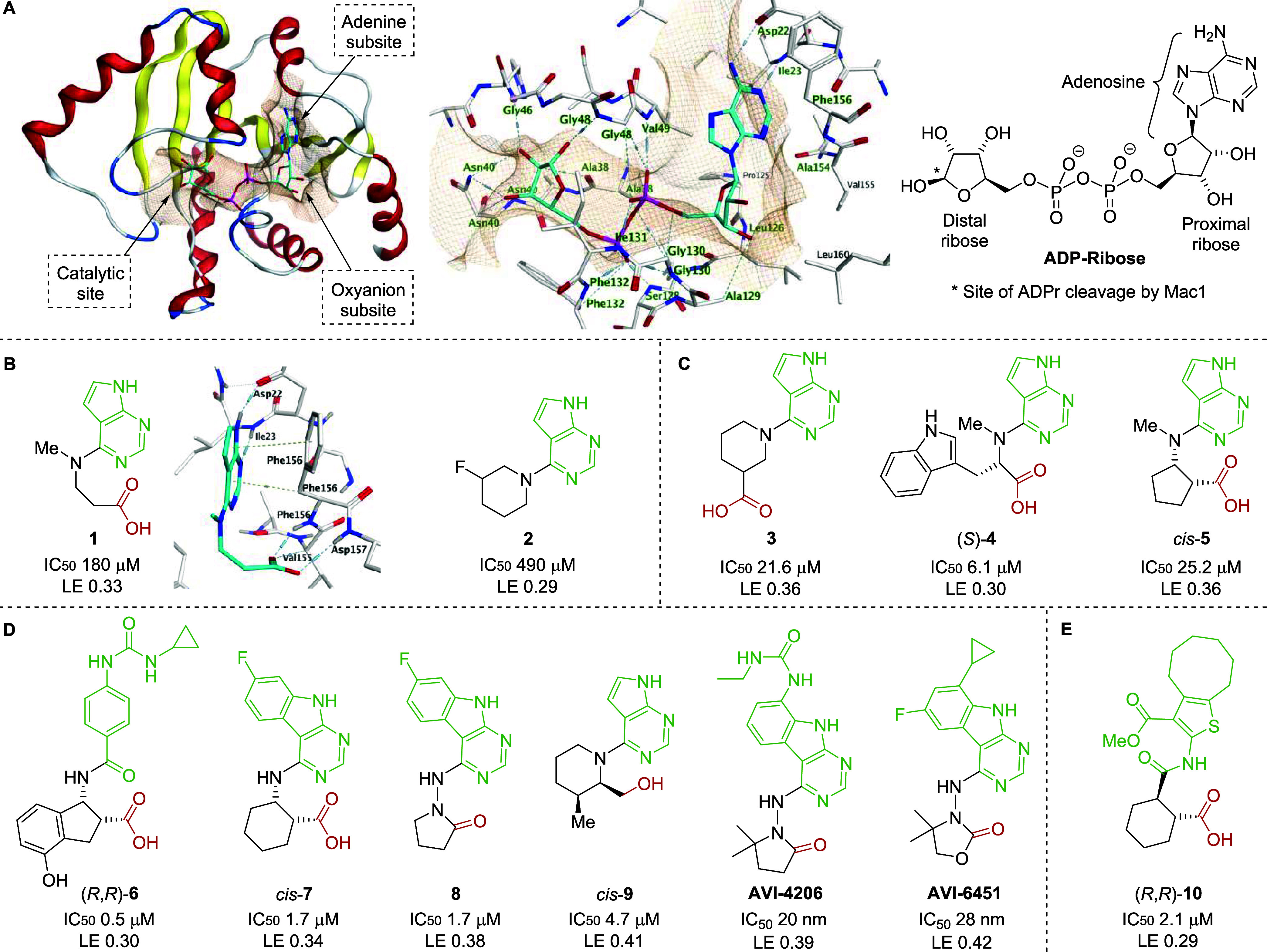
**A.** X-ray structure of the ADP-ribose-Mac1 complex (PDB: 6W02) (left panel) and the molecular interactions within the active site (middle panel); structure of ADP-ribose (right panel). **B.** von Delft, Shoichet, Fraser, Ahel et al.’s two most active fragments with X-ray structure of **1** and Mac1. **C.** Selection of Fehr, Ferraris et al.’s most active compounds. **D.** Selection of Fraser, Shochiet, Renslo, Walters, Ott et al.’s most active compounds; **E.** Fehr, Heiskanen, Lehtiö et al.’s most active compound from high-throughput screening. LE = ligand efficiency.

In 2021, the results of a comprehensive crystallographic fragment screen and computational docking campaign, carried out by the von Delft, Shoichet, Fraser and Ahel groups, were reported. This study included 234 fragment-bound X-ray crystal structures.^7^ Of these, 99 fragments bound in the adenine subsite of Mac1 and showed similar hydrogen-bonding networks to Asp22, Ile23, and Phe156 as found in the ADPr-Mac1 structure. In addition, 54 fragments bound in a newly discovered site, labeled the oxyanion subsite; ADPr does not have any direct interactions with residues in this subsite. Almost all of these 54 fragments contained a carboxylic acid substituent. One example illustrating binding to both the adenine and oxyanion subsites is pyrrolopyrimidine acid **1** (PDB: 5RSG) (Figure 1B). Hydrogen bonds of the pyrrolopyrimidine to the carboxylate of Asp22 and the backbone NH of Ile23, as well as π–π and π-CH interactions with Phe156, were observed in the adenine subsite. For the oxyanion subsite, there were hydrogen bonds from the carboxylic acid to backbone NH groups of both Phe156 and Asp157. Similar adenine subsite interactions were observed with piperidine pyrrolopyrimidine **2**. An HTRF-based peptide displacement assay was used to determine IC_50_ values. Here, an ADP-ribose-imitating part of the peptide produces a FRET-based HTRF signal upon binding to Mac1, which is disrupted by inhibitors that target the Mac1 active site. Fragments **1** and **2** exhibited the lowest IC_50_ values of 180 μM and 490 μM, respectively (Figure 1B); binding in *both* subsites led to the best inhibition of Mac1. We have calculated the ligand efficiency (LE) for all of the reported inhibitors shown in Figures 1B–E (see Experimental Section). LE is a metric that describes the binding energy per atom (excluding hydrogen atoms) of a ligand to a protein and is readily calculated from the IC_50_ value and the number of heavy (nonhydrogen) atoms in a ligand.^26^ In terms of fragments, LE values of ≥ 0.35 are useful. In these cases, there was a higher LE for **1** (LE 0.33) compared to **2** (LE 0.29).

This initial report provided the basis for follow-on inhibitor designs from Fehr, Ferraris et al. (Figure 1C).^27,28^ By combining the features of fragments **1** and **2**, piperidine **3**, which exhibited IC_50_ 21.6 μM and LE 0.36, was identified. Alternatively, α- and β-amino acid analogues of fragment **1** were explored: indole α-amino acid (*S*)-**4** (IC_50_ 6.1 μM, LE 0.30) and cyclopentane β-amino acid *cis*-**5** (IC_50_ 25.2 μM, LE 0.36) were the most effective inhibitors. Initially, no X-ray structures with fragments **3**, (*S*)-**4** and *cis-* **5** were reported, but docking studies indicated the expected binding poses in the adenine and oxyanion subsites. Subsequently, an X-ray structure (*S*)-**4** and Mac1 was disclosed.^28^

The most wide-ranging fragment follow-on work has been carried out by Fraser, Shochiet, Renslo, Walters, Ott and co-workers, and selected compounds are shown in Figure 1D.^29–32^ A fragment merging strategy identified (*R*,*R*)-**6** as a potent inhibitor (IC_50_ 0.5 μM).^29^ Further structure–activity studies led to the discovery of *cis*-**7** (IC_50_ 1.7 μM, LE 0.34),^29^ which contained a pyrrolopyrimidine derivative as found in compounds **1–3**, (*S*)-**4** and *cis-* **5**, in which the (*R*,*R*)-configured enantiomer had a similar binding pose in the adenine and oxyanion subsites. An alternative strategy that used screening by computational docking identified numerous compounds with low IC_50_ values. Efforts were also focused on addressing potential cell permeability issues afforded by the carboxylic acid in the previously developed compounds. As an example, hydrazide **8** exhibited IC_50_ 1.7 μM and LE 0.38.^29^ The X-ray structure indicated, in addition to the expected binding in the adenine subsite, two hydrogen bonds from the hydrazide carbonyl group to the backbone NH groups of Phe156 and Asp157 in the oxyanion subsite. More recent work from Fraser, Renslo, Walters et al.^30^ described a wide-ranging structure–activity study of the Mac1 binding site in which shape-based fragment linking and active learning were utilized. Relatively simple compounds, such as alcohol *cis*-**9** (IC_50_ 4.7 μM, LE 0.41), which was also structurally characterized by X-ray, had low IC_50_ values (Figure 1D). This work laid the foundations for the Fraser group’s development of **AVI-4206** (IC_50_ 20 nM, LE 0.39)^31^ and **AVI-6451** (IC_50_ 28 nM, LE 0.42),^32^ two potent inhibitors of Mac1, in which the binding modes of both have been structurally characterized. Initially, **AVI-4206** was optimized and was shown to be the first Mac1 inhibitor with in vivo efficacy in mouse models. This provided definitive proof that Mac1 is a therapeutic target.^31^ Subsequently, **AVI-6451**, which exhibited high bioavailability^32^ was developed and shows much promise for the development of future therapeutics targeting Mac1. Finally, in terms of a fragment starting point, there have been a recent report of a hit-to-lead optimization.^33^

As well as the fragment screening, merging and optimization studies summarized here (see Figure 1B–D), there has been a few examples of the discovery of Mac1 inhibitors from virtual and high-throughput screening (HTS) of larger molecular weight compounds.^34–38^ Of these, Fehr, Heiskanen, Lehtiö et al. reported the X-ray structure-characterized inhibitor (*R*,*R*)-**10** (Figure 1E), with IC_50_ 2.1 μM, which also contained a carboxylic acid binding in the oxyanion subsite.^37^ Acid (*R*,*R*)-**10** was selective for SARS-CoV-2 Mac1 and was the first Mac1-targeted inhibitor of coronavirus replication in a cell model.

The starting point for our structure-based optimization of fragment hits against Mac1 was the X-ray structures of fragments *cis*-**11** (racemic) (PDB: 5S3T) and *cis*-**12** (racemic) (PDB: 5S3X, not shown) (Figure 2).^7^ These two fragments were initially considered as they were synthetically enabled O’Brien group 3-D fragments that presented scaffolds for Mac1 inhibitor development in potential novel chemical space. Both fragments had no measurable inhibition of Mac1 when assayed up to 2 mM concentration. In the X-ray structure of *cis*-**11**, the carboxylate was hydrogen-bonded to backbone NH groups of both Phe156 and Asp157 in the oxyanion subsite. In addition, there was evidence of a weak π–π interaction between the thiophene group in *cis*-**11** and the phenyl ring of Phe156. The thiophene group projected into the adenine-binding subsite and appeared ideally suited to design variations. For initial efforts, we planned to maintain the carboxylic acid group since our work predated the recent developments reported by the Fraser group (e.g., **8**, and *cis*-**9**). Due to the simplicity of the scaffold in *cis*-**11** and commercial availability of its β-keto ester precursor, the cyclopentane scaffold was selected. Variation of the aryl group would be straightforward, as 3-D fragments such as *cis*-**11** were designed to enable follow-on elaboration.^12^

**Figure 2:**
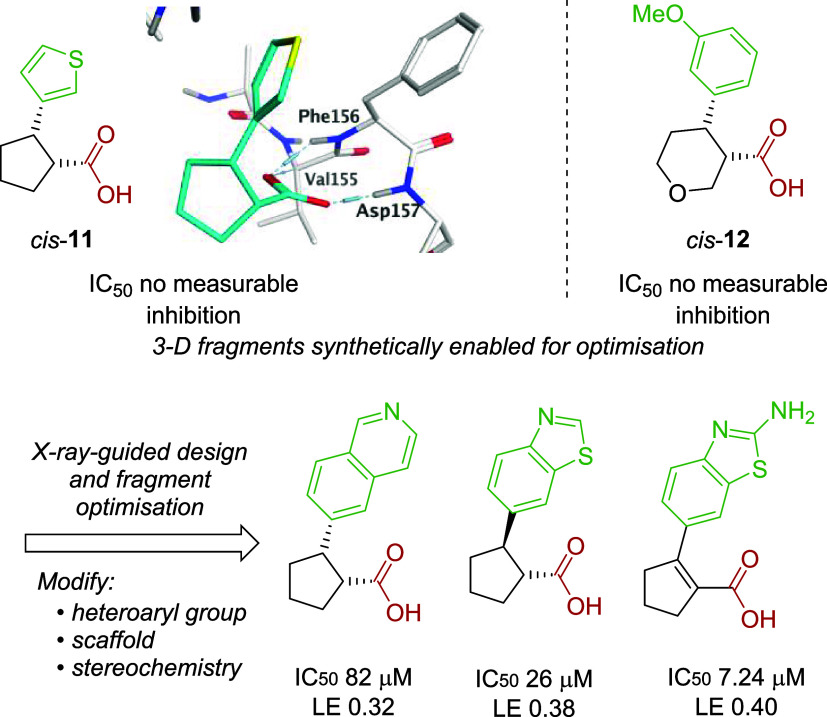
Inhibitor design and development described in this work.

In this paper, we report the design, synthesis, and Mac1 inhibition profiling of 25 analogues of crystallographic fragment hit *cis*-**11** in which the heteroaryl group and scaffold were varied (*cis*- and *trans*-cyclopentane and cyclopentene). The *cis*-cyclopentanes and cyclopentenes were readily synthesized using established routes utilizing the design features of our synthetically enabled 3-D fragments.^12^ In contrast, two new approaches for cross-coupling to give the *trans-*cyclopentanes needed to be developed and are described herein. Our results are supported by X-ray crystal structures of 19 compounds bound to Mac1. Of note, a new family of Mac1 inhibitors incorporating a benzothiazole or amino benzothiazole group was discovered and characterized. Our fragment optimization studies culminated with the development of readily synthesized cyclopentene amino benzothiazoles in novel chemical space which had IC_50_ values of 6–8 μM and LE values up to 0.40. Herein, we describe our results.

## Results and Discussion

The starting point for the design of cyclopentyl aryl carboxylic acid-based inhibitors of Mac1 was the X-ray crystal structure of fragment *cis*-**11** bound to Mac1 (PDB: 5S3T) (Figure 2).^7^ For the first round of designs, we planned to maintain the *cis*-cyclopentane carboxylic acid framework and explore variation of the thiophene to produce a series of analogues. Preliminary computational docking studies were carried out to aid identification of suitable heteroaryl groups. Since the original synthetic route to 3-D fragment *cis*-**11** involved Suzuki–Miyaura cross-coupling of an enol triflate ester with thiophene boronic acid and subsequent alkene hydrogenation,^12^ heteroaryl groups for the modeling were based on commercially available heteroaryl boronic acids at that time. From this, 62 aryl/heteroaryl groups were enumerated and computationally docked, resulting in two sets of interest. The computational methods used are summarized in the Experimental Section, and the docked poses are shown in the Supporting Information (SI). The selection of poses was made based on the lowest possible ligand strain and RMSD of the cyclopentyl core with respect to that in the X-ray structure of the *cis*-**11**-Mac1 complex (PDB: 5S3T), together with identification of hydrogen-bonding interactions with Asp22 and/or Ile23 in the adenine-binding subsite (see docked poses in SI). In the priority 1 set (18 compounds), the heteroaryl groups interacted via at least one hydrogen bond with either Asp22 or Ile23. The priority 2 set (6 compounds) contained a polarized −CH group able to interact with the side chain of Asp22.

With 24 potential Mac1 inhibitors based on a *cis*-cyclopentyl heteroaryl carboxylic acid motif identified, attention turned to their syntheses. Compared with our previous approach,^12^ a slightly modified strategy for the synthesis of heteroaryl cyclopentyl acids *cis*-**11** was developed (Scheme 1). For the conversion of heteroaryl bromides **13** into pinacol boronate (Bpin) derivatives **14** and subsequently into heteroaryl cyclopentyl esters **16**, we utilized a one-pot Miyaura borylation–Suzuki–Miyaura cross-coupling method, based on methodology reported by Hoarau and co-workers.^39^ In addition, we also used enol triflate *benzyl* ester **15** for the cross-coupling step with the intention that hydrogenation/hydrogenolysis could be achieved in one step to deliver the targeted acids *cis*-**17**.

**Scheme 1:**
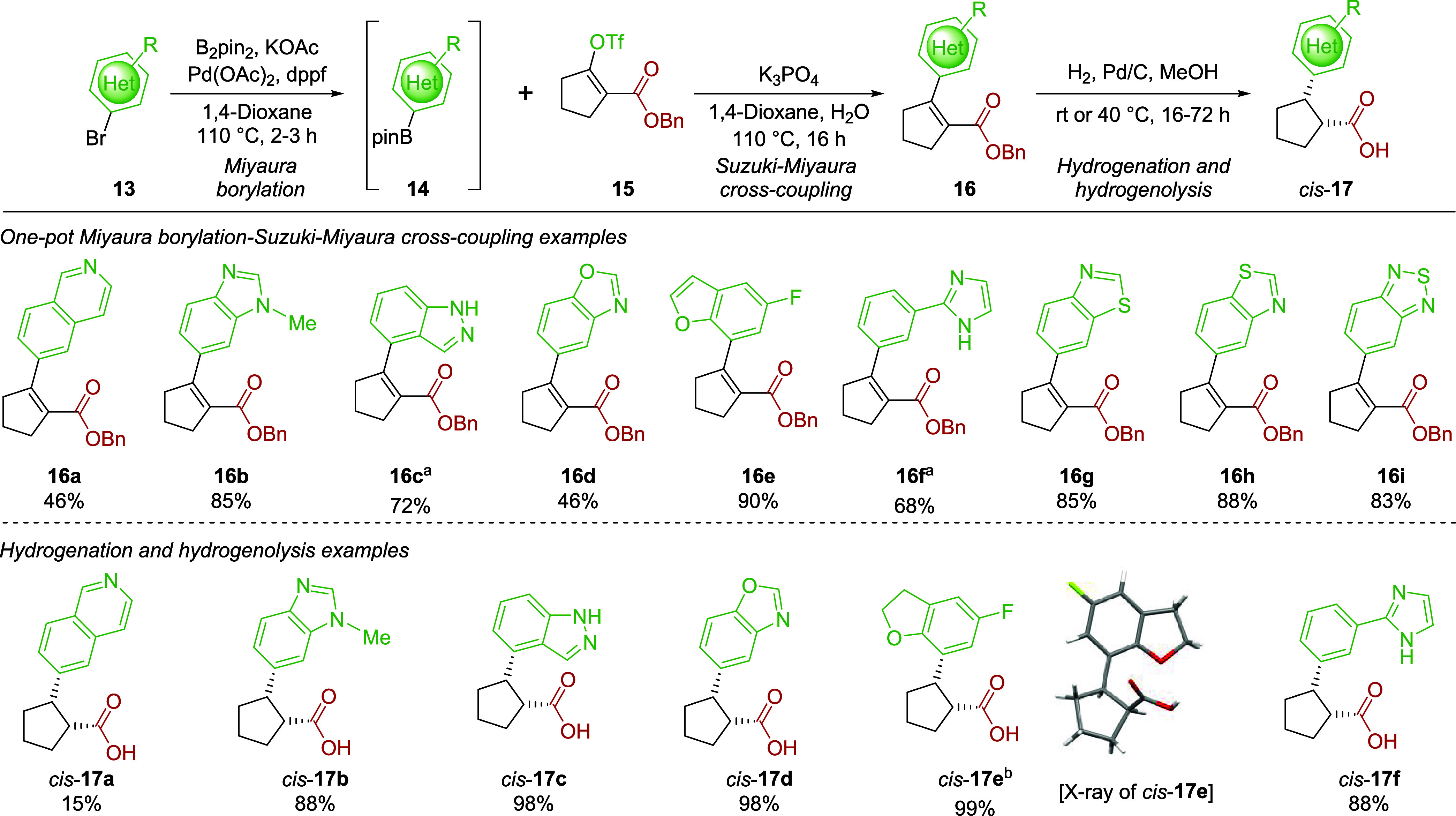
Synthesis of *cis-*Cyclopentane Acids

a
**16c** and **16f** were obtained from Cbz-protected aryl bromide.
b Cis-**17e** was obtained from benzofuran **16e**. Yields (%) are provided below each compound.

To start, Miyaura borylation of heteroaryl bromides **13** was carried out using B_2_pin_2_, KOAc, Pd­(OAc)_2_, and dppf in 1,4-dioxane at 110 °C to deliver heteroaryl Bpins **14**. Then, in the same vessel, Suzuki–Miyaura cross-coupling was accomplished with enol triflate **15** in the presence of aqueous K_3_PO_4_ using the *in situ* generated Pd(0)/dppf catalyst from the first step. In this way, cross-coupled cyclopentene benzyl esters **16a**–**i** were produced in 46–90% yields (Scheme 1). For cross-coupled products **16c** and **16f**, Cbz-protected heteroaryl bromides were used with the intention of *N*-deprotection being facilitated in the subsequent hydrogenation step. However, the Cbz groups were in fact removed in the one-pot process, almost certainly mediated by the hydroxide present in the Suzuki–Miyaura cross-coupling step (via nucleophilic attack). This delivered NH heteroaryl cyclopentyl esters **16c** (72%) and **16f** (68%).

Next, alkene hydrogenation and benzyl ester hydrogenolysis of **16a**–**f** using hydrogen and 10% Pd/C catalyst in MeOH delivered cyclopentyl heteroaryl carboxylic acids *cis*-**17a**–**f** respectively (Scheme 1). In some cases, the reactions were sluggish and were therefore carried out at 40 °C (see Experimental Section for details). Cyclopentyl acids *cis*-**17b**–**d** and *cis*-**17f** were isolated in high yields (88–98%). In contrast, sulfur-containing heteroaryl alkenes **16g**–**i** were resistant to hydrogenation/hydrogenolysis even under the more forcing 40 °C conditions. In two cases, issues with hydrogenation of the heteroaryl group were observed. Hydrogenation of isoquinoline alkene ester gave a low yield (15%) of cyclopentyl acid *cis*-**11a**, as significant over-reduction to the tetrahydroisoquinoline occurred. With benzofuran **16e**, hydrogenation of the heterocycle could not be suppressed even at room temperature, and dihydrofuran *cis*-**17e** was formed exclusively (99% yield). The expected cis configuration from the hydrogenations was confirmed by X-ray crystallography of dihydrofuran *cis*-**17e** (Scheme 1, CCDC: 2482170). The configurations of cyclopentyl heteroaryl carboxylic acids *cis*-**17a**–**d** and *cis*-**17f** were assigned by analogy and were supported by the synthesis of diastereomeric acids *trans-* **17a**–**b** *via* a *trans*-selective synthetic route (photoredox cross-coupling, *vide infra*).

Given the mixed success with the hydrogenation reactions, it was decided to synthesize a set of cyclopentenyl heteroaryl carboxylic acids **20**. This would allow us to prepare carboxylic acids with heteroaryl groups that were not accessible *via* hydrogenation and also to evaluate whether a *cis*-configured carboxylic acid was important for inhibition of Mac1. The desired cyclopentenyl aryl carboxylic acids **20** were mostly prepared using the one-pot Miyaura borylation-Suzuki–Miyaura cross-coupling reaction with enol triflate methyl ester **18** and subsequent hydrolysis. Using this method, heteroaryl methyl esters **19a**–**f** were obtained in 26–89% yields (Scheme 2). Two slightly different sets of conditions were used for ester hydrolysis starting from methyl esters **19a**–**f** and benzyl esters **16h**–**i**. These reactions proceeded uneventfully to give heteroaryl carboxylic acids **20a**–**h** in 39–95% yields (Scheme 2).

**Scheme 2:**
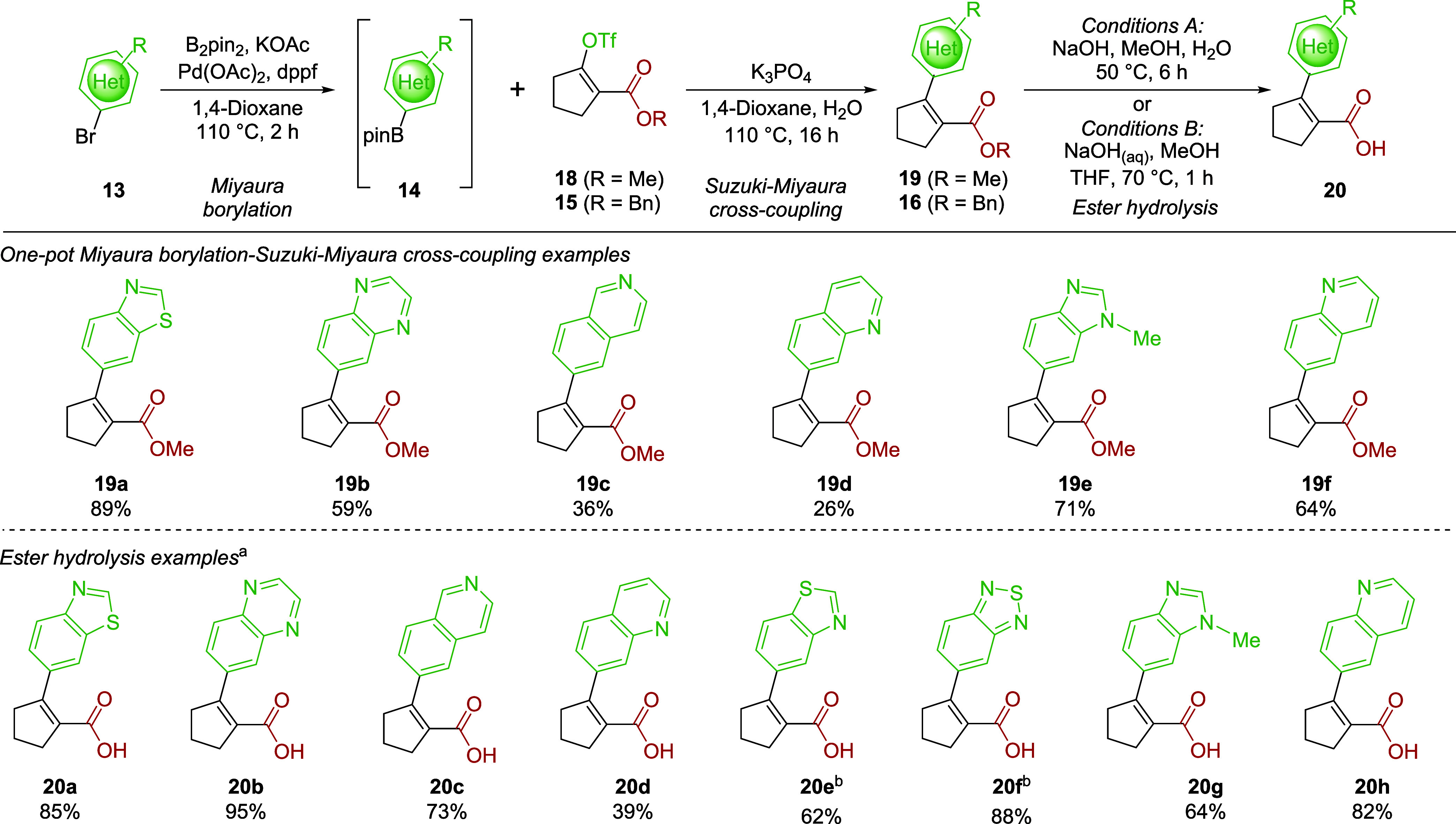
Synthesis of Cyclopentene Acids

a All examples were obtained from methyl esters using conditions A, except where stated.
b
**20e** and **20f** were obtained from benzyl esters **16h** and **16i**, respectively, using conditions B. Yields (%) are provided below each compound.

To summarize, this initial synthetic work resulted in 14 compounds (*cis*-**17a**–**f** and **20a**–**g**) being successfully prepared for Mac1 inhibition studies. Of the 24 compounds that originated from the computational designs, we prepared either a *cis*-cyclopentane (*cis*-**17a**–**d** and *cis*-**17f**) or cyclopentene (**20a**–**g**) scaffold with ten distinct heteroaryl groups. For two heteroaryl groups, compounds on both scaffolds (*cis*-**17a**/**20c** and *cis*-**17b**/**20g**) were also available for comparative purposes.

The potential of all 14 compounds (*cis*-**17a**–**f** and **20a**–**g**) as Mac1 inhibitors was assessed using an established HTRF-based peptide displacement assay.^7^ The binding of Mac1 to the ADP-ribose-imitating part of the peptide produces a FRET-based HTRF signal, which is disrupted by inhibitors targeting the active site of Mac1. The results obtained are summarized in Tables 1 and 2. Of note, three compounds, isoquinoline *cis*-**17a** (IC_50_ 82 μM), benzothiazole **20a** (IC_50_ 42 μM), and quinoxaline **20b** (IC_50_ 86 μM), showed IC_50_ values of <100 μM, with suitable LE values of 0.32–0.36. These compounds are *ca* 10-fold less active than the natural ligand ADPr which has an IC_50_ value of 1.2–2.2 μM. In both examples where the heteroaryl group was the same on two scaffolds (isoquinoline and *N*-methylbenzimidazole), the *cis*-cyclopentane scaffold resulted in more potent Mac1 inhibitors (compare *cis*-**17a** with **20c** and *cis*-**17b** with **20g**).

**Table 1:**
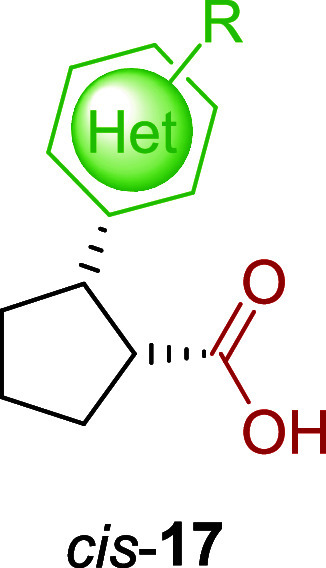

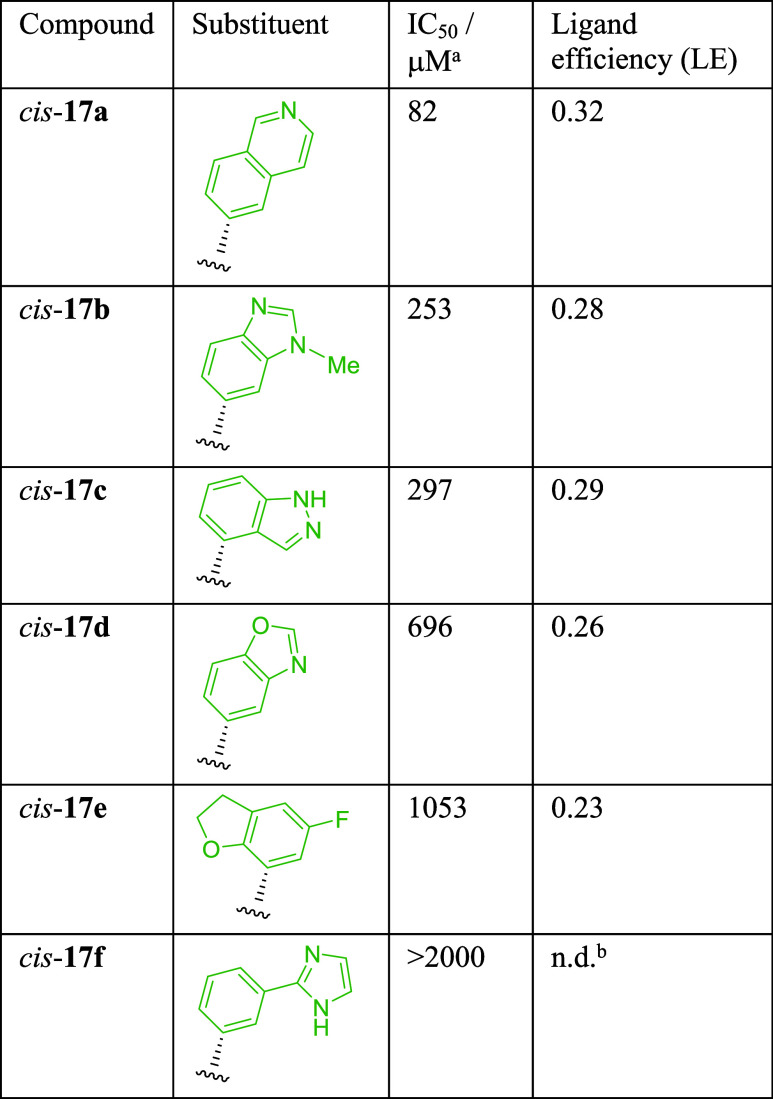
IC_50_ and LE Values for *cis*-Cyclopentane Acids *cis*-**17**

a IC_50_ values are mean values ± SD of duplicate measurements.

b Not determined.

**Table 2:**
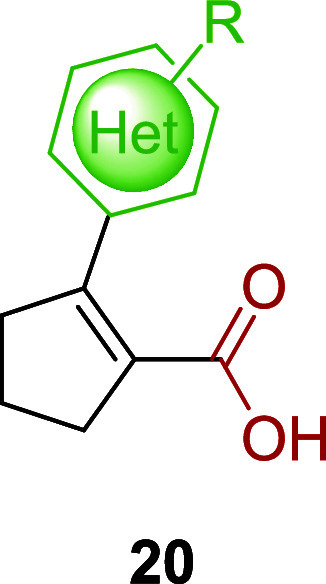

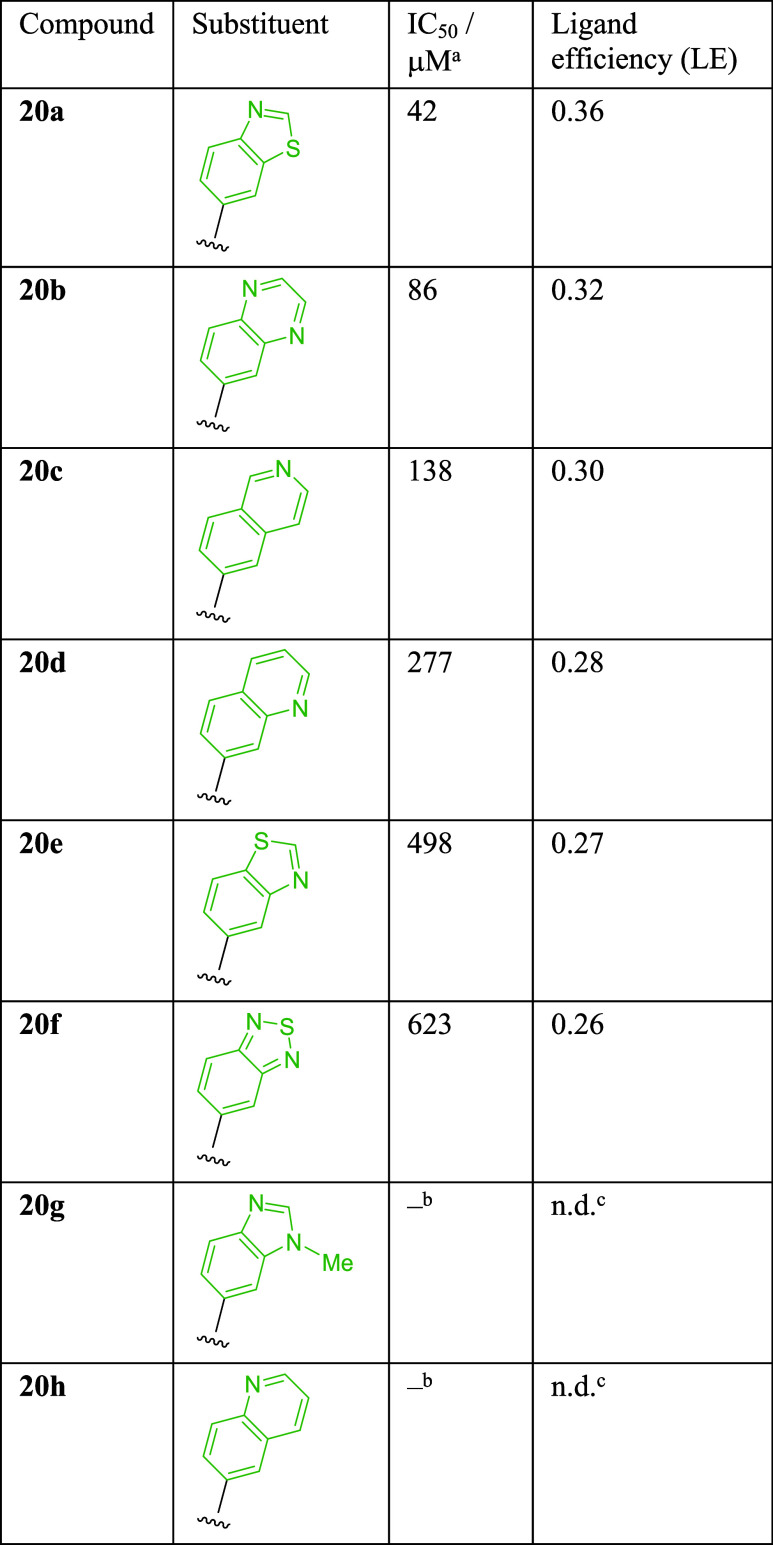
IC_50_ and LE Values for Cyclopentene Acids **20**

a IC_50_ values are mean values ± SD of duplicate measurements.

b No measurable inhibition was observed.

c Not determined.

We also attempted to characterize complexes between Mac1 and the 14 compounds (*cis*-**17a**–**f** and **20a**–**g**) by X-ray crystallography. Using methods described previously,^7^ crystal soaking was carried out at the Diamond XChem facility. Suitable electron density for binding in the adenosine site was obtained for nine compounds: *cis*-**17a** (PDB: 7IJO), *cis*-**17b** (PDB: 7IJP), *cis*-**17c** (PDB: 7IJN), *cis*-**17d** (PDB: 7IJQ), **20a** (PDB: 7IJT/7IJY; binding pose taken from 7IJY), **20b** (PDB: 7IJV), **20c** (PDB: 7IJU), **20d** (PDB: 7IK8), and **20h** (PDB: 7IJZ); the X-ray crystal structures are shown in Figure 3.

**Figure 3:**
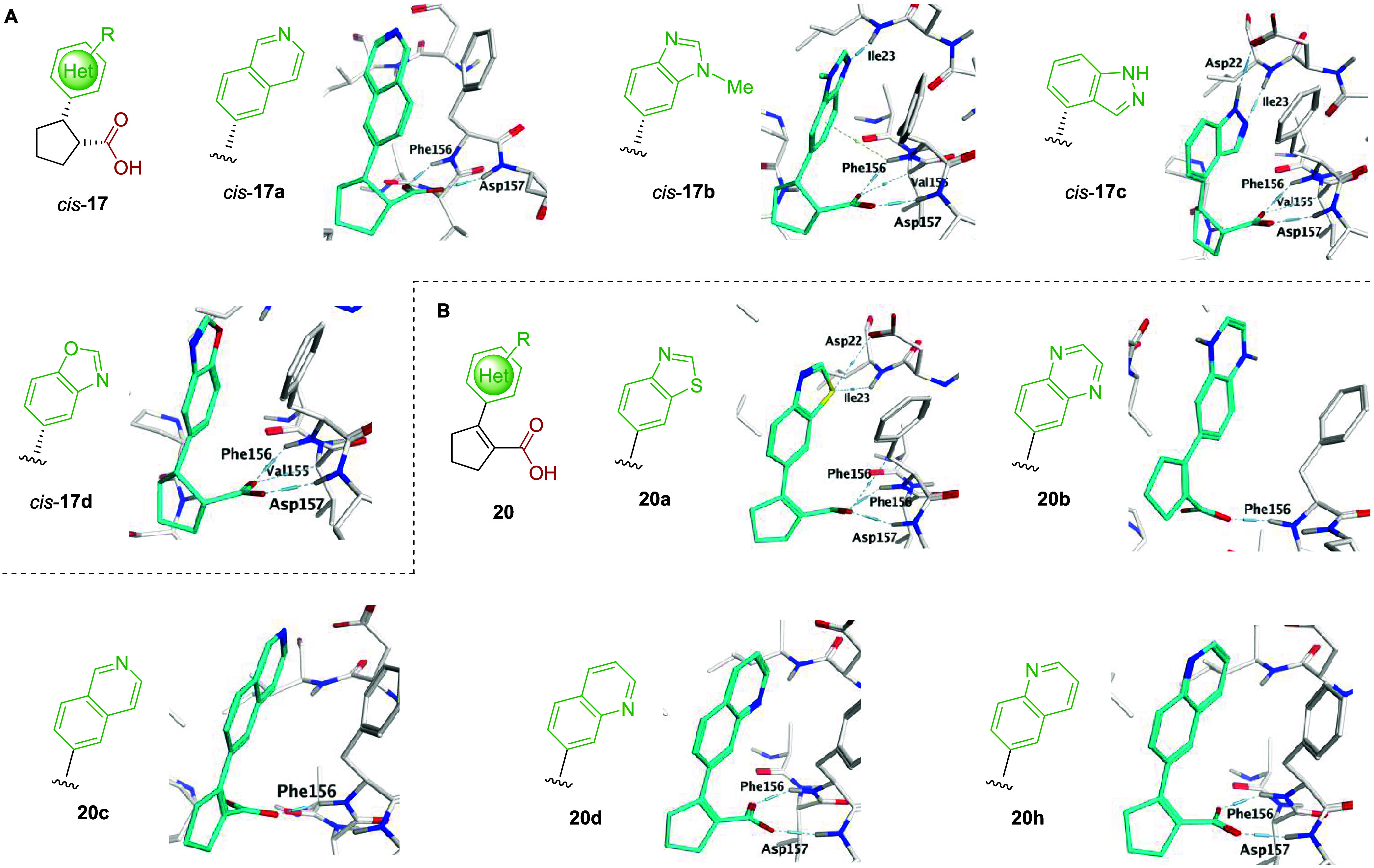
**A.** X-ray crystal structures of fragments bound to Mac1 for *cis-*cyclopentane acids *cis*-**17**. **B.** X-ray crystal structures of fragments bound to Mac1 for cyclopentene acids **20**.

All nine structures showed the expected pose with the carboxylate hydrogen bonding to both backbone NH groups of Phe156 and Asp157 in the oxyanion subsite. There was also some evidence of weak π–π or π-CH interactions with the phenyl ring of Phe156. In addition, *cis*-**17b** (IC_50_ 253 μM), with a *N*-methylbenzimidazole group, showed additional hydrogen bonding to Ile23 and *cis*-**17c** (IC_50_ 297 μM), with an indazole substituent, also showed hydrogen bonding to Asp22 and Ile23. It is notable that seven of these X-ray structures corresponded to compounds with the lowest IC_50_ values (42–297 μM). Within the cyclopentene series **20a**–**h**, there were clear structure–activity relationship (SAR) examples. For the isoquinoline/quinoline series **20c**, **20d**, and **20h**, where the position of the pyridine-like nitrogen was varied, **20c** was the most active inhibitor (IC_50_ 138 μM). A cocrystal X-ray structure was obtained for **20h** even though there was no measurable Mac1 inhibition in the HTRF assay. This likely reflects the difference in the concentration of the two experiments: in the HTRF assay, the top concentration was 2 mM, whereas the X-ray soaking experiments were carried out at 10 mM.

Building on these initial results, a series of *trans*-cyclopentane heteroaryl carboxylic acids **17** was explored next. For this, our focus was on heteroaryl groups that had shown the most promise, i.e., benzothiazole, isoquinoline, regioisomeric quinolines, quinoxaline, and *N*-methylbenzimidazole. To access carboxylic acids *trans*-**17**, two approaches were explored: (i) Ir/Ni-mediated photoredox cross-coupling of alcohols developed by Dong and MacMillan;^40^ (ii) cross-coupling of sulfonyl hydrazones and aryl boronic acids introduced by Barluenga, Valdés, and co-workers^41^ followed by base-mediated epimerization.

The results for the synthesis of heteroaryl cyclopentyl esters *trans*-**22** are shown in Scheme 3. Starting from β-hydroxy esters *cis*-**21**, *trans*-**21** or a diastereomeric mixture of *cis*- and *trans*-**21**, reaction with a benzoxazolium salt generates an adduct which, under Ir­(III) photoredox catalysis, is oxidized and undergoes β-scission of the carbon–oxygen bond to generate a planar carbon-centered radical. This is trapped by a Ni­(II) oxidative addition intermediate and undergoes cross-coupling with heteroaryl bromides to produce heteroaryl esters *trans*-**22**. The observed *trans*-stereoselectivity is precedent in related systems.^40^ Using 6-bromobenzothiazole, starting from either *trans*-**21** or *cis*-**21** gave the same heteroaryl cyclopentyl ester *trans*-**22** in 40 and 34% yields, respectively (due to the planar radical intermediate). Cross-coupling with other heteroaryl bromides gave esters *trans*-**22b**–**f** in 4–62% yields. For *trans*-**22c**–**f**, the starting material was a 78:22 mixture of *trans*- and *cis*-**21**. These reactions, while appearing mostly successful, proved to be very challenging to purify due to the number of reagents used and, in three cases (*trans*-**22c**–**e**), products were isolated along with starting material, a byproduct, or solvent (DMA). The *trans* configuration of ester *trans*-**22b** was assigned as it had different ^1^H and ^13^C NMR spectra from ester *cis*-**22b**, which was synthesized by *cis*-stereospecific hydrogenation of alkene ester **19c** (see SI). The configuration of the other photoredox cross-coupled products *trans*-**22a** and *trans*-**22c**–**f** was assigned by analogy, assignments which were consistent with the observation that each of the diastereomeric esters *cis*/*trans*-**22** and acids *cis*/*trans*-**17** have characteristic, distinguishable signals and trends in δ_H_ values for the C*H*Ar and C*H*CO resonances in their ^1^H NMR spectra (see SI). Finally, ester hydrolysis was carried out on four of the esters (*trans*-**22a**–**c** and *trans*-**22f**) and proceeded smoothly. Thus, heteroaryl cyclopentyl carboxylic acids *trans-* **17a** and **17g**–**i** were isolated in 53–94% yields (Scheme 3). The configuration of *trans*-**17i** was confirmed by X-ray crystallography (CCDC: 2482171) and the configuration of the other three acids was assigned by analogy.

**Scheme 3:**
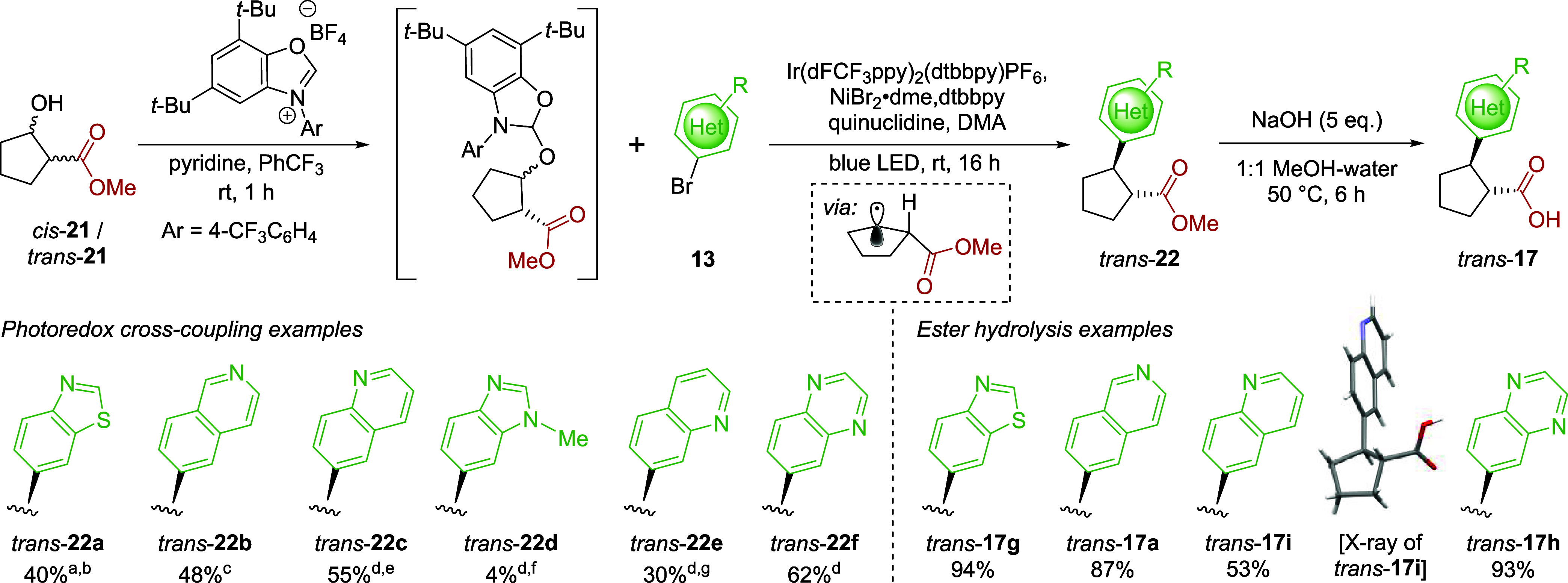
Synthesis of *trans-C*yclopentane Acids Using Photoredox Cross-Coupling

a From *trans*-**21**.
b 34% yield of *trans*-**22** from *cis*-**21**.
c From *cis*-**21**.
d From 78:22 mixture of *trans*- and *cis*-**21**.
e Isolated as a mixture of alcohol **21** and DMA.
f Isolated as a mixture with *N*-methylbenzimidazole.
g Isolated as a mixture with alcohol **21**. Yields (%) are provided below each compound.

Although four of the targeted heteroaryl cyclopentyl carboxylic acids *trans*-**17** were successfully accessed using the photoredox approach, an alternative synthesis was considered due to issues with purification, the lack of a suitable photoredox scale-up setup in our laboratory, and the low yield of *N*-methylbenzimidazole ester *trans*-**22d**. For this, we were attracted to a metal-free cross-coupling method reported by Barluenga, Valdés et al., which combined arylsulfonyl hydrazones and aryl boronic acids under simple basic conditions.^41^ Since the original report, improvements using different arylsulfonyl groups^42^ and new methodology have been disclosed.^43–45^ For cyclic sulfonyl hydrazones, use of a *p*-methoxyphenyl group and Cs_2_CO_3_ had been shown to give the best results, and so these conditions were adopted. Of note, there have been no reports on the stereoselectivity of these types of cross-coupling reactions. However, mechanistically, it is likely that any stereoselectivity will be established in the final protodeboronation step. Due to the presence of the ester in our substrates, it seemed likely that base-mediated epimerization to the *trans* diastereomers could occur. If high *trans*-stereoselectivity was not observed, we planned to access esters *trans*-**22** via epimerization under basic conditions in a subsequent step.

With this background in mind, starting from the β-keto ester, *p*-methoxyphenylsulfonyl hydrazone **24** was prepared. This compound actually existed as a 34:33:33 mixture of readily interconvertible enamide **23**, (*E*)-**24**, and (*Z*)-**24**. Using this mixture, reaction with the benzothiazole boronic acid (HCl salt) in the presence of Cs_2_CO_3_ in dioxane at 110 °C gave an 82:18 mixture of heteroaryl cyclopentenyl esters *trans*- and *cis*-**22a** in 42% yield (Scheme 4). Unfortunately, extension to other heteroaryl groups was lower yielding: 12–26% yields of *trans*-**22c**, **22d**, and *cis-* **22e**. Furthermore, although three examples were *trans*-stereoselective, the *cis* diastereomer, *cis*-**22e**, was in fact the major product for one of the quinoline regioisomers, for no obvious reason. Thus, the Barluenga–Valdés cross-coupling approach was successful in delivering the desired esters **22**, albeit in low yields and with variable stereoselectivity. Purifications were straightforward, although *N*-methylbenzimidazole cyclopentyl ester *trans*-**22d** was isolated with *N*-methylbenzimidazole. Ultimately, we preferred this method to the photoredox approach as it was readily scaled up to the gram-scale.

**Scheme 4:**
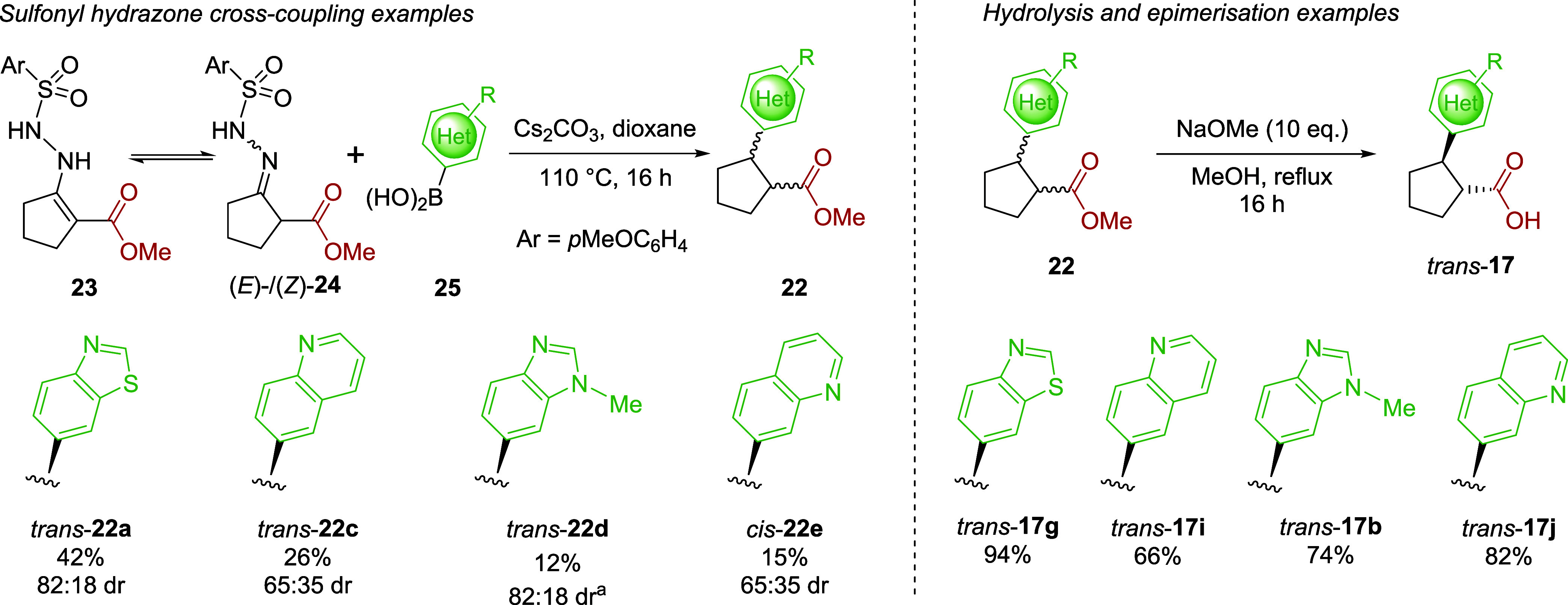
Synthesis of *trans-C*yclopentane Acids Using Sulfonyl Hydrazone Cross-Coupling

a Isolated as a mixture with *N*-methylbenzimidazole. Yields (%) are provided below each compound.

The final step in the route to heteroaryl cyclopentyl carboxylic acids *trans*-**17** was the base-mediated epimerization and concomitant ester hydrolysis. For this, based on precedent in related systems,^46,47^ we found that NaOMe in refluxing MeOH was sufficient to accomplish both steps. Presumably, the NaOMe and/or the MeOH are wet enough to provide the hydroxide required for the hydrolysis step. This method worked well, and the four products (diastereomeric mixtures) from the Barluenga–Valdés cross-coupling reactions were converted into acids *trans*-**17b**, **17g**, **17i**, and **17j** in 66–94% yields (Scheme 4). Two of these (*trans*-**17g** and **17i**) were identical to those formed from the photoredox approach, and the configuration of the other two (*trans*-**17b** and **17j**) was assigned by analogy.

The six heteroaryl cyclopentyl carboxylic acids *trans*-**17** were confirmed as Mac1 inhibitors using the HTRF-based biochemical assay (Table 3), and, in five cases, by X-ray crystal structures of ligand-bound Mac1 complexes (Figure 4). Notably, three compounds exhibited IC_50_ values of <100 μM, with good LE values of 0.32–0.38: benzothiazole *trans*-**17g** (IC_50_ 26 μM), isoquinoline *trans*-**17a** (IC_50_ 61 μM) and quinoline *trans*-**17i** (IC_50_ 75 μM). As with cyclopentyl acids *cis*-**17** and cyclopentene acids **20**, the benzothiazole and isoquinoline heteroaryl groups emerged as the most promising. In five cases, including the three most potent inhibitors, crystal structures clearly showed the targeting of the adenosine site of Mac1 by the heteroaryl moieties: *trans*-**17g** (PDB: 7IJS/7IJX; binding pose taken from 71JX), *trans*-**17a** (PDB: 7IJR/7IJW; binding pose taken from 7IJW), *trans*-**17i** (PDB: 7IK3), *trans*-**17j** (PDB: 7IK4) and *trans*-**17h** (PDB: 7IK0). Similar poses were adopted in each case, consistent with those shown in Figure 3, with the carboxylate hydrogen bonding to Phe156 and Asp157 in the oxyanion subsite; weak π–π and π-CH interactions with Phe156 were also observed. Benzothiazole *trans*-**17g** had the lowest IC_50_ value (26 μM). In terms of SAR, the minor conformational differences due to the *trans*-stereochemistry in cyclopentyl acids *trans*-**17** appears to give some binding benefits: compare benzothiazoles *trans*-**17g** (IC_50_ 26 μM) and **20a** (IC_50_ 42 μM) as well as isoquinolines *trans*-**17a** (IC_50_ 61 μM), *cis*-**17a** (IC_50_ 82 μM) and **20c** (IC_50_ 138 μM). The inhibition exhibited by quinoline *trans*-**17i** (IC_50_ 75 μM) emphasizes this, as there was no measurable inhibition with the corresponding cyclopentene analogue **20h**.

**Figure 4:**
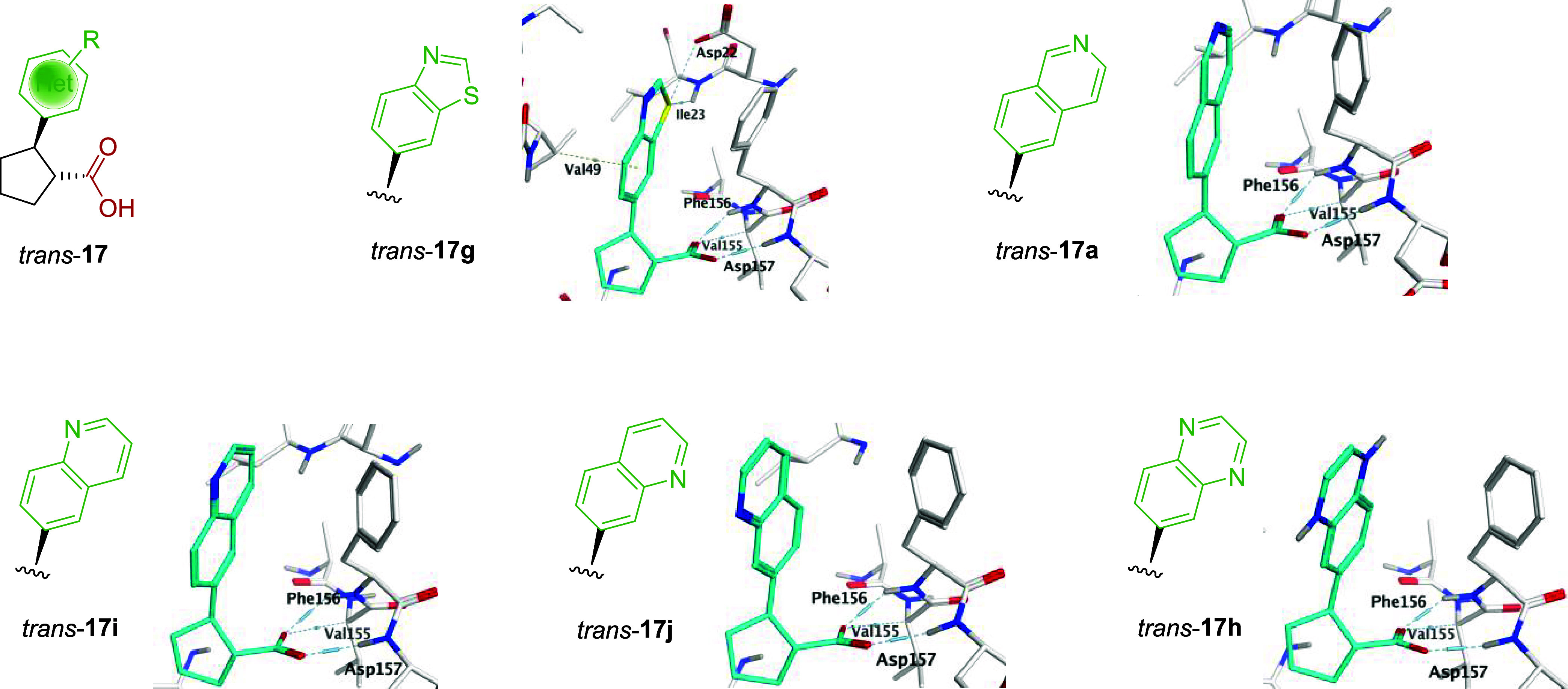
X-ray crystal structures of fragments bound to Mac1 for *trans-*cyclopentane acids *trans*-**17**.

**Table 3:**
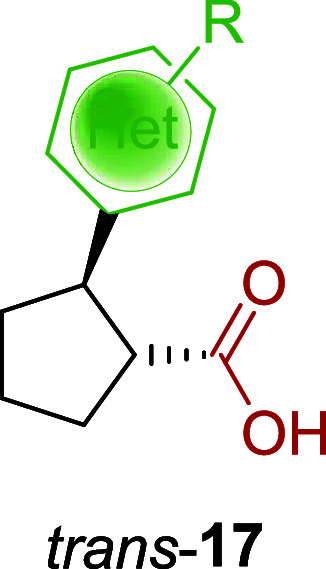

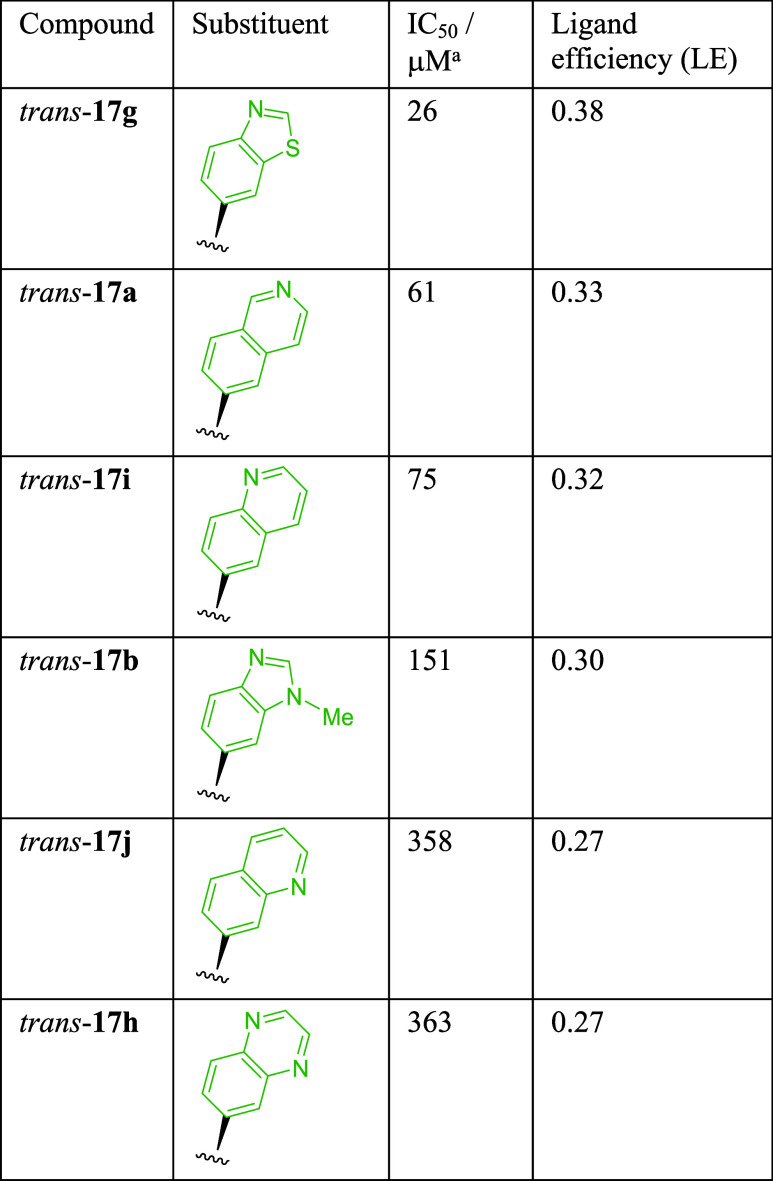
IC_50_ and LE Values for *trans*-Cyclopentane Acids *trans*-**17**

a IC_50_ values are mean values ± SD of duplicate measurements.

From the results presented so far, the benzothiazole substituent was identified as the most promising heteroaryl group. To enhance binding interactions within the adenine subsite, we investigated structural modifications of the two most effective inhibitors from the cyclopentene (**20a**) and *trans*-cyclopentane (*trans*-**17g**) series. Our design strategy focused on enabling these analogues to form additional hydrogen bonds, particularly with residues such as Asp22 and/or Ile23. The X-ray structure of the benzothiazole cyclopentenyl acid **20a**-Mac1 complex (PDB: 7IJT) provided insight for the design, since it involved a water molecule linking the benzothiazole group to Asp22 via hydrogen bonding (Figure 5). As a result, we designed amino benzothiazole **26a**, which, when docked into Mac1, showed its ability to replace the water with its amino group, thereby providing an additional hydrogen bond to the carbonyl group of Asp22 (Figure 5). Similar docking studies were carried out starting from the X-ray structure of the benzothiazole cyclopentyl acid *trans*-**17g-**Mac 1-complex (PDB: 7IJS), our best compound, and this indicated that the amino benzothiazole acid *trans*-**27** should exhibit a similar binding pose to that adopted by **26a** (Figure 5). From a synthetic perspective, it should be easier to synthesize heteroaryl cyclopentene acids (e.g., **26a**) than *trans*-cyclopentyl acids (e.g., *trans*-**27**). Hence, a small family of cyclopentene amino benzothiazoles (**26a**–**c**) was designed, together with a urea moiety (**26d**) (Figure 5). This would allow us to explore the steric and electronic effects of the substituents on the amino groups.

**Figure 5:**
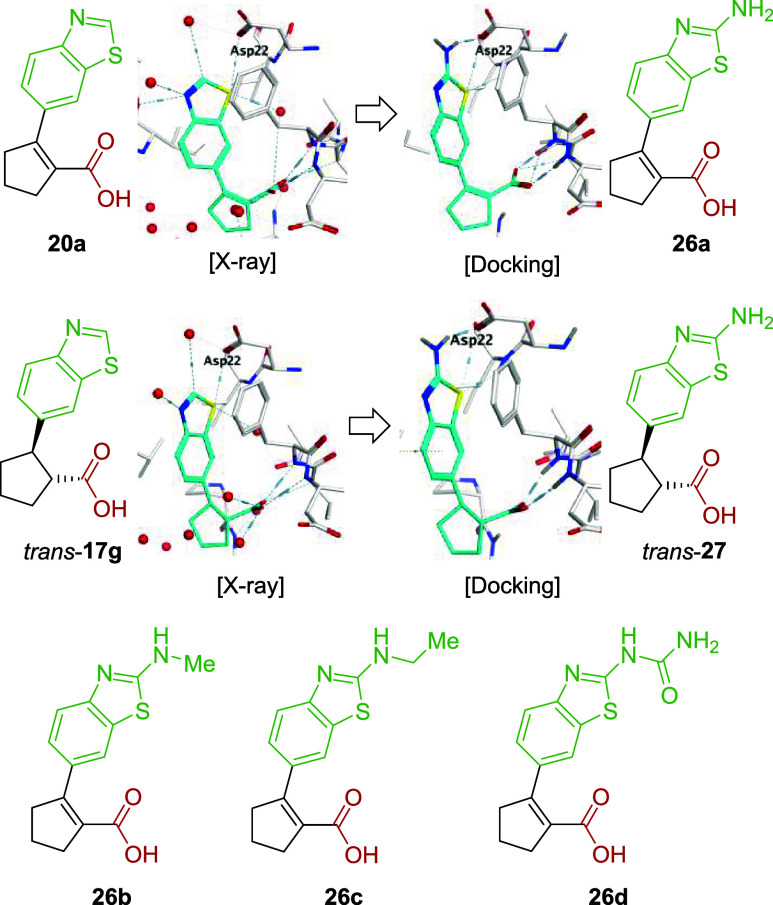
Design of amino benzothiazoles **26a**, *trans*-**27**, and related compounds **26b**–**d**.

The synthesis of the amino benzothiazole family of compounds is summarized in Scheme 5. Miyaura borylation of amino benzothiazole bromide **28** using B_2_pin_2_, KOAc, and Pd­(PPh_3_)_2_Cl_2_ in 1,4-dioxane at 110 °C gave Bpin derivative **29** in 82% yield. The Suzuki–Miyaura cross-coupling of Bpin **29** in the presence of an unprotected amino group has precedent.^48,49^ Indeed, reaction between Bpin **29** and enol triflate methyl ester **18** in the presence of Pd­(PPh_3_)_2_Cl_2_ and aqueous K_2_CO_3_ gave the cross-coupled product **30a** in 80% yield. Ester hydrolysis then gave amino benzothiazole cyclopentenyl acid **26a**. Three other derivatives were prepared from ester **30a**. Reductive amination using an established method^50,51^ was used to prepare *N*-methyl (**30b**) and *N*-ethyl (**30c**) variants. Ester hydrolysis then delivered acids **26b** and **26c**. A reported method^52^ was used to convert ester **30a** into urea derivative **30d**. Thus, reaction of amino benzothiazole ester **30a** with urea at 170 °C gave urea **30d**, which was not isolated but subjected to ester hydrolysis to form urea benzothiazole acid **26d** (40% yield over two steps).

**Scheme 5:**
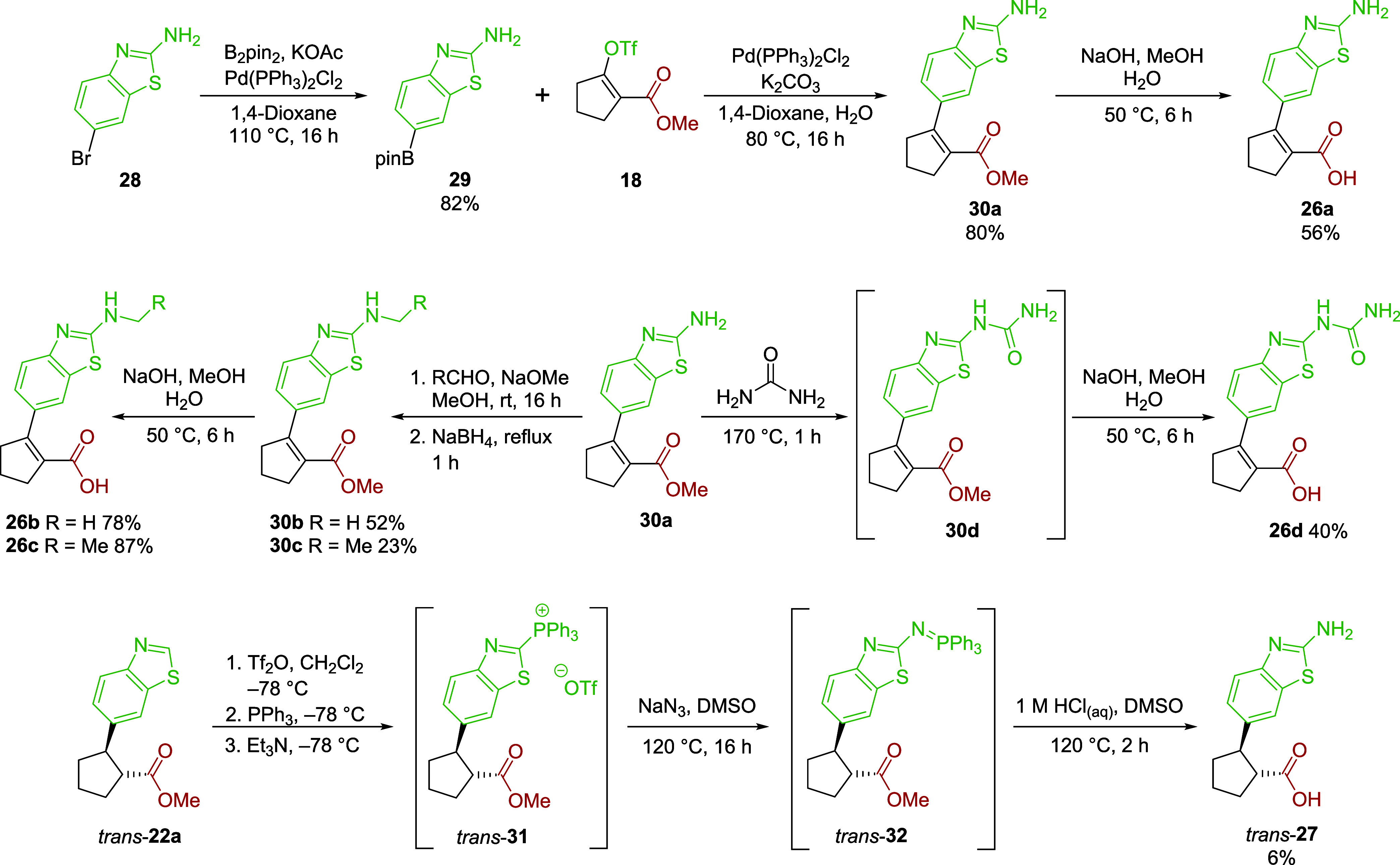
Synthesis of Amino and Urea Benzothiazoles

For the synthesis of amino benzothiazole cyclopentyl acid *trans*-**27**, a different strategy was adopted in which we explored the direct amination of benzothiazole ester *trans*-**22a**. Using a reported method,^53^ phosphonium salt *trans*-**31** was formed from benzothiazole ester *trans*-**22a** using Tf_2_O and PPh_3_. Attempts to isolate phosphonium salt *trans*-**31** by crystallization were unsuccessful and, therefore, crude *trans*-**31** was used in the subsequent S_N_Ar reaction with NaN_3_.^53,54^ Thus, crude *trans*-**31** was reacted with NaN_3_ in DMSO at 120 °C to give the presumed iminophosphorane intermediate *trans*-**32**. Without isolation, the iminophosphorane and the ester were hydrolyzed under acidic conditions^54,55^ to form amino benzothiazole cyclopentyl acid *trans*-**27** (6% yield, unoptimized). Although the overall sequence was low yielding, sufficient quantities of *trans*-**27** were obtained.

In support of our designs, all five amino benzothiazoles **26a**–**d** and *trans*-**27** exhibited low IC_50_ values in the HTRF-based biochemical assay, ranging from 6.74 to 41.8 μM (Table 4). Furthermore, cocrystal structures of all five bound to Mac1 were also determined by X-ray crystallography: **26a** (PDB: 7IK5), **26b** (PDB: 7IK6), **26c** (PDB: 7IK7), **26d** (PDB: 7IK2), and *trans*-**27** (PDB: 7IK1) (Figure 6). The three most potent Mac1 inhibitors were **26a** (IC_50_ 7.24 μM, LE 0.40), **26b** (IC_50_ 6.74 μM, LE 0.38) and **26c** (IC_50_ 7.00 μM, LE 0.36) each of which showed similar Mac1 targeting as the natural ligand ADPr, which has an IC_50_ value of 1.2–2.2 μM. In each case, the predicted hydrogen bonding between the amino group and Asp22 was apparent from the X-ray structures. Similar hydrogen-bonding interactions with Asp22 were also observed with urea **26d** and *trans*-**27**. Thus, using X-ray structure-guided design, we have been able to optimize three Mac1 inhibitors with IC_50_ < 10 μM and lead molecule-like LE values (0.36–0.40).

**Figure 6:**
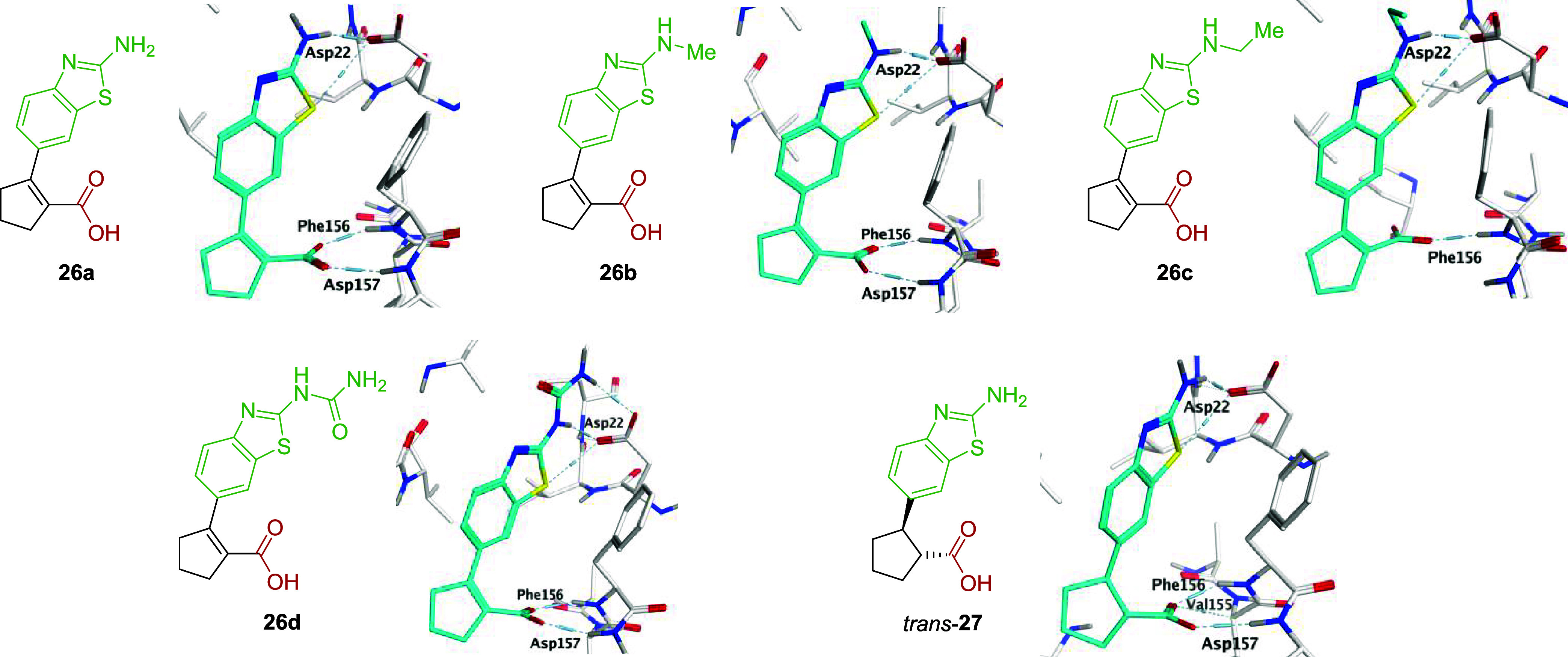
X-ray crystal structures of fragments bound to Mac1 for amino and urea benzothiazoles **26a**–**d** and *trans*-**27**.

**Table 4:**
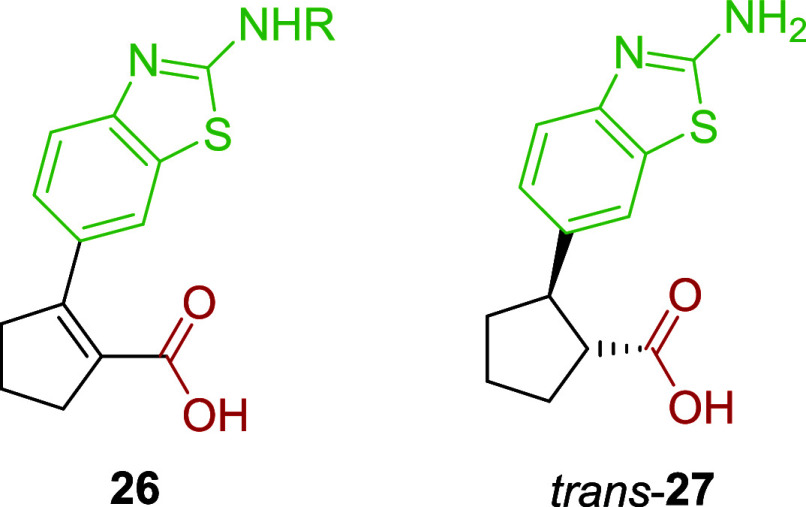

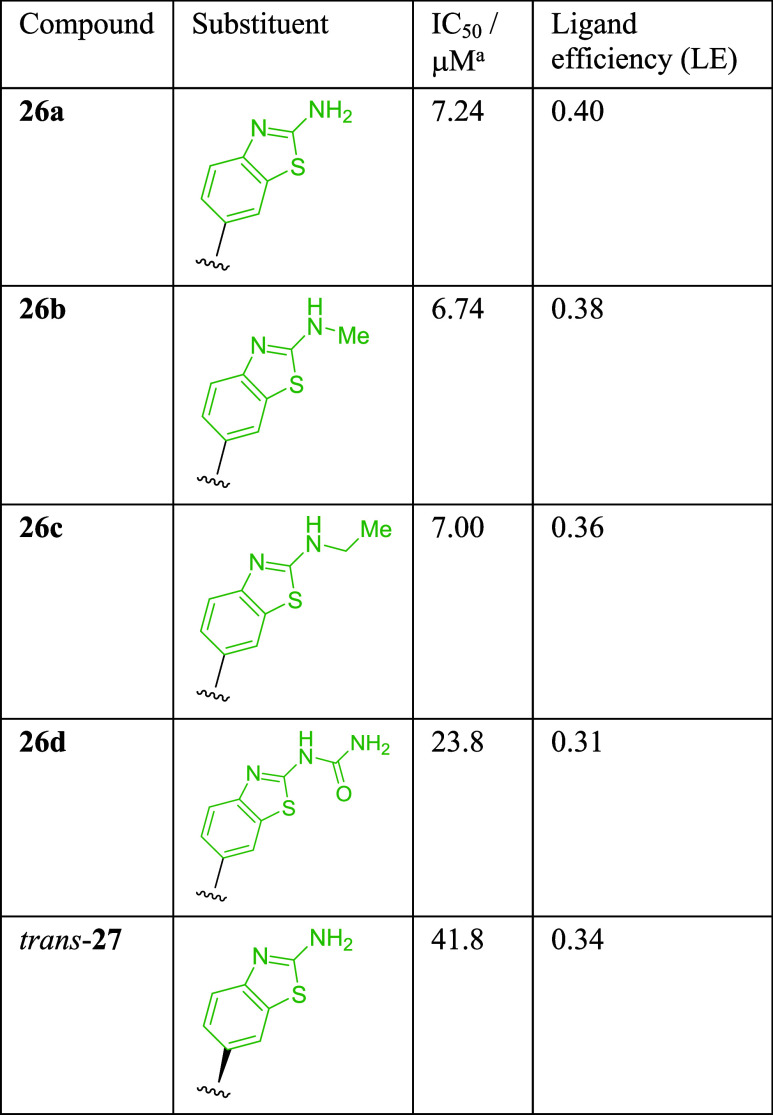
IC_50_ and LE Values for Amino and Urea Benzothiazoles **26a**–**d** and *trans*-**27**

a IC_50_ values are mean values ± SD of duplicate measurements.

Four inhibitors (**26a**–**c** and *trans*-**17g**) were evaluated for inhibition of Mac1 hydrolytic activity using a pre-established *in vitro* AMP-Glo assay^56,57^ with glutamate-ADP-ribose,^58^ previously identified as one of its primary substrates,^59^ used as the assay substrate. Unfortunately, no detectable inhibition was observed for compounds **26a**–**c**/*trans*-**17g** at 1 mM, 500 μM, or 250 μM, as assessed by adenosine monophosphate (AMP) production (see Experimental Section and SI).

We provide a final observation in the X-ray structures of all the benzothiazole-containing compounds, namely **20a** (Figure 3), *trans*-**17g** (Figure 4), **26a**–**d** (Figure 6) and *trans*-**27** (Figure 6). In each case, the benzothiazole sulfur atom was held at a relatively close distance (2.9–3.4 Å) to Asp 22 in the adenine subsite. We initially considered that this was an example of a sulfur σ-hole interaction^60,61^ between the compound’s σ*_C–S_ orbital and the lone pair on the carbonyl oxygen of Asp22. However, it appears that the angle of the lone pairs on the oxygen of Asp22 are far from that expected^61^ for optimum orbital overlap. It is therefore possible that there is some kind of electrostatic interaction bringing the benzothiazole sulfur atom close to Asp22. Alternatively, it could be that the benzothiazole sulfur atom and the carbonyl oxygen of Asp22 are held close to each other by the conformation of the protein for these binding poses.

For Mac1 inhibitors with IC_50_ values <100 μM, the following properties were calculated using bespoke machine learning models in AstraZeneca’s Predictive Insight Platform (see Experimental Section):^62^ AZlogD, clogP, DMSO solubility, topological polar surface area (TPSA), human hepatocyte intrinsic clearance (HH CLint) and human liver microsomes intrinsic clearance (HLM CLint) (Tables 5 and 6). These compounds exhibited AZlogD values from −1.1 to 0.03 as well as good predicted aqueous solubility and metabolic stability (low clearance values). These are in line with those expected for these small-sized carboxylic acid-containing compounds. In particular, the AZlogD values are typical of carboxylic acids, which is a functionality that may present issues with respect to cell permeability. However, given the fragment/lead-like size of the best compounds, high solubility and low TPSA, it is possible that the compounds in Tables 5 and 6 would indeed be cell permeable despite low AZlogD values. Nevertheless, the presence of a carboxylic acid is certainly something that will need to be fully addressed as the series is further developed, and the compounds become larger in size (and therefore less permeable).

**Table 5:** Calculated AZlogD, clogP, Solubility and TPSA for Compounds with IC_50_ Values <100

Compound	AZlogD	clogP	Solubility^a^ (μM)	TPSA^b^ (Å^2^)
*cis*-**17a**	0.15	2.5	1169	50.2
**20a**	–0.61	3.1	563.6	50.2
**20b**	–1.1	2.3	749.9	63.1
*trans*-**17g**	0.03	2.8	879.0	50.2
*trans*-**17a**	0.15	2.5	1169	50.2
*trans*-**17i**	0.11	2.7	1012	50.2
**26a**	–0.68	2.9	857.0	76.2
**26b**	–0.43	3.7	456.0	62.2
**26c**	–0.08	4.2	452.9	62.2
**26d**	–0.76	2.9	545.8	105
*trans*-**27**	0.05	2.5	727.8	76.2

a Dried DMSO Solubility.

b Topological Polar Surface Area.

**Table 6:** Calculated Intrinsic Clearance for Compounds with IC_50_ Values <100

Compound	HH CLint^a^ (μL min^–1^ 10^6^ cells^–1^)	HLM CLint^b^ (μL min^–1^ mg^–1^)
*cis*-**17a**	18.0	6.9
**20a**	9.0	8.8
**20b**	6.4	5.3
*trans*-**17g**	5.7	8.3
*trans*-**17a**	18.0	7.0
*trans*-**17i**	2.6	6.3
**26a**	2.8	6.3
**26b**	2.6	6.4
**26c**	3.1	8.1
**26d**	2.6	5.7
*trans*-**27**	2.2	5.7

a Human Hepatocyte Intrinsic Clearance

b Human Liver Microsomes Intrinsic Clearance.

## Conclusion

In summary, we have reported the design, synthesis, and Mac1 inhibition data of 25 analogues of an initial thiophene cyclopentane crystallographic fragment hit *cis*-**11**. For this, the heteroaryl group and the scaffold (*cis*- and *trans*-cyclopentane and cyclopentene) were varied. The timelines for the early stages of this project were accelerated as fragment hit *cis*-**11** was specifically designed to be synthetically enabled for follow-up work. Thus, heteroaryl group variation was relatively straightforward and allowed us to identify compounds with low μM inhibitory activity on Mac1. These included isoquinoline *cis*-**17a** (IC_50_ 82 μM), benzothiazole **20a** (IC_50_ 42 μM), and quinoxaline **20b** (IC_50_ 86 μM), which, furthermore, had lead compound-like LE values of 0.32–0.36.

With a view to exploring SAR around the stereochemistry of the cyclopentane ring, in particular to compare the *cis-* and *trans-*cyclopentane diastereomers, two routes were successfully explored for the synthesis of cyclopentyl acids *trans*-**17**. The required *trans*-stereoselectivity was achieved in one of two ways. Using MacMillan’s Ir/Ni-mediated photoredox cross-coupling of alcohols, a planar radical intermediate enabled cross-coupling to occur opposite to the sterically hindered ester group. Alternatively, since the Barluenga–Valdés metal-free cross-coupling method showed variable stereoselectivity, heteroaryl cyclopentyl acids *trans*-**17** were accessed using base-mediated enolate formation and epimerization. From these analogues, we identified three μM-potent Mac1 inhibitors: benzothiazole *trans*-**17g** (IC_50_ 26 μM), isoquinoline *trans*-**17a** (IC_50_ 61 μM) and quinoline *trans*-**17i** (IC_50_ 75 μM).

The most potent Mac1 inhibitors we have developed so far were designed based on the amino benzothiazole series, which form direct hydrogen-bonding interactions with Asp22, as predicted by docking studies. This led to our three best Mac1 inhibitors: amino benzothiazole cyclopentenyl acids **26a** (IC_50_ 7.24 μM, LE 0.40), **26b** (IC_50_ 6.74 μM, LE 0.38), and **26c** (IC_50_ 7.00 μM, LE 0.36). Of note, due to the cyclopentene scaffold, these three compounds were readily accessed in high yields, using the Miyaura borylation, Suzuki–Miyaura cross-coupling, and ester hydrolysis reactions that were developed to synthesize most of the compounds in this study. These three compounds exhibit suitable LE values and, as a result, they are primed for further rounds of design, make, test, and analyze to support the ongoing efforts for coronaviral small-molecule therapeutics. One area that will need to be addressed is the exploration of carboxylic acid bioisosteres in order to generate lead-like compounds with a higher chance of cell permeability. It will also be important to focus future efforts on the development of analogues that can inhibit both enzyme binding *and* enzyme activity, as well as successfully target Mac1 in cell culture.

In closing, it is useful to compare and contrast the approach adopted in this project with those utilized by the Fehr and Fraser groups who ultimately developed more potent Mac1 inhibitors on a similar time scale. Both of those groups adopted a fragment merging approach which, in Fraser’s case, was supported by hundreds of X-ray cocrystal structures and exploration of purchasable chemical space through Enamine’s considerable library and synthetic resources. In this way, as summarized in Figure 1, the Fraser group developed the two current best compounds, **AVI-4206** (IC_50_ 20 nM, LE 0.39) and **AVI-6451** (IC_50_ 28 nM, LE 0.42). The Fehr group optimized (*S*)-**4** (IC_50_ 6.1 μM, LE 0.30) from a fragment merging approach, which has a similar profile to our best compounds. Fehr et al. also deployed a HTS approach to develop their current best compound, (*R*,*R*)-**10** (IC_50_ 2.1 μM, LE 0.29). In contrast, our approach focused on a specific fragment optimization on cyclopentane and cyclopentene scaffolds using compounds which sit outside “purchasable chemical space”. Instead, as the initial 3-D fragment hit had been designed to be easily modifiable synthetically, this meant that we were able to improve potency around these specific scaffolds with a relatively small amount of synthetic input (25 compounds synthesized). Although we did not reach the potency levels of Fraser et al.’s Mac1 inhibitors, the work presented herein does demonstrate that a synthetic-led optimization approach in novel fragment space still has a role in the overall efforts toward developing potent Mac1 inhibitors.

## Experimental Section

### Chemistry: General

All final compounds were ≥95% pure by HPLC or ^1^H NMR. The LCMS data and ^1^H/^13^C NMR spectra can be found in the SI. All nonaqueous reactions were carried out under oxygen-free Ar or N_2_, and the reactions were performed in dried glassware. Chemicals and solvents were commercially available. THF, CH_2_Cl_2_, DMSO, and 1,4-dioxane were dried before use. Brine refers to a saturated solution. Water is distilled water. Flash column chromatography was carried out using Fluka Chemie GmbH silica (220–440 mesh). Thin layer chromatography was carried out using commercially available Merck F_254_ aluminum backed silica plates. Proton (400 MHz) and carbon (100.6 MHz) NMR spectra were recorded on a Jeol ECX-400 instrument using an internal deuterium lock at room temperature. For samples recorded in CDCl_3_, chemical shifts are quoted in parts per million relative to CHCl_3_ (δ_H_ 7.26) and CDCl_3_ (δ_C_ 77.16, central line of triplet). For samples recorded in DMSO-*d*_6_, chemical shifts are quoted in parts per million relative to DMSO (δ_H_ 2.50, central line of quintet) and DMSO-*d*_6_ (δ_C_ 39.52, central line of septet). For samples recorded in CD_3_OD, chemical shifts are quoted in parts per million relative to methanol (δ_H_ 3.31, central line of quintet) and methanol (δ_C_ 49.00, central line of septet). Carbon NMR spectra were recorded with broad band proton decoupling and assigned using DEPT experiments. Coupling constants (*J*) are quoted in Hertz (Hz). Data are reported as follows: s = singlet, d = doublet, t = triplet, q = quartet and m = multiplet. Melting points were determined on a Gallenkamp melting point apparatus. Infrared spectra were recorded on a PerkinElmer UATR Two FT-IR spectrometer. Electrospray high and low resonance mass spectra were recorded at room temperature on a Bruker Daltronics microOTOF spectrometer. Compound purity was determined using a Thermo Scientific Vanquish HPLC system with the Acquity UPLC BEH C18 column, 1.7 μm, 2.1 mm * 50 mm. The mobile phases were as follows: A = H_2_O + 0.1% formic acid (v/v) and B = MeOH with a flow rate of 0.3 mL/min at 40 °C, run with a gradient from 5% to 100% B. The purity of the compound was determined as the relative absorbance of the integrated peaks at UV_Vis_3 = 300 nM or UV_Vis_4 = 254 nM.

### General Procedure A: Tandem Miyaura Borylation/Suzuki–Miyaura Cross-Coupling

Bis­(pinacolato)­diboron (1.2 equiv), aryl bromide (1.2 equiv), dppf (0.05 equiv), Pd­(OAc)_2_ (0.05 equiv), and KOAc (2.4 equiv) were added to a pressure tube or round-bottomed flask. The pressure tube or round-bottomed flask was purged with Ar or N_2_ and then dry, degassed 1,4-dioxane (3.5–4 mL) was added. The resulting mixture was stirred and heated at 110 °C for 3 h under Ar or N_2_ in a sealed pressure tube or round-bottomed flask. Then, the pressure tube or round-bottomed flask was removed from the heating block. K_3_PO_4_ (5.0 equiv), enol triflate (1.0 equiv, 0.72–1.0 mmol), dry, degassed 1,4-dioxane (1 mL), and degassed H_2_O (1 mL) were added. The resulting mixture was stirred and heated at 110 °C for 16 h under Ar or N_2_ in a sealed pressure tube or round-bottomed flask. After being allowed to cool to rt, the solids were removed by filtration through a short plug of Celite and washed with EtOAc (30–50 mL). The solvents were evaporated under reduced pressure to give the crude product.

### General Procedure B: Hydrogenation

Alkene (1.0 equiv, 0.128–0.743 mmol) and 10% Pd/C (10% by mass) were added to a flask that was evacuated and backfilled with Ar (3×). Then, MeOH (6 or 10 mL) was added, and the flask was evacuated and backfilled with H_2_ (5×). The resulting mixture was stirred vigorously at rt or 40 °C for 16–72 h under a balloon of H_2_. After cooling to rt (for reactions at 40 °C), the solids were removed by filtration through Celite and washed with MeOH. The filtrate was evaporated under reduced pressure to give the crude product.

### General Procedure C-1: Ester Hydrolysis

NaOH (5.0 equiv) was added to a stirred solution of arylated ester (1.0 equiv, 0.232–2.2 mmol) in MeOH (1.0–3.0 mL) and H_2_O (1.0–3.0 mL) at rt. The resulting solution was stirred and heated at 50 °C for 6–12 h. After being allowed to cool to rt, H_2_O (30 mL) was added. The mixture was extracted with CH_2_Cl_2_ or EtOAc (3 × 10–15 mL). Then, 2 M HCl_(aq)_ was added to the aqueous layer until pH = 3–6 and the mixture was extracted with EtOAc or 10:1 CH_2_Cl_2_-MeOH (2–8 × 10–30 mL). The combined organic extracts were dried (MgSO_4_), and evaporated under reduced pressure to give the carboxylic acid.

### General Procedure C-2: Ester Hydrolysis

2 M NaOH_(aq)_ (3.5 mL) was added dropwise to a stirred solution of arylated ester (0.832–0.883 mmol) in MeOH (3.5 mL) and THF (3.5 mL) at rt under Ar. The resulting solution was stirred and heated at 70 °C for 1 h. After being allowed to cool to rt, H_2_O (15 mL) was added. The mixture was washed with CH_2_Cl_2_ (10 mL). Then, 1 M HCl_(aq)_ (10 mL) was added, and the mixture was extracted with CH_2_Cl_2_ (3 × 50 mL). The organic extracts were dried (Na_2_SO_4_), and evaporated under reduced pressure to give the carboxylic acid.

### General Procedure D: Ni-Catalyzed Photoredox Cross-Coupling of Alcohols

Alcohol (0.9 or 1.6 equiv), benzoxazolium salt (1.36 or 1.6 equiv), pyridine (1.60 equiv), and anhydrous benzotrifluoride (2.5 mL) were added to a screw cap vial equipped with a Suba-Seal and magnetic stirrer bar. The resulting mixture was purged with N_2_ for 2–3 min and then stirred at rt under N_2_ for 1 h to give a suspension of the alcohol adduct in benzotrifluoride (vial 1). Separately, 4,4’-di-*tert*-butyl-2,2’-bipyridine (0.05 equiv), NiBr_2_·dme (0.05 equiv), and anhydrous THF (0.5–1.0 mL) were added to a screw cap vial fitted with a PTFE septum and equipped with a magnetic stirrer bar. The vial was capped, and the resulting suspension was heated with a heat gun until the nickel salt was fully solubilized to give a bright green solution of NiBr_2_(dtbbpy). The solvent was evaporated under reduced pressure. Then, [Ir­(dFCF_3_ppy]_2_(dtbpy)]­PF_6_ (0.0015 equiv), quinuclidine (1.75 equiv), aryl bromide (1.00 equiv, 0.3 mmol), and anhydrous DMA (2.5 mL) were added (vial 2). The suspension of the alcohol adduct in benzotrifluoride (vial 1) was taken up in a syringe with a 19-gauge needle attached. The 19-gauge needle was then removed and replaced with a syringe filter attached to a 21-gauge needle in order to transfer the contents into vial 2. The resulting solution was purged with N_2_ for 20 min. The screw cap was wrapped with Parafilm and stirred approximately 4 cm away from a 60-W blue LED light at rt for 16 h. Then, EtOAc (10–30 mL) and water (30–60 mL) were added, and the two layers were separated. The aqueous layer was extracted with EtOAc (3 × 15–30 mL). Then, the combined organics were washed with brine (3 × 10–20 mL), dried (MgSO_4_), and evaporated under reduced pressure to give the crude product.

### General Procedure E: Miyaura Borylation

Bis­(pinacolato)­diboron (1.0 equiv), aryl bromide (1.0 equiv, 7.0 or 9.6 mmol), dppf (0.05 equiv), Pd­(OAc)_2_ (0.05 equiv), and KOAc (1.2 equiv) were added to a round-bottomed flask. The round-bottomed flask was purged with N_2_, and then dry, degassed 1,4-dioxane (20 mL) was added. The resulting mixture was stirred and heated at 110 °C (heating block temperature) for 2 h under N_2_. After being allowed to cool to rt, the solids were removed by filtration through silica gel and washed with EtOAc (50 mL). The filtrate was diluted with H_2_O (100 mL) and extracted with EtOAc (3 × 50 mL) or 20:1 CH_2_Cl_2_-MeOH (3 × 100 mL). The combined organic extracts were washed with sat. brine (50 mL), dried (MgSO_4_), and evaporated under reduced pressure to give the crude product.

### General Procedure F: Conversion of Aryl Bpin Compounds into Aryl Boronic Acids

A suspension of aryl Bpin (1.0 equiv, 1.15–7.84 mmol) in 6 M HCl_(aq)_ (3–15 mL) was stirred and heated at 120 °C (heating block temperature) for 3 h. After being allowed to cool to rt, the mixture was evaporated under reduced pressure to give the crude product. The crude product was triturated using CH_2_Cl_2_ (3–10 mL) and, after standing for 15 min, the solid was collected by filtration to give the aryl boronic acid hydrochloride salt.

### General Procedure G: Barluenga Reaction between Aryl Boronic Acids and Sulfonyl Hydrazone

Aryl boronic acid (1.15 equiv) and a 34:33:33 mixture of sulfonyl hydrazone **23** and (*Z*)-**24** and (*E*)-**24** (1.0 equiv, 0.92–3.8 mmol) were added to a suspension of Cs_2_CO_3_ (5 equiv) in 1,4-dioxane (5–15 mL) in a 20 mL, 60 or 120 mL pressure tube. N_2_ was bubbled through the resulting mixture for 5 min, and then the pressure tube was sealed with a PTFE cap. The mixture was stirred and heated at 110 °C (oil bath) for 16 h. After being allowed to cool to rt, the mixture was diluted with H_2_O (50–100 mL) and extracted with EtOAc (3 × 30–50 mL). The combined organic extracts were washed with sat. brine (30–50 mL), dried (MgSO_4_), and evaporated under reduced pressure to give the crude product.

### General Procedure H: Ester Hydrolysis and Epimerization of *cis*- and *trans*-Esters into *trans*-Acids

A suspension of *cis*- and *trans*-esters (1.0 equiv, 0.29–0.98 mmol) and NaOMe (10.0 equiv) in MeOH (3–8 mL) was stirred and heated at reflux for 16 h. After being allowed to cool to rt, the mixture was diluted with H_2_O (20–30 mL) and extracted with EtOAc (3 × 10 mL). Then, 2 M HCl_(aq)_ was added to the aqueous layer until pH = 3–5, and the mixture was extracted with EtOAc (3 × 15 mL). The combined organic extracts were washed with sat. brine (10–20 mL), dried (MgSO_4_), and evaporated under reduced pressure to give the carboxylic acid.
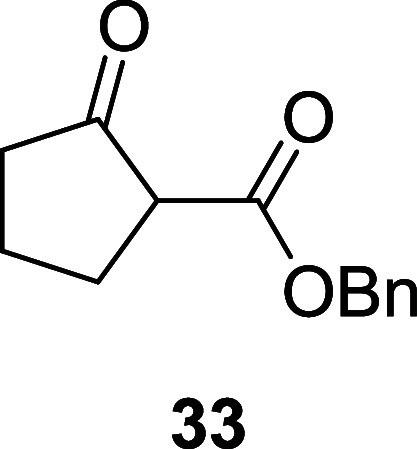


### Benzyl 2-Oxocyclopentane-1-carboxylate **33**

Methyl cyclopentanone-2-carboxylate (3.2 mL, 25.6 mmol, 1.0 equiv), benzyl alcohol (4.0 mL, 38.4 mmol, 1.5 equiv), DMAP (156 mg, 1.28 mmol, 0.05 equiv) and cyclohexane (25 mL) were added to a flask connected to a Dean–Stark apparatus with a cyclohexane trap. The apparatus was purged with Ar. Then, the resulting solution was stirred and heated at 120 °C for 24 h. After being allowed to cool to rt, the solvent was evaporated under reduced pressure to give the crude product. Purification by flash column chromatography on silica with 9:1 toluene-EtOAc as eluent gave benzyl ester **33** (5.21 g, 93%) as a pink oil, *R*_F_ (9:1 toluene-EtOAc) 0.51; IR (ATR) 1753 (C = O, ester), 1722 (C = O, ketone), 1179, 1108 cm^–1^; ^1^H NMR (400 MHz, CDCl_3_) δ 7.30–7.28 (m, 5H, Ph), 5.07 (s, 2H, OCH_2_), 3.10 (dd, *J* = 9.0, 9.0 Hz, 1H, CHC­(O)), 2.26–2.10 (m, 4H, CH), 2.05–1.94 (m, 1H, CH), 1.79–1.66 (m, 1H, CH); ^13^C NMR (100.6 MHz, CDCl_3_) δ 212.2 (C = O, ketone), 169.4 (C = O, ester), 135.8 (*ipso*-Ph), 128.7 (Ph), 128.3 (Ph), 128.1 (Ph), 67.0 (OCH_2_), 54.8 (CH), 38.1 (CH_2_), 27.5 (CH_2_), 21.0 (CH_2_); HRMS (ESI) *m*/*z* calcd for C_13_H_14_O_3_ (M + Na)^+^ 241.0835, found 241.0835 (+1.6 ppm error). Spectroscopic data consistent with those reported in the literature.^63^ Lab book reference WTWB-24.

### Benzyl 2-(((Trifluoromethyl)­sulfonyl)­oxy)­cyclopent-1-ene-1-carboxylate **15**

*N*-Ethyl-*N*,*N*-diisopropylamine (24 mL, 137 mmol, 5.0 equiv) was added dropwise to a stirred solution of benzyl ester **S1** (5.3 mL, 27.5 mmol, 1.0 equiv) in CH_2_Cl_2_ (180 mL) at −78 °C under Ar. The resulting solution was stirred at −78 °C for 10 min. Then, trifluoromethanesulfonic anhydride (5.6 mL, 33.0 mmol, 1.2 equiv) was added dropwise over 15 min. The solution was allowed to warm to rt and then stirred at rt for 16 h. H_2_O (100 mL) and 5% citric acid_(aq)_ (100 mL) were added, and the aqueous layer was extracted with CH_2_Cl_2_ (3 × 100 mL). The combined organic extracts were dried (Na_2_SO_4_), and evaporated under reduced pressure to give the crude product. Purification by flash column chromatography on silica with 9:1 hexane-Et_2_O as eluent gave enol triflate **15** (8.98 g, 93%) as a yellow oil, *R*_F_ (9:1 hexane-Et_2_O) 0.19; IR (ATR) 1719 (C = O), 1425, 1205, 1138 cm^–1^; ^1^H NMR (400 MHz, CDCl_3_) δ 7.42–7.30 (m, 5H, Ar), 5.24 (s, 2H, OCH_2_), 2.79–2.68 (m, 4H, = CCH_2_), 2.01 (tt, *J* = 8.0, 8.0 Hz, 2H, CH_2_C*H*_2_CH_2_); ^13^C NMR (100.6 MHz, CDCl_3_) δ 162.2 (C = O), 154.3 (=C–O) 135.5 (*ipso*-Ar), 128.7 (Ar), 128.7 (Ar), 128.5 (Ar), 123.1 (=*C*CO_2_Bn), 118.4 (q, *J* = 320.0 Hz, CF_3_), 66.8 (OCH_2_), 32.8 (=C*CH*_2_), 29.3 (=C*CH*_2_), 18.9 (CH_2_ *CH*_2_CH_2_); ^19^F NMR (376.5 MHz, CDCl_3_) δ −74.3 (s, CF_3_); HRMS (ESI) *m*/*z* calcd for C_14_H_13_F_3_O_5_S (M + Na)^+^ 373.0328, found 373.0328 (+2.9 ppm error). Lab book reference WTWB-23.

### Benzyl 2-(Isoquinolin-6-yl)­cyclopent-1-ene-1-carboxylate **16a**

Using general procedure A in a sealed pressure tube, bis­(pinacolato)­diboron (305 mg, 1.20 mmol, 1.2 equiv), 6-bromoisoquinoline (250 mg, 1.20 mmol, 1.2 equiv), dppf (28 mg, 0.050 mmol, 0.05 equiv), Pd­(OAc)_2_ (11 mg, 0.050 mmol, 0.05 equiv) and KOAc (236 mg, 2.40 mmol, 2.4 equiv) in dry, degassed 1,4-dioxane (4 mL) and then K_3_PO_4_ (1.06 g, 5.00 mmol, 5.0 equiv) and enol triflate **15** (350 mg, 1.00 mmol, 1.0 equiv) in dry, degassed 1,4-dioxane (1 mL) and degassed H_2_O (1 mL) gave the crude product. Purification by flash column chromatography on silica with 70:30 hexane-EtOAc as eluent gave benzyl ester **16a** (153 mg, 46%) as an orange oil that solidified on standing, mp 69–70 °C; *R*_F_ (7:3 hexane-EtOAc) 0.23; IR (ATR) 3038, 2958, 2850, 1711 (C = O), 1623, 1488, 1456, 1389, 1377, 1259, 1233, 1198, 1135, 1032, 937, 883, 829, 691, 735, 574, 474 cm^–1^; ^1^H NMR (400 MHz, CDCl_3_) δ 9.20 (s, 1H, Ar), 8.50 (d, *J* = 6.0 Hz, 1H, Ar), 7.82 (d, *J* = 8.5 Hz, 1H, Ar), 7.67 (s, 1H, Ar), 7.53 (d, *J* = 6.0 Hz, 1H, Ar), 7.47 (dd, *J* = 8.5, 1.5 Hz, 1H, Ar), 7.19 (dd, *J* = 7.5, 7.5 Hz, 1H, Ar), 7.11 (dd, *J* = 7.5, 7.5 Hz, 2H, Ar), 6.99 (d, *J* = 7.5 Hz, 1H, Ar), 5.04 (s, 2H, CH_2_O), 3.00–2.86 (m, 4H, = CCH_2_), 2.06 (tt, *J* = 7.5, 7.5 Hz, 2H, CH_2_); ^13^C NMR (100.6 MHz, CDCl_3_) δ 165.5 (C = O), 153.2 (=*C*Ar), 152.2 (Ar), 143.3 (Ar), 139.7 (Ar), 135.6 (Ar), 135.5 (Ar), 131.0 (=*C*CO_2_Bn), 128.3 (Ar), 128.1 (Ar), 128.02 (Ar), 127.98 (C), 127.6 (Ar), 127.0 (Ar), 124.9 (Ar), 120.7 (Ar), 66.1 (CH_2_O), 40.7 (=C*C*H_2_), 35.2 (=C*C*H_2_), 22.1 (CH_2_ *C*H_2_CH_2_); HRMS (ESI) *m*/*z* calcd for C_22_H_19_NO_2_ (M + H)^+^ 330.1489, found 330.1489 (−0.1 ppm error). Lab book reference JRD_XI_44.

### Benzyl 2-(1-Methyl-1*H*-benzo­[*d*]­imidazol-6-yl)­cyclopent-1-ene-1-carboxylate **16b**

Using general procedure A in a sealed pressure tube, bis­(pinacolato)­diboron (305 mg, 1.20 mmol, 1.2 equiv), 6-bromo-1-methyl-1*H*-benzo­[*d*]­imidazole (253 mg, 1.20 mmol, 1.2 equiv), dppf (28 mg, 0.050 mmol, 0.05 equiv), Pd­(OAc)_2_ (11 mg, 0.050 mmol, 0.05 equiv) and KOAc (236 mg, 2.40 mmol, 2.4 equiv) in dry, degassed 1,4-dioxane (4 mL) and then K_3_PO_4_ (1.06 g, 5.00 mmol, 5.0 equiv) and enol triflate **15** (350 mg, 1.00 mmol, 1.0 equiv) in dry, degassed 1,4-dioxane (1 mL) and degassed H_2_O (1 mL) gave the crude product. Purification by flash column chromatography on silica with 50:50 THF-CH_2_Cl_2_ as eluent, and then a second purification with EtOAc as eluent gave benzyl ester **16b** (284 mg, 85%) as a pale orange gum, *R*_F_ (EtOAc) 0.15; IR (ATR) 2946, 1696 (C = O), 1621, 1498, 1457, 1345, 1289, 1249, 1204, 1118, 1032, 909, 817, 729, 697 cm^–1^; ^1^H NMR (400 MHz, CDCl_3_) δ 7.82 (s, 1H, Ar), 7.70 (d, *J* = 8.5 Hz, 1H, Ar), 7.31 (s, 1H, Ar), 7.23–7.17 (m, 2H, Ar), 7.17–7.11 (dd, *J* = 7.5, 7.5 Hz, 1H, Ar), 7.01 (d, *J* = 7.5 Hz, 1H, Ar), 5.05 (s, 2H, CH_2_O), 3.61 (s, 3H, Me), 2.99–2.81 (m, 4H, = CCH_2_), 2.01 (tt, *J* = 7.5, 7.5 Hz, 2H, CH_2_); ^13^C NMR (100.6 MHz, CDCl_3_) δ 166.1 (C = O), 154.5 (=*C*Ar), 144.1 (Ar), 143.4 (*ipso*-Ar), 135.9 (*ipso*-Ar), 134.1 (Ar), 132.1 (Ar), 128.8 (=*C*CO_2_Bn), 128.2 (Ar), 127.9 (Ar), 127.8 (Ar), 122.2 (Ar), 119.4 (Ar), 109.2 (Ar), 65.8 (CH_2_O), 41.0 (=C*C*H_2_), 35.2 (=C*C*H_2_), 30.9 (Me), 22.0 (CH_2_ *C*H_2_CH_2_); HRMS (ESI) *m*/*z* calcd for C_21_H_20_N_2_O_2_ (M + H)^+^ 333.1598, found 333.1603 (−1.8 ppm error). Lab book reference JRD_XI_39
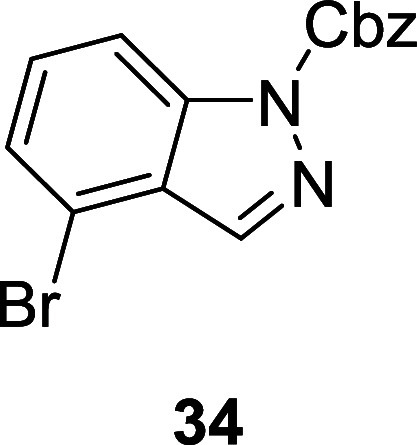


### Benzyl 4-Bromo-1*H*-indazole-1-carboxylate **34**

NaH (305 mg of a 60% suspension in mineral oil, 7.61 mmol, 1.5 equiv) was added to a stirred solution of 4-bromo-1H-indazole (1.00 g, 5.08 mmol, 1.0 equiv) in THF (25 mL) at 0 °C under Ar. The resulting mixture was stirred at 0 °C for 1 h. Then, Cbz-Cl (0.87 mL, 6.09 mmol, 1.2 equiv) was added dropwise, and the solution was stirred at rt for 16 h. H_2_O (50 mL) was carefully added, and the mixture was extracted with EtOAc (3 × 50 mL). The combined organic extracts were washed with saturated NaHCO_3(aq)_ (50 mL), dried (Na_2_SO_4_), and evaporated under reduced pressure to give the crude product. Purification by recrystallization from hexane (100 mL) gave aryl bromide **34** (1.36 g, 81%) as an off-white, crystalline solid, mp 88–89 °C; IR (ATR) 1728 (C = O), 1419, 1396, 1267, 1171 cm^–1^; ^1^H NMR (400 MHz, CDCl_3_) δ 8.20 (s, 1H, Ar), 8.16 (d, *J* = 8.5 Hz, 1H, Ar), 7.56–7.51 (m, 2H, Ar), 7.47 (d, *J* = 8.0 Hz, 1H, Ar), 7.44–7.34 (m, 4H, Ar), 5.55 (s, 2H, OCH_2_); ^13^C NMR (100.6 MHz, CDCl_3_) δ 150.5 (C = O), 140.7 (Ar), 140.0 (Ar), 134.7 (Ar), 130.3 (Ar), 129.1 (Ar), 129.0 (Ar), 128.9 (Ar), 127.1 (Ar), 127.0 (Ar), 114.7 (Ar), 113.7 (Ar), 69.8 (OCH_2_); HRMS (ESI) *m*/*z* calcd for C_15_H_11_^79^BrN_2_O_2_ (M­(^79^Br) + Na)^+^ 352.9896, found 352.9896 (−0.8 ppm error). Lab book reference WTWB-27.

### Benzyl 2-(1*H*-Indazol-4-yl)­cyclopent-1-ene-1-carboxylate **16c**

Using general procedure A in a sealed pressure tube, bis­(pinacolato)­diboron (305 mg, 1.20 mmol, 1.2 equiv), benzyl 4-bromo-1*H*-indazole-1-carboxylate **S2** (397 mg, 1.20 mmol, 1.2 equiv), dppf (28 mg, 0.0500 mmol, 0.05 equiv), Pd­(OAc)_2_ (11 mg, 0.0500 mmol, 0.05 equiv) and KOAc (236 mg, 2.40 mmol, 2.4 equiv) in dry, degassed 1,4-dioxane (4 mL) and then K_3_PO_4_ (1.06 g, 5.00 mmol, 5.0 equiv) and enol triflate **15** (350 mg, 1.00 mmol, 1.0 equiv) in dry, degassed 1,4-dioxane (1 mL) and degassed H_2_O (1 mL) gave the crude product. Purification by flash column chromatography on silica with 60:40 hexane-EtOAc as eluent gave benzyl ester **16c** (230 mg, 72%) as a yellow oil, *R*_F_ (6:4 hexane-EtOAc) 0.17; IR (ATR) 3310 (br, NH), 2954, 1698 (C = O), 1340, 1238, 1134 cm^–1^; ^1^H NMR (400 MHz, CDCl_3_) δ 11.39 (br s, 1H, NH), 7.98 (s, 1H, Ar), 7.40–7.24 (m, 2H, Ar), 7.21–7.13 (m, 3H, Ar), 7.02 (d, *J* = 7.0 Hz, 1H, Ar), 6.90–6.83 (m, 2H, Ar), 4.96 (s, 2H, OCH_2_), 2.99 (t, *J* = 7.5 Hz, 4H, = C*CH*_2_), 2.11 (tt, *J* = 7.5, 7.5 Hz, 2H, CH_2_ *CH*_2_CH_2_); ^13^C NMR (100.6 MHz, CDCl_3_) δ 165.7 (C = O), 152.7 (=C), 135.6 (Ar), 131.2 (Ar), 131.0 (=C), 129.0 (Ar), 128.6 (Ar), 128.3 (Ar), 127.9 (Ar), 127.8 (Ar), 126.5 (Ar), 119.7 (Ar), 109.6 (Ar), 66.0 (OCH_2_), 41.1 (=C*CH*_2_), 34.9 (=C*CH*_2_), 22.3 (CH_2_ *CH*_2_CH_2_); HRMS (ESI) *m*/*z* calcd for C_20_H_18_N_2_O_2_ (M + Na)^+^ 341.1260, found 341.1260 (+0.3 ppm error). Lab book reference WTWB-1-29.

### Benzyl 2-(Benzo­[*d*]­oxazol-5-yl)­cyclopent-1-ene-1-carboxylate **16d**

Using general procedure A in a sealed pressure tube, bis­(pinacolato)­diboron (305 mg, 1.20 mmol, 1.2 equiv), 5-bromobenzo­[*d*]­oxazole (238 mg, 1.20 mmol, 1.2 equiv), dppf (28 mg, 0.050 mmol, 0.05 equiv), Pd­(OAc)_2_ (11 mg, 0.050 mmol, 0.05 equiv) and KOAc (236 mg, 2.40 mmol, 2.4 equiv) in dry, degassed 1,4-dioxane (4 mL) and then K_3_PO_4_ (1.06 g, 5.00 mmol, 5.0 equiv) and enol triflate **15** (350 mg, 1.00 mmol, 1.0 equiv) in dry, degassed 1,4-dioxane (1 mL) and degassed H_2_O (1 mL) gave the crude product. Purification by flash column chromatography on silica with 90:10 to 80:20 hexane-EtOAc as eluent and then a second purification with 60:40 hexane-Et_2_O as eluent gave benzyl ester **16d** (42 mg, 46%) as a pale-yellow solid, mp 80–82 °C, *R*_F_ (6:4 hexane-Et_2_O) 0.20; IR (ATR) 2955, 1699 (C = O), 1517, 1474, 1345, 1240, 1202, 1115, 1064, 812, 766, 697 cm^–1^; ^1^H NMR (400 MHz, CDCl_3_) δ 8.09 (s, 1H, Ar), 7.72 (d, *J* = 1.5 Hz, 1H, Ar), 7.46 (d, *J* = 8.5 Hz, 1H, Ar), 7.33 (dd, *J* = 8.5, 1.5 Hz, 1H, Ar), 7.27–7.19 (m, 3H, Ar), 7.15–7.08 (m, 2H, Ar), 5.07 (s, 2H, CH_2_O), 2.95–2.88 (m, 4H, = CCH_2_), 2.04 (tt, *J* = 7.5, 7.5 Hz, 2H, CH_2_); ^13^C NMR (100.6 MHz, CDCl_3_) δ 165.8 (C = O), 153.8 (=*C*Ar), 152.9 (Ar), 149.6 (Ar), 139.9 (Ar), 135.9 (Ar), 134.1 (Ar), 129.6 (=*C*CO_2_Bn), 128.4 (Ar), 128.1 (Ar), 128.0 (Ar), 125.9 (Ar), 119.8 (Ar), 110.3 (Ar), 66.0 (CH_2_O), 41.0 (=C*C*H_2_), 35.1 (=*C*CH_2_), 22.0 (CH_2_ *CH*_2_CH_2_); HRMS (ESI) *m*/*z* calcd for C_20_H_17_NO_3_ (M + H)^+^ 320.1281, found 320.1280 (+0.5 ppm error). Lab book reference JRD_XI_47.

### Benzyl 2-(5-Fluorobenzofuran-7-yl)­cyclopent-1-ene-1-carboxylate **16e**

Using general procedure A in a sealed pressure tube, bis­(pinacolato)­diboron (305 mg, 1.20 mmol, 1.2 equiv), 7-bromo-5-fluorobenzofuran (258 mg, 1.20 mmol, 1.2 equiv), dppf (28 mg, 0.0500 mmol, 0.05 equiv), Pd­(OAc)_2_ (11 mg, 0.0500 mmol, 0.05 equiv) and KOAc (236 mg, 2.40 mmol, 2.4 equiv) in dry, degassed 1,4-dioxane (4 mL) and then K_3_PO_4_ (1.06 g, 5.00 mmol, 5.0 equiv) and enol triflate **15** (350 mg, 1.00 mmol, 1.0 equiv) in dry, degassed 1,4-dioxane (1 mL) and degassed H_2_O (1 mL) gave the crude product. Purification by flash column chromatography on silica with 95:5 hexane:EtOAc as eluent gave benzyl ester **16e** (326 mg, 90%) as an off-white solid, mp 91–93 °C; *R*_F_ (95:5 hexane:EtOAc) 0.33; IR (ATR) 1700 (C = O), 1406, 1325, 1264, 1156, 1097 cm^–1^; ^1^H NMR (400 MHz, CDCl_3_) δ 7.52 (d, *J* = 2.0 Hz, 1H, Ar), 7.27–7.23 (m, 3H, Ph), 7.19 (dd, *J* = 8.0, 2.5 Hz, 1H, Ar), 7.01–6.98 (m, 2H, Ph), 6.95 (dd, *J* = 10.0, 2.5 Hz, 1H, Ar), 6.70 (d, *J* = 2.0 Hz, 1H, Ar), 5.01 (s, 2H, OCH_2_), 3.01–2.95 (m, 4H, = C*CH*_2_), 2.11 (tt, *J* = 8.0, 8.0 Hz, 2H, CH_2_ *CH*_2_CH_2_); ^13^C NMR (100.6 MHz, CDCl_3_) δ 165.6 (C = O), 158.8 (d, *J* = 238.5 Hz, *ipso-*Ar), 148.2 (=C), 147.0 (d, *J* = 2.0 Hz, *ipso*-Ar), 146.4 (Ar), 135.8 (Ph), 132.7 (=C), 128.4 (Ph), 128.2 (d, *J* = 11.0 Hz, *ipso*-Ar), 128.0 (Ph), 127.9 (Ph), 122.7 (d, *J* = 10.0 Hz, *ipso*-Ar), 111.4 (d, *J* = 28.0 Hz, Ar), 107.0 (d, *J* = 4.5 Hz, Ar), 106.2 (d, *J* = 25.5 Hz, Ar), 66.1 (OCH_2_), 39.7 (=C*CH*_2_), 35.0 (=C*CH*_2_), 22.1 (CH_2_ *CH*_2_CH_2_); ^19^F NMR (376.5 MHz, CDCl_3_) δ−121.3 (dd, *J* = 10.0, 8.0 Hz, F); HRMS (ESI) *m*/*z* calcd for C_21_H_17_FO_3_ (M + K)^+^ 375.0793, found 375.0793 (−0.1 ppm error). Lab book reference WTWB-1-53
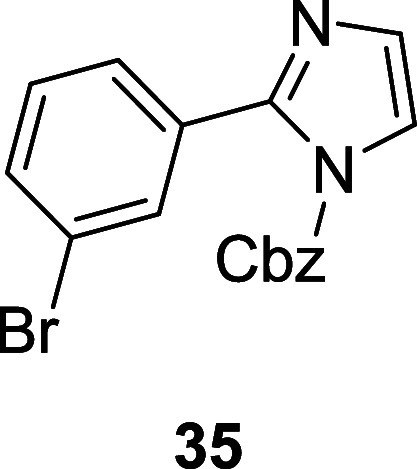
.

### Benzyl 2-(3-Bromophenyl)-1*H*-imidazole-1-carboxylate **35**

NaH (134 mg of a 60% suspension in mineral oil, 3.36 mmol, 1.5 equiv) was added to a stirred solution of 2-(3-bromophenyl)-1*H*-imidazole (500 mg, 2.24 mmol, 1.0 equiv) in THF (15 mL) at 0 °C under Ar. The resulting mixture was stirred at 0 °C for 1 h. Then, Cbz-Cl (0.38 mL, 2.69 mmol, 1.2 equiv) was added dropwise, and the solution was stirred at rt for 16 h. H_2_O (50 mL) was carefully added, and the mixture was extracted with EtOAc (3 × 50 mL). The combined organic extracts were washed with saturated NaHCO_3(aq)_ (50 mL), dried (Na_2_SO_4_), and evaporated under reduced pressure to give the crude product. Purification by recrystallization from hexane (50 mL) gave aryl bromide **35** (491 mg, 61%) as a white, crystalline solid, mp 63–64 °C; IR (ATR) 1753 (C = O), 1393, 1307, 1291, 1175 cm^–1^; ^1^H NMR (400 MHz, CDCl_3_) δ 7.71 (dd, *J* = 2.0, 2.0 Hz, 1H, Ar), 7.54 (d, *J* = 2.0 Hz, 1H, Ar), 7.51 (ddd, *J* = 8.0, 2.0, 2.0 Hz, 1H, Ar), 7.46 (ddd, *J* = 8.0, 2.0, 2.0 Hz, 1H, Ar), 7.38–7.33 (m, 3H, Ph), 7.29–7.24 (m, 2H, Ph) 7.21 (dd, *J* = 8.0, 8.0 Hz, 1H, Ar), 7.07 (d, *J* = 2.0 Hz, 1H, Ar), 5.28 (s, 2H, OCH_2_); ^13^C NMR (100.6 MHz, CDCl_3_) δ 149.1 (C = O), 147.8 (Ar), 133.8 (Ar), 133.3 (Ar), 132.6 (Ar), 132.4 (Ar), 129.44 (Ar), 129.35 (Ar), 129.2 (Ar), 128.9 (Ar), 128.9 (Ar), 128.3 (Ar), 121.9 (Ar), 120.1 (Ar), 70.1 (OCH_2_); HRMS (ESI) *m*/*z* calcd for C_17_H_13_^79^BrN_2_O_2_ (M­(^79^Br) + Na)^+^ 379.0053, found 379.0053 (−0.4 ppm error). Lab book reference WTWB-35.

### Benzyl 2-(3-(1*H*-Imidazol-2-yl)­phenyl)­cyclopent-1-ene-1-carboxylate **16f**

Using general procedure A in a sealed pressure tube, bis­(pinacolato)­diboron (305 mg, 1.20 mmol, 1.2 equiv), benzyl 2-(3-bromophenyl)-1*H*-imidazole-1-carboxylate **S3** (429 mg, 1.20 mmol, 1.2 equiv), dppf (28 mg, 0.0500 mmol, 0.05 equiv), Pd­(OAc)_2_ (11 mg, 0.0500 mmol, 0.05 equiv) and KOAc (236 mg, 2.40 mmol, 2.4 equiv) in dry, degassed 1,4-dioxane (4 mL) and then K_3_PO_4_ (1.06 g, 5.00 mmol, 5.0 equiv) and enol triflate **15** (350 mg, 1.00 mmol, 1.0 equiv) in dry, degassed 1,4-dioxane (1 mL) and degassed H_2_O (1 mL) gave the crude product. Purification by flash column chromatography on silica with 80:20 EtOAc:hexane as eluent gave benzyl ester **16f** (234 mg, 68%) as a colorless oil, *R*_F_ (8:2 EtOAc:hexane) 0.28; IR (ATR) 1697 (C = O), 1264, 1225, 1107 cm^–1^; ^1^H NMR (400 MHz, CDCl_3_) δ 7.77 (s, 1H, Ar), 7.73 (dd, *J* = 5.0, 5.0 Hz, 1H, Ar), 7.23–7.18 (m, 5H, Ar), 7.14–7.11 (m, 2H, Ph), 7.06 (s, 2H, Ar), 5.04 (s, 2H, OCH_2_), 2.82 (t, *J* = 8.0 Hz, 2H, = C*CH*_2_), 2.72 (t, *J* = 8.0 Hz, 2H, = C*CH*_2_), 1.91 (tt, *J* = 8.0, 8.0 Hz, 2H, CH_2_ *CH*_2_CH_2_); ^13^C NMR (100.6 MHz, CDCl_3_) δ 166.0 (C = O), 154.3 (=C), 146.7 (Ar), 137.7 (Ar), 135.9 (Ar), 129.9 (Ar), 129.3 (Ar), 128.5 (Ar), 128.5 (Ar), 128.1 (Ar), 128.0 (Ar), 125.5 (=C), 124.9 (Ar), 124.7 (Ar), 123.2 (Ar), 123.2 (Ar), 66.0 (OCH_2_), 40.5 (=C*CH*_2_), 35.2 (=C*CH*_2_), 22.0 (CH_2_ *CH*_2_CH_2_); HRMS (ESI) *m*/*z* calcd for C_22_H_20_N_2_O_2_ (M + H)^+^ 345.1598, found 345.1598 (−0.1 ppm error). Lab book reference WTWB-1-39.

### Benzyl 2-(Benzo­[*d*]­thiazol-6-yl)­cyclopent-1-ene-1-carboxylate **16g**

Using general procedure A in a sealed pressure tube, bis­(pinacolato)­diboron (305 mg, 1.20 mmol, 1.2 equiv), 6-bromobenzo­[*d*]­thiazole (257 mg, 1.20 mmol, 1.2 equiv), dppf (28 mg, 0.050 mmol, 0.05 equiv), Pd­(OAc)_2_ (11 mg, 0.050 mmol, 0.05 equiv) and KOAc (236 mg, 2.40 mmol, 2.4 equiv) in dry, degassed 1,4-dioxane (4 mL) and then K_3_PO_4_ (1.06 g, 5.00 mmol, 5.0 equiv) and enol triflate **15** (350 mg, 1.00 mmol, 1.0 equiv) in dry, degassed 1,4-dioxane (1 mL) and degassed H_2_O (1 mL) gave the crude product. Purification by flash column chromatography on silica with 60:40 hexane-Et_2_O as eluent gave benzyl ester **16g** (284 mg, 85%) as a yellow oil, *R*_F_ (6:4 hexane-Et_2_O) 0.16; IR (ATR) 2952, 2852, 1697 (C = O), 1468, 1438, 1342, 1262, 1221, 1116, 1039, 838, 737, 696 cm^–1^; ^1^H NMR (400 MHz, CDCl_3_) δ 8.96 (s, 1H, Ar), 8.03 (d, *J* = 8.5 Hz, 1H, Ar), 7.89 (d, *J* = 1.5 Hz, 1H, Ar), 7.43 (dd, *J* = 8.5, 1.5 Hz, 1H, Ar), 7.25–7.14 (m, 3H, Ar), 7.10–7.03 (m, 2H, Ar), 5.07 (s, 2H, CH_2_O), 2.97–2.85 (m, 4H, = CCH_2_), 2.02 (tt, *J* = 7.5, 7.5 Hz, 2H, CH_2_C*H*_2_CH_2_); ^13^C NMR (100.6 MHz, CDCl_3_) δ 165.7 (C = O), 154.3 (Ar), 153.2 (=*C*Ar), 152.8 (C), 135.7 (C), 134.8 (C), 133.5 (C), 130.0 (=*C*CO_2_Bn), 128.3 (Ar), 127.98 (Ar), 127.95 (Ar), 126.3 (Ar), 122.8 (Ar), 121.1 (Ar), 66.0 (CH_2_O), 40.8 (=C*C*H_2_), 35.2 (=C*C*H_2_), 22.0 (CH_2_ *C*H_2_CH_2_); HRMS (ESI) *m*/*z* calcd for C_20_H_17_NO_2_S (M + Na)^+^ 358.0872, found 358.0873 (−0.3 ppm error). Lab book reference JRD_XI_54.

### Benzyl 2-(Benzo­[*d*]­thiazol-5-yl)­cyclopent-1-ene-1-carboxylate **16h**

Using general procedure A in a sealed pressure tube, bis­(pinacolato)­diboron (305 mg, 1.20 mmol, 1.2 equiv), 5-bromobenzo­[*d*]­thiazole (257 mg, 1.20 mmol, 1.2 equiv), dppf (28 mg, 0.050 mmol, 0.05 equiv), Pd­(OAc)_2_ (11 mg, 0.050 mmol, 0.05 equiv) and KOAc (236 mg, 2.40 mmol, 2.4 equiv) in dry, degassed 1,4-dioxane (4 mL) and then K_3_PO_4_ (1.06 g, 5.00 mmol, 5.0 equiv) and enol triflate **15** (350 mg, 1.00 mmol, 1.0 equiv) in dry, degassed 1,4-dioxane (1 mL) and degassed H_2_O (1 mL) gave the crude product. Purification by flash column chromatography on silica with 60:40 hexane-Et_2_O as eluent gave benzyl ester **16h** (296 mg, 88%) as a yellow oil, *R*_F_ (6:4 hexane-Et_2_O) 0.17; IR (ATR) 2952, 2852, 1698 (C = O), 1439, 1344, 1254, 1223, 1188, 1114, 1039, 883, 810, 696 cm^–1^; ^1^H NMR (400 MHz, CDCl_3_) δ 9.02 (s, 1H, Ar), 8.13 (d, *J* = 1.5 Hz, 1H, Ar), 7.85 (d, *J* = 8.0 Hz, 1H, Ar), 7.43 (dd, *J* = 8.0, 1.5 Hz, 1H, Ar), 7.30–7.19 (m, 3H, Ar), 7.16–7.09 (m, 2H, Ar), 5.13 (s, 2H, CH_2_O), 3.03–2.92 (m, 4H, = CCH_2_), 2.08 (tt, *J* = 7.5, 7.5 Hz, CH_2_C*H*_2_CH_2_); ^13^C NMR (100.6 MHz, CDCl_3_) δ 165.6 (C = O), 154.2 (Ar), 153.4 (=CAr), 153.0 (Ar), 135.7 (Ar), 135.5 (Ar), 133.1 (Ar), 129.7 (=*C*CO_2_Bn), 128.2 (Ar), 127.9 (Ar), 127.8 (Ar), 125.7 (Ar), 122.4 (Ar), 121.0 (Ar), 65.8 (CH_2_O), 40.7 (=C*C*H_2_), 35.1 (=C*C*H_2_), 21.9 (CH_2_ *C*H_2_CH_2_); HRMS (ESI) *m*/*z* calcd for C_20_H_17_NO_2_S (M + Na)^+^ 358.0872, found 358.0874 (−0.5 ppm error). Lab book reference JRD_XI_53.

### Benzyl 2-(Benzo­[*c*]­[1,2,5]­thiadiazol-5-yl)­cyclopent-1-ene-1-carboxylate **16i**

Using general procedure A in a sealed pressure tube, bis­(pinacolato)­diboron (305 mg, 1.20 mmol, 1.2 equiv), 5-bromobenzo­[*c*]­[1,2,5]­thiadiazole (258 mg, 1.20 mmol, 1.2 equiv), dppf (28 mg, 0.050 mmol, 0.05 equiv), Pd­(OAc)_2_ (11 mg, 0.050 mmol, 0.05 equiv) and KOAc (236 mg, 2.40 mmol, 2.4 equiv) in dry, degassed 1,4-dioxane (4 mL) and then K_3_PO_4_ (1.06 g, 5.00 mmol, 5.0 equiv) and enol triflate **15** (350 mg, 1.00 mmol, 1.0 equiv) in dry, degassed 1,4-dioxane (1 mL) and degassed H_2_O (1 mL) gave the crude product. Purification by flash column chromatography on silica with 90:10 hexane-Et_2_O as eluent gave benzyl ester **16i** (280 mg, 83%) as a green oil that solidified on standing to give a gray solid, mp 58–60 °C; *R*_F_ (9:1 hexane-Et_2_O) 0.15; IR (ATR) 2953, 1702 (C = O), 1249, 1198, 1128, 1114, 1028, 817, 752, 697 cm^–1^; ^1^H NMR (400 MHz, CDCl_3_) δ 7.87–7.84 (m, 1H, Ar), 7.82 (d, *J* = 9.0 Hz, 1H, Ar), 7.47 (dd, *J* = 9.0, 1.5 Hz, 1H, Ar), 7.24–7.13 (m, 3H, Ar), 7.11–7.06 (m, 2H, Ar), 5.06 (s, 2H, CH_2_O), 2.98–2.89 (m, 4H, =CCH_2_), 2.07 (tt, *J* = 7.5, 7.5 Hz, 2H, CH_2_C*H*_2_CH_2_); ^13^C NMR (100.6 MHz, CDCl_3_) δ 165.4 (C = O), 154.7 (C), 154.3 (C), 152.6 (=*C*Ar), 139.0 (C), 135.6 (C), 131.4 (=*C*CO_2_Bn), 130.7 (Ar), 128.3 (Ar), 128.14 (Ar), 128.11 (Ar), 120.4 (Ar), 119.5 (Ar), 66.2 (*C*H_2_O), 40.6 (=C*C*H_2_), 35.1 (=C*C*H_2_), 22.1 (CH_2_ *C*H_2_CH_2_); HRMS (ESI) *m*/*z* calcd for C_19_H_16_N_2_O_2_S (M + Na)^+^ 359.0825, found 359.0832 (−2.1 ppm error). Lab book reference JRD_XI_52.

### 2-(Isoquinolin-6-yl)­cyclopentane-1-carboxylic Acid *cis*-**17a**

Using general procedure B, alkene **16a** (153 mg, 0.464 mmol, 1.0 equiv) and 10% Pd/C (15 mg) in MeOH (6 mL) at 40 °C for 72 h gave the crude product. Purification by flash column chromatography on silica with 95:5 to 0:100 CH_2_Cl_2_-MeOH as eluent gave isoquinolinyl cyclopentane carboxylic acid *cis*-**17a** (17 mg, 15%, ≥ 95% pure by ^1^H NMR) as a colorless solid, *R*_F_ (19:1 CH_2_Cl_2_-MeOH) 0.37, IR (ATR) 2953, 2871, 1713 (C = O), 1632, 1392, 1205, 1038, 889, 832, 669, 476 cm^–1^; ^1^H NMR (400 MHz, MeOD-*d*_4_) 9.13 (s, 1H, Ar), 8.35 (d, *J* = 5.5 Hz, 1H, Ar), 7.98 (d, *J* = 8.5 Hz, 1H, Ar), 7.78 (s, 1H, Ar), 7.75 (d, *J* = 5.5 Hz, 1H, Ar), 7.63 (m, 1H, Ar), 4.00 (s, 2H, CH_2_O), 3.71–3.62 (m, 1H, C*H*Ar), 3.34–3.26 (m, 1H, C*H*CO_2_H), 2.34–2.02 (m, 5H, CH), 1.90–1.76 (m, 1H, CH); ^13^C NMR (100.6 MHz, MeOD-*d*_4_) δ 177.6 (C = O), 151.9 (Ar), 146.8 (Ar), 141.9 (Ar), 136.9 (Ar), 129.8 (Ar), 128.4 (Ar), 127.8 (Ar), 125.2 (Ar), 121.7 (Ar), 50.4 (C*H*CO_2_H), 49.7 (C*H*Ar), 31.5 (=C*C*H_2_), 29.3 (=C*C*H_2_), 24.8 (CH_2_ *CH*_2_CH_2_); HRMS (ESI) *m*/*z* calcd for C_15_H_15_NO_2_ (M + H)^+^ 242.1176, found 242.1179 (−1.4 ppm error). Lab book reference JRD_XI_46.

### 2-(1-Methyl-1*H*-benzo­[*d*]­imidazol-6-yl)­cyclopentane-1-carboxylic Acid *cis*-**17b**

Using general procedure B, alkene **16b** (200 mg, 0.602 mmol, 1.0 equiv) and 10% Pd/C (20 mg) in MeOH (6 mL) at 40 °C for 24 h gave cyclopentane carboxylic acid *cis*-**17b** (130 mg, 88%, ≥ 95% pure by ^1^H NMR) as a pale brown solid, IR (ATR) 2922, 2855, 1717 (C = O), 1551, 1440, 1171, 1099, 824, 632, 603 cm^–1^; ^1^H NMR (400 MHz, MeOD-*d*_4_) δ 8.80 (s, 1H, Ar), 7.69–7.57 (m, 2H, Ar), 7.44 (d, *J* = 8.0 Hz, 1H, Ar), 4.00 (s, 3H, Me), 3.72–3.61 (m, 1H, C*H*Ar), 3.29–3.21 (m, 1H, C*H*CO_2_H), 2.81–2.01 (m, 5H, CH), 1.90–1.72 (m, 1H, CH); ^13^C NMR (100.6 MHz, MeOD-*d*_4_) δ 177.7 (C = O), 142.7 (Ar), 140.8 (Ar), 134.7 (Ar), 133.6 (Ar), 126.5 (Ar), 115.9 (Ar), 111.0 (Ar), 50.7 (*C*HCO_2_H), 49.6 (*C*HAr), 32.0 (=C*C*H_2_), 31.9 (Me), 29.2 (=C*C*H_2_), 24.8 (CH_2_ *C*H_2_CH_2_); HRMS (ESI) *m*/*z* calcd for C_14_H_16_N_2_O_2_ (M + H)^+^ 245.1285, found 245.1287 (−1.1 ppm error). Lab book reference JRD_XI_48.

### 2-(1*H*-Indazol-4-yl)­cyclopentane-1-carboxylic Acid *cis*-**17c**

Using general procedure B, alkene **16c** (230 mg, 0.128 mmol, 1.0 equiv) and 10% Pd/C (23 mg) in MeOH (6 mL) at rt for 32 h gave cyclopentane carboxylic acid *cis*-**17c** (153 mg, 98%, ≥ 95% pure by HPLC) as a pale-yellow gum, IR (ATR) 3278, 2956, 2873, 1694 (C = O), 1614, 1361, 1296, 1219, 1089, 952, 857, 790, 744, 682 cm^–1^; ^1^H NMR (400 MHz, MeOD-*d*_4_) δ 8.14 (s, 1H, Ar), 7.30 (d, *J* = 7.0 Hz, 1H, Ar), 7.22 (dd, *J* = 7.0, 7.0 Hz, 1H, Ar), 6.96 (d, *J* = 7.0 Hz, 1H, Ar), 3.92–3.77 (m, 1H, C*H*Ar), 3.28–3.24 (m, 1H, C*H*CO_2_H), 2.35–2.18 (m, 1H, CH), 2.18–1.91 (m, 4H, CH), 1.82–1.64 (m, 1H, CH); ^13^C NMR (100.6 MHz, MeOD-*d*_4_) δ 177.9 (C = O), 140.9 (Ar), 135.8 (Ar), 133.4 (Ar), 127.2 (Ar), 123.8 (Ar), 118.8 (Ar), 108.6 (Ar), 49.9 (C*H*CO_2_H), 46.8 (*C*HAr), 31.1 (CH*C*H_2_), 29.1 (CH*C*H_2_), 24.5 (CH_2_ *CH*_2_CH_2_); HRMS (ESI) *m*/*z* calcd for C_13_H_12_N_2_O_2_ (M + H)^+^ 229.0983, found 229.0978 (+1.8 ppm error). Lab book reference JRD_XI_51.

### 2-(Benzo­[*d*]­oxazol-5-yl)­cyclopentane-1-carboxylic Acid *cis*-**17d**

Using general procedure B, alkene **16d** (41 mg, 0.128 mmol, 1.0 equiv) and 10% Pd/C (15 mg) in MeOH (6 mL) at rt for 16 h gave cyclopentane carboxylic acid *cis*-**17d** (29 mg, 98%, ≥ 95% pure by ^1^H NMR) as a brown gum, IR (ATR) 2980, 1679 (C = O), 1384, 1341, 1201, 1135 cm^–1^; ^1^H NMR (400 MHz, MeOD-*d*_4_) δ 8.41 (s, 1H, Ar), 7.64 (s, 1H, Ar), 7.54 (d, *J* = 8.5 Hz, Ar), 7.36 (d, *J* = 8.5 Hz, 1H, Ar), 3.64–3.55 (m, 1H, C*H*Ar), 3.24–3.15 (m, 1H, C*H*CO_2_H), 2.24–1.96 (m, 5H, CH), 1.87–1.81 (m, 1H, CH); ^13^C NMR (100.6 MHz, MeOD-*d*_4_) δ 181.1 (C = O), 157.9 (Ar), 152.6 (C), 143.24 (C), 143.19 (C), 130.3 (Ar), 122.6 (Ar), 113.8 (Ar), 54.0 (*C*HCO_2_H), 52.7 (*C*HAr), 35.3 (CH*C*H_2_), 32.3 (CH*C*H_2_), 28.0 (CH_2_ *CH*_2_CH_2_); HRMS (ESI) *m*/*z* calcd for C_13_H_11_NO_3_ (M + H)^+^ 230.0823, found 230.0817 (+2.3 ppm error). Lab book reference JRD_XI_49.

### 2-(5-Fluoro-2,3-dihydrobenzofuran-7-yl)­cyclopentane-1-carboxylic Acid *cis*-**17e**

Using general procedure B, alkene **16e** (250 mg, 0.743 mmol, 1.0 equiv) and 10% Pd/C (25 mg, 0.007 mmol, 0.01 equiv) in MeOH (10 mL) at rt for 24 h gave acid *cis-* **17e** (186 mg, 99%, ≥ 95% pure by ^1^H NMR) as an off-white solid, mp 124–126 °C; IR (ATR) 2921, 2696 (br, OH), 1692 (C = O), 1469, 1432, 1226, 1181 cm^–1^; ^1^H NMR (400 MHz, CDCl_3_) δ 8.12 (br s, 1H, OH), 6.72 (dd, *J* = 8.0, 2.5 Hz, 1H, Ar), 6.68 (dd, *J* = 10.5, 2.5 Hz, 1H, Ar), 4.54–4.41 (m, 2H, O*CH*_2_), 3.36 (ddd, *J* = 8.5, 8.5, 8.5 Hz, 1H, CHAr), 3.15–3.04 (m, 3H, CH), 2.07–1.84 (m, 5H, CH), 1.71–1.58 (m, 1H, CH); ^13^C NMR (100.6 MHz, CDCl_3_) δ 180.3 (C = O), 157.2 (d, *J* = 237.5 Hz, *ipso*-Ar), 154.3 (*ipso*-Ar), 127.2 (d, *J* = 9.0 Hz, *ipso*-Ar), 124.8 (d, *J* = 8.0 Hz, *ipso*-Ar), 112.7 (d, *J* = 24.5 Hz, Ar), 109.7 (d, *J* = 25.0 Hz, Ar), 71.2 (OCH_2_), 48.7 (*C*HCO_2_H), 42.9 (CHAr), 30.3 (CH_2_Ar), 30.1 (CH*C*H_2_), 29.1 (CH*C*H_2_), 24.3 (CH_2_ *C*H_2_CH_2_); ^19^F NMR (376.5 MHz, CDCl_3_) δ−124.5 (dd, *J* = 10.5, 8.0 Hz, F); HRMS (ESI) *m*/*z* calcd for C_14_H_15_FO_3_ (M - H + 2Na)^+^ 295.0717, found 295.0717 (+0.1 ppm error). The *cis* configuration was confirmed by X-ray crystallography: CCDC 2482170. Lab book reference WTWB-1-21.

### 2-(3-(1*H*-Imidazol-2-yl)­phenyl)­cyclopentane-1-carboxylic Acid *cis*-**17f**

Using general procedure B, alkene **16f** (197 mg, 0.572 mmol, 1.0 equiv) and 10% Pd/C (20 mg, 0.006 mmol, 0.01 equiv) in MeOH (10 mL) at 40 °C for 24 h gave acid *cis-* **17f** (130 mg, 88%, ≥ 95% pure by HPLC) as a brown oil, IR (ATR) 3065 (br, OH), 2952, 1557 (C = O), 1405, 1107 cm^–1^; ^1^H NMR (400 MHz, CD_3_OD) δ 7.72 (d, *J* = 4.0 Hz, 1H, Ar), 7.67–7.63 (m, 1H, Ar), 7.31–7.28 (m, 2H, Ar), 7.12–7.09 (m, 2H, Ar), 3.49–3.40 (m, 1H, CHAr), 3.17–3.09 (m, 1H, *CH*CO_2_H), 2.24–1.93 (m, 5H, CH), 1.80–1.69 (m, 1H, CH); ^13^C NMR (100.6 MHz, CD_3_OD) δ 180.7 (C = O), 147.8 (Ar), 144.7 (Ar), 130.1 (Ar), 129.4 (Ar), 128.8 (Ar), 125.7 (Ar), 123.8 (Ar), 123.0 (Ar), 123.0 (Ar), 52.7 (CHAr), 49.5 (*C*HCO_2_H), 32.1 (CH*CH*_2_), 29.5 (CH*CH*_2_), 24.8 (CH_2_ *CH*_2_CH_2_); HRMS (ESI) *m*/*z* calcd for C_15_H_16_N_2_O_2_ (M–H)^−^ 255.1139, found 255.1139 (−1.2 ppm error). Lab book reference WTWB-1-42.

### Methyl 2-(Trifluoromethanesulfonyloxy)­cyclopent-1-ene-1-carboxylate **18**

*N*-Ethyl-*N*,*N*-diisopropylamine (12.9 mL, 73.9 mmol, 5.0 equiv) was added dropwise to a stirred solution of methyl cyclopentanone-2-carboxylate (2.01 g, 14.8 mmol, 1.0 equiv) in CH_2_Cl_2_ (100 mL) at −78 °C under N_2_. The resulting solution was stirred at −78 °C for 10 min. Then, trifluoromethanesulfonic anhydride (2.98 mL, 17.7 mmol, 1.2 equiv) was added dropwise over 15 min. The solution was allowed to warm to rt and then stirred at rt for 16 h. H_2_O (50 mL) and 5% citric acid_(aq)_ (200 mL) were added, and the aqueous layer was extracted with CH_2_Cl_2_ (3 × 50 mL). The combined organic extracts were dried (MgSO_4_), and evaporated under reduced pressure to give the crude product. Purification by flash column chromatography on silica with 91:9 *n*-hexane-EtOAc as eluent gave impure product. Further purification by flash column chromatography on silica with 94:6 *n*-hexane-EtOAc as eluent gave **18** (2.72 g, 70%) as a yellow solid, *R*_F_ (10:1 *n-*hexane- EtOAc) 0.65; IR (ATR) 1724 (C = O), 1668 (C = C), 1424, 1202, 1127 cm^–1^; ^1^H NMR (400 MHz, CDCl_3_) δ 3.80 (s, 3H, OMe), 2.78–2.68 (m, 4H, = CCH_2_), 2.02 (tt, *J* = 7.5, 7.5 Hz, 2H, CH_2_C*H*_2_CH_2_); ^13^C NMR (100.6 MHz, CDCl_3_) δ 162.8 (C = O), 154.1 (=C–O), 123.1 (=*C*CO_2_Me), 118.5 (q, *J*_*C–F*_ = 318.4 Hz, CF_3_), 52.0 (OMe), 32.9 (=C*CH*_2_), 29.3 (=C*CH*_2_), 19.0 (CH_2_ *C*H_2_CH_2_); ^19^F NMR (376.5 MHz, CDCl_3_) δ−74.41 (s, Ar–F); HRMS (ESI) *m*/*z* C_8_H_9_F_3_O_5_S (M + Na)^+^ 297.0015, found 297.0011 (+1.4 ppm error). Lab book reference XW-001-065.

### Methyl 2-(Benzo­[*d*]­thiazol-6-yl)­cyclopent-1-ene-1-carboxylate **19a**

Using general procedure A in a round-bottomed flask, bis­(pinacolato)­diboron (305 mg, 1.2 mmol, 1.2 equiv), 6-bromobenzo­[*d*]­thiazole (257 mg, 1.2 mmol, 1.2 equiv), dppf (28 mg, 0.05 mmol, 0.05 equiv), Pd­(OAc)_2_ (11 mg, 0.05 mmol, 0.05 equiv) and KOAc (236 mg, 2.4 mmol, 2.4 equiv) in dry, degassed 1,4-dioxane (4.0 mL) and then K_3_PO_4_ (1.06 g, 5.0 mmol, 5.0 equiv) and enol triflate **18** (275 mg, 1.0 mmol, 1.0 equiv) in dry, degassed 1,4-dioxane (1.0 mL) and degassed H_2_O (1.0 mL) gave the crude product. Purification by flash column chromatography on silica with 75:25 *n-*hexane-EtOAc as eluent gave arylated ester **19a** (230 mg, 89%) as a yellow oli, *R*_F_ (3:1 *n-*hexane- EtOAc) 0.40; IR (ATR) 2948, 1705 (C = O), 1626 (C = C), 1469, 1435, 1228 cm^–1^; ^1^H NMR (400 MHz, CDCl_3_) δ 8.99 (br s, 1H, Ar), 8.08 (d, *J* = 8.5 Hz, 1H, Ar), 7.96 (d, *J* = 1.5 Hz, 1H, Ar), 7.48 (dd, *J* = 8.5, 1.5 Hz, 1H, Ar), 3.63 (s, 3H, OMe), 2.94–2.85 (m, 4H, = CCH_2_), 2.03 (tt, *J* = 7.5, 7.5 Hz, 2H, CH_2_C*H*_2_CH_2_); ^13^C NMR (100.6 MHz, CDCl_3_) δ 166.5 (C = O), 154.5 (Ar), 153.0 (*ipso*-Ar), 152.9 (=*C–*Ar), 134.7 (*ipso*-Ar), 133.6 (*ipso*-Ar), 129.9 (=*C*–CO), 126.6 (Ar), 122.9 (Ar), 121.2 (Ar), 51.4 (OMe), 40.6 (*=*C*C*H_2_), 35.3 (*=*C*C*H_2_), 22.1 (CH_2_ *C*H_2_CH_2_); HRMS (ESI) *m*/*z* calcd for C_14_H_13_NO_2_S (M + Na)^+^ 282.0559, found 282.0557 (+0.7 ppm error). Lab book reference XW-001-078.

### Methyl 2-(Quinoxalin-6-yl)­cyclopent-1-ene-1-carboxylate **19b**

Using general procedure A in a round-bottomed flask, bis­(pinacolato)­diboron (218 mg, 0.86 mmol, 1.2 equiv), 6-bromoquinoxaline (180 mg, 0.86 mmol, 1.2 equiv), dppf (20 mg, 0.04 mmol, 0.05 equiv), Pd­(OAc)_2_ (8.1 mg, 0.04 mmol, 0.05 equiv), and KOAc (169 mg, 1.72 mmol, 2.4 equiv) in dry, degassed 1,4-dioxane (4.0 mL) and then K_3_PO_4_ (764 mg, 3.6 mmol, 5.0 equiv) and enol triflate **18** (197 mg, 0.72 mmol, 1.0 equiv) in dry, degassed 1,4-dioxane (1.0 mL) and degassed H_2_O (1.0 mL) gave the crude product. Purification by flash column chromatography on silica with 75:25 *n-*hexane-EtOAc as eluent gave arylated ester **19b** (108 mg, 59%) as a yellow oil, *R*_F_ (3:1 *n-*hexane- EtOAc) 0.20; IR (ATR) 2950, 1713 (C = O), 1634, 1611, 1495, 1435, 1369, 1355, 1340, 1257, 1233 cm^–1^; ^1^H NMR (400 MHz, CDCl_3_) δ 8.84 (m, 2H, Ar), 8.07 (d, *J* = 8.5 Hz, 1H, Ar), 8.03 (d, *J* = 1.5 Hz, 1H, Ar), 7.77 (dd, *J* = 8.5, 1.5 Hz, 1H, Ar), 3.64 (s, 3H, OMe), 3.02–2.89 (m, 4H, = CCH_2_), 2.08 (tt, *J* = 7.5, 7.5 Hz, 2H, CH_2_C*H*_2_CH_2_); ^13^C NMR (100.6 MHz, CDCl_3_) δ 166.3 (C = O), 152.3 (=*C–*Ar), 145.3 (Ar), 145.1 (Ar), 142.8 (*ipso*-Ar), 142.8 (*ipso*-Ar), 139.3 (Ar), 131.1 (*ipso*-Ar), 130.9 (=*C*–CO), 128.7 (Ar), 128.0 (Ar), 51.5 (OMe), 40.4 (*=*C*C*H_2_), 35.4 (*=*C*C*H_2_), 22.2 (CH_2_ *C*H_2_CH_2_); HRMS (ESI) *m*/*z* calcd for C_15_H_14_N_2_O_2_ (M + Na)^+^ 277.0947, found 277.0948 (−0.1 ppm error). Lab book reference XW-001-077.

### Methyl 2-(Isoquinolin-6-yl)­cyclopent-1-ene-1-carboxylate **19c**

Using general procedure A in a round-bottomed flask, bis­(pinacolato)­diboron (305 mg, 1.2 mmol, 1.2 equiv), 6-bromoisoquinoline (250 mg, 1.2 mmol, 1.2 equiv), dppf (28 mg, 0.05 mmol, 0.05 equiv), Pd­(OAc)_2_ (11 mg, 0.05 mmol, 0.05 equiv), and KOAc (236 mg, 2.4 mmol, 2.4 equiv) in dry, degassed 1,4-dioxane (4.0 mL) and then K_3_PO_4_ (1.06 g, 5.0 mmol, 5.0 equiv) and enol triflate **18** (275 mg, 1.0 mmol, 1.0 equiv) in dry, degassed 1,4-dioxane (1.0 mL) and degassed H_2_O (1.0 mL) gave the crude product. Purification by flash column chromatography on silica with 83:17–75:25–50:50 *n-*hexane-EtOAc as eluent gave arylated ester **19c** (91 mg, 36%) as a brown solid, mp 58–62 °C; *R*_F_ (3:1 *n-*hexane- EtOAc) 0.20; IR (ATR) 2949, 1711 (C = O), 1626 (C = C), 1434, 1254, 1234, 1197 cm^–1^; ^1^H NMR (400 MHz, CDCl_3_) δ 9.23 (br s, 1H, Ar), 8.51 (d, *J* = 5.5 Hz, 1H, Ar), 7.93 (d, *J* = 8.5 Hz, 1H, Ar), 7.74 (s, 1H, Ar), 7.55 (d, *J* = 5.5 Hz, 1H, Ar), 7.55 (dd, *J* = 8.5, 1.5 Hz, 1H, Ar), 3.62 (s, 3H, OMe), 2.96 (tt, *J* = 7.5, 2.5 Hz, 2H, = CCH_2_), 2.89 (tt, *J* = 7.5, 2.5 Hz, 2H, = CCH_2_), 2.07 (tt, *J* = 8.0, 8.0 Hz, 2H, CH_2_C*H*_2_CH_2_); ^13^C NMR (100.6 MHz, CDCl_3_) δ 166.3 (C = O), 153.0 (=*C*–Ar), 152.3 (Ar), 143.4 (Ar), 139.5 (*ipso*-Ar), 135.6 (*ipso*-Ar), 130.9 (*ipso*-Ar), 128.1 (=*C*–CO), 127.8 (Ar), 127.0 (Ar), 125.1 (Ar), 120.8 (Ar), 51.5 (OMe), 40.5 (*=*C*C*H_2_), 35.3 (*=*C*C*H_2_), 22.2 (CH_2_ *C*H_2_CH_2_); HRMS (ESI) *m*/*z* calcd for C_16_H_15_NO_2_ (M + H)^+^ 254.1176, found 254.1171 (+1.7 ppm error). Lab book reference XW-001-076.

### Methyl 2-(Quinolin-7-yl)­cyclopent-1-ene-1-carboxylate **19d**

Using general procedure A in a round-bottomed flask, bis­(pinacolato)­diboron (305 mg, 1.2 mmol, 1.2 equiv), 7-bromoquinoline (250 mg, 1.2 mmol, 1.2 equiv), dppf (28 mg, 0.05 mmol, 0.05 equiv), Pd­(OAc)_2_ (11.0 mg, 0.05 mmol, 0.05 equiv), and KOAc (236 mg, 2.4 mmol, 2.4 equiv) in dry, degassed 1,4-dioxane (4.0 mL) and then K_3_PO_4_ (1.06 g, 5.0 mmol, 5.0 equiv) and enol triflate **18** (275 mg, 1.0 mmol, 1.0 equiv) in dry, degassed 1,4-dioxane (1.0 mL) and degassed H_2_O (1.0 mL) gave the crude product. Purification by flash column chromatography on silica with 83:17 hexane-EtOAc as eluent gave **19d** (78 mg, 26%) as an orange solid, mp 59–66 °C; *R*_F_ (2:1 *n*-hexane: EtOAc) 0.45; IR (ATR) 3023, 2976, 2949, 2868, 2851, 1702 (C = O), 1239, 840, 764 cm^–1^; ^1^H NMR (400 MHz, CDCl_3_) δ 8.88 (dd, *J* = 4.0, 2.0 Hz, 1H, Ar), 8.10 (dd, *J* = 8.0, 2.0 Hz, 1H, Ar), 8.02 (br s, 1H, Ar), 7.73 (d, *J* = 8.5 Hz, 1H, Ar), 7.53 (dd, *J* = 8.0, 1.5 Hz, 1H, Ar), 7.37 (dd, *J* = 8.0, 4.0 Hz, 1H, Ar), 3.61 (s, 3H, OMe), 3.02–2.83 (m, 4H, = CCH_2_), 2.10–1.98 (m, 2H, CH_2_C*H*_2_CH_2_); ^13^C NMR (100.6 MHz, CDCl_3_) δ 166.5 (C = O), 152.9 (=*C*–Ar), 150.7 (Ar), 148.0 (*ipso*-Ar), 138.5 (*ipso*-Ar), 135.9 (Ar), 130.3 (*ipso*-Ar), 128.0 (Ar), 127.9 (=*C*–CO), 127.3 (Ar), 127.0 (Ar), 121.3 (Ar), 51.4 (OMe), 40.3 (CH_2_), 35.4 (CH_2_), 22.1 (CH_2_); HRMS (ESI) *m*/*z* calcd for C_16_H_15_NO_2_ (M + H)^+^ 254.1176 found 254.1174 (+0.6 ppm error). Lab book reference AS-1-14.

### Methyl 2-(1-Methyl-1*H*-benzo­[*d*]­imidazol-6-yl)­cyclopent-1-ene-1-carboxylate **19e**

Using general procedure A in a round-bottomed flask, bis­(pinacolato)­diboron (305 mg, 1.2 mmol, 1.2 equiv), 6-bromo-1-methyl-1*H*-benzo­[*d*]­imidazole (253.3 mg, 1.2 mmol, 1.2 equiv), dppf (28 mg, 0.05 mmol, 5 mol %), Pd­(OAc)­2 (11 mg, 0.05 mmol, 5 mol %), and KOAc (236 mg, 2.40 mmol, 2.4 equiv) in dry, degassed 1,4-dioxane (4.0 mL) and then K_3_PO_4_ (1.06 g, 5.0 mmol, 5.0 equiv) and enol triflate **18** (275 mg, 1.0 mmol, 1.0 equiv) in dry, degassed 1,4-dioxane (1.0 mL) and degassed H_2_O (1.0 mL) gave the crude product. Purification by flash column chromatography on silica with 50:50–0:100 *n*-hexane-EtOAc as eluent gave arylated ester **19e** (181 g, 71%) as a brown solid, mp 55–57 °C; *R*_F_ (1:1 *n*-hexane-EtOAc) 0.05; IR (ATR) 2948, 1704 (C = O), 1620, 1500, 1462, 1434, 1349, 1290, 1251, 1222, 1121, 1045 cm^–1^; ^1^H NMR (400 MHz, CDCl_3_) δ 7.87 (br s, 1H, Ar), 7.73 (d, *J* = 8.5 Hz, 1H, Ar), 7.43 (d, *J* = 1.5 Hz, 1H, Ar), 7.24 (dd, *J* = 8.5, 1.5 Hz, 1H, Ar), 3.83 (s, 3H, NMe), 3.62 (s, 3H, OMe), 2.93 (tt, *J* = 7.5, 2.5 Hz, 2H, = CCH_2_), 2.86 (tt, *J* = 7.5, 2.5 Hz, 2H, = CCH_2_), 2.02 (tt, *J* = 7.5, 7.5 Hz, 2H, CH_2_C*H*_2_CH_2_); ^13^C NMR (100.6 MHz, CDCl_3_) δ 167.0 (C = O), 154.1 (=*C*Ar), 144.3 (Ar), 143.6 (*ipso*-Ar), 134.3 (*ipso*-Ar), 132.0 (*ipso*-Ar), 128.7 (=*C*CO), 122.6 (Ar), 119.6 (Ar), 109.0 (Ar), 51.3 (OMe), 40.8 (CH_2_), 35.4 (CH_2_), 31.2 (NMe), 22.1 (CH_2_); HRMS (ESI) *m*/*z* calcd for C_15_H_17_N_2_O_2_ (M + H)^+^ 257.1285, found 257.1293 (−3.4 ppm error). Lab book reference XW-001-158.

### Methyl 2-(Quinolin-6-yl)­cyclopent-1-ene-1-carboxylate **19f**

Using general procedure A in a round-bottomed flask, bis­(pinacolato)­diboron (305 mg, 1.2 mmol, 1.2 equiv), 6-bromoquinoline (0.16 mL, 1.2 mmol, 1.2 equiv), dppf (28 mg, 0.05 mmol, 0.05 equiv), Pd­(OAc)_2_ (11.0 mg, 0.05 mmol, 0.05 equiv), and KOAc (236 mg, 2.4 mmol, 2.4 equiv) in dry, degassed 1,4-dioxane (4.0 mL) and then K_3_PO_4_ (1.06 g, 5.0 mmol, 5.0 equiv) and enol triflate **18** (275 mg, 1.0 mmol, 1.0 equiv) in dry, degassed 1,4-dioxane (1.0 mL) and degassed H_2_O (1.0 mL) gave the crude product. Purification by flash column chromatography on silica with 83:17 *n*-hexane-EtOAc as eluent gave **19f** (191 mg, 64%) as an orange low melting point solid, *R*_F_ (2:1 *n*-hexane: EtOAc) 0.4; IR (ATR) 2950, 2855, 1713 (C = O), 1241, 1186, 837, 478 cm^–1^; ^1^H NMR (400 MHz, CDCl_3_) δ 8.89 (dd, *J* = 4.5, 2.0 Hz, 1H, Ar), 8.13 (dd, *J* = 8.5, 2.0 Hz, 1H, Ar), 8.05 (d, *J* = 9.0 Hz, 1H, Ar), 7.77 (d, *J* = 2.0 Hz, 1H, Ar), 7.68 (dd, *J* = 9.0, 2.0 Hz, 1H, Ar), 7.39 (dd, *J* = 8.5, 4.5 Hz, 1H, Ar), 3.62 (s, 3H, OMe), 3.00–2.84 (m, 4H, = CCH_2_), 2.11–1.99 (m, 2H, CH_2_C*H*_2_CH_2_); ^13^C NMR (100.6 MHz, CDCl_3_) δ 166.5 (C = O), 152.9 (=*C*–Ar), 150.7 (Ar), 148.0 (*ipso*-Ar), 136.4 (Ar), 135.5 (*ipso*-Ar), 130.2 (*ipso*-Ar), 130.0 (Ar), 128.8 (Ar), 127.9 (=*C*–CO), 126.6 (Ar), 121.4 (Ar), 51.4 (OMe), 40.4 (CH_2_), 35.3 (CH_2_), 22.1 (CH_2_); HRMS (ESI) *m*/*z* calcd for C_16_H_15_NO_2_ (M + H)^+^ 254.1176 found 254.1177 (−0.7 ppm error). Lab book reference AS-1-12.

### 2-(Benzo­[*d*]­thiazol-6-yl)­cyclopent-1-ene-1-carboxylic Acid **20a**

Using general procedure C-1, NaOH (440 mg, 11.0 mmol, 5.0 equiv) and arylated ester **19a** (570 mg, 2.2 mmol, 1.0 equiv) in MeOH (3.0 mL) and H_2_O (3.0 mL) at 50 °C for 6 h, using H_2_O (50 mL), EtOAc (3 × 10 mL) and then EtOAc (3 × 20 mL) in the workup gave carboxylic acid **20a** (460 mg, 85%, ≥ 95% pure by HPLC) as an orange solid, mp 180–182 °C; IR (ATR) 2953 (br, O–H), 1679 (C = O), 1265 cm^–1^; ^1^H NMR (400 MHz, DMSO-*d*_6_) δ 9.38 (br s, 1H, Ar), 8.14 (d, *J* = 1.5 Hz, 1H, Ar), 8.02 (d, *J* = 8.5 Hz, 1H, Ar), 7.52 (dd, *J* = 8.5, 1.5 Hz, 1H, Ar), 2.90 (tt, *J* = 7.5, 2.5 Hz, 2H, = CCH_2_), 2.76 (tt, *J* = 7.5, 2.5 Hz, 2H, = CCH_2_), 1.94 (tt, *J* = 7.5, 7.5 Hz, 2H, CH_2_C*H*_2_CH_2_); ^13^C NMR (100.6 MHz, DMSO-*d*_6_) δ 167.1 (C = O), 156.6 (=*C–*Ar), 152.4 (Ar), 150.2 (*ipso*-Ar), 134.3 (*ipso*-Ar), 133.3 (*ipso*-Ar), 130.4 (=*C*–CO), 126.4 (Ar), 122.1 (Ar), 121.5 (Ar), 35.3 (*=*C*C*H_2_), 21.4 (CH_2_ *C*H_2_CH_2_) (one *=*C*C*H_2_ resonance not resolved); HRMS (ESI) *m*/*z* calcd for C_13_H_11_NO_2_S (M + Na)^+^ 268.0403, found 268.0401 (+0.6 ppm error). Lab book reference XW-001-102.

### 2-(Quinoxalin-6-yl)­cyclopent-1-ene-1-carboxylic Acid **20b**

Using general procedure C-1, NaOH (78 mg, 1.95 mmol, 5.0 equiv) and arylated ester **19b** (100 mg, 0.39 mmol, 1.0 equiv) in MeOH (1.0 mL) and H_2_O (1.0 mL) at 50 °C for 6 h, using H_2_O (20 mL), EtOAc (3 × 10 mL) and then EtOAc (3 × 20 mL) in the workup gave carboxylic acid **20b** (90 mg, 95%, ≥ 95% pure by HPLC) as an off-white solid, mp 212–216 °C; IR (ATR) 2920 (br, O–H), 1685 (C = O), 1499, 1432, 1231 cm^–1^; ^1^H NMR (400 MHz, DMSO-*d*_6_) δ 8.94–8.92 (m, 2H, Ar), 8.04–8.02 (m, 2H, Ar), 7.84 (d, *J* = 8.5 Hz, 1H, Ar), 7.52 (dd, *J* = 8.5, 1.5 Hz, 1H, Ar), 2.97 (tt, *J* = 7.5, 2.5 Hz, 2H, = CCH_2_), 2.80 (tt, *J* = 7.5, 2.5 Hz, 2H, = CCH_2_), 1.98 (tt, *J* = 7.5, 7.5 Hz, 2H, CH_2_C*H*_2_CH_2_); ^13^C NMR (100.6 MHz, DMSO-*d*_6_) δ 146.0 (=*C–*Ar), 145.8 (Ar), 142.1 (*ipso*-Ar), 141.9 (Ar), 141.7 (*ipso*-Ar), 138.9 (Ar), 132.0 (*ipso*-Ar), 130.7 (Ar), 128.2 (=*C*–CO), 127.4 (Ar), 35.4 (*=*C*C*H_2_), 21.5 (CH_2_ *C*H_2_CH_2_) (one *=*C*C*H_2_ and C = O resonances not resolved); HRMS (ESI) *m*/*z* calcd for C_14_H_12_N_2_O_2_ (M + Na)^+^ 263.0791, found 263.0793 (−0.9 ppm error). Lab book reference XW-001-097.

### 2-(Isoquinolin-6-yl)­cyclopent-1-ene-1-carboxylic Acid **20c**

Using general procedure C-1, NaOH (64 mg, 1.6 mmol, 5.0 equiv) and arylated ester **19c** (80 mg, 0.32 mmol, 1.0 equiv) in MeOH (1.0 mL) and H_2_O (1.0 mL) at 50 °C for 6 h, using H_2_O (20 mL), EtOAc (3 × 10 mL) and then EtOAc (5 × 20 mL) in the workup gave carboxylic acid **20c** (55 mg, 73%, ≥ 95% pure by HPLC) as a yellow solid, mp 208–212 °C; IR (ATR) 2950 (br, O–H), 1668 (C = O), 1631 cm^–1^; ^1^H NMR (400 MHz, DMSO-*d*_6_) δ 9.28 (br s, 1H, Ar), 8.48 (d, *J* = 6.0 Hz, 1H, Ar), 8.05 (d, *J* = 9.0 Hz, 1H, Ar), 7.90 (s, 1H, Ar), 7.79 (d, *J* = 6.0 Hz, 1H, Ar), 7.64 (dd, *J* = 8.5, 1.0 Hz, 1H, Ar), 2.96 (tt, *J* = 7.5, 3.0 Hz, 2H, = CCH_2_), 2.78 (tt, *J* = 7.5, 3.0 Hz, 2H, = CCH_2_), 1.97 (tt, *J* = 7.5, 7.5 Hz, 2H, CH_2_C*H*_2_CH_2_); ^13^C NMR (100.6 MHz, DMSO-*d*_6_) δ 152.0 (=*C*–Ar), 143.1 (Ar), 139.2 (*ipso*-Ar), 134.9 (*ipso*-Ar), 127.8 (Ar), 127.4 (=*C*–CO), 126.7 (Ar), 124.8 (Ar), 120.4 (Ar), 35.3 (*=*C*C*H_2_), 21.5 (CH_2_ *C*H_2_CH_2_) (C = O, Ar, *ipso*-Ar and one *=*C*C*H_2_ resonances not resolved); HRMS (ESI) *m*/*z* calcd for C_15_H_13_NO_2_ (M + H)^+^ 240.1019, found 240.1021 (−0.7 ppm error). Lab book reference XW-001-096.

### 2-(Quinolin-7-yl)­cyclopent-1-ene-1-carboxylic Acid **20d**

Using general procedure C-1, arylated ester **19d** (58.8 mg, 0.232 mmol, 1.0 equiv), NaOH (46 mg, 1.16 mmol, 5.0 equiv) in MeOH (1.0 mL) and H_2_O (1.0 mL), using H_2_O (20 mL), CH_2_Cl_2_ (3 × 15 mL) and then EtOAc (8 × 15 mL) in the workup gave carboxylic acid **20d** (21.7 mg, 39%, ≥ 95% pure by HPLC) as a pale brown solid, mp decomposed at 220 °C; IR (ATR) 2918, 2848, 2509 (br, O–H), 1687 (C = O), 1188, 844, 475 cm^–1^; ^1^H NMR (400 MHz, DMSO-*d*_6_) δ 9.12 (d, *J* = 4.5 Hz, 1H, Ar), 8.81 (d, *J* = 8.0 Hz, 1H, Ar), 8.19–8.12 (m, 2H, Ar), 7.84 (dd, *J* = 8.0, 4.5 Hz, 1H, Ar), 7.77 (dd, *J* = 9.0, 1.0 Hz, 1H, Ar), 3.00–2.91 (m, 2H, = CCH_2_), 2.86–2.76 (m, 2H, = CCH_2_), 2.06–1.94 (m, 2H, CH_2_C*H*_2_CH_2_); ^13^C NMR (100.6 MHz, DMSO-*d*_6_) δ 166.7 (C = O), 149.3 (Ar), 147.8 (*ipso*-Ar), 140.9 (*ipso*-Ar), 132.4 (*ipso*-Ar), 128.5 (Ar), 127.9 (=*C*–CO), 121.9 (Ar), 35.3 (*=*C*C*H_2_), 21.5 (CH_2_ *C*H_2_CH_2_) (*=C*–Ar, three Ar and *=*C*C*H_2_ resonances not resolved); HRMS (ESI) *m*/*z* calcd for C_15_H_13_NO_2_ (M + H)^+^ 240.1019 found 240.1012 (+2.8 ppm error). Lab book reference AS-1-10.

### 2-(Benzo­[*d*]­thiazol-5-yl)­cyclopent-1-ene-1-carboxylic Acid **20e**

Using general procedure C-2, 2 M NaOH_(aq)_ (3.5 mL) and arylated ester **16h** (296 mg, 0.883 mmol) in MeOH (3.5 mL) and THF (3.5 mL) gave carboxylic acid **20e** (135 mg, 62%, ≥ 95% pure by ^1^H NMR) as an off-white solid, mp 184–186 °C; IR (ATR) 2489 (br, OH), 1621 (C = O), 1548, 1390, 1243 cm^–1^; ^1^H NMR (400 MHz, CDCl_3_) δ 8.80 (s, 1H, Ar), 7.98 (d, *J* = 2.0 Hz, 1H, Ar), 7.70 (d, *J* = 8.5 Hz, 1H, Ar), 7.30 (dd, *J* = 8.5, 2.0, 1H, Ar), 2.78 (t, *J* = 7.5 Hz, 2H, = CCH_2_), 2.70 (t, *J* = 7.5 Hz, 2H, = CCH_2_), 1.87 (tt, *J* = 7.5, 7.5 Hz, 2H, CH_2_ *CH*_2_CH_2_); ^13^C NMR (100.6 MHz, CDCl_3_) δ 170.4 (C = O), 154.7 (Ar), 153.9 (=C), 152.9 (Ar), 151.9 (Ar), 135.6 (=C), 133.1 (Ar), 129.5 (Ar), 127.9 (Ar), 127.6 (Ar), 40.5 (=C*CH*_2_), 35.6 (=C*CH*_2_), 22.0 (CH_2_ *CH*_2_CH_2_); HRMS (ESI) *m*/*z* calcd for C_13_H_11_NO_2_S (M–H)^−^ 244.0438, found 244.0438 (−2.7 ppm error). Lab book reference WTWB-1-44.

### 2-(Benzo­[*c*]­[1,2,5]­thiadiazol-5-yl)­cyclopent-1-ene-1-carboxylic Acid **20f**

Using general procedure C-2, 2 M NaOH_(aq)_ (3.5 mL) and arylated ester **16i** (280 mg, 0.832 mmol) in MeOH (3.5 mL) and THF (3.5 mL) gave carboxylic acid **20f** (180 mg, 88%, ≥ 95% pure by ^1^H NMR) as a yellow solid, mp 183–184 °C; IR (ATR) 2533 (br, OH), 1663 (C = O), 1625 (C = C), 1302, 1275 cm^–1^; ^1^H NMR (400 MHz, CDCl_3_) δ 7.84 (d, *J* = 2.0 Hz, 1H, Ar), 7.83 (d, *J* = 9.0 Hz, 1H, Ar), 7.48 (dd, *J* = 9.0, 2.0 Hz, 1H, Ar), 2.89 (tt, *J* = 7.5, 3.0 Hz, 2H, = CCH_2_), 2.80 (tt, *J* = 7.5, 3.0 Hz, 2H, = CCH_2_), 1.99 (tt, *J* = 7.5, 7.5 Hz, 2H, CH_2_C*H*_2_CH_2_); ^13^C NMR (100.6 MHz, CDCl_3_) δ 170.5 (C = O), 154.7 (Ar), 154.7 (Ar), 154.7 (Ar), 154.4 (=C), 138.6 (=C), 130.7 (Ar), 120.5 (Ar), 119.8 (Ar), 41.0 (=C*CH*_2_), 35.1 (=C*CH*_2_), 22.0 (CH_2_ *CH*_2_CH_2_); HRMS (ESI) *m*/*z* calcd for C_12_H_10_N_2_O_2_S (M + H)^+^ 247.0536, found 247.0536 (+4.2 ppm error). Lab book reference WTWB-1-43.

### 2-(1-Methyl-1*H*-benzo­[*d*]­imidazol-6-yl)­cyclopent-1-ene-1-carboxylic Acid **20g**

Using general procedure C-1, NaOH (118 mg, 2.95 mmol, 5.0 equiv) and arylated ester **19g** (150 mg, 0.59 mmol, 1.0 equiv) in MeOH (1.5 mL) and H_2_O (1.5 mL) at 50 °C for 6 h, using H_2_O (50 mL), EtOAc (3 × 15 mL) and then EtOAc (3 × 50 mL) in the workup gave carboxylic acid **20g** (91 mg, 64%, ≥ 95% pure by HPLC) as a red-brown solid, mp 204–206 °C; IR (ATR) 3106, 2917 (br, O–H), 2845, 1664 (C = O), 1612, 1505, 1465, 1342, 1294, 1254, 1193, 1121 cm^–1^; ^1^H NMR (400 MHz, DMSO-*d*_6_) δ 12.15 (br s, 1H, COOH), 8.19 (s, 1H, Ar), 7.57–7.55 (m, 2H, Ar), 7.22 (dd, *J* = 8.5, 1.5 Hz, 1H, Ar), 3.81 (s, 3H, NMe), 2.89 (tt, *J* = 7.5, 2.5 Hz, 2H, = CCH_2_), 2.75 (tt, *J* = 7.5, 2.5 Hz, 2H, = CCH_2_), 1.93 (tt, *J* = 7.5, 7.5 Hz, 2H, CH_2_C*H*_2_CH_2_); ^13^C NMR (100.6 MHz, CDCl_3_) δ 167.5 (C = O), 151.0 (=C–Ar), 145.2 (Ar), 142.9 (*ipso*-Ar), 134.1 (*ipso*-Ar), 131.0 (*ipso*-Ar), 128.9 (=C–CO), 122.0 (Ar), 118.3 (Ar), 109.5 (Ar), 35.4 (*=*C*C*H_2_), 30.7 (NMe), 21.4 (CH_2_ *CH*_2_CH_2_) (one (*=*C*C*H_2_) resonance not resolved); HRMS (ESI) *m*/*z* calcd for C_14_H_14_N_2_O_2_ (M + H)^+^ 243.112, found 243.1129 (−0.3 ppm error). Lab book reference XW-001-166.

### 2-(Quinolin-6-yl)­cyclopent-1-ene-1-carboxylic Acid **20h**

Using general procedure C-1, arylated ester **19f** (122.7 mg, 0.484 mmol, 1.0 equiv), NaOH (97 mg, 2.425 mmol, 5.01 equiv) in MeOH (1.0 mL) and H_2_O (1.0 mL), using H_2_O (20 mL), CH_2_Cl_2_ (3 × 15 mL) and then EtOAc (8 × 15 mL) in the workup gave carboxylic acid **20h** (21.7 mg, 39%, ≥ 95% pure by HPLC) as a pale-yellow solid, mp 198–211 °C, IR (ATR) 2455 (br, OH), 1682 (C = O), 1261, 775 cm^–1^; ^1^H NMR (400 MHz, DMSO-*d*_6_) δ 12.30 (s, 1H, COOH), 8.88 (dd, *J* = 4.0, 2.0 Hz, 1H, Ar), 8.33 (dd, *J* = 8.0, 1.0 Hz, 1H, Ar), 7.98–7.91 (m, 2H, Ar), 7.74 (dd, *J* = 9.0, 2.0 Hz, 1H, Ar), 7.52 (dd, *J* = 8.0, 4.0 Hz, 1H, Ar), 2.96–2.91 (m, 2H, = CC*H*_2_), 2.51–2.49 (m, 2H, = CC*H*_2_), 2.00–1.91 (m, 2H, CH_2_C*H*_2_CH_2_); ^13^C NMR (100.6 MHz, DMSO-*d*_6_) δ 167.0 (C = O), 150.6 (=C–Ar), 150.1 (*ipso*-Ar), 147.2 (Ar), 136.1 (Ar), 135.0 (*ipso*-Ar), 130.8 (Ar), 129.9 (Ar), 128.0 (=C–CO), 127.4 (*ipso*-Ar), 126.6 (Ar), 121.7 (Ar), 35.3 (*=*C*C*H_2_), 21.5 (CH_2_ *CH*_2_CH_2_) (*=*C*C*H_2_ resonance not resolved); HRMS (ESI) *m*/*z* calcd for C_15_H_13_NO_2_ (M + H)^+^ 240.1019 found 240.1018 (+0.3 ppm error). Lab book reference AS-1-11.

### Methyl-2-hydroxycyclopentane-1-carboxylate *cis*- and *tran*s-**21**

NaBH_4_ (1.46 g, 38.7 mmol, 1.1 equiv) was added slowly to a stirred solution of methyl 2-oxocyclopentane-1-carboxylate (5.0 g, 35.2 mmol, 1.0 equiv) in MeOH (50 mL) at 0 °C. The resulting mixture was stirred at 0 °C for 1 h. Sat. NH_4_Cl_(aq.)_ (100 mL) was added, and the mixture was extracted with EtOAc (3 × 50 mL). The combined organics were washed with sat. brine (50 mL), dried (MgSO_4_), and evaporated under reduced pressure to give the crude product. Purification by flash column chromatography on silica with 83:17 *n-*hexane-EtOAc as eluent gave alcohol *cis*-**21** (1.335 g, 26%) as a yellow oil, *R*_F_ (5:1 *n-*hexane-EtOAc) 0.26; IR (ATR) 3452 (O–H), 2953, 1721 (C = O), 1437, 1199 cm^–1^; ^1^H NMR (400 MHz, CDCl_3_) δ 4.45–4.42 (m, 1H, C*H*OH), 3.72 (s, 3H, OMe), 3.01 (d, *J* = 3.5 Hz, 1H, OH), 2.69 (ddd, *J* = 10.0, 9.5, 4.5 Hz, 1H, CHCO), 2.07–1.87 (m, 3H, CH), 1.80–1.76 (m, 2H, CH), 1.68–1.58 (m, 1H, CH); ^13^C NMR (100.6 MHz, CDCl_3_) δ 175.4 (C = O), 73.8 (OCH), 51.9 (OMe), 49.6 (*C*HCO), 34.1 (CH_2_), 26.5 (CH_2_), 22.2 (CH_2_); HRMS (ESI) *m*/*z* calcd for C_7_H_12_O_3_ (M + Na)^+^ 167.0679, found 167.0682 (−2.2 ppm error), a 56:44 mixture of *trans*-**21** and *cis*-**21** (631 mg, 12%) as a colorless oil and *trans*-**21** (980 mg, 18%) as a colorless oil, *R*_F_ (5:1 *n-*hexane-EtOAc) 0.25; IR (ATR) 3423 (O–H), 2955, 1731 (C = O), 1437, 1199 cm^–1^; ^1^H NMR (400 MHz, CDCl_3_) δ 4.38 (dddd, *J* = 6.5, 6.5, 6.5, 3.0 Hz, 1H, C*H*–OH), 3.71 (s, 3H, OMe), 2.67 (ddd, *J* = 8.5, 8.5, 6.5 Hz, 1H, CH–CO), 2.13–1.96 (m, 3H,–OH, CH), 1.86–1.59 (m, 4H, CH); ^13^C NMR (100.6 MHz, CDCl_3_) δ 175.6 (C = O), 76.5 (OCH), 52.6 (OMe), 52.0 (*C*HCO), 34.3 (CH_2_), 27.3 (CH_2_), 22.1 (CH_2_); HRMS (ESI) *m*/*z* calcd for C_7_H_12_O_3_ (M + Na)^+^ 167.0679, found 167.0685 (−3.9 ppm error). Spectroscopic data consistent with those reported in the literature.^64,65^ Lab book reference XW-001-085.

NaBH_4_ (3.99 g, 105.4 mmol, 3.0 equiv) was added slowly to a stirred solution of methyl 2-oxocyclopentane-1-carboxylate (5.0 g, 35.2 mmol, 1.0 equiv) in MeOH (50 mL) at 0 °C. The resulting mixture was stirred at 0 °C for 1 h. Sat. NH_4_Cl_(aq.)_ (100 mL) was added, and the mixture was extracted with EtOAc (3 × 50 mL). The combined organic extracts were washed with sat. brine (50 mL), dried (MgSO_4_), and evaporated under reduced pressure to give the crude product. Purification by flash column chromatography on silica with 83:17 *n-*hexane-EtOAc as eluent gave a 78:22 mixture (by ^1^H NMR spectroscopy) of alcohols *trans*-**21** and *cis*-**21** (1.1 g, 22%) as a pale-yellow oil, identical (by ^1^H NMR spectroscopy) to those described above. Lab book reference XW-001-157.

### Methyl-2-(benzo­[*d*]­thiazol-6-yl)­cyclopentane-1-carboxylate *trans-* **22a**

Using general procedure D, *cis*-methyl 2-hydroxycyclopentane-1-carboxylate *cis*-**21** (129.8 mg, 0.90 mmol, 3.0 equiv), benzoxazolium salt (189.7 mg, 0.41 mmol, 1.36 equiv) and pyridine (39 μL, 0.48 mmol, 1.60 equiv) in anhydrous benzotrifluoride (2.5 mL) followed by 4,4’-di-*tert*-butyl-2,2’-bipyridine (4.0 mg, 0.015 mmol, 0.05 equiv), NiBr_2_·dme (4.6 mg, 0.015 mmol, 0.05 equiv), [Ir­(dFCF_3_ppy]_2_(dtbpy)]­PF_6_ (5.0 mg, 0.0045 mmol, 0.015 equiv), quinuclidine (58.4 mg, 0.53 mmol, 1.75 equiv) and 6-bromobenzo­[*d*] thiazole (64 mg, 0.30 mmol, 1.00 equiv) in anhydrous DMA (2.5 mL) gave the crude product. Purification by flash column chromatography on silica with 83:17 *n-*hexane-EtOAc as eluent gave *trans-* **22a** (27 mg, 34%) as a white solid, mp 60–64 °C; *R*_F_ (3:1 *n-*hexane-EtOAc) 0.48; IR (ATR) 2951, 1728 (C = O), 1473, 1435, 1197, 1169 cm^–1^; ^1^H NMR (400 MHz, CDCl_3_) δ 8.93 (br s, 1H, Ar), 8.05 (d, *J* = 8.5 Hz, 1H, Ar), 7.83 (d, *J* = 2.0 Hz, 1H, Ar), 7.40 (dd, *J* = 8.5, 2.0 Hz, 1H, Ar), 3.61 (s, 3H, OMe), 3.50 (ddd, *J* = 9.5, 9.5, 7.5 Hz, 1H, C*H*Ar), 2.91 (ddd, *J* = 9.5, 8.5, 8.5 Hz, 1H, CHCO), 2.27–2.15 (m, 2H, CH), 2.05–1.80 (m, 4H, CH); ^13^C NMR (100.6 MHz, CDCl_3_) δ 176.2 (C = O), 153.6 (Ar), 152.2 (*ipso*-Ar), 141.9 (*ipso*-Ar), 134.2 (*ipso*-Ar), 125.9 (Ar), 123.6 (Ar), 120.2 (Ar), 52.4 (OMe), 51.9 (CH), 49.9 (CH), 35.5 (CH_2_), 31.0 (CH_2_), 25.2 (CH_2_); HRMS (ESI) *m*/*z* calcd for C_14_H_15_NO_2_S (M + Na)^+^ 284.0716, found 284.0717 (−0.5 ppm error). Lab book reference XW-001-089.

Using general procedure D, *trans*-methyl 2-hydroxycyclopentane-1-carboxylate *trans*-**21** (129.8 mg, 0.90 mmol, 3.0 equiv), benzoxazolium salt (189.7 mg, 0.41 mmol, 1.36 equiv) and pyridine (39 μL, 0.48 mmol, 1.60 equiv) in anhydrous benzotrifluoride (2.5 mL) followed by 4,4’-di-*tert*-butyl-2,2’-bipyridine (4.0 mg, 0.015 mmol, 0.05 equiv), NiBr_2_·dme (4.6 mg, 0.015 mmol, 0.05 equiv), [Ir­(dFCF_3_ppy]_2_(dtbpy)]­PF_6_ (5.0 mg, 0.0045 mmol, 0.015 equiv), quinuclidine (58.4 mg, 0.53 mmol, 1.75 equiv) and 6-bromobenzo­[*d*]­thiazole (64 mg, 0.30 mmol, 1.00 equiv) in anhydrous DMA (2.5 mL) gave the crude product. Purification by flash column chromatography on silica with 83:17 *n-*hexane-EtOAc as eluent gave *trans-* **22a** (31 mg, 40%) as a white solid, identical (by ^1^H NMR spectroscopy) to that described above. Lab book reference XW-001-100.

### Methyl 2-(Isoquinolin-6-yl)­cyclopentane-1-carboxylate *trans-* **22b**

Using general procedure D, *cis*-methyl 2-hydroxycyclopentane-1-carboxylate *cis*-**21** (129.8 mg, 0.90 mmol, 3.0 equiv), benzoxazolium salt (189.7 mg, 0.41 mmol, 1.36 equiv) and pyridine (39 μL, 0.48 mmol, 1.60 equiv) in anhydrous benzotrifluoride (2.5 mL) followed by 4,4’-di-*tert*-butyl-2,2’-bipyridine (4.0 mg, 0.015 mmol, 0.05 equiv), NiBr_2_·dme (4.6 mg, 0.015 mmol, 0.05 equiv), [Ir­(dFCF_3_ppy]_2_(dtbpy)]­PF_6_ (5.0 mg, 0.0045 mmol, 0.015 equiv), quinuclidine (58.4 mg, 0.53 mmol, 1.75 equiv) and 6-bromoisoquinoline (62 mg, 0.30 mmol, 1.00 equiv) in anhydrous DMA (2.5 mL) gave the crude product. Purification by flash column chromatography on silica with 83:17 *n-*hexane-EtOAc as eluent gave *trans-* **22b** (36 mg, 48%) as a white solid, mp 66–68 °C; *R*_F_ (1:1 *n-*hexane-EtOAc) 0.30; IR (ATR) 2952, 1729 (C = O), 1631, 1435, 1197, 1163 cm^–1^; ^1^H NMR (400 MHz, CDCl_3_) δ 9.18 (br s, 1H, Ar), 8.47 (d, *J* = 6.0 Hz, 1H, Ar), 7.90 (d, *J* = 8.5 Hz, 1H, Ar), 7.64 (d, *J* = 2.0 Hz 1H, Ar), 7.58 (d, *J* = 6.0 Hz, 1H, Ar), 7.49 (dd, *J* = 8.5, 2.0 Hz, 1H, Ar), 3.59 (s, 3H, OMe), 3.53 (ddd, *J* = 9.5, 9.5, 9.5 Hz, 1H, C*H*Ar), 2.91 (ddd, *J* = 9.5, 9.0, 9.0 Hz, 1H, CHCO), 2.29–2.16 (m, 2H, CH), 2.06–1.79 (m, 4H, CH); ^13^C NMR (100.6 MHz, CDCl_3_) δ 176.1 (C = O), 152.3 (Ar), 146.6 (*ipso*-Ar), 143.4 (Ar), 136.2 (*ipso*-Ar), 128.0 (Ar), 127.8 (*ipso*-Ar), 127.4 (Ar), 124.2 (Ar), 120.5 (Ar), 52.0 (OMe), 51.9 (CH), 50.1 (CH), 35.1 (CH_2_), 31.1 (CH_2_), 25.3 (CH_2_); HRMS (ESI) *m*/*z* calcd for C_16_H_17_NO_2_ (M + H)^+^ 256.1332, found 256.1332 (−0.1 ppm error). Lab book reference XW-001-110.

### Methyl-2-(quinolin-6-yl)­cyclopentane-1-carboxylate *trans-* **22c**

Using general procedure D, a 78:22 mixture of methyl 2-hydroxycyclopentane-1-carboxylates *trans*- and *cis*-**21** (69.2 mg, 0.48 mmol, 1.6 equiv), benzoxazolium salt (221.9 mg, 0.48 mmol, 1.6 equiv) and pyridine (39 μL, 0.48 mmol, 1.60 equiv) in anhydrous benzotrifluoride (2.5 mL) followed by 4,4’-di-*tert*-butyl-2,2’-bipyridine (4.0 mg, 0.015 mmol, 0.05 equiv), NiBr_2_·dme (4.6 mg, 0.015 mmol, 0.05 equiv), [Ir­(dFCF_3_ppy]_2_(dtbpy)]­PF_6_ (5.0 mg, 0.0045 mmol, 0.015 equiv), quinuclidine (58.4 mg, 0.53 mmol, 1.75 equiv) and 6-bromoquinoline (62.4 mg, 0.30 mmol, 1.00 equiv) in anhydrous DMA (2.5 mL) gave the crude product. Two batches of crude product were combined and purification by flash column chromatography on silica with 83:17 *n-*hexane-EtOAc as eluent gave a 70:15:15 mixture (by ^1^H NMR spectroscopy) of ester *trans-* **22c**, alcohol *trans*-**21** and DMA (101 mg, i.e., 84 mg (55%) of ester *trans*-**22c**) as a yellow oil, *R*_F_ (3:1 *n-*hexane-EtOAc) 0.25; ^1^H NMR (400 MHz, CDCl_3_) for ester *trans*-**22c** δ 8.86 (dd, *J* = 4.0, 1.5 Hz, 1H, Ar), 8.11 (dd, *J* = 8.0, 1.5 Hz, 1H, Ar), 8.05 (d, *J* = 8.5 Hz, 1H, Ar), 7.65 (d, *J* = 2.0 Hz, 1H, Ar), 7.62 (dd, *J* = 8.5, 2.0 Hz, 1H, Ar), 7.38 (dd, *J* = 8.0, 4.0 Hz, 1H, Ar), 3.60 (s, 3H, OMe), 3.54 (ddd, *J* = 9.5, 9.0, 8.5 Hz, 1H, CHAr), 2.96 (ddd, *J* = 9.0, 9.0, 9.0 Hz, 1H, CHCO), 2.29–2.17 (m, 2H, CH), 2.05–1.85 (m, 4H, CH); ^13^C NMR (100.6 MHz, CDCl_3_) for ester *trans*-**22c** δ 176.3 (C = O), 150.0 (Ar), 147.4 (*ipso*-Ar), 142.4 (*ipso*-Ar), 136.0 (Ar), 129.7 (Ar), 129.5 (Ar), 128.4 (*ipso*-Ar), 125.5 (Ar), 121.3 (Ar), 52.1 (OMe), 51.9 (CH), 49.8 (CH), 35.2 (CH_2_), 31.1 (CH_2_), 25.3 (CH_2_); HRMS (ESI) *m*/*z* calcd for C_16_H_17_NO_2_ (M + H)^+^ 256.1332, found 256.1340 (+3.3 ppm error). Lab book reference XW-001-164.

### Methyl-2-(1-methyl-1*H*-benzo­[*d*]­imidazol-6-yl)­cyclopentane-1-carboxylate *trans*-**22d**

Using general procedure D, a 78:22 mixture of methyl 2-hydroxycyclopentane-1-carboxylates *trans*- and *cis*-**21** (69.2 mg, 0.48 mmol, 1.6 equiv), benzoxazolium salt (221.9 mg, 0.48 mmol, 1.6 equiv) and pyridine (39 μL, 0.48 mmol, 1.60 equiv) in anhydrous benzotrifluoride (2.5 mL) followed by 4,4’-di-*tert*-butyl-2,2’-bipyridine (4.0 mg, 0.015 mmol, 0.05 equiv), NiBr_2_·dme (4.6 mg, 0.015 mmol, 0.05 equiv), [Ir­(dFCF_3_ppy]_2_(dtbpy)]­PF_6_ (5.0 mg, 0.0045 mmol, 0.015 equiv), quinuclidine (58.4 mg, 0.53 mmol, 1.75 equiv) and 6-bromo-1-methyl-1*H*-benzo­[*d*]­imidazole (63.3 mg, 0.30 mmol, 1.00 equiv) in anhydrous DMA (2.5 mL) gave the crude product. Two batches of crude product were combined and purification by flash column chromatography on silica with 50:50–0:100 *n-*hexane-EtOAc as eluent gave impure product. Further purification by prep-TLC with 100% EtOAc as eluent gave a 56:44 mixture (by ^1^H NMR spectroscopy) of 1-methyl-1*H*-benzo­[*d*]­imidazole and ester *trans-* **22d** (10 mg, i.e., 6.0 mg (4%) of ester *trans*-**22d**) as a colorless oil, *R*_F_ (100% EtOAc) 0.3; ^1^H NMR (400 MHz, CDCl_3_) for ester *trans*-**22d** δ 7.81 (s, 1H, Ar), 7.71 (d, *J* = 8.5 Hz, 1H, Ar), 7.24 (d, *J* = 1.5 Hz, 1H, Ar), 7.17 (dd, *J* = 8.5, 1.5 Hz, 1H, Ar), 3.80 (s, 3H, NMe), 3.58 (s, 3H, OMe), 3.48 (ddd, *J* = 9.5, 9.5, 9.0 Hz, 1H, CHAr), 2.91 (ddd, *J* = 9.0, 9.0, 9.0 Hz, 1H, CHCO), 2.25–2.18 (m, 2H, CH), 2.02–1.81 (m, 4H, CH); ^13^C NMR (100.6 MHz, CDCl_3_) for ester *trans*-**22d** δ 176.5 (C = O), 143.5 (Ar), 142.5 (*ipso*-Ar), 139.2 (Ar), 121.7 (Ar), 120.2 (Ar), 107.9 (Ar), 52.6 (CH), 51.8 (OMe), 50.4 (*C*H), 35.8 (CH_2_), 31.1 (CH_2_), 31.0 (NMe), 25.2 (CH_2_) (one *ipso*-Ar resonance not resolved); HRMS (ESI) *m*/*z* calcd for C_15_H_18_N_2_O_2_ (M + H)^+^ 259.1141, found 259.1443 (−0.8 ppm error). Lab book reference XW-001-167.

### Methyl-2-(quinolin-7-yl)­cyclopentane-1-carboxylate *trans-* **22e**

Using general procedure D, a 78:22 mixture of methyl 2-hydroxycyclopentane-1-carboxylates *trans*- and *cis*-**21** (69.2 mg, 0.48 mmol, 1.6 equiv), benzoxazolium salt (221.9 mg, 0.48 mmol, 1.6 equiv) and pyridine (39 μL, 0.48 mmol, 1.60 equiv) in anhydrous benzotrifluoride (2.5 mL) followed by 4,4’-di-*tert*-butyl-2,2’-bipyridine (4.0 mg, 0.015 mmol, 0.05 equiv), NiBr_2_·dme (4.6 mg, 0.015 mmol, 0.05 equiv), [Ir­(dFCF_3_ppy]_2_(dtbpy)]­PF_6_ (5.0 mg, 0.0045 mmol, 0.015 equiv), quinuclidine (58.4 mg, 0.53 mmol, 1.75 equiv) and 7-bromoquinoline (62.4 mg, 0.30 mmol, 1.00 equiv) in anhydrous DMA (2.5 mL) gave the crude product. Two identical reactions were set up. The two batches of crude product were combined and purified by flash column chromatography on silica with 83:17 *n-*hexane-EtOAc as eluent gave a 50:50 mixture (by ^1^H NMR spectroscopy) of ester *trans-* **22e** and alcohol *trans*-**21** (78 mg, i.e., 45 mg (30%) of ester *trans*-**22e**) as a yellow oil, *R*_F_ (3:1 *n-*hexane-EtOAc) 0.3; ^1^H NMR (400 MHz, CDCl_3_) for ester *trans*-**222** δ 8.83 (dd, *J* = 4.0, 2.0 Hz, 1H, Ar), 8.08 (dd, *J* = 8.0, 2.0 Hz, 1H, Ar), 7.92 (d, *J* = 2.0 Hz 1H, Ar), 7.72 (d, *J* = 8.5 Hz, 1H, Ar), 7.42 (dd, *J* = 8.5, 2.0 Hz, 1H, Ar), 7.31 (dd, *J* = 8.0, 4.0 Hz, 1H, Ar), 3.57 (s, 3H, OMe), 3.54–3.50 (m, 1H, CHAr), 2.96 (ddd, *J* = 9.0, 9.0, 9.0 Hz, 1H, CHCO), 2.26–2.15 (m, 2H, CH), 2.03–1.90 (m, 4H, CH); ^13^C NMR (100.6 MHz, CDCl_3_) for ester *trans*-**22e** δ 176.2 (C = O), 150.4 (Ar), 148.3 (*ipso*-Ar), 145.9 (*ipso*-Ar), 135.9 (Ar), 127.9 (Ar), 127.1 (*ipso*-Ar), 127.0 (Ar), 126.6 (Ar), 120.7 (Ar), 51.9 (OMe), 51.8 (CH), 49.8 (CH), 35.1 (CH_2_), 30.9 (CH_2_), 25.2 (CH_2_); HRMS (ESI) *m*/*z* calcd for C_16_H_17_NO_2_ (M + H)^+^ 256.1329, found 256.1332 (+1.1 ppm error). Lab book reference XW-001-170.

### Methyl-2-(quinoxalin-6-yl)­cyclopentane-1-carboxylate *trans*-**22f**

Using general procedure D, a 78:22 mixture of methyl 2-hydroxycyclopentane-1-carboxylates *trans*- and *cis*-**21** (129.8 mg, 0.90 mmol, 3.0 equiv), benzoxazolium salt (189.7 mg, 0.41 mmol, 1.36 equiv) and pyridine (39 μL, 0.48 mmol, 1.60 equiv) in anhydrous benzotrifluoride (2.5 mL) followed by 4,4’-di-*tert*-butyl-2,2’-bipyridine (4.0 mg, 0.015 mmol, 0.05 equiv), NiBr_2_·dme (4.6 mg, 0.015 mmol, 0.05 equiv), [Ir­(dFCF_3_ppy]_2_(dtbpy)]­PF_6_ (5.0 mg, 0.0045 mmol, 0.015 equiv), quinuclidine (58.4 mg, 0.53 mmol, 1.75 equiv) and 6-bromoquinoxaline (62.7 mg, 0.30 mmol, 1.00 equiv) in anhydrous DMA (2.5 mL) gave the crude product. Two identical reactions were set up. The two batches of crude product were combined and purified by flash column chromatography on silica with 83:17 *n-*hexane-EtOAc as the eluent, giving the impure product. Further purification by flash column chromatography on silica with 83:17 *n-*hexane-EtOAc as eluent gave impure product. Further purification by flash column chromatography on silica with 83:17 *n-*hexane-EtOAc as eluent gave *trans-* **22f** (95 mg, 62%) as a yellow oil, *R*_F_ (3:1 *n-*hexane-EtOAc) 0.3; IR (ATR) 2952, 2873, 1729 (C = O), 1620, 1499, 1449, 1436, 1369, 1264, 1199, 1164, 1025 cm^–1^; ^1^H NMR (400 MHz, CDCl_3_) δ 8.81 (d, *J* = 2.0 Hz, 1H, Ar), 8.79 (d, *J* = 2.0 Hz, 1H, Ar), 8.04 (d, *J* = 8.5 Hz, 1H, Ar), 7.95 (d, *J* = 2.0 Hz, 1H, Ar), 7.68 (dd, *J* = 8.5, 2.0 Hz, 1H, Ar), 3.70–3.58 (m, 4H, OMe, CHAr), 2.98 (ddd, *J* = 9.0, 9.0, 9.0 Hz, 1H, CHCO), 2.33–2.16 (m, 2H, CH), 2.06–1.86 (m, 4H, CH); ^13^C NMR (100.6 MHz, CDCl_3_) δ 176.0 (C = O), 146.7 (*ipso*-Ar), 145.1 (Ar), 144.6 (Ar), 143.2 (*ipso*-Ar), 142.2 (*ipso*-Ar), 130.6 (Ar), 129.6 (Ar), 126.8 (Ar), 52.0 (OMe), 51.9 (CH), 49.7 (CH), 35.2 (CH_2_), 31.0 (CH_2_), 25.3 (CH_2_); HRMS (ESI) *m*/*z* calcd for C_15_H_16_N_2_O_2_ (M + Na)^+^ 279.1104, found 279.1103 (+0.3 ppm error). Lab book reference XW-001-163.

### Methyl 2-(2-((4-Methoxyphenyl)­sulfonyl)­hydrazineyl)­cyclopent-1-ene-1-carboxylate **23** and Methyl 2-(2-((4-Methoxyphenyl)­sulfonyl)­hydrazineylidene)­cyclopentane-1-carboxylates (*Z*)- and (*E*)-**24**

A solution of methyl 2-oxocyclopentane-1-carboxylate (300 mg, 2.11 mmol, 1.0 equiv) and 4-methoxybenzenesulfonohydrazide (426.7 mg, 2.11 mmol, 1.0 equiv) in MeOH (5 mL) was stirred at rt for 3 h. The solid was collected by filtration to give sulfonyl hydrazone **23** (610 mg, 89%) as a white solid, mp 150–152 °C; *R*_F_ (3:1 *n-*hexane-EtOAc) 0.30; IR (ATR) 3219 (NH), 2952, 1737 (C = O), 1666, 1597, 1498, 1340, 1263, 1160, 1094 cm^–1^; ^1^H NMR (400 MHz, CDCl_3_) δ 8.27 (s, 1H, NH), 7.80 (d, *J* = 9.0 Hz, 2H, Ar), 7.01 (d, *J* = 9.0 Hz, 2H, Ar), 6.23 (s, 1H, NH), 3.89 (s, 3H, OMe), 3.66 (s, 3H, OMe), 2.62 (t, *J* = 7.5 Hz, 2H, = CCH_2_), 2.51 (t, *J* = 7.5 Hz, 2H, = CCH_2_), 1.76 (tt, *J* = 7.5, 7.5 Hz, 2H, CH_2_C*H*_2_CH_2_); ^13^C NMR (100.6 MHz, CDCl_3_) δ 164.1 (C = O), 163.7 (=CN), 145.0 (*ipso*-Ar), 130.6 (Ar), 127.8 (Ar), 114.7 (*ipso*-Ar), 98.4 (=*C*CO), 55.9 (OMe), 50.8 (OMe), 32.4 (CH_2_), 30.0 (CH_2_), 20.5 (CH_2_); HRMS (ESI) *m*/*z* calcd for C_14_H_18_N_2_O_5_S (M + Na)^+^ 349.0829, found 349.0820 (+2.5 ppm error). Lab book reference XW-001-091.

A solution of methyl 2-oxocyclopentane-1-carboxylate (5.0 g, 35.1 mmol, 1.0 equiv) and 4-methoxybenzenesulfonohydrazide (7.1 g, 35.1 mmol, 1.0 equiv) in MeOH (20 mL) was stirred at rt for 3 h. The solid was collected by filtration to give a 34:33:33 mixture of sulfonyl hydrazone **23** and (*Z*)-**24** and (*E*)-**24** (10.57 g, 92%) as a white solid, ^1^H NMR (400 MHz, CDCl_3_) δ 9.05 (s, 0.34H, NH), 8.27 (s, 0.33H, NH), 7.91–7.79 (m, 2H, Ar), 7.12 (s, 0.33H, NH), 7.02–6.95 (m, 2H, Ar), 6.30 (s, 0.34H, NH), 3.89 (s, 0.99H, OMe), 3.87 (s, 2.01H, OMe), 3.71 (s, 1.02H, OMe), 3.65 (s, 0.99H, OMe), 3.63 (s, 0.99H, OMe), 3.61–3.59 (m, 0.33H, CH), 3.49–3.44 (m, 0.33H, CH), 2.64–1.69 (m, 6H, CH). Lab book reference XW-002-010
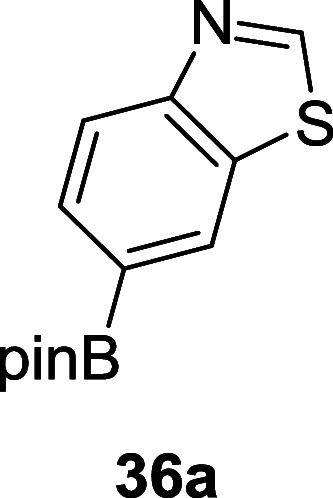
.

### 6-(4,4,5,5-Tetramethyl-1,3,2-dioxaborolan-2-yl)­benzo­[*d*]­thiazole **36a**

Using general procedure E, 6-bromobenzo­[*d*]­thiazole (1.5 g, 7.0 mmol, 1.0 equiv), bis­(pinacolato)­diboron (1.78 g, 7.0 mmol, 1.0 equiv), Pd­(OAc)_2_ (78.6 mg, 0.35 mmol, 5 mol %), dppf (194.0 mg, 0.35 mmol, 5 mol %) and KOAc (1.37 g, 14.0 mmol, 2.0 equiv) in 1,4-dioxane (20 mL), using H_2_O (100 mL), EtOAc (3 × 50 mL) and sat. brine (50 mL) in the workup gave the crude product. Purification by flash column chromatography on silica with 91:9 *n*-hexane-EtOAc as eluent gave BPin **36a** (1.784 g, 98%) as a light-yellow solid, mp 92–96 °C, *R*_F_ (5:1 *n-*hexane-EtOAc) 0.50; IR (ATR) 2978, 1597, 1474, 1442, 1386, 1350, 1290, 1144, 1096 cm^–1^; ^1^H NMR (400 MHz, CDCl_3_) δ 9.05 (s, 1H, Ar), 8.45 (br s, 1H, Ar), 8.13 (d, *J* = 8.0 Hz, 1H, Ar), 7.94 (dd, *J* = 8.0, 1.0 Hz, 1H, Ar), 1.38 (s, 12H, CMe_2_); ^13^C NMR (100.6 MHz, CDCl_3_) δ 155.6 (*ipso*-Ar), 155.3 (*ipso*-Ar), 133.5 (*ipso*-Ar), 132.1 (Ar), 129.1 (Ar), 123.1 (Ar), 84.3 (O*C*Me_2_), 25.1 (Me); ^11^B NMR (128 MHz, CDCl_3_) δ 30.17; HRMS (ESI) *m*/*z* calcd for C_13_H_16_BNO_2_S (M + Na)^+^ 262.1068, found 262.1077 (−2.6 ppm error). Lab book reference XW-001-090
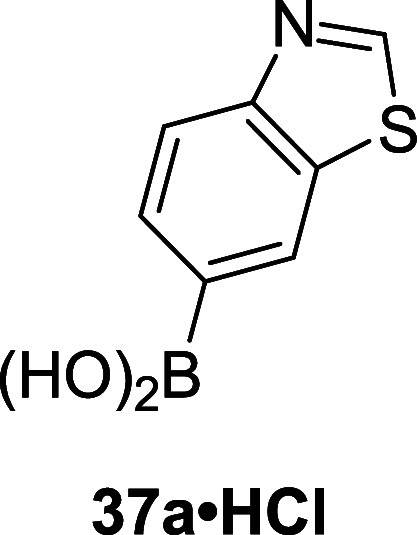
.

### Benzo­[*d*]­thiazol-6-ylboronic Acid **37a•HCl Salt**

Using general procedure F, BPin **36a** (300 mg, 1.15 mmol, 1.0 equiv) in 6 M HCl_(aq)_ (3 mL), using CH_2_Cl_2_ (3 mL) in the purification, gave boronic acid **37a•HCl** (236 mg, 96%) as a gray solid, mp >300 °C; IR (ATR) 3245 (OH), 2593, 1595, 1577, 1405, 1329, 1231, 1152, 1133 cm^–1^; ^1^H NMR (400 MHz, DMSO-*d*_6_) δ 9.43 (s, 1H, Ar), 8.52 (s, 1H, Ar), 8.04 (d, *J* = 8.0 Hz, 1H, Ar), 7.94 (d, *J* = 8.0 Hz, 1H, Ar); ^13^C NMR (100.6 MHz, DMSO-*d*_6_) δ 157.0 (Ar), 154.2 (*ipso*-Ar), 133.1 (*ipso*-Ar), 131.7 (Ar), 128.4 (Ar), 122.0 (Ar) (one *ipso*-Ar resonance not resolved); ^11^B NMR (128 MHz, DMSO-*d*_6_) δ 27.28; HRMS (ESI) *m*/*z* calcd for C_7_H_6_BNO_2_S (M - H)^−^ 178.0140, found 178.0135 (+3.1 ppm error). Lab book reference XW-001-184.

### Methyl-2-(benzo­[*d*]­thiazol-6-yl)­cyclopentane-1-carboxylate *trans-* **22a**

Using general procedure G, a 34:33:33 mixture of sulfonyl hydrazone **23** and (*Z*)-**24** and (*E*)-**24** (300 mg, 0.92 mmol, 1.0 equiv), boronic acid **37a•HCl** (247.0 mg, 1.15 mmol, 1.25 equiv) and Cs_2_CO_3_ (449.6 mg, 1.38 mmol, 1.5 equiv) in 1,4-dioxane (5 mL) gave the crude product. Purification by flash column chromatography on silica with 91:9 *n-*hexane-EtOAc as eluent gave ester *trans-* **22a** (27 mg, 11%) as a yellow oil and a 76:24 mixture of esters *trans*-**22a** and *cis*-**22a** (74 mg, 31%) as a yellow oil. In total, an 82:18 mixture of esters *trans*-**22a** and *cis*-**22a** (101 mg, 42%) was obtained. Ester *trans*-**22a** was identical (by ^1^H NMR spectroscopy) to that described above. Diagnostic signal for ester *cis*-**22a**: ^1^H NMR (400 MHz, CDCl_3_) δ 3.26–3.21 (m, 1H, CHCO). Lab book reference XW-001-186.
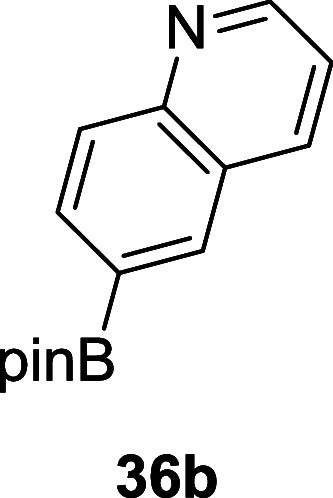
.

### 6-(4,4,5,5-Tetramethyl-1,3,2-dioxaborolan-2-yl) Quinoline **36b**

Using general procedure E, 6-bromoquinoline (2.0 g, 9.61 mmol, 1.0 equiv), bis­(pinacolato)­diboron (2.44 g, 9.61 mmol, 1.0 equiv), Pd­(OAc)_2_ (107.9 mg, 0.48 mmol, 5 mol %), dppf (266.4 mg, 0.48 mmol, 5 mol %) and KOAc (1.89 g, 19.2 mmol, 2.0 equiv) in 1,4-dioxane (20 mL), using H_2_O (300 mL), EtOAc (3 × 100 mL) and sat. brine (100 mL) in the workup gave the crude product. Purification by flash column chromatography on silica with 83:17 *n*-hexane-EtOAc as eluent gave BPin **36b** (2.29 g, 93%) as a yellow-brown oil, *R*_F_ (5:1 *n-*hexane-EtOAc) 0.20; IR (ATR) 2978, 1622, 1460, 1355, 1296, 1142, 1177 cm^–1^; ^1^H NMR (400 MHz, CDCl_3_) δ 8.94 (dd, *J* = 4.0, 2.0 Hz, 1H, Ar), 8.34 (br s, 1H, Ar), 8.19 (dd, *J* = 8.0, 2.0 Hz, 1H, Ar), 8.08–8.07 (m, 2H, Ar), 7.40 (dd, *J* = 8.0, 4.0 Hz, 1H, Ar), 1.39 (s, 12H, CMe_2_); ^13^C NMR (100.6 MHz, CDCl_3_) δ 151.5 (Ar), 149.9 (*ipso*-Ar), 136.8 (Ar), 136.3 (Ar), 134.4 (Ar), 128.6 (Ar), 127.8 (*ipso*-Ar), 121.3 (Ar), 84.3 (O*C*Me_2_), 25.1 (Me) (one *ipso*-Ar resonance not resolved); ^11^B NMR (128 MHz, CDCl_3_) δ 29.85; HRMS (ESI) *m*/*z* calcd for C_15_H_18_BNO_2_ (M + H)^+^ 256.1503, found 256.1507 (−0.3 ppm error). Lab book reference XW-002-036
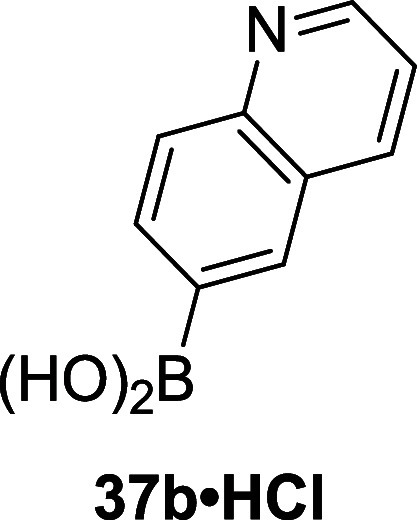
.

### Quinolin-6-ylboronic Acid **S5b•HCl** Salt

Using general procedure F, BPin **36b** (2.0 g, 7.84 mmol, 1.0 equiv) in 6 M HCl_(aq)_ (15 mL), using CH_2_Cl_2_ (10 mL) in the purification, gave boronic acid **37b•HCl** (1.52 g, 93%) as a gray solid, mp >300 °C; IR (ATR) 3335 (OH), 3139, 2564, 1636, 1589, 1556, 1454, 1407, 1343, 1294, 1202, 1122, 1044 cm^–1^; ^1^H NMR (400 MHz, DMSO-*d*_6_) δ 9.26 (d, *J* = 5.0 Hz, 1H, Ar), 9.13 (d, *J* = 8.0 Hz, 1H, Ar), 8.70 (s, 1H, Ar), 8.42 (d, *J* = 8.5 Hz, 1H, Ar), 8.28 (d, *J* = 8.5 Hz, 1H, Ar), 8.02 (dd, *J* = 8.0, 5.0 Hz, 1H, Ar); ^13^C NMR (100.6 MHz, DMSO-*d*_6_) δ 146.4 (Ar), 145.6 (Ar), 140.2 (*ipso*-Ar), 138.4 (Ar), 135.8 (Ar), 120.8 (*ipso*-Ar), 122.0 (Ar), 120.8 (Ar) (one *ipso-*Ar resonance not resolved); ^11^B NMR (128 MHz, DMSO-*d*_6_) δ 26.41; HRMS (ESI) *m*/*z* calcd for C_9_H_9_BNO_2_ M^+^ 174.0721, found 174.0722 (+0.5 ppm error). Lab book reference XW-002-041.

### Methyl-2-(quinolin-6-yl)­cyclopentane-1-carboxylate *trans-* **22c**

Using general procedure G, a 34:33:33 mixture of sulfonyl hydrazone **23** and (*Z*)-**24** and (*E*)-**24** (1.5 g, 4.6 mmol, 1.0 equiv), boronic acid **37b•HCl salt** (1.2 g, 5.8 mmol, 1.25 equiv) and Cs_2_CO_3_ (2.2 g, 6.9 mmol, 1.5 equiv) in 1,4-dioxane (15 mL) gave the crude product. Purification by flash column chromatography on silica with 75:25 *n-*hexane-EtOAc as eluent gave a 65:35 mixture (by ^1^H NMR spectroscopy) of esters *trans-* **22c** and *cis*-**22c** (302 mg, 26%) as a red oil, *R*_F_ (3:1 *n-*hexane-EtOAc) 0.25; IR (ATR) 2952, 1730 (C = O), 1500, 1435, 1197, 1167 cm^–1^; ^1^H NMR (400 MHz, CDCl_3_) δ 8.86 (dd, *J* = 4.0, 1.5 Hz, 1H, Ar), 8.10 (dd, *J* = 8.0, 1.5 Hz, 1H, Ar), 8.05 (d, *J* = 8.5 Hz, 0.65H, Ar), 8.00 (d, *J* = 8.5 Hz, 0.35H, Ar), 7.65–7.57 (m, 2H, Ar), 7.39–7.35 (m, 1H, Ar), 3.62–3.51 (m, 1H, C*H*Ar), 3.60 (s, 1.05H, OMe), 3.30–3.25 (m, 0.35H, CHCO), 3.14 (s, 1.95H, OMe), 2.95 (ddd, *J* = 9.0, 9.0, 9.0 Hz, 0.65H, CHCO), 2.29–1.85 (m, 6H, CH); ^13^C NMR (100.6 MHz, CDCl_3_) δ 176.3 (C = O), 175.1 (C = O), 150.1 (Ar), 150.0 (Ar), 147.6 (*ipso*-Ar), 147.4 (*ipso*-Ar), 142.4 (*ipso*-Ar), 140.3 (*ipso*-Ar), 136.0 (Ar), 135.9 (Ar), 130.7 (Ar), 129.8 (Ar), 129.4 (Ar), 129.0 (Ar), 128.4 (*ipso*-Ar), 128.2 (*ipso*-Ar), 125.9 (Ar), 125.5 (Ar), 121.3 (Ar), 121.2 (Ar), 52.1 (OMe), 51.9 (CH), 51.1 (OMe), 49.9 (CH), 49.8 (CH), 49.2 (CH), 35.2 (CH_2_), 31.4 (CH_2_), 31.1 (CH_2_), 28.9 (CH_2_), 25.3 (CH_2_), 24.9 (CH_2_); HRMS (ESI) *m*/*z* calcd for C_16_H_17_NO_2_ (M + H)^+^ 256.1332, found 256.1334 (−0.7 ppm error). Ester *trans*-**22c** was identical (by ^1^H NMR spectroscopy) to that described above. Lab book reference XW-002-049.

### 1-Methyl-6-(4,4,5,5-tetramethyl-1,3,2-dioxaborolan-2-yl)-1*H*-benzo­[*d*]­imidazole **36c**

Using general procedure E, 6-bromo-1-methyl-1*H*-benzo­[*d*]­imidazole (2.0 g, 9.48 mmol, 1.0 equiv), bis­(pinacolato)­diboron (2.41 g, 9.48 mmol, 1.0 equiv), Pd­(OAc)_2_ (105.5 mg, 0.47 mmol, 5 mol %), dppf (260.6 mg, 0.47 mmol, 5 mol %) and KOAc (1.86 g, 19.0 mmol, 2.0 equiv) in 1,4-dioxane (20 mL), using H_2_O (300 mL), 20:1 CH_2_Cl_2_-MeOH (3 × 100 mL) and sat. brine (100 mL) in the workup gave the crude product. Purification by flash column chromatography on silica with 95:5 CH_2_Cl_2_-MeOH as eluent gave impure product. Purification by flash column chromatography on silica with 75:25 *n*-hexane-EtOAc as eluent gave BPin **36c** (1.10 g, 93%) as a yellow solid, mp 103–105 °C; *R*_F_ (20:1 CH_2_Cl_2_-MeOH) 0.25; IR (ATR) 2978, 1619, 1498, 1367, 1349, 1291, 1240, 1143, 1108 cm^–1^; ^1^H NMR (400 MHz, CDCl_3_) δ 7.90 (br s, 2H, Ar), 7.79 (d, *J* = 8.0 Hz, 1H, Ar), 7.74 (d, *J* = 8.0 Hz, 1H, Ar), 3.87 (s, 3H, NMe), 1.38 (s, 12H, CMe_2_); ^13^C NMR (100.6 MHz, CDCl_3_) δ 146.3 (*ipso*-Ar), 144.6 (Ar), 134.5 (*ipso*-Ar), 128.4 (Ar), 119.7 (Ar), 116.3 (Ar), 83.9 (O*C*Me_2_), 31.3 (OMe), 25.0 (Me) (one *ipso*-Ar resonance not resolved); ^11^B NMR (128 MHz, CDCl_3_) δ 30.45; HRMS (ESI) *m*/*z* calcd for C_14_H_19_BN_2_O_2_ (M + H)^+^ 259.1612, found 259.1614 (+0.2 ppm error). Lab book reference XW-002-043.

### (1-Methyl-1*H*-benzo­[*d*]­imidazol-6-yl)­boronic Acid **37c•HCl** Salt

Using general procedure F, BPin **36c** (1.0 g, 3.87 mmol, 1.0 equiv) in 6 M HCl_(aq)_ (10 mL), using CH_2_Cl_2_ (5 mL) in the purification, gave boronic acid **37c•HCl** (845.8 mg, 88%) as a gray solid, mp 215–218 °C; IR (ATR) 3256 (OH), 3007, 1558, 1417, 1341, 1273, 1151, 1058, 1030 cm^–1^; ^1^H NMR (400 MHz, DMSO-*d*_6_) δ 9.59 (s, 1H, Ar), 8.32 (s, 1H, Ar), 8.01 (d, *J* = 8.0 Hz, 1H, Ar), 7.82 (d, *J* = 8.0 Hz, 1H, Ar), 4.07 (s, 1H, NMe); ^13^C NMR (100.6 MHz, DMSO-*d*_6_) δ 141.9 (Ar), 132.1 (*ipso*-Ar), 131.6 (Ar), 131.5 (*ipso*-Ar), 118.7 (Ar), 113.6 (Ar), 32.9 (NMe) (one *ipso-*Ar resonance not resolved); ^11^B NMR (128 MHz, DMSO-*d*_6_) δ 30.74; HRMS (ESI) *m*/*z* calcd for C_8_H_10_BN_2_O_2_ M^+^ 177.0830, found 177.0832 (−0.4 ppm error). Lab book reference XW-002-045.

### Methyl-2-(1-methyl-1*H*-benzo­[*d*]­imidazol-6-yl)­cyclopentane-1-carboxylate *trans*-**22d**

Using general procedure G, a 34:33:33 mixture of sulfonyl hydrazone **23** and (*Z*)-**24** and (*E*)-**24** (1.0 g, 3.06 mmol, 1.0 equiv), boronic acid **37c•HCl** (813.6 mg, 3.83 mmol, 1.25 equiv) and Cs_2_CO_3_ (1.5 g, 4.59 mmol, 1.5 equiv) in 1,4-dioxane (10 mL) gave the crude product. Purification by flash column chromatography on silica with 95:5 CH_2_Cl_2_-MeOH as eluent gave impure product. Further purification by flash column chromatography on silica with 97:3 CH_2_Cl_2_-MeOH as eluent gave impure product. Further purification by flash column chromatography on silica with 50:50–0:100 *n-*hexane-EtOAc as eluent gave a 73:22:5 mixture (by ^1^H NMR spectroscopy) of 1-methyl-*1H*-benzo­[*d*]­imidazole and esters *trans-* **22d** and *cis*-**22d** (224 mg, i.e., 93 mg (12%) of an 82:18 mixture of esters *trans-* **22d** and *cis*-**22d**) as a brown oil, *R*_F_ (20:1 CH_2_Cl_2_-MeOH) 0.2. The ratio of 1-methyl-*1H*-benzo­[*d*]­imidazole:esters *trans/cis*-**22d** was 73:27 (by ^1^H NMR spectroscopy). Diagnostic signals for ester *trans-* **22d**: ^1^H NMR (400 MHz, CDCl_3_) δ 7.81 (s, 1H, Ar), 7.72 (d, *J* = 8.5 Hz, 1H, Ar), 7.25 (d, *J* = 1.5 Hz, 1H, Ar), 7.17 (dd, *J* = 8.5, 1.5 Hz, 1H, Ar), 3.81 (s, 3H, NMe), 3.59 (s, 3H, OMe), 3.53–3.46 (m, 1H, CHAr), 2.91 (ddd, *J* = 9.0, 9.0, 9.0 Hz, 1H, CHCO), 2.25–1.80 (m, 6H, CH); diagnostic signals for ester *cis-* **22d**: ^1^H NMR (400 MHz, CDCl_3_) δ 7.81 (s, 1H, Ar), 7.67 (d, *J* = 8.5 Hz, 1H, Ar), 7.22 (d, *J* = 1.5 Hz, 1H, Ar), 7.14 (dd, *J* = 8.5, 1.5 Hz, 1H, Ar), 3.81 (s, 3H, NMe), 3.16 (s, 3H, OMe), 3.58–3.46 (m, 1H, CHAr), 3.25–3.19 (m, 1H, CHCO), 2.25–1.80 (m, 6H, CH); diagnostic signals for 1-methyl-*1H*-benzo­[*d*]­imidazole: ^1^H NMR (400 MHz, CDCl_3_) δ 7.87 (s, 1H, Ar), 7.81 (dd, *J* = 7.5, 1.5 Hz, 1H, Ar), 7.41 (dd, *J* = 7.5, 1.5 Hz, 1H, Ar), 7.34–7.27 (m, 2H, Ar), 3.85 (s, 3H, NMe); HRMS (ESI) for esters *trans/cis*-**22d** *m*/*z* calcd for C_15_H_18_N_2_O_2_ (M + H)^+^ 259.1441, found 259.1446 (−2.1 ppm error); HRMS (ESI) for 1-methyl-*1H*-benzo­[*d*]­imidazole *m*/*z* calcd for C_8_H_8_N_2_ (M + H)^+^ 133.0760, found 133.0764 (−2.9 ppm error). Ester *trans*-**22d** was identical (by ^1^H NMR spectroscopy) to that described above. Lab book reference XW-002-050
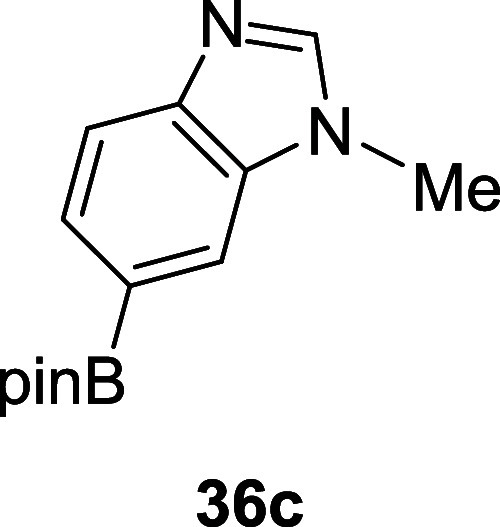
.

### 7-(4,4,5,5-Tetramethyl-1,3,2-dioxaborolan-2-yl)­quinoline **36d**

Using general procedure E, 7-bromoquinoline (2.0 g, 9.61 mmol, 1.0 equiv), bis­(pinacolato)­diboron (2.44 g, 9.61 mmol, 1.0 equiv), Pd­(OAc)_2_ (107.9 mg, 0.48 mmol, 5 mol %), dppf (266.4 mg, 0.48 mmol, 5 mol %) and KOAc (1.89 g, 19.2 mmol, 2.0 equiv) in 1,4-dioxane (20 mL), using H_2_O (300 mL), EtOAc (3 × 100 mL) and sat. brine (100 mL) in the workup gave the crude product. Purification by flash column chromatography on silica with 83:17 *n*-hexane-EtOAc as eluent gave BPin **36d** (2.29 g, 93%) as a gray solid, mp 48–52 °C; *R*_F_ (5:1 *n-*hexane-EtOAc) 0.20; IR (ATR) 2978, 1598, 1502, 1447, 1351, 1319, 1141, 1127, 1073 cm^–1^; ^1^H NMR (400 MHz, CDCl_3_) δ 8.94 (dd, *J* = 4.0, 2.0 Hz, 1H, Ar), 8.60 (br s, 1H, Ar), 8.14 (dd, *J* = 8.5, 2.0 Hz, 1H, Ar), 7.90 (dd, *J* = 8.0, 1.0 Hz, 1H, Ar), 7.79 (d, *J* = 8.0 Hz, 1H, Ar), 7.41 (dd, *J* = 8.5, 4.0 Hz, 1H, Ar), 1.39 (s, 12H, CMe_2_); ^13^C NMR (100.6 MHz, CDCl_3_) δ 150.6 (Ar), 147.9 (*ipso*-Ar), 137.5 (Ar), 135.9 (Ar), 131.2 (Ar), 130.1 (*ipso*-Ar), 127.0 (Ar), 121.9 (Ar), 84.3 (O*C*Me_2_), 25.0 (Me) (one *ipso*-Ar resonance not resolved); ^11^B NMR (128 MHz, CDCl_3_) δ 30.03; HRMS (ESI) *m*/*z* calcd for C_15_H_18_BNO_2_ (M + Na)^+^ 278.1323, found 278.1325 (+0.1 ppm error). Lab book reference XW-002-042
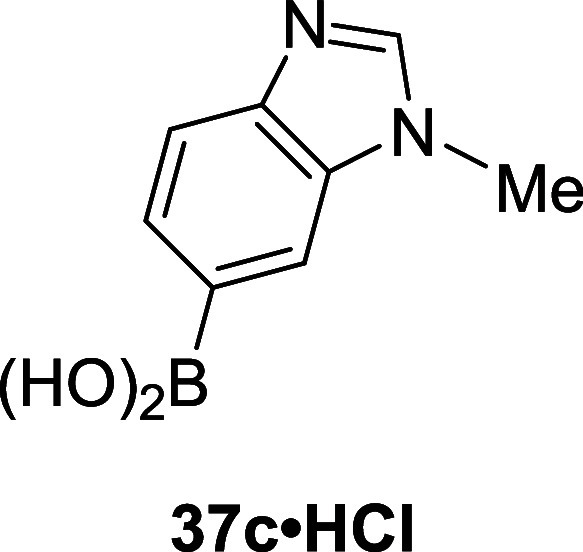
.

### Quinolin-7-ylboronic Acid **37d•HCl** Salt

Using general procedure F, BPin **36d** (1.9 g, 7.4 mmol, 1.0 equiv) in 6 M HCl_(aq)_ (15 mL), using CH_2_Cl_2_ (5 mL) in the purification, gave boronic acid **37d•HCl** (1.53 g, 98%) as a gray solid, mp 186–190 °C; IR (ATR) 3325 (OH), 2752, 1644, 1605, 1488, 1393, 1329, 1233, 1202, 1097, 1033 cm^–1^; ^1^H NMR (400 MHz, DMSO-*d*_6_) δ 9.26 (dd, *J* = 5.0, 1.5 Hz, 1H, Ar), 9.09 (d, *J* = 8.5 Hz, 1H, Ar), 8.71 (br s, 1H, Ar), 8.28 (d, *J* = 8.5 Hz, 1H, Ar), 8.23 (d, *J* = 8.5 Hz, 1H, Ar), 8.04 (dd, *J* = 8.5, 5.0 Hz, 1H, Ar); ^13^C NMR (100.6 MHz, DMSO-*d*_6_) δ 145.9 (Ar), 145.1 (Ar), 139.1 (*ipso*-Ar), 133.5 (Ar), 129.3 (Ar), 128.5 (*ipso*-Ar), 127.6 (Ar), 122.4 (Ar) (one *ipso-*Ar resonance not resolved); ^11^B NMR (128 MHz, DMSO-*d*_6_) δ 30.18; HRMS (ESI) *m*/*z* calcd for C_9_H_9_BNO_2_ M^+^ 174.0721, found 174.0726 (−1.8 ppm error). Lab book reference XW-002-051.

### Methyl-2-(quinolin-7-yl)­cyclopentane-1-carboxylate *cis-* **22e**

Using general procedure G, a 34:33:33 mixture of sulfonyl hydrazone **23** and (*Z*)-**24** and (*E*)-**24** (1.0 g, 3.06 mmol, 1.0 equiv), boronic acid **37d•HCl** (802.3 mg, 3.83 mmol, 1.25 equiv) and Cs_2_CO_3_ (1.5 g, 4.59 mmol, 1.5 equiv) in 1,4-dioxane (10 mL) gave the crude product. Purification by flash column chromatography on silica with 75:25 *n-*hexane-EtOAc as eluent gave a 65:35 mixture (by ^1^H NMR spectroscopy) of esters *cis-* **22e** and *trans*-**22e** (116 mg, 15%) as a light-orange oil, *R*_F_ (3:1 *n-*hexane-EtOAc) 0.3; IR (ATR) 2950, 1727 (C = O), 1625, 1502, 1449, 1435, 1196, 1167 cm^–1^; ^1^H NMR (400 MHz, CDCl_3_) δ 8.88–8.86 (m, 1H, Ar), 8.12–8.08 (m, 1H, Ar), 7.95 (d, *J* = 2.0 Hz, 0.35H, Ar), 7.91 (d, *J* = 2.0 Hz, 0.65H, Ar), 7.75 (d, *J* = 8.5 Hz, 0.35H, Ar), 7.71 (d, *J* = 8.5 Hz, 0.65H, Ar), 7.45 (dd, *J* = 8.5, 2.0 Hz, 0.35H, Ar), 7.41 (dd, *J* = 8.5, 2.0 Hz, 0.65H, Ar), 7.36–7.32 (m, 1H, Ar), 3.67–3.53 (m, 1H, CHAr), 3.60 (s, 1.05H, OMe), 3.31–3.25 (m, 0.65H, CHCO), 3.19 (s, 1.95H, OMe), 2.98 (ddd, *J* = 9.0, 9.0, 9.0 Hz, 0.35H, CHCO), 2.31–1.76 (m, 6H, CH); ^13^C NMR (100.6 MHz, CDCl_3_) δ 176.2 (C = O), 174.9 (C = O), 150.6 (Ar), 150.5 (Ar), 148.6 (*ipso*-Ar), 148.4 (*ipso*-Ar), 145.9 (*ipso*-Ar), 143.7 (*ipso*-Ar), 135.9 (Ar), 135.8 (Ar), 127.9 (Ar), 127.8­(Ar), 127.5 (Ar), 127.3 (Ar), 127.1 (*ipso*-Ar), 127.0 (Ar), 126.8 (Ar), 126.6 (Ar), 120.8 (Ar), 120.7 (Ar), 51.9 (OMe), 51.8 (OMe), 51.1 (CH), 49.9 (CH), 49.8 (CH), 49.3 (CH), 35.2 (CH_2_), 31.2 (CH_2_), 31.0 (CH_2_), 28.7 (CH_2_), 25.3 (CH_2_), 24.7 (CH_2_) (one *ipso*-Ar resonance not resolved); HRMS (ESI) *m*/*z* calcd for C_16_H_17_NO_2_ (M + H)^+^ 256.1332, found 256.1324 (+3.2 ppm error). Ester *trans*-**22e** was identical (by ^1^H NMR spectroscopy) to that described above. Lab book reference XW-002-053.

### 2-(Isoquinolin-6-yl)­cyclopentane-1-carboxylic Acid *trans-* **17a**

(Scheme 3) Using general procedure C-1, NaOH (50 mg, 1.25 mmol, 5.0 equiv) and arylated ester *trans*-**22b** (65 mg, 0.74 mmol, 1.0 equiv) in MeOH (1.0 mL) and H_2_O (1.0 mL) at 50 °C for 6 h, using H_2_O (20 mL), EtOAc (3 × 10 mL) and then EtOAc (3 × 20 mL) in the workup gave carboxylic acid *trans-* **17a** (53.7 mg, 87%, ≥ 95% pure by HPLC) as a light-yellow solid, mp 154–158 °C; IR (ATR) 2955 (br, O–H), 1710 (C = O), 1632, 1202 cm^–1^; ^1^H NMR (400 MHz, DMSO-*d*_6_) δ 9.18 (br s, 1H, Ar), 8.42 (d, *J* = 6.0 Hz, 1H, Ar), 7.90 (d, *J* = 8.5 Hz, 1H, Ar), 7.69 (s, 1H, Ar), 7.59 (d, *J* = 6.0 Hz, 1H, Ar), 7.55 (dd, *J* = 8.5, 1.5 Hz, 1H, Ar), 3.53 (ddd, *J* = 9.5, 9.5, 9.5 Hz, 1H, CHAr), 2.91 (ddd, *J* = 9.0, 9.0, 9.0 Hz, 1H, CHCO), 2.31–2.23 (m, 2H, CH), 2.14–2.05 (m, 1H, CH), 1.98–1.82 (m, 3H, CH); ^13^C NMR (100.6 MHz, CDCl_3_) δ 175.0 (C = O), 151.5 (Ar), 147.4 (*ipso*-Ar), 141.9 (Ar), 136.4 (*ipso*-Ar), 128.3 (Ar), 127.8 (Ar), 127.7 (*ipso*-Ar), 124.3 (Ar), 120.9 (Ar), 51.9 (CH), 50.1 (CH), 35.3 (CH_2_), 31.2 (CH_2_), 25.4 (CH_2_); HRMS (ESI) *m*/*z* calcd for C_15_H_15_NO_2_ (M + H)^+^ 242.1176, found 242.1181 (−2.4 ppm error). Lab book reference XW-001-115.

### 2-(1-Methyl-1*H*-benzo­[*d*]­imidazol-6-yl)­cyclopentane-1-carboxylic Acid *trans*-**17b**

(Scheme 4) A suspension of a 73:22:5 mixture of 1-methyl-1*H*-benzo­[*d*]­imidazole and esters *trans-* **22d** and *cis*-**22d** (200 mg, i.e., 76 mg of an 82:18 mixture of esters *trans-* **22d** and *cis*-**22d**, 0.29 mmol, 1.0 equiv) and NaOMe (156.7 mg, 2.9 mmol, 10.0 equiv) in MeOH (6 mL) was stirred and heated at reflux for 16 h. After being allowed to cool to rt, the mixture was diluted with H_2_O (20 mL) and extracted with EtOAc (3 × 10 mL). The combined organic extracts were washed with sat. brine (10 mL), dried (MgSO_4_), and evaporated under reduced pressure to give recovered 1-methyl-1*H*-benzo­[*d*]­imidazole (112 mg, 90%) as a yellow-brown oil, *R*_F_ (20:1 CH_2_Cl_2_-MeOH) 0.30; ^1^H NMR (400 MHz, CDCl_3_) δ 7.86 (s, 1H, Ar), 7.81 (dd, *J* = 7.5, 1.5 Hz, 1H, Ar), 7.39 (dd, *J* = 7.5, 1.5 Hz, 1H, Ar), 7.34–7.27 (m, 2H, Ar), 3.84 (s, 3H, NMe); ^13^C NMR (100.6 MHz, CDCl_3_) δ 143.9 (*ipso*-Ar), 143.7 (Ar), 134.7 (*ipso*-Ar), 123.0 (Ar), 122.2 (Ar), 120.4 (Ar), 109.4 (Ar), 31.1 (NMe). The aqueous layer was acidified with 2 M HCl_(aq)_ until pH = 3–5, and the solvent was evaporated under reduced pressure to give the crude product. The crude product was triturated using EtOH (2 mL) and, after standing at rt for 15 min, the solid was collected by filtration to give impure product. The impure product was triturated using EtOAc (2 mL) and, after standing at rt for 15 min, the solid was collected by filtration to give carboxylic acid *trans*-**17b** (53 mg, 74%, ≥ 95% pure by HPLC) as a brown solid, mp 218–220 °C; IR (ATR) 2625 (br, O–H), 1714 (C = O), 1556, 1468, 1427, 1359, 1258, 1177, 1148 cm^–1^; ^1^H NMR (400 MHz, DMSO-*d*_6_) δ 9.50 (s, 1H, Ar), 7.88 (d, *J* = 1.5 Hz, 1H, Ar), 7.77 (d, *J* = 8.5 Hz, 1H, Ar), 7.52 (dd, *J* = 8.5, 1.5 Hz, 1H, Ar), 4.04 (s, 3H, NMe), 3.41 (ddd, *J* = 9.5, 9.5, 9.5 Hz 1H, CHAr), 2.91 (ddd, *J* = 9.5, 9.0, 9.0 Hz, 1H, CHCO), 2.19–1.72 (m, 6H, CH); ^13^C NMR (100.6 MHz, DMSO-*d*_6_) δ 176.2 (C = O), 142.6 (*ipso*-Ar), 141.9 (Ar), 132.2 (*ipso*-Ar), 129.8 (*ipso*-Ar), 126.1 (Ar), 114.7 (Ar), 110.9 (Ar), 51.6 (CH), 51.9 (CH), 35.6 (CH_2_), 32.8 (NMe), 30.3 (CH_2_), 24.6 (CH_2_); HRMS (ESI) *m*/*z* calcd for C_14_H_16_N_2_O_2_ (M + H)^+^ 245.1285, found 245.1285 (−0.2 ppm error). Spectroscopic data for 1-methyl-1*H*-benzo­[*d*]­imidazole, consistent with those reported in the literature.^66^ Lab book reference XW-002-061.

### 2-(Benzo­[*d*]­thiazol-6-yl)­cyclopentane-1-carboxylic Acid *trans*-**17g**

(Scheme 3) Using general procedure C-1, NaOH (42 mg, 1.05 mmol, 5.0 equiv) and arylated ester *trans-* **22a** (56 mg, 0.21 mmol, 1.0 equiv) in MeOH (1.0 mL) and H_2_O (1.0 mL), using H_2_O (20 mL), EtOAc (3 × 10 mL) and then EtOAc (3 × 20 mL) in the workup gave carboxylic acid *trans-* **17g** (50 mg, 94%, ≥ 95% pure by HPLC) as a white solid, mp 160–162 °C; IR (ATR) 2925 (br, O–H), 1703 (C = O), 1473 cm^–1^; ^1^H NMR (400 MHz, CDCl_3_) δ 8.94 (br s, 1H, Ar), 8.05 (d, *J* = 8.5 Hz, 1H, Ar), 7.84 (d, *J* = 2.0 Hz, 1H, Ar), 7.41 (dd, *J* = 8.5, 2.0 Hz, 1H, Ar), 3.50 (ddd, *J* = 9.5, 9.0, 8.0 Hz 1H, CHAr), 2.94 (ddd, *J* = 9.0, 9.0, 9.0 Hz, 1H, CHCO), 2.29–2.19 (m, 2H, CH), 2.10–2.00 (m, 1H, CH), 1.96–1.77 (m, 3H, CH); ^13^C NMR (100.6 MHz, CDCl_3_) δ 180.0 (C = O), 153.8 (Ar), 152.1 (*ipso*-Ar), 141.7 (*ipso*-Ar), 134.2 (*ipso*-Ar), 125.9 (Ar), 123.6 (Ar), 120.3 (Ar), 52.0 (CH), 49.8 (CH), 35.7 (CH_2_), 31.0 (CH_2_), 25.3 (CH_2_); HRMS (ESI) *m*/*z* calcd for C_13_H_13_NO_2_S (M + Na)^+^ 270.0559, found 270.0567 (−2.8 ppm error). Lab book reference XW-001-106.

(Scheme 4) Using general procedure H, a 77:23 mixture of esters *trans*-**22a** and *cis*-**22a** (100 mg, 0.38 mmol, 1.0 equiv) and NaOMe (205.2 mg, 3.8 mmol, 10.0 equiv) in MeOH (3 mL) gave carboxylic acid *trans*-**17g** (88.5 mg, 94%) as a white solid, identical (by ^1^H NMR spectroscopy) to that described above. Lab book reference XW-002-057.

### 2-(Quinoxalin-6-yl)­cyclopentane-1-carboxylic Acid *trans*-**17h**

(Scheme 3) Using general procedure C-1, NaOH (62 mg, 1.55 mmol, 5.0 equiv) and arylated ester *trans-* **22f** (80 mg, 0.31 mmol, 1.0 equiv) in MeOH (1.0 mL) and H_2_O (1.0 mL), using H_2_O (30 mL), EtOAc (3 × 10 mL) and then 10:1 CH_2_Cl_2_-MeOH (4 × 50 mL) in the workup gave carboxylic acid *trans-* **17h** (70 mg, 93%, ≥ 95% pure by HPLC) as a pale-yellow solid, mp 95–120 °C; IR (ATR) 2954 (br, O–H), 1717 (C = O), 1500, 1451, 1372, 1200 cm^–1^; ^1^H NMR (400 MHz, CDCl_3_) δ 8.83–8.81 (m, 2H, Ar), 8.06 (d, *J* = 9.0 Hz, 1H, Ar), 8.03 (d, *J* = 2.0 Hz, 1H, Ar), 7.72 (dd, *J* = 9.0, 2.0 Hz, 1H, Ar), 3.64 (ddd, *J* = 9.0, 9.0, 9.0 Hz 1H, CHAr), 3.02 (ddd, *J* = 9.0, 9.0, 9.0 Hz, 1H, CHCO), 2.35–2.24 (m, 2H, CH), 2.13–2.06 (m, 1H, CH), 1.99–1.86 (m, 3H, CH); ^13^C NMR (100.6 MHz, CDCl_3_) δ 179.5 (C = O), 146.8 (*ipso*-Ar), 144.6 (Ar), 144.5 (Ar), 142.6 (*ipso*-Ar), 142.1 (*ipso*-Ar), 130.5 (Ar), 129.5 (Ar), 126.6 (Ar), 51.7 (CH), 49.6 (CH), 35.1 (CH_2_), 31.0 (CH_2_), 25.3 (CH_2_); HRMS (ESI) *m*/*z* calcd for C_14_H_14_N_2_O_2_ (M + Na)^+^ 265.0947, found 265.0952 (−1.5 ppm error). Lab book reference XW-001-169.

### 2-(Quinolin-6-yl)­cyclopentane-1-carboxylic Acid *trans*-**17i**

(Scheme 4) Using general procedure H, a 65:35 mixture of esters *trans*-**22b** and *cis*-**22b** (250 mg, 0.98 mmol, 1.0 equiv) and NaOMe (529.2 mg, 9.8 mmol, 10.0 equiv) in MeOH (8 mL) gave carboxylic acid *trans*-**17i** (156 mg, 66%, ≥ 95% pure by HPLC) as a pale-yellow solid, mp 130–132 °C; IR (ATR) 2951 (br, O–H), 1700 (C = O), 1503, 1272, 1204 cm^–1^; ^1^H NMR (400 MHz, CDCl_3_) δ 8.85 (d, *J* = 4.5 Hz, 1H, Ar), 8.13 (dd, *J* = 8.5, 1.5 Hz, 1H, Ar), 7.99 (d, *J* = 8.5 Hz, 1H, Ar), 7.69 (d, *J* = 2.0 Hz, 1H, Ar), 7.61 (dd, *J* = 8.5, 2.0 Hz, 1H, Ar), 7.38 (dd, *J* = 8.5, 4.5 Hz, 1H, Ar), 3.55 (ddd, *J* = 9.0, 9.0, 9.0 Hz 1H, C*H*Ar), 2.97 (ddd, *J* = 9.0, 9.0, 9.0 Hz, 1H, CHCO), 2.30–1.85 (m, 6H, CH); ^13^C NMR (100.6 MHz, CDCl_3_) δ 179.8 (C = O), 149.1 (Ar), 146.3 (*ipso*-Ar), 142.8 (*ipso*-Ar), 136.9 (Ar), 130.3 (Ar), 128.5 (Ar), 125.4 (Ar), 121.3 (Ar), 52.3 (CH), 49.8 (CH), 35.1 (CH_2_), 31.1 (CH_2_), 25.2 (CH_2_) (one *ipso*-Ar resonance not resolved); HRMS (ESI) *m*/*z* calcd for C_15_H_15_NO_2_ (M + Na)^+^ 242.1176, found 242.1178 (−1.1 ppm error). The *trans* configuration was confirmed by X-ray crystallography: CCDC 2482171. Lab book reference XW-002-054.

(Scheme 3) Using general procedure C-1, NaOH (62 mg, 1.55 mmol, 5.0 equiv) and a 70:15:15 mixture of ester *trans-* **22c**, alcohol *trans*-**21** and DMA (80 mg, i.e., 67 mg of ester *trans*-**22c**, 0.31 mmol, 1.0 equiv) in MeOH (1.0 mL) and H_2_O (1.0 mL), using H_2_O (30 mL), EtOAc (3 × 10 mL) and then 10:1 CH_2_Cl_2_-MeOH (4 × 50 mL) in the workup gave carboxylic acid *trans-* **17i** (33 mg, 53%) as a yellow-brown solid, identical (by ^1^H NMR spectroscopy) to that described above. Lab book reference XW-001-168.

### 2-(Quinolin-7-yl)­cyclopentane-1-carboxylic Acid *trans-* **17j**

(Scheme 4) Using general procedure H, a 65:35 mixture of esters *cis*-**22e** and *trans*-**22e** (100 mg, 0.39 mmol, 1.0 equiv) and NaOMe (210.7 mg, 3.9 mmol, 10.0 equiv) in MeOH (3.1 mL) gave carboxylic acid *trans*-**17j** (80 mg, 82%, ≥ 95% pure by HPLC) as a pale-yellow solid, mp 133–136 °C; IR (ATR) 2954 (br, O–H), 1707 (C = O), 1626, 1503, 1452, 1278, 1205 cm^–1^; ^1^H NMR (400 MHz, CDCl_3_) δ 8.90 (d, *J* = 4.5 Hz, 1H, Ar), 8.27 (s, 1H, Ar), 8.16 (d, *J* = 8.0 Hz, 1H, Ar), 7.77 (d, *J* = 8.0 Hz, 1H, Ar), 7.55 (d, *J* = 8.5 Hz, 1H, Ar), 7.37 (dd, *J* = 8.0, 4.5 Hz, 1H, Ar), 3.76 (ddd, *J* = 10.0, 10.0, 9.5 Hz 1H, CHAr), 3.05 (ddd, *J* = 9.5, 9.5, 9.5 Hz, 1H, CHCO), 2.39–1.86 (m, 6H, CH); ^13^C NMR (100.6 MHz, CDCl_3_) δ 178.7 (C = O), 148.8 (Ar), 146.9 (*ipso*-Ar), 146.5 (*ipso*-Ar), 137.5 (Ar), 128.0 (Ar), 127.2 (*ipso*-Ar), 126.8 (Ar), 125.7 (Ar), 120.7 (Ar), 52.5 (CH), 50.2 (CH), 34.5 (CH_2_), 31.1 (CH_2_), 25.1 (CH_2_); HRMS (ESI) *m*/*z* calcd for C_15_H_15_NO_2_ (M + Na)^+^ 242.1176, found 242.1179 (−1.6 ppm error). Lab book reference XW-002-062.

### 6-(4,4,5,5-Tetramethyl-1,3,2-dioxaborolan-2-yl)­benzo­[*d*]­thiazol-2-amine **29**

Bis­(pinacolato)­diboron (495.2 mg, 1.95 mmol, 1.5 equiv), 6-bromobenzo­[*d*]­thiazol-2-amine **28** (300 mg, 1.30 mmol, 1.0 equiv), Pd­(PPh_3_)_2_Cl_2_ (46.0 mg, 0.065 mmol, 0.05 equiv) and KOAc (255.2 mg, 2.60 mmol, 2.0 equiv) were added to a round-bottomed flask. The round-bottomed flask was purged with N_2_, and then dry, degassed 1,4-dioxane (5 mL) was added. The resulting mixture was stirred and heated at 110 °C (oil bath) for 16 h under N_2_. After being allowed to cool to rt, the solids were removed by filtration through Celite and washed with EtOAc (30 mL). The filtrate was diluted with H_2_O (30 mL) and extracted with EtOAc (3 × 15 mL). The combined organic extracts were washed with sat. brine (15 mL), dried (MgSO_4_), and evaporated under reduced pressure to give the crude product. Purification by flash column chromatography on silica with 67:33 hexane-EtOAc as eluent gave impure product. The impure product was triturated using *n*-hexane (5 mL) and, after standing at rt, the solid was collected by filtration to give amino benzothiazole Bpin **29** (274 mg, 76%) as a gray solid, mp 175–181 °C, *R*_F_ (3:1 *n*-hexane-EtOAc) 0.10; IR (ATR) 3317 (NH), 3124 (NH), 2978, 1632, 1597, 1557, 1526, 1346, 1296, 1142 cm^–1^; ^1^H NMR (400 MHz, CDCl_3_) δ 8.06 (s, 1H, Ar), 7.75 (d, *J* = 8.0 Hz, 1H, Ar), 7.51 (d, *J* = 8.0 Hz, 1H, Ar), 5.59 (br s, 2H, NH_2_), 1.35 (s, 12H, CMe_2_); ^13^C NMR (100.6 MHz, CDCl_3_) δ 167.4 (Ar), 154.6 (*ipso*-Ar), 132.6 (Ar), 131.4 (*ipso*-Ar), 127.9 (Ar), 118.7 (Ar), 83.9 (O*C*Me_2_), 25.0 (Me) (one *ipso*-Ar resonance not resolved); ^11^B NMR (128 MHz, CDCl_3_) δ 30.57; HRMS (ESI) *m*/*z* calcd for C_13_H_17_BN_2_O_2_S (M + H)^+^ 277.1177, found 277.1177 (+0.8 ppm error). Lab book reference XW-002-097.

Bis­(pinacolato)­diboron (4.99 g, 19.65 mmol, 1.5 equiv), 6-bromobenzo­[*d*]­thiazol-2-amine **28** (3.0 g, 13.10 mmol, 1.0 equiv), Pd­(PPh_3_)_2_Cl_2_ (459.7 mg, 0.65 mmol, 0.05 equiv), and KOAc (2.57 g, 26.2 mmol, 2.0 equiv) were added to a round-bottomed flask. The round-bottomed flask was purged with N_2_, and then dry, degassed 1,4-dioxane (30 mL) was added. The resulting mixture was stirred and heated at 110 °C (oil bath) for 16 h under N_2_. After being allowed to cool to rt, the solids were removed by filtration through Celite and washed with EtOAc (300 mL). The filtrate was washed with 1 M HCl_(aq)_ (3 × 100 mL). The combined aqueous phases were basified with Na_2_CO_3_ powder until pH ≥ 8, and extracted with EtOAc (3 × 100 mL). The combined organic extracts were washed with sat. brine (100 mL), dried (MgSO_4_), and evaporated under reduced pressure to give amino benzothiazole Bpin **29** (2.97 g, 82%) as a yellow solid, identical (by ^1^H NMR spectroscopy) to that described above. Lab book reference XW-002-130.

### Methyl 2-(2-Aminobenzo­[*d*]­thiazol-6-yl)­cyclopent-1-ene-1-carboxylate **30a**

Enol triflate **18** (2.5 g, 9.12 mmol, 1.0 equiv), amino benzothiazole Bpin **29** (3.0 g, 10.94 mmol, 1.2 equiv), Pd­(PPh_3_)_2_Cl_2_ (640.1 mg, 0.91 mmol, 0.1 equiv) and K_2_CO_3_ (3.78 g, 27.36 mmol, 3.0 equiv) were added to a round-bottomed flask. The round-bottomed flask was purged with N_2_, and then dry, degassed 1,4-dioxane (25 mL) and H_2_O (25 mL) were added. The resulting mixture was stirred and heated at 80 °C (oil bath) for 16 h under N_2_. After being allowed to cool to rt, the mixture was diluted with H_2_O (150 mL) and extracted with EtOAc (3 × 50 mL). The combined organics were washed with 1 M HCl_(aq)_ (3 × 50 mL), and the combined aqueous phases were basified with Na_2_CO_3_ powder until pH ≥ 8, and extracted with EtOAc (3 × 50 mL). The combined organic extracts were stirred with 1 M LiOH_(aq)_ (200 mL) at rt for 20 min. Then, the two layers were separated, and the organic layer was washed with H_2_O (2 × 50 mL), sat. brine (100 mL), dried (MgSO_4_), and evaporated under reduced pressure to give arylated ester **30a** (2.0 g, 80%) as a yellow-brown solid, mp 103–105 °C, *R*_F_ (2:1 hexane-EtOAc) 0.15; IR (ATR) 3322 (NH), 3121 (NH), 2949, 1694 (C = O), 1622, 1526, 1461, 1144, 1306, 1256, 1223, 1122 cm^–1^; ^1^H NMR (400 MHz, CDCl_3_) δ 7.62 (d, *J* = 2.0 Hz, 1H, Ar), 7.45 (d, *J* = 8.5 Hz, 1H, Ar), 7.28 (dd, *J* = 8.5, 2.0 Hz, 1H, Ar), 5.81 (br s, 2H, NH_2_), 3.54 (s, 3H, OMe), 2.89–2.81 (m, 4H, = CCH_2_), 1.98 (tt, *J* = 7.5, 7.5 Hz, 2H, CH_2_C*H*_2_CH_2_); ^13^C NMR (100.6 MHz, CDCl_3_) δ 167.0 (C = O), 167.0 (*ipso*-Ar), 153.5 (=*C–*Ar), 152.0 (*ipso*-Ar), 131.2 (*ipso*-Ar), 130.9 (*ipso*-Ar), 128.3 (=*C*–CO), 126.3 (Ar), 120.6 (Ar), 118.2 (Ar), 51.4 (OMe), 40.3 (=C*C*H_2_), 35.4 (=C*C*H_2_), 22.0 (CH_2_ *C*H_2_CH_2_); HRMS (ESI) *m*/*z* calcd for C_14_H_14_N_2_O_2_S (M + H)^+^ 275.0857, found 275.0849 (−3.0 ppm error). Lab book reference XW-002-131.

### Methyl 2-(2-(Methylamino)­benzo­[*d*]­thiazol-6-yl)­cyclopent-1-ene-1-carboxylate **30b**

Paraformaldehyde (66.1 mg, 2.20 mmol, 2.0 equiv) was added to a stirred mixture of arylated ester **30a** (300 mg, 1.10 mmol, 1.0 equiv) and NaOMe (297.1 mg, 5.5 mmol, 5.0 equiv) in dry MeOH (3 mL) at rt. The resulting mixture was stirred at rt for 16 h. Then, NaBH_4_ (124.8 mg, 3.30 mmol, 3.0 equiv) was added slowly. The resulting mixture was stirred and heated at reflux for 5 h. After being allowed to cool to rt, H_2_O (50 mL) was added. The mixture was extracted with EtOAc (3 × 20 mL). The combined organics were washed with sat. brine (20 mL), dried (MgSO_4_), and evaporated under reduced pressure to give the crude product. Purification by flash column chromatography on silica with 67:33 *n-*hexane-EtOAc (with 0.1% Et_3_N) as eluent gave arylated ester **30b** (165 mg, 52%) as a white solid, mp 125–127 °C, *R*_F_ (1:1 hexane-EtOAc, and 0.1% Et_3_N) 0.30; IR (ATR) 3201 (NH), 3102 (NH), 2947, 2845, 1715 (C = O), 1623, 1583, 1547, 1465, 1434, 1414, 1298, 1251, 1121 cm^–1^; ^1^H NMR (400 MHz, CDCl_3_) δ 7.65 (d, *J* = 2.0 Hz, 1H, Ar), 7.47 (d, *J* = 8.5 Hz, 1H, Ar), 7.30 (dd, *J* = 8.5, 2.0 Hz, 1H, Ar), 5.80 (br s, 1H, NH), 3.54 (s, 3H, OMe), 3.10 (s, 3H, NMe), 2.91–2.81 (m, 4H, = CCH_2_), 1.98 (tt, *J* = 7.5, 7.5 Hz, 2H, CH_2_C*H*_2_CH_2_); ^13^C NMR (100.6 MHz, CDCl_3_) δ 169.2 (*ipso*-Ar), 167.0 (C = O), 153.3 (=*C–*Ar), 152.5 (*ipso*-Ar), 130.2 (*ipso*-Ar), 128.1 (=*C*–CO), 126.4 (Ar), 120.6 (Ar), 118.0 (Ar), 51.3 (OMe), 40.3 (=CCH_2_), 35.5 (=CCH_2_), 31.9 (NMe), 22.1 (CH_2_ *C*H_2_CH_2_) (one *ipso*-Ar resonance not resolved); HRMS (ESI) *m*/*z* calcd for C_15_H_16_N_2_O_2_S (M + H)^+^ 289.1004, found 289.1005 (+3.0 ppm error). Lab book reference XW-002-135.

### Methyl 2-(2-(Ethylamino)­benzo­[*d*]­thiazol-6-yl)­cyclopent-1-ene-1-carboxylate **30c**

Acetaldehyde (96.9 mg, 2.20 mmol, 2.0 equiv) was added to a stirred mixture of arylated ester **30a** (300 mg, 1.10 mmol, 1.0 equiv) and NaOMe (297.1 mg, 5.5 mmol, 5.0 equiv) in dry MeOH (3 mL) at rt. The resulting mixture was stirred at rt for 16 h. Then, NaBH_4_ (41.6 mg, 1.10 mmol, 1.0 equiv) was added slowly. The resulting mixture was stirred and heated at reflux for 1 h. After being allowed to cool to rt, H_2_O (50 mL) was added. The mixture was extracted with EtOAc (3 × 20 mL). The combined organics were washed with sat. brine (20 mL), dried (MgSO_4_), and evaporated under reduced pressure to give the crude product. Purification by flash column chromatography on silica with 75:25–67:33–50:50 *n-*hexane-EtOAc (with 0.1% Et_3_N) as eluent gave arylated ester **30c** (76.5 mg, 23%) as a white solid, mp 103–105 °C, *R*_F_ (1:1 hexane-EtOAc) 0.65; IR (ATR) 3349 (NH), 3205 (NH), 2948, 1697 (C = O), 1604, 1568, 1535, 1460, 1434, 1349, 1310, 1256, 1212, 1122 cm^–1^; ^1^H NMR (400 MHz, CDCl_3_) δ 7.64 (d, *J* = 2.0 Hz, 1H, Ar), 7.46 (d, *J* = 8.5 Hz, 1H, Ar), 7.29 (dd, *J* = 8.5, 2.0 Hz, 1H, Ar), 5.50 (br s, 1H, NH), 3.64 (s, 3H, OMe), 3.47 (q, *J* = 7.0 Hz, 2H, *CH*_2_Me), 2.90–2.81 (m, 4H, = CCH_2_), 1.98 (tt, *J* = 7.5, 7.5 Hz, 2H, CH_2_C*H*_2_CH_2_), 1.33 (t, *J* = 7.0 Hz, 3H, CH_2_ *Me*); ^13^C NMR (100.6 MHz, CDCl_3_) δ 168.1 (*ipso*-Ar), 167.0 (C = O), 153.3 (=*C–*Ar), 152.4 (*ipso*-Ar), 130.2 (*ipso*-Ar), 130.1 (*ipso*-Ar), 128.1 (=*C*–CO), 126.4 (Ar), 120.6 (Ar), 118.0 (Ar), 51.3 (OMe), 40.5 (N*CH*_2_Me), 40.3 (=CCH_2_), 35.5 (=CCH_2_), 22.1 (CH_2_ *C*H_2_CH_2_), 15.0 (NCH_2_ *Me*); HRMS (ESI) *m*/*z* calcd for C_16_H_18_N_2_O_2_S (M + H)^+^ 303.1164, found 303.1162 (−0.7 ppm error). Lab book reference XW-002-134.

### 2-(2-Aminobenzo­[*d*]­thiazol-6-yl)­cyclopent-1-ene-1-carboxylic Acid **26a**

Using general procedure C-1, NaOH (66 mg, 1.65 mmol, 5.0 equiv) and arylated ester **30a** (90 mg, 0.33 mmol, 1.0 equiv) in MeOH (1.5 mL) and H_2_O (1.5 mL), using H_2_O (30 mL), EtOAc (3 × 10 mL) and then EtOAc (8 × 30 mL) in the workup, gave carboxylic acid **26a** (48 mg, 56%, ≥ 95% pure by HPLC) as a yellow-gray solid, mp 255–260 °C; IR (ATR) 3379 (NH), 3171 (NH), 2922, 2848, 1615 (C = O), 1584, 1535, 1463, 1342, 1272, 1129 cm^–1^; ^1^H NMR (400 MHz, DMSO-*d*_6_) δ 12.14 (br s, 1H, COOH), 7.68 (br s, 1H, Ar), 8.02 (br s, 2H, NH_2_), 7.24 (br s, 2H, Ar), 2.82 (m, 2H, = CCH_2_), 2.71 (m, 2H, = CCH_2_), 1.88 (m, 2H, CH_2_C*H*_2_CH_2_); ^13^C NMR (100.6 MHz, DMSO-*d*_6_) δ 167.5 (*ipso*-Ar), 167.0 (C = O), 152.6 (*ipso*-Ar), 150.2 (=*C–*Ar), 130.4 (*ipso*-Ar), 129.1 (*ipso*-Ar), 128.2 (=*C*–CO), 125.8 (Ar), 120.4 (Ar), 116.7 (Ar), 39.2 (=C*C*H_2_), 35.4 (=C*C*H_2_), 21.3 (CH_2_ *C*H_2_CH_2_); HRMS (ESI) *m*/*z* calcd for C_13_H_12_N_2_O_2_S (M + H)^+^ 261.0696, found 261.0692 (−1.6 ppm error). Lab book reference XW-002-126.

### 2-(2-(Methylamino)­benzo­[*d*]­thiazol-6-yl)­cyclopent-1-ene-1-carboxylic Acid **26b**

NaOH (70 mg, 1.75 mmol, 5.0 equiv) was added to a stirred solution of arylated ester **30b** (100 mg, 0.35 mmol, 1.0 equiv) in MeOH (1.5 mL) and H_2_O (1.5 mL) at rt. The resulting solution was stirred and heated at 50 °C for 24 h. After being allowed to cool to rt, NaOH (70 mg, 1.75 mmol, 5.0 equiv) and MeCN (1.5 mL) were added, and the resulting solution was stirred and heated at reflux for 2 h. After being allowed to cool to rt, H_2_O (50 mL) was added. The mixture was extracted with EtOAc (3 × 15 mL). Then, 2 M HCl_(aq)_ was added to the aqueous layer until pH ∼ 6 to give a solid precipitate. The solids were collected by filtration. The filtrate was extracted with EtOAc (3 × 20 mL). The combined organic extracts were washed with sat. brine (20 mL), dried (MgSO_4_), and evaporated under reduced pressure to give a solid. The two solid samples were combined to give carboxylic acid **26b** (74.0 mg, 78%, ≥ 95% pure by HPLC) as a white solid, mp 215–218 °C; IR (ATR) 3228 (NH), 2838, 2324, 1977, 1637 (C = O), 1584, 1547, 1470, 1405, 1332, 1236, 1128 cm^–1^; ^1^H NMR (400 MHz, DMSO-*d*_6_) δ 12.18 (br s, 1H, COOH), 8.01 (br s, 1H, NH), 7.70 (d, *J* = 1.5 Hz, 1H, Ar), 7.31 (d, *J* = 8.5 Hz, 1H, Ar), 7.26 (dd, *J* = 8.5, 1.5 Hz, 1H, Ar), 2.93 (s, 3H, NMe), 2.82 (t, *J* = 7.5 Hz, 2H, = CCH_2_), 2.71 (t, *J* = 7.5 Hz, 2H, = CCH_2_), 1.98 (tt, *J* = 7.5, 7.5 Hz, 2H, CH_2_C*H*_2_CH_2_); ^13^C NMR (100.6 MHz, DMSO-*d*_6_) δ 167.6 (*ipso*-Ar), 167.4 (C = O), 152.4 (=*C–*Ar), 149.8 (*ipso*-Ar), 129.8 (*ipso*-Ar), 129.2 (=*C*–CO), 128.5 (*ipso*-Ar), 125.9 (Ar), 120.4 (Ar), 116.9 (Ar), 40.0 (=C*C*H_2_), 35.5 (=C*C*H_2_), 30.5 (NMe), 21.3 (CH_2_ *C*H_2_CH_2_); HRMS (ESI) *m*/*z* calcd for C_14_H_14_N_2_O_2_S (M + H)^+^ 275.0853, found 275.0849 (−1.4 ppm error). Lab book reference XW-002-138.

### 2-(2-(Ethylamino)­benzo­[*d*]­thiazol-6-yl)­cyclopent-1-ene-1-carboxylic Acid **26c**

NaOH (66.1 mg, 1.65 mmol, 5.0 equiv) was added to a stirred solution of arylated ester **30c** (100 mg, 0.33 mmol, 1.0 equiv) in MeOH (1.5 mL) and H_2_O (1.5 mL) at rt. The resulting solution was stirred and heated at 50 °C for 24 h. After being allowed to cool to rt, NaOH (70 mg, 1.75 mmol, 5.0 equiv) and MeCN (1.5 mL) were added, and the resulting solution was stirred and heated at reflux for 2 h. After being allowed to cool to rt, H_2_O (50 mL) was added. The mixture was extracted with EtOAc (3 × 15 mL). Then, 2 M HCl_(aq)_ was added to the aqueous layer until pH ∼ 6 to give a solid precipitate. The solids were collected by filtration. The filtrate was extracted with EtOAc (3 × 20 mL). The combined organic extracts were washed with sat. brine (20 mL), dried (MgSO_4_), and evaporated under reduced pressure to give a solid. The two solid samples were combined to give carboxylic acid **26c** (83.3 mg, 87%, ≥ 95% pure by HPLC) as a white solid, mp 226–230 °C; IR (ATR) 3211 (NH), 2974, 1975, 1637 (C = O), 1579, 1468, 1430, 1307, 1256, 1128 cm^–1^; ^1^H NMR (400 MHz, DMSO-*d*_6_) δ 12.16 (br s, 1H, COOH), 8.06 (t, *J* = 5.5 Hz, 1H, NH), 7.69 (d, *J* = 2.0 Hz, 1H, Ar), 7.30 (d, *J* = 8.5 Hz, 1H, Ar), 7.25 (dd, *J* = 8.5, 2.0 Hz, 1H, Ar), 3.37 (m, 2H, N*CH*_2_Me), 2.82 (m, 2H, = CCH_2_), 2.77 (m, 2H, = CCH_2_), 1.88 (tt, *J* = 7.5, 7.5 Hz, 2H, CH_2_C*H*_2_CH_2_), 1.19 (t, *J* = 7.0 Hz, 1H, NCH_2_ *Me*); ^13^C NMR (100.6 MHz, DMSO-*d*_6_) δ 167.6 (*ipso*-Ar), 166.5 (C = O), 152.4 (=*C–*Ar), 149.8 (*ipso*-Ar), 129.7 (*ipso*-Ar), 129.1 (=*C*–CO), 128.4 (*ipso*-Ar), 125.8 (Ar), 120.4 (Ar), 116.9 (Ar), 40.0 (=C*C*H_2_), 38.8 (NC*H*_2_Me), 35.5 (=C*C*H_2_), 21.3 (CH_2_ *C*H_2_CH_2_), 14.4 (NCH_2_ *Me*); HRMS (ESI) *m*/*z* calcd for C_15_H_16_N_2_O_2_S (M + H)^+^ 289.1005, found 289.1005 (+0.1 ppm error). Lab book reference XW-002-139.

### 2-(2-Ureidobenzo­[*d*]­thiazol-6-yl)­cyclopent-1-ene-1-carboxylic Acid **26d**

Arylated ester **30a** (250 mg, 0.91 mmol, 1.0 equiv) and urea (247.3 mg, 9.10 mmol, 10.0 equiv) were added to a round-bottomed flask. The resulting mixture was stirred and heated at 170 °C for 1 h. After being allowed to cool to rt, the solid formed was recrystallized from H_2_O (10 mL), and the resulting solid was collected by filtration. EtOAc (20 mL) was added to the solid, and the resulting suspension was stirred and heated at reflux for 16 h. After being allowed to cool to rt, the suspension was washed with 2 M HCl_(aq)_ (3 × 20 mL) and H_2_O (3 × 20 mL). The organic suspension was collected and evaporated under reduced pressure to give a residue. NaOH (182 mg, 4.55 mmol, 5.0 equiv) was added to a stirred solution of the residue in MeOH (2.0 mL) and H_2_O (2.0 mL) at rt. The resulting solution was stirred and heated at 50 °C for 6 h. After being allowed to cool to rt, H_2_O (50 mL) was added. The mixture was extracted with EtOAc (3 × 20 mL). Then, 2 M HCl_(aq)_ was added to the aqueous layer until pH ∼ 5. The mixture was extracted with EtOAc (3 × 30 mL). The combined organic extracts were washed with sat. brine (30 mL), dried (MgSO_4_), and evaporated under reduced pressure to give carboxylic acid **26d** (116.2 mg, 40% over 2 steps, ≥ 95% pure by HPLC) as a pale-yellow solid, mp 183–186 °C; IR (ATR) 3345 (NH), 2940, 1710 (C = O), 1607, 1547, 1463, 1407, 1375, 1297, 1224 cm^–1^; ^1^H NMR (400 MHz, DMSO-*d*_6_) δ 12.14 (br s, 1H, COOH), 10.76 (br s, 1H, CONH), 7.87 (d, *J* = 2.0 Hz, 1H, Ar), 7.54 (d, *J* = 8.5 Hz, 1H, Ar), 7.36 (dd, *J* = 8.5, 2.0 Hz, 1H, Ar), 6.61 (br s, 2H, CONH_2_), 2.85 (t, *J* = 7.5 Hz, 2H, = CCH_2_), 2.73 (t, *J* = 7.5 Hz, 2H, = CCH_2_), 1.92 (tt, *J* = 7.5, 7.5 Hz, 2H, CH_2_C*H*_2_CH_2_); ^13^C NMR (100.6 MHz, DMSO-*d*_6_) δ 167.3 (*ipso*-Ar), 160.4 (C = O), 154.5 (=*C–*Ar), 150.4 (*ipso*-Ar), 148.8 (*ipso*-Ar), 131.1 (*ipso*-Ar), 131.1 (C = O), 129.1 (=*C*–CO), 126.0 (Ar), 120.6 (Ar), 118.7 (Ar), 39.4 (=C*C*H_2_), 35.4 (=C*C*H_2_), 21.4 (CH_2_ *C*H_2_CH_2_); HRMS (APCI) *m*/*z* calcd for C_14_H_13_N_3_O_3_S (M + H)^+^ 304.0754, found 304.0750 (+1.3 ppm error). Lab book reference XW-002-148.

### 2-(2-Aminobenzo­[*d*]­thiazol-6-yl)­cyclopentane-1-carboxylic Acid *trans*-**27**

Trifluoromethanesulfonic anhydride (0.129 mL, 0.77 mmol, 1.0 equiv) was added to a stirred solution of *trans*-**22a** (200 mg, 0.77 mmol, 1.0 equiv) in dry CH_2_Cl_2_ (3 mL) at −78 °C under N_2_. The resulting solution was stirred at −78 °C under N_2_ for 30 min. Then, triphenylphosphine (222 mg, 0.85 mmol, 1.1 equiv) was added, and the resulting mixture was degassed and backfilled with N_2_ three times. The resulting mixture was stirred at −78 °C for 30 min. Triethylamine (0.107 mL, 0.77 mmol, 1.0 equiv) was added at −78 °C, and the resulting mixture was allowed to warm slowly to rt and stirred for 30 min at rt. H_2_O (3 mL) and CH_2_Cl_2_ (20 mL) were added. The two layers were separated, and the organic layer was washed with H_2_O (3 × 15 mL) and sat. brine (20 mL), dried (MgSO_4_), and evaporated under reduced pressure to give a residue. NaN_3_ (62.4 mg, 0.96 mmol, 1.25 equiv) was added to a stirred solution of the residue in DMSO (0.62 mL). The resulting mixture was stirred and heated at 120 °C for 16 h. One M HCl_(aq)_ (1.2 mL) was added, and the resulting mixture was stirred and heated at 120 °C for 2 h. After being allowed to cool to rt, 1 M HCl_(aq)_ (20 mL) was added. The mixture was extracted with EtOAc (3 × 10 mL). The aqueous phase was basified with sat. Na_2_CO_3(aq)_ until pH = 7 ∼ 8, and then extracted with EtOAc (6 × 20 mL). The combined organics were washed with sat. brine (30 mL), dried (MgSO_4_), and evaporated under reduced pressure to give the crude product. The crude product was triturated using CH_2_Cl_2_ (2 mL) and, after standing at rt, the solid was collected by filtration to give impure product. Further purification by prep-TLC with 20:1 CH_2_Cl_2_-MeOH as eluent gave carboxylic acid *trans*-**27** (12 mg, 6%, ≥ 95% pure by ^1^H NMR) as a yellow solid, mp 115–118 °C; IR (ATR) 3357 (NH), 3273 (NH), 2945, 2870, 2539, 1698 (C = O), 1612, 1489, 1448, 1412, 1275, 1198, 1155 cm^–1^; ^1^H NMR (400 MHz, CD_3_OD) δ 7.05–7.03 (m, 2H, Ar), 6.70 (d, *J* = 7.5 Hz, 1H, Ar), 3.12–3.05 (m, 1H, CHAr), 2.64–2.57 (m, 1H, CHCO), 2.16–1.97 (m, 2H, CH), 1.93–1.74 (m, 3H, CH), 1.63–1.54 (m, 1H, CH); ^13^C NMR (100.6 MHz, CD_3_OD) δ 179.9 (C = O), 149.1 (*ipso*-Ar), 135.8 (Ar), 134.8 (*ipso*-Ar), 134.7 (*ipso*-Ar), 131.6 (Ar), 119.8 (*ipso*-Ar), 116.7 (Ar), 120.4 (Ar), 116.9 (Ar), 53.6 (CH), 50.4 (CH), 35.9 (CH_2_), 31.8­(CH_2_), 25.8 (CH_2_); Attempted HRMS (ESI) was unsuccessful. Lab book reference XW-002-082, XW-02-087.

### Ligand Efficiency Calculations

Ligand efficiency values^26^ were calculated from the IC_50_ value and the number of heavy (nonhydrogen) atoms in the ligand using this web-based calculator.^67^

### Initial Molecular Modeling of Mac1 Inhibitors. Molecular Modeling

Molecular modeling experiments were carried out using Schrodinger’s Maestro v.13.0 (Schrodinger, Inc.: Maestro v13.0, 2021-4).

### Protein and Ligand Preparation

The three-dimensional coordinates, protonation states, and tautomeric forms of the ligands were generated using the LigPrep module in Maestro, with Epik employed to model the compounds at a pH of 7.4 ± 1.0.^68,69^ For each ligand, five representative conformers were produced using ConfGen,^70^ and these conformers were subsequently utilized in the docking studies. The Protein Preparation Wizard workflow implemented in Maestro^69^ was used to assign bond orders and protonation states at pH 7.0 ± 2.0 and to add hydrogen atoms and fill missing residues to three published X-ray crystal structures of Mac1 in complex with fragment *cis*-**11** (PDB ID: 5S3T).^7^ Hydrogen atoms were then minimized using the OPLS-4 force field.^71^ Water molecules were removed for the docking calculations.

### Molecular Docking

Ligand docking was performed with Glide.^72^ A docking grid was generated using the prepared crystal structure PDB ID: 5S3T with the cocrystallized ligand selected as the center of the grid. For the docking run, the flexible docking standard precision (SP) option was selected with a core restraint on the position of the cyclopentanoic acid and an RMSD tolerance of 0.2 Å from the cocrystallized coordinates. Aromatic −CH groups were included as the hydrogen-bond donors. The rest of the parameters were kept as default. Glide was requested to return 2 poses per ligand. Strain energy contributions and corrected-docking scores were calculated for each pose with the tool available in Maestro. Docked poses with ligand strain below 5 kcal/mol were retained for further analysis. The docking protocol was validated by docking the cocrystallized ligand into 5S3T. The best heavy atom root-mean-square deviation (RMSD) of the cocrystallized molecule vs the docked pose was 0.62 Å. The docked poses were further refined by Molecular Mechanics Generalized Born Surface Area (MM-GBSA) in Prime^73^ using the default solvation model and allowing the residues 5 Å around the ligand to relax. Poses with the lowest MM-GBSA scoring were visually inspected. The structures of the priority 1 and priority 2 compounds and the docked poses can be found in the SI.

### HTRF Assay for Mac1 Inhibitors

Inhibition of SARS-CoV-2 Nsp3 macrodomain Mac1 was assessed by the displacement of an ADP-ribose-conjugated biotin peptide from His_6_-tagged protein using a HTRF-technology-based screening assay which was performed as previously described by Schuller et al.^7^ ADP-ribose was tested as reference with a top concentration of 40 μM, while Mac1 inhibitors were tested at a concentration of 200–2000 μM in duplicate measurements with an 8 point 1:1 dilution series in duplicate measurements. The inhibitory activity of **26a**–**c** was additionally confirmed with an 11-point curve measurement with 1:1 dilution series. Compounds were dispensed into ProxiPlate-384 Plus (PerkinElmer) assay plates using an Echo 525 liquid handler (Labcyte). Binding assays were conducted in a final volume of 16 μL with 12.5 nM SARS-CoV-2 NSP3 macrodomain Mac1, 400 nM peptide ARTK­(Bio)­QTARK­(Aoa-RADP)­S, 1:20000 Anti-His_6_-Eu^3+^ cryptate (HTRF donor, PerkinElmer) and 1:125 Streptavidin-XL665 (HTRF acceptor, PerkinElmer) in assay buffer (25 mM HEPES pH 7.0, 20 mM NaCl, 0.05% bovine serum albumin and 0.05% Tween-20). Macrodomain protein and peptide were first dispensed and incubated for 30 min at room temperature. This was followed by the addition of the HTRF reagents and incubation at room temperature for 1 h. Fluorescence was measured using a PHERAstar microplate reader (BMG) using the HTRF module with a dual-emission protocol (A = excitation of 320 nm, emission of 665 nm, and B = excitation of 320 nm, emission of 620 nm). Raw data were processed to give an HTRF ratio (channel A/B × 10,000), which was used to generate IC_50_ curves. IC_50_ values were determined by nonlinear regression using GraphPad Prism v.10.5 (GraphPad Software, CA, USA). The results are shown in the IC_50_ curves in the SI.

### X-ray Crystallography of Mac1-Inhibitor Cocrystals

SARS-CoV-2 NSP3 macrodomain Mac1 was crystallized in either space group *P*4_3_ or *P*12_1_1 as previously described.^7,32^ To obtain liganded crystal structures of Mac1, compounds (100 mM) were soaked into drops containing Mac1 crystals at 10% (v/v) DMSO using acoustic dispensing^74^ with an Echo 650 liquid handler (Labcyte). After incubation for 1 h at 20 °C, crystals were harvested and cryo-cooled in liquid nitrogen. Data were collected at the I04-1 beamline (Diamond Light Source, UK) at 100 K and automatically processed with Diamond Light Source’s autoprocessing pipelines with the default settings. Data analysis was carried out using XChemExplorer.^75^ Electron density maps were generated with DIMPLE,^76^ ligand restraints were calculated with GRADE^77^ and ligand-binding events were identified using PanDDA2.^78^ Ligands were modeled into PanDDA2-calculated event maps using its autobuild function or manually using Coot,^79^ and structures were refined using BUSTER.^80^ Coordinates, structure factors, and PanDDA2 event maps for the Mac1 structures discussed in this paper are deposited in the Protein Data Bank (group deposition G_1002351). Data collection and refinement statistics are summarized in Table S7.

### Molecular Modeling of Amino Benzothiazole Mac1 Inhibitors: Molecular Docking

The results of this study are shown in Figure 1. Molecular docking experiments were carried out using the MOE.^81^ The binding pose for amino benzothiazole **26a** was generated by starting with the X-ray crystal structure of the benzothiazole cyclopentenyl acid **20a**-Mac1 complex (PDB: 7IJT), adding in the amino group, and then minimizing the energy. The binding pose for amino benzothiazole *trans*-**27a** was generated by starting with the X-ray crystal structure of the benzothiazole cyclopentenyl acid *trans*-**17g**-Mac1 complex (PDB: 7IJS), adding in the amino group, and then minimizing the energy. The two binding poses are listed in Figure 5.

### *In Vitro* De-ADP-Ribosylation Activity Assay Using AMP-Glo Luminescence Detection

The hydrolytic activity of selected enzymes was assessed using the well-established AMP-Glo assay^56,57^ with a chemically synthesized glutamate-ADPr substrate, as described previously.^58^ Hydrolases are purified using standard protocols as described previously.^7,82^ Assays were performed with 10 μM glutamate-ADPr and either 62.5 nM Mac1 or 500 nM NudT16 (Nudix hydrolase 16),^83^ ARH1 (ADP-ribosylhydrolase 1), or ARH3 (ADP-ribosylhydrolase 3) in reaction buffer containing 50 mM Tris-HCl [pH 7.5], 200 mM NaCl, 10 mM MgCl_2_, and 1 mM Dithiothreitol (DTT), together with 0.2 μM NudT5, and incubated for 30 min at 30 ^◦^C. For conditions with inhibitory compounds, Mac1 was preincubated with compounds **26a**–**c** and *trans*-**17g** (or an equivalent volume of DMSO as control) at room temperature for 20 min prior to assay initiation.

### Calculated Properties for Compounds with IC_50_ Values <100 (Tables 5 and 6)

Properties were calculated using bespoke machine learning models in AstraZeneca’s Predictive Insight Platform.^62^ AZLogD and solubility were calculated using a multitask Graph Convolutional Neural Network (GCNN) model that predicts in parallel AZlogD, ePSA (exposed polar surface area expressed in Å^2^), ChromlogD and Solubility DD (dried DMSO solubility). clogP is the octanol/water partition coefficient predicted using Daylight/Biobyte software.^84^ TPSA is calculated using Oeselma, a program for generating most common 2D molecular descriptors. The intrinsic clearance (CLint) in hepatocytes (HH) and human liver microsomes (HLM) model is a multitask model based on GCNNs.

## Supplementary Material





## Notes

LCMS data traces; additional information on proof of stereochemistry; poses for the initial molecular modeling of Mac1 inhibitors; IC_50_ curves for the HTRF assay of Mac1 inhibitors; data collection and refinement statistics for X-ray crystallography of Mac1-inhibitor cocrystals; water map modeling of Mac1 inhibitors; and ^1^H/^13^C NMR spectra of all compounds (PDF)
Molecular formula strings (CSV)

## ○

Max Planck Institute of Biochemistry, Am Klopferspitz 18, 82152 Planegg/Martinsried, Germany

## Abbreviations Used

ADPr: adenosine diphosphate ribose
AMP: adenosine monophosphate
ARH1: ADP-ribosylhydrolase 1
ARH3: ADP-ribosylhydrolase 3
Cbz: carbobenzyloxy
CCDC: Cambridge Crystallographic Data Centre
COVID-19: coronavirus disease 2019
3-D: three-dimensional
DMA: *N,N*-dimethylacetamide
dme: dimethoxy ethane
DMSO: dimethyl sulfoxide
dppf: 1,1′-bis­(diphenylphosphino)­ferrocene
dtbbpy: 4,4′-bis­(1,1-dimethylethyl)-2,2′-bipyridine-N1,N1′
DTT: Dithiothreitol
FRET: fluorescence resonance energy transfer
GCNN: Graph Convolutional Neural Network
HH CLint: human hepatocyte intrinsic clearance
HLM CLint: human liver microsomes intrinsic clearance
HTS: high-throughput screening
[Ir­(dFCF_3_ppy)_2_(dtbbpy)]­PF_6_: **[**4,4′-bis­(1,1-dimethylethyl)-2,2′-bipyridine-N1,N1′]­bis­[3,5-difluoro-2-[5-(trifluoromethyl)-2-pyridinyl-N]­phenyl-C]­iridium­(III) hexafluorophosphate
LE: ligand efficiency
LED: light emitting diode
M^Pro^: main protease
Nsp3: nonstructural protein 3
Nsp13: nonstructural protein 13 helicase
Nsp14: nonstructural protein 14 exonuclease/methyltransferase
Nsp15: nonstructural protein 15 endoribonuclease
NudT16: Nudix hydrolase 16
PDB: Protein Data Bank
pin: pinacolate
RMSD: root-mean-square deviation
SARS CoV-2: severe acute respiratory syndrome coronavirus 2
SD: standard deviation
THF: tetrahydrofuran
Tf: trifluoromethanesulfonyl
TPSA: topological polar surface area
