# Supplementary material for: X‑ray Crystallography-Guided Design and Synthesis of Cyclopentyl Heteroaryl Carboxylic Acid-Based Inhibitors of the SARS-CoV‑2 Nsp3 Macrodomain (Mac1)
Source: J Med Chem. 2026 Jul 13;69(14):16570–600. doi: 10.1021/acs.jmedchem.6c00236 (PMC13403239; doi:10.1021/acs.jmedchem.6c00236)

Supporting Information

X-ray Crystallography-Guided Design and Synthesis of Cyclopentyl Heteroaryl Carboxylic Acid-based Inhibitors of the SARS-CoV-2 Nsp3 Macrodomain (Mac1)

Xinyu Wang,[a] William T. W. Butler,[a] James R. Donald,[a] Yuran Wang,[a] Alice L. Shaw,[a] Marion Schuller,[b,†] Daren Fearon,[c,d] Jasmin C. Aschenbrenner,[c,d] Peter G. Marples,[c,d] Grant Watt,[c,d] Yang Lu,[b] Simon C. C. Lucas,[e] Silvia Bonomo,[e] Jennifer E. Nelson,[e] Ivan Ahel,*[b] Frank von Delft*[c,d,f,g] and Peter O'Brien*[a]

[a] Department of Chemistry, University of York, York, YO10 5DD, UK.
[b] Sir William Dunn School of Pathology, University of Oxford, South Parks Road, Oxford, OX1 3RE, UK.
[c] Diamond Light Source Ltd, Harwell Science and Innovation Campus, Didcot, Oxfordshire, OX11 0DE, UK.
[d] Research Complex at Harwell, Harwell Science and Innovation Campus, Didcot, Oxfordshire, OX11 0DE, UK.
[e] Hit Discovery, Discovery Sciences, R&D, AstraZeneca, 1 Francis Crick Ave, Cambridge, CB2 0AA, UK.
[f] Centre for Medicines Discovery, University of Oxford, NDM Research Building, Oxford, Oxfordshire, OX3 7FZ, UK.
[g] Department of Biochemistry, University of Johannesburg, Johannesburg, 2092, South Africa.

† Current address: Max Planck Institute of Biochemistry, Am Klopferspitz 18, 82152 Planegg/ Martinsried, Germany.

peter.obrien@york.ac.uk, ivan.ahel@path.ox.ac.uk, frank.von-delft@diamond.ac.uk

Contents of Supporting Information:

**1**	**LCMS Data**	**S2**
**2**	**Proof of Stereochemistry**	**S20**
**3**	**Initial Molecular Modelling of Mac1 Inhibitors**	**S24**
**4**	**HTRF Assay of Mac1 Inhibitors**	**S33**
**5**	**X-ray Crystallography of Mac1-inhibitor Co-crystals**	**S37**
**6**	**WaterMap Modelling of Mac1 Inhibitors**	**S43**
**7**	***In vitro* de-ADP-ribosylation activity assay using AMP-Glo luminescence detection**	**S45**
**8**	**^{1}H and ^{13}C NMR Spectra**	**S46**

1 LCMS Data

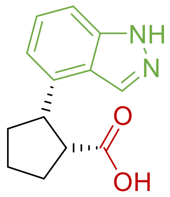

cis-**17c**

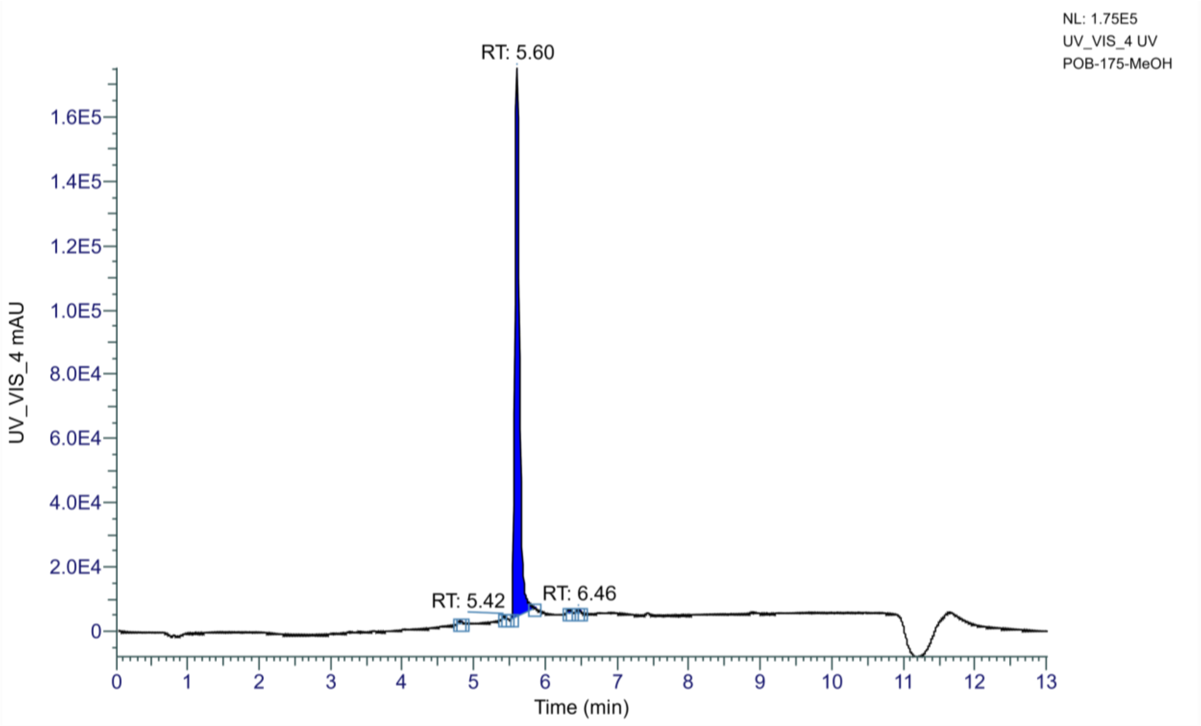

RT (Min)	% Area	Integration Method
4.81	0.22	Automated - ICIS
5.42	0.19	Automated - ICIS
5.6	98.91	Automated - ICIS
6.34	0.31	Automated - ICIS
6.46	0.37	Automated - ICIS

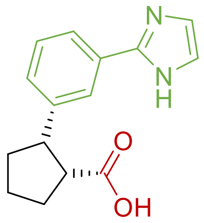

cis-**17f**

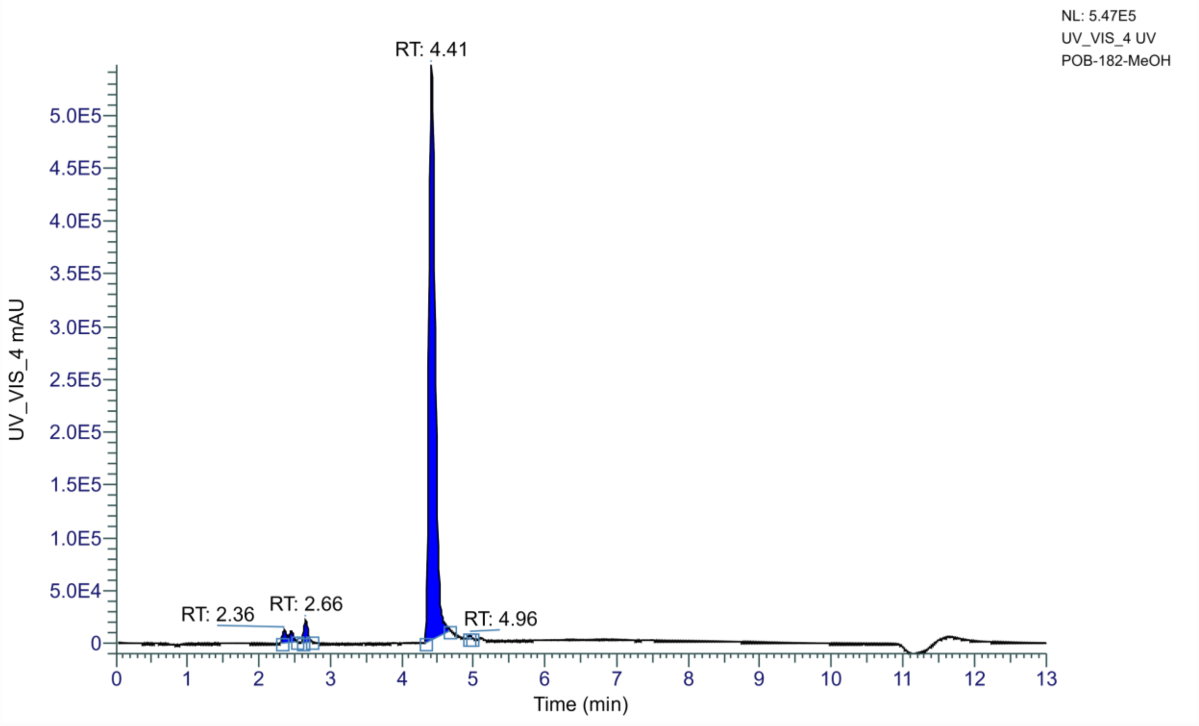

RT (Min)	% Area	Integration Method
2.36	2.71	Automated - ICIS
2.66	2.39	Automated - ICIS
4.41	94.84	Automated - ICIS
4.96	0.06	Automated - ICIS

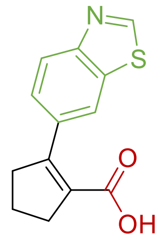

20a

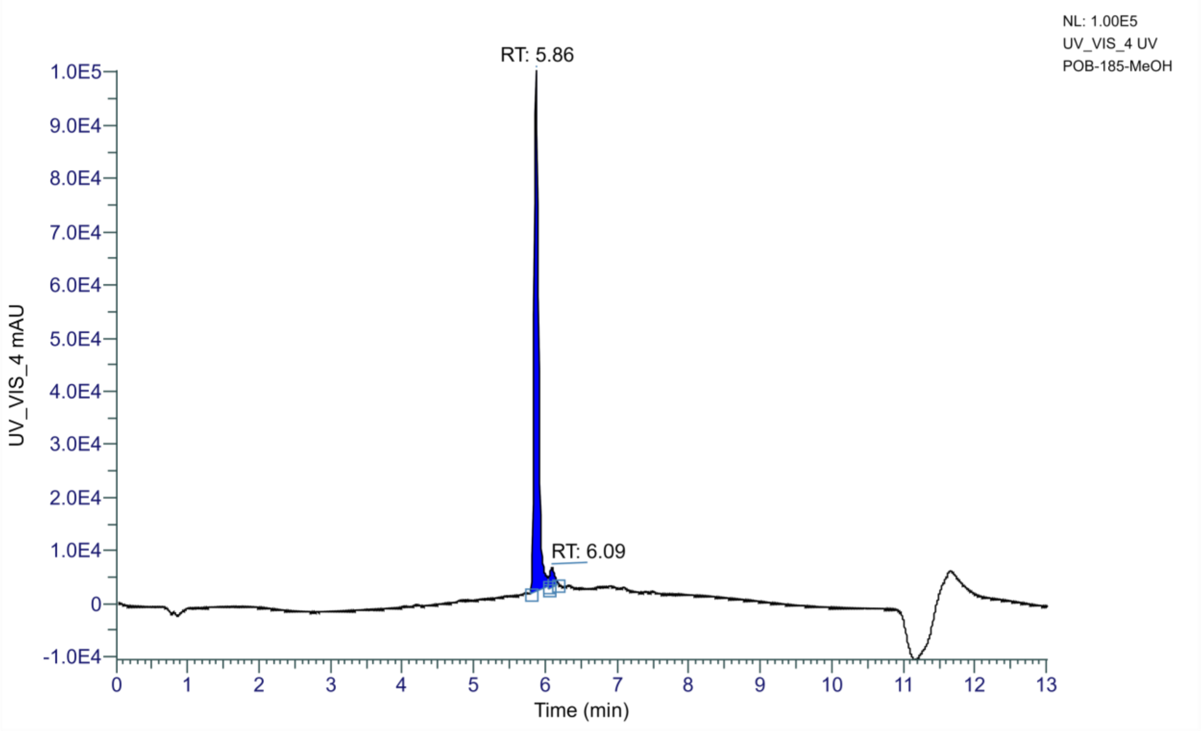

RT (Min)	% Area	Integration Method
5.86	96.07	Automated - ICIS
6.09	3.93	Automated - ICIS

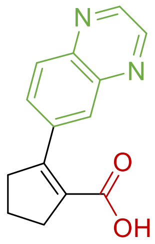

20b

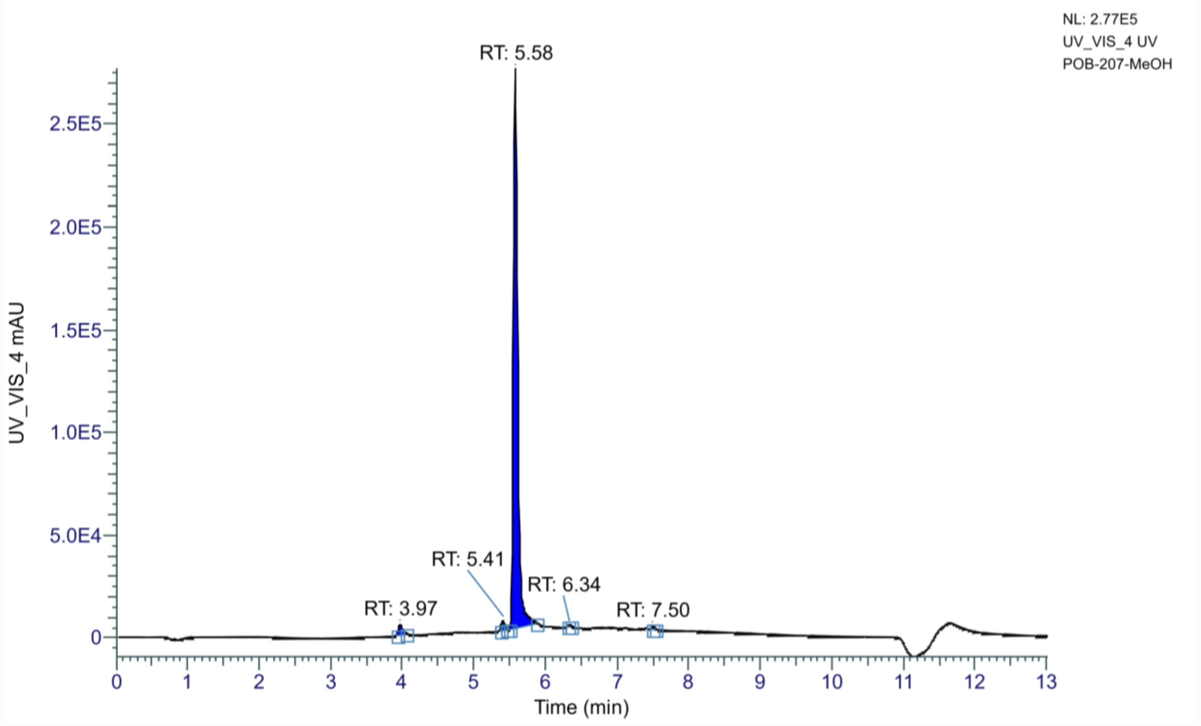

RT (Min)	% Area	Integration Method
3.97	1.73	Automated - ICIS
5.41	1.28	Automated - ICIS
5.58	96.71	Automated - ICIS
6.34	0.11	Automated - ICIS
7.5	0.17	Automated - ICIS

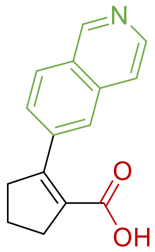

20c

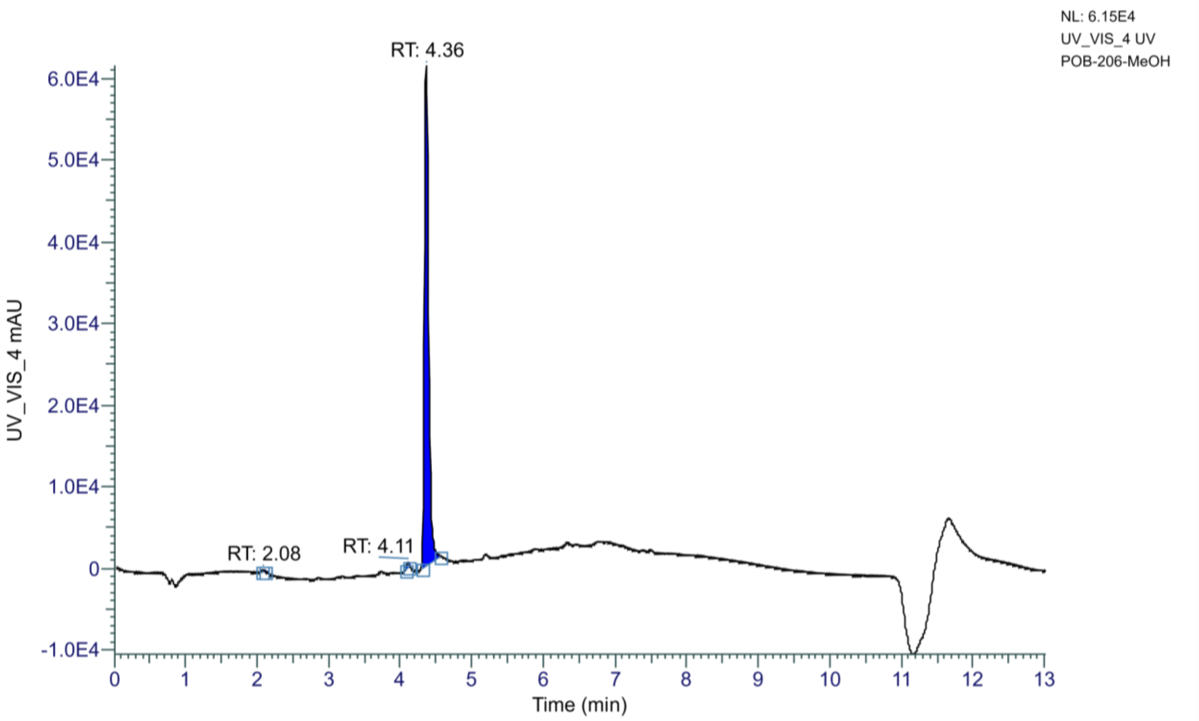

RT (Min)	% Area	Integration Method
2.08	0.17	Automated - ICIS
4.11	0.54	Automated - ICIS
4.36	99.28	Automated - ICIS

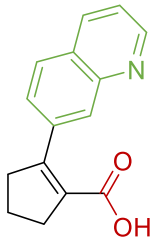

20d

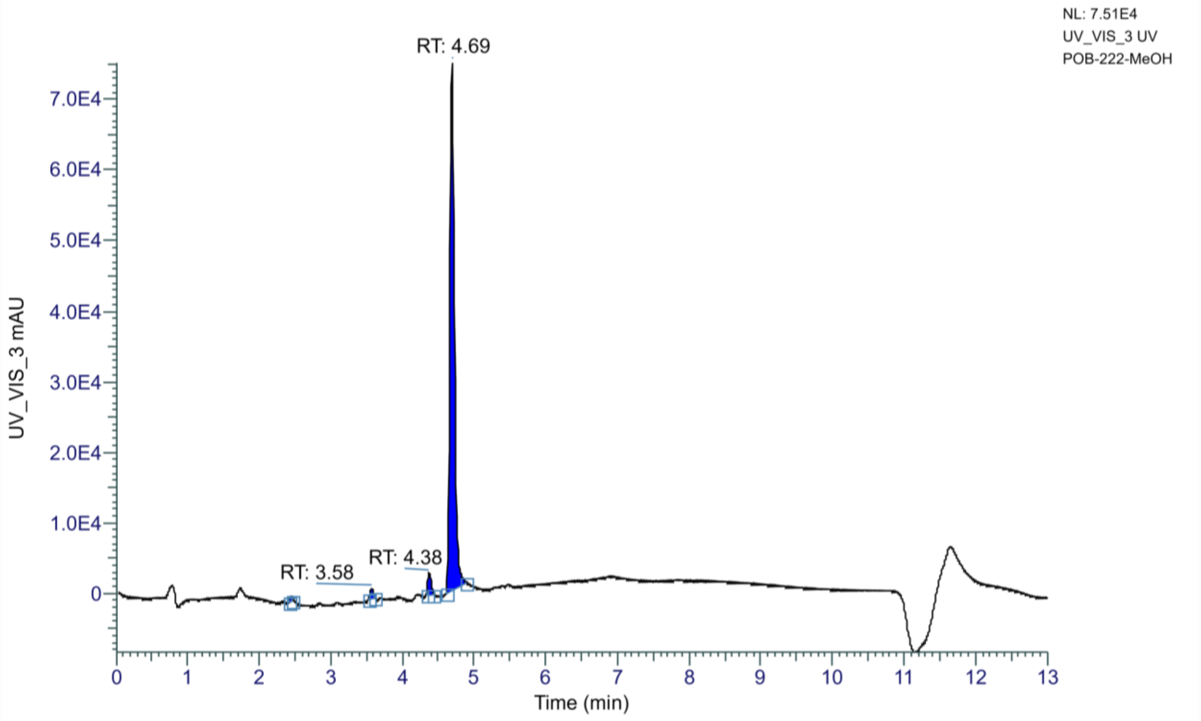

RT (Min)	% Area	Integration Method
2.45	0.37	Automated - ICIS
3.58	1.31	Automated - ICIS
4.38	3.23	Automated - ICIS
4.69	95.1	Automated - ICIS

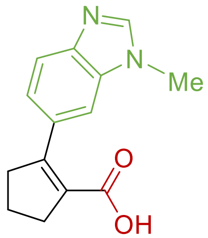

20g

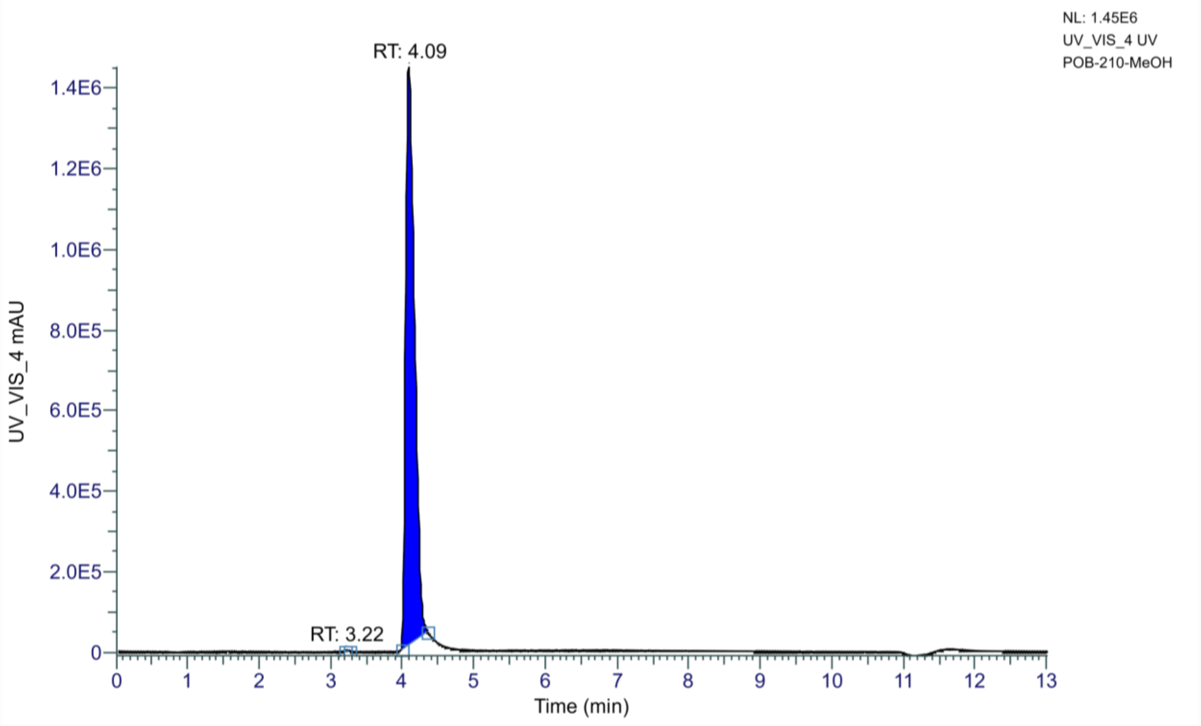

RT (Min)	% Area	Integration Method
3.22	0.07	Automated - ICIS
4.09	99.93	Automated - ICIS

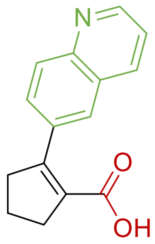

20h

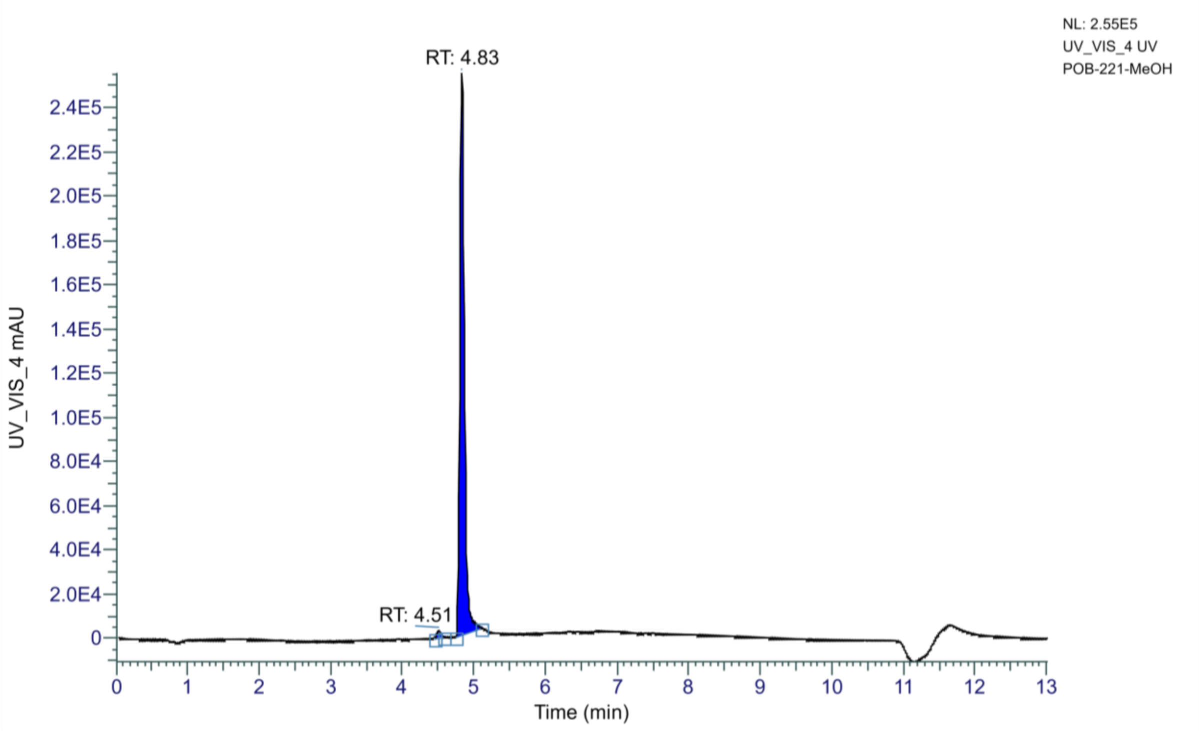

RT (Min)	% Area	Integration Method
4.51	1.14	Automated - ICIS
4.83	98.86	Automated - ICIS

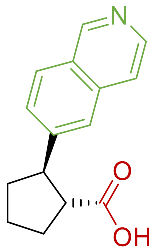

trans-**17a**

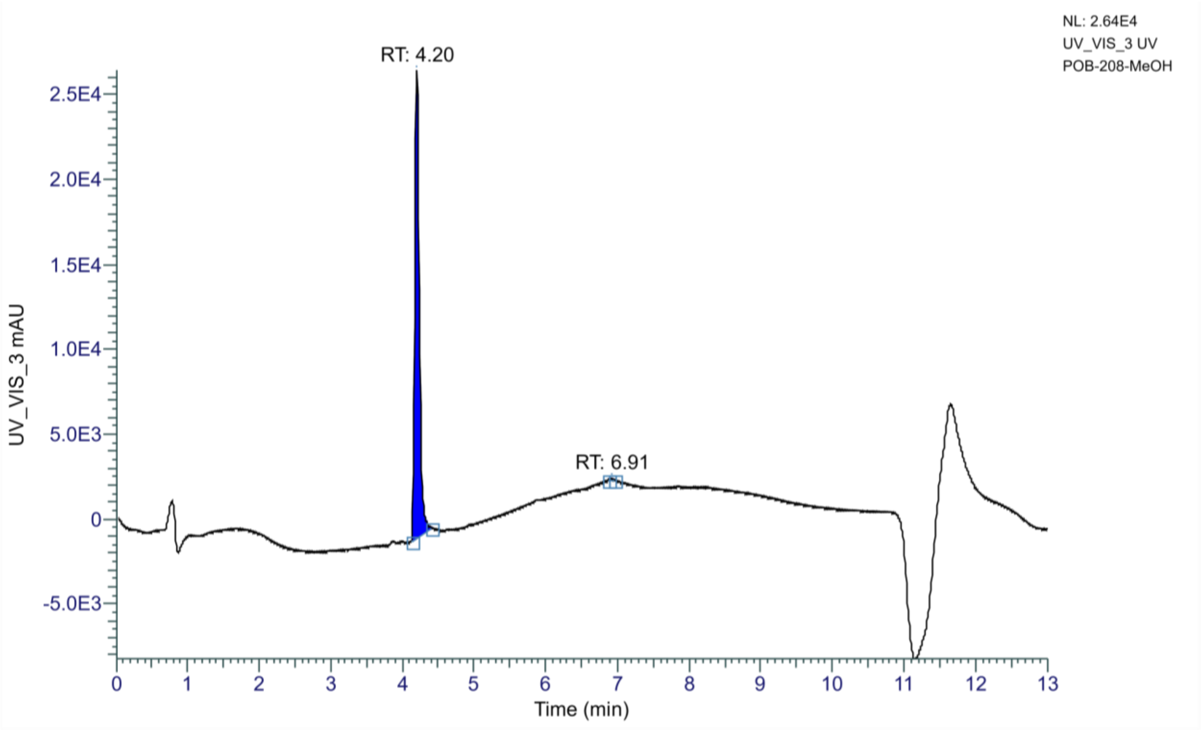

RT (Min)	% Area	Integration Method
4.2	99.67	Automated - ICIS
6.91	0.33	Automated - ICIS

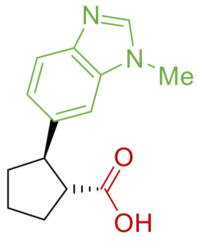

trans-**17b**

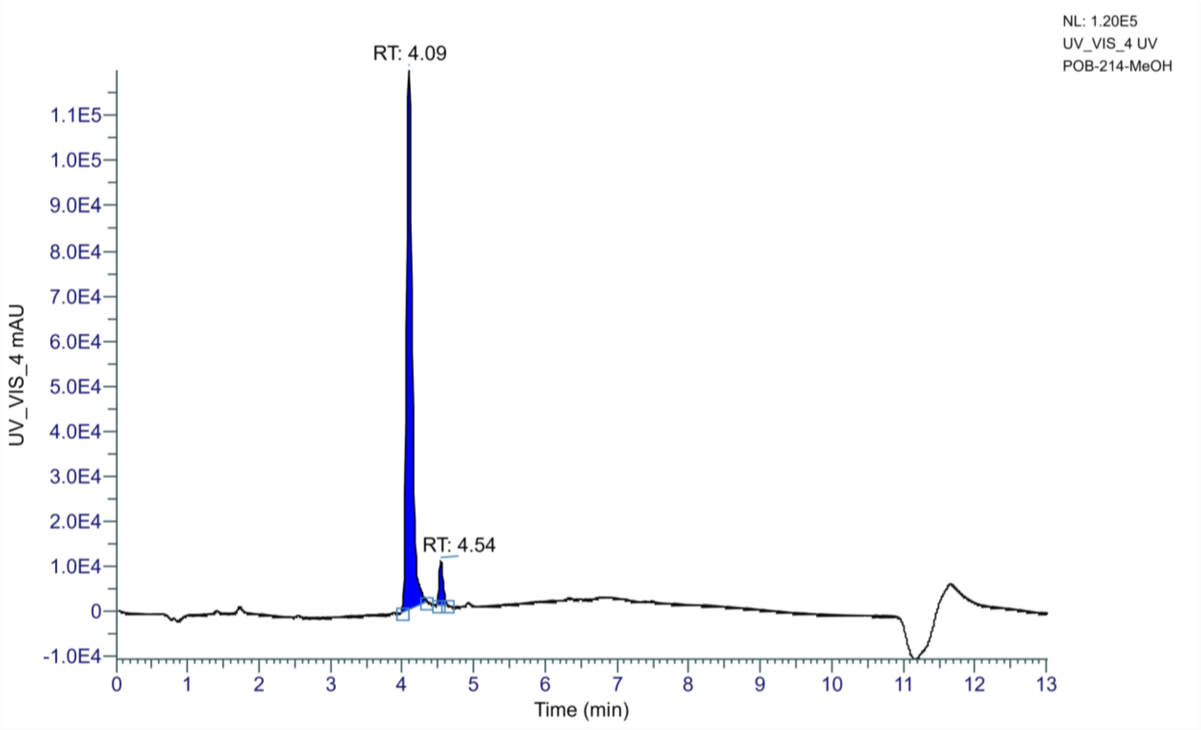

RT (Min)	% Area	Integration Method
4.09	94.69	Automated - ICIS
4.54	5.31	Automated - ICIS

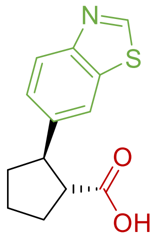

trans-**17g**

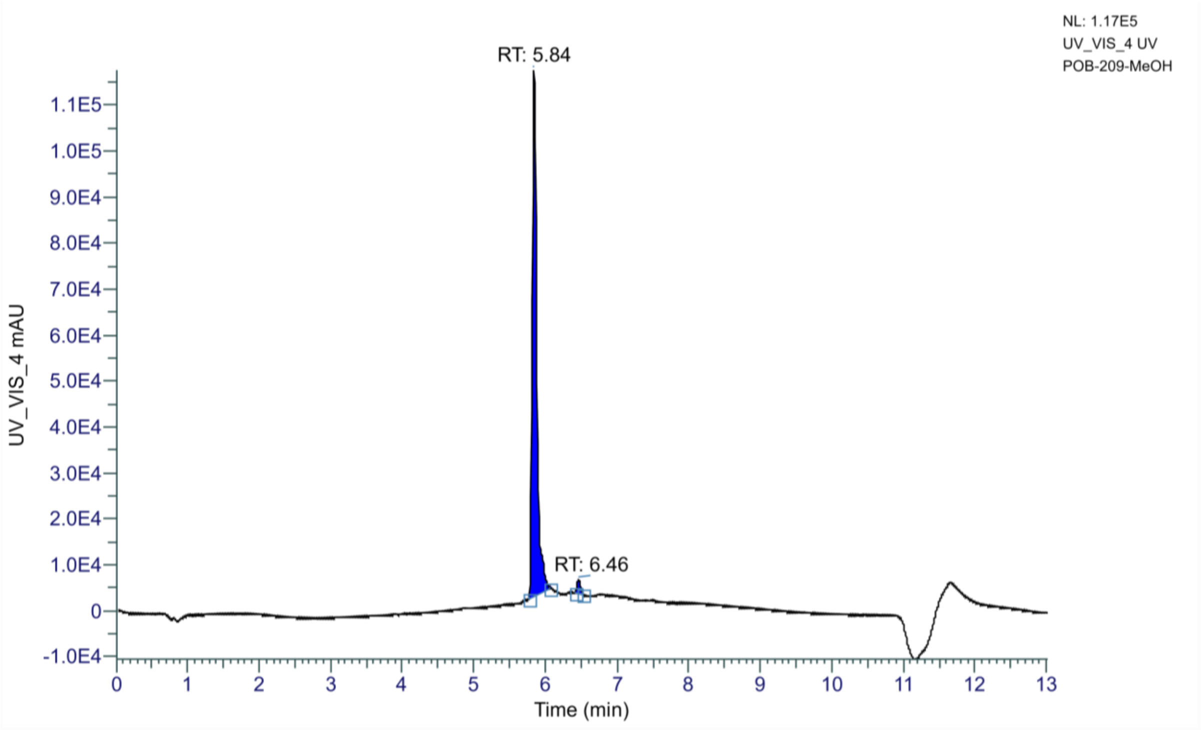

RT (Min)	% Area	Integration Method
5.84	98.11	Automated - ICIS
6.46	1.89	Automated - ICIS

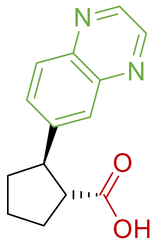

trans-**17h**

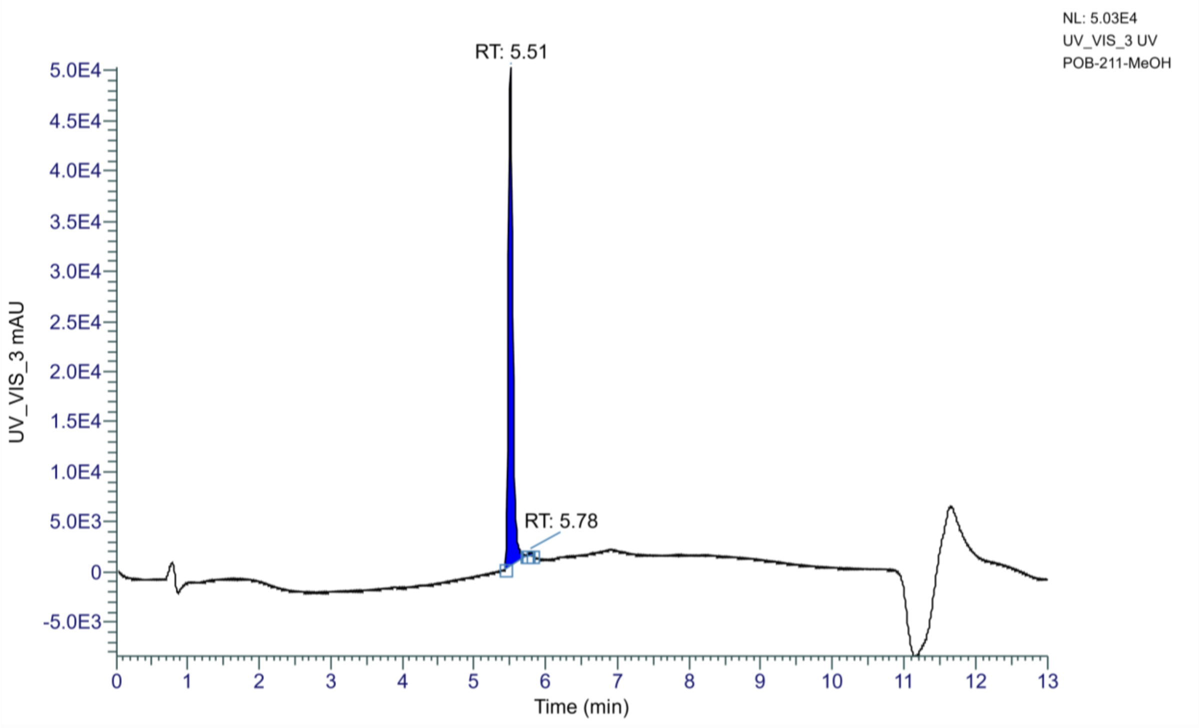

RT (Min)	% Area	Integration Method
5.51	99.54	Automated - ICIS
5.78	0.46	Automated - ICIS

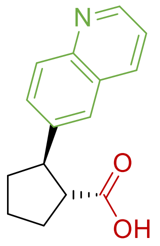

trans-**17i**

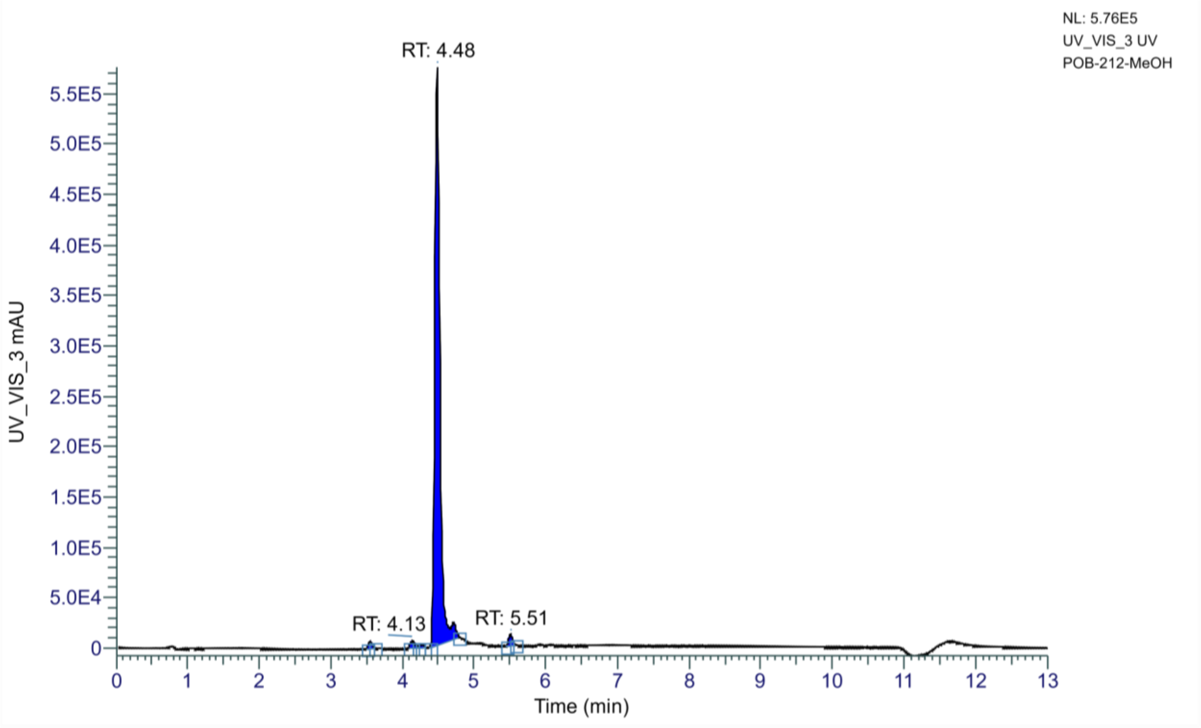

RT (Min)	% Area	Integration Method
3.55	0.85	Automated - ICIS
4.13	1.03	Automated - ICIS
4.26	0.45	Automated - ICIS
4.48	96.27	Automated - ICIS
5.09	0.01	Automated - ICIS
5.51	1.39	Automated - ICIS

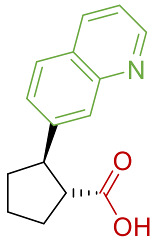

trans-**17j**

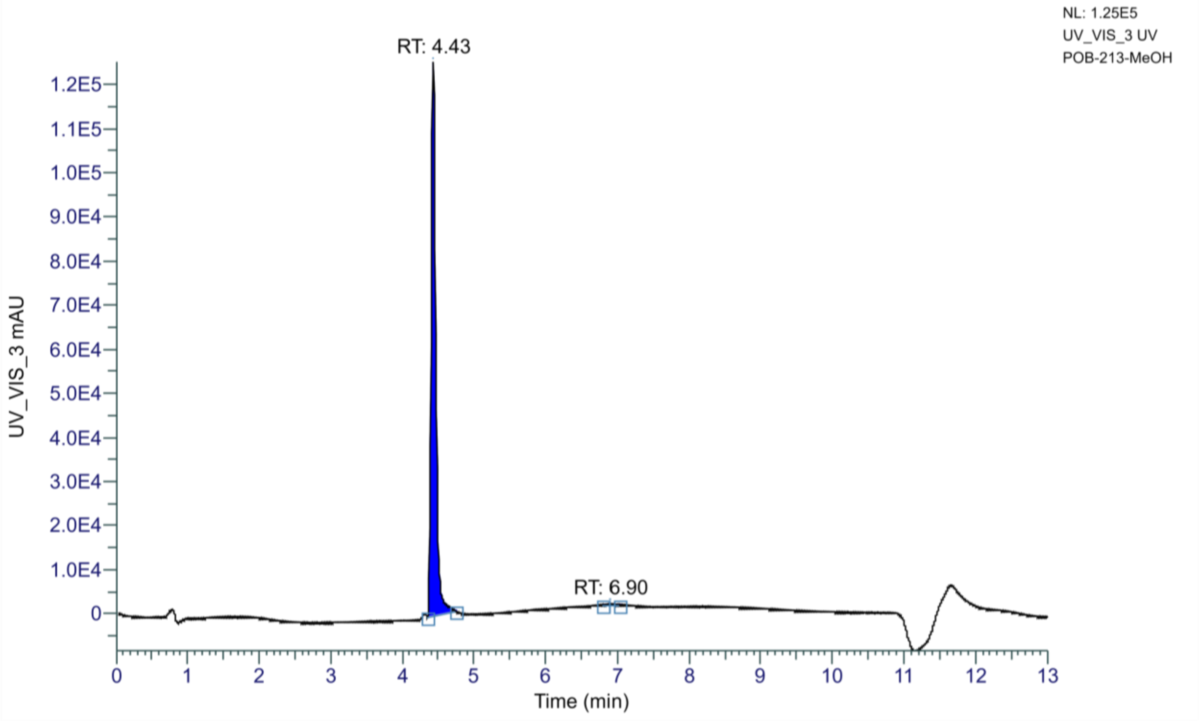

RT (Min)	% Area	Integration Method
4.43	99.72	Automated - ICIS
6.9	0.28	Automated - ICIS

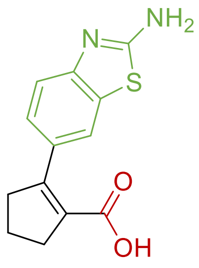

26a

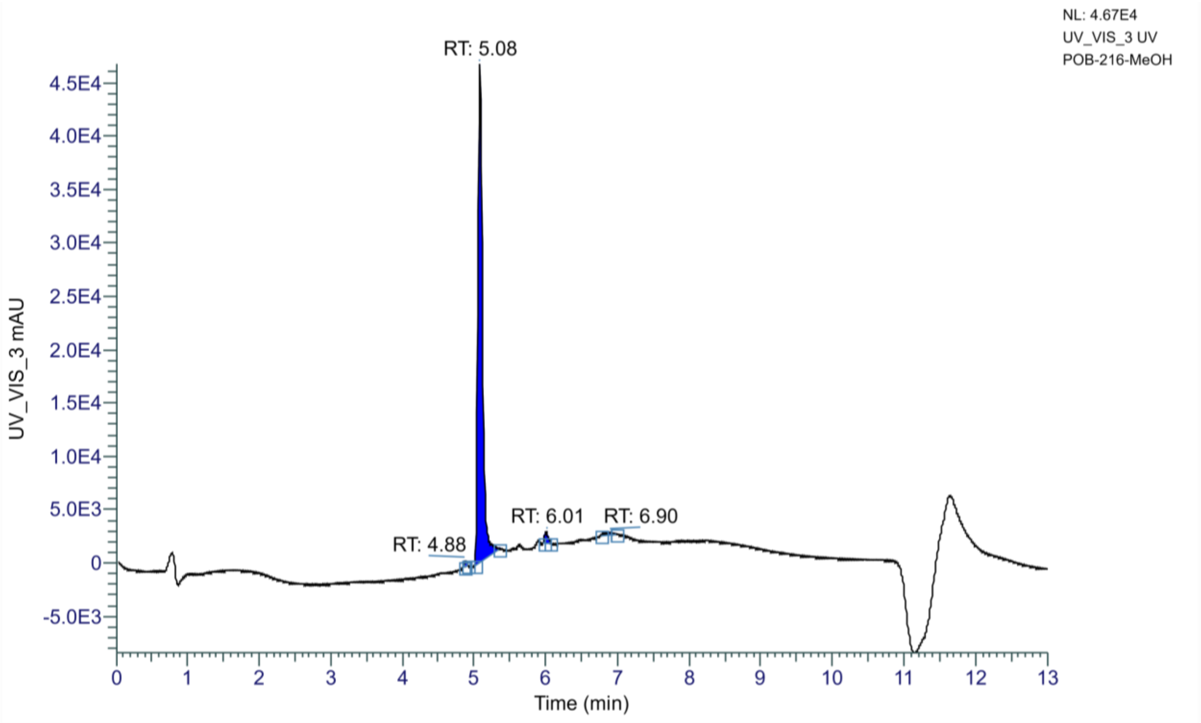

RT (Min)	% Area	Integration Method
4.88	0.42	Automated - ICIS
5.08	96.99	Automated - ICIS
6.01	1.57	Automated - ICIS
6.9	1.02	Automated - ICIS

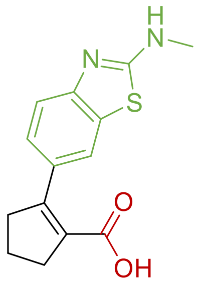

26b

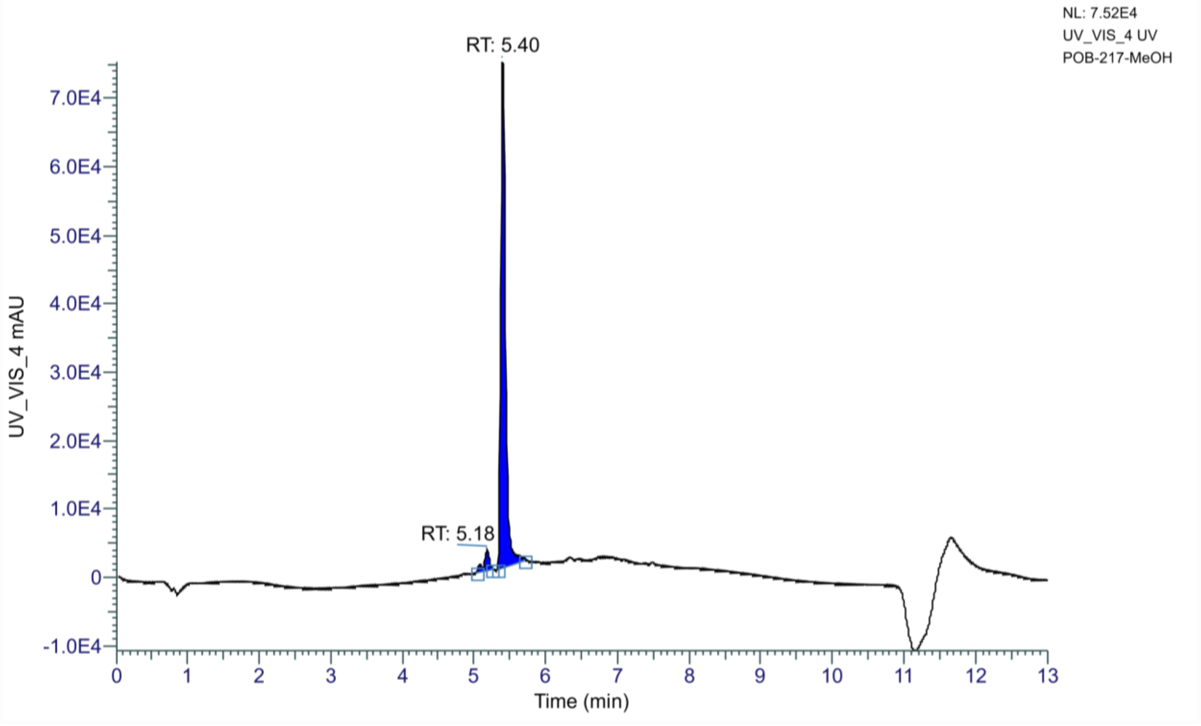

RT (Min)	% Area	Integration Method
5.18	4.44	Automated - ICIS
5.4	95.56	Automated - ICIS

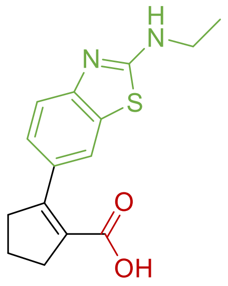

26c

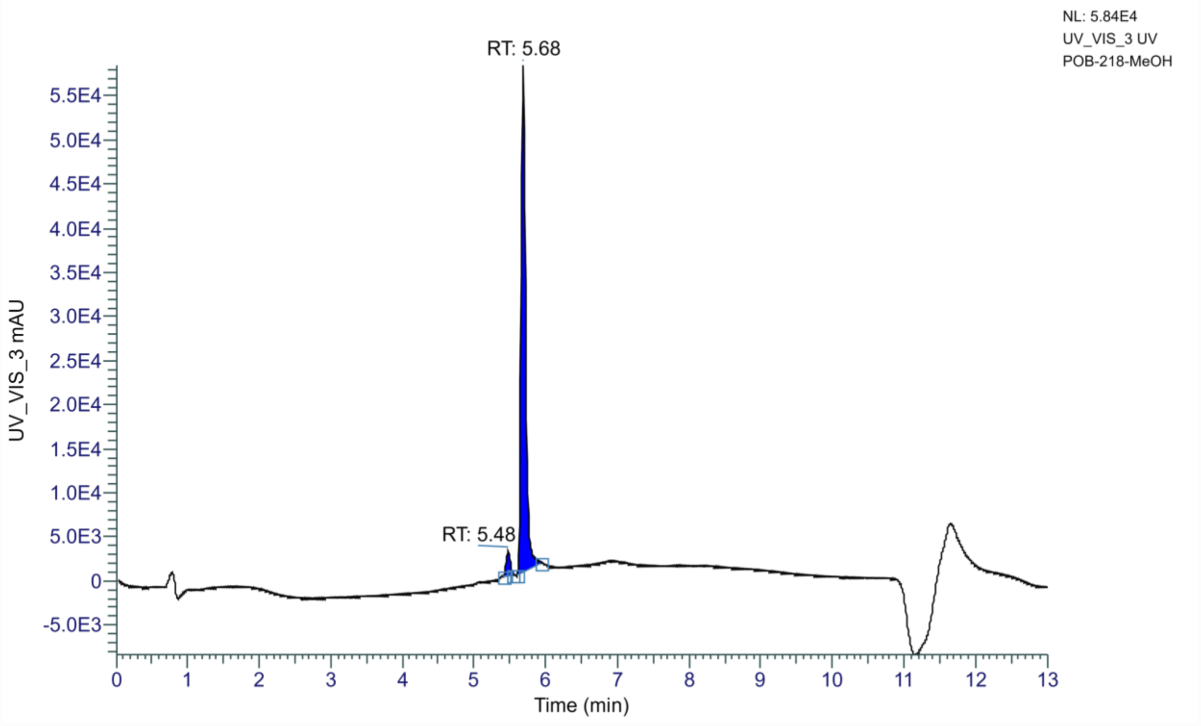

RT (Min)	% Area	Integration Method
5.48	3.95	Automated - ICIS
5.68	96.05	Automated - ICIS

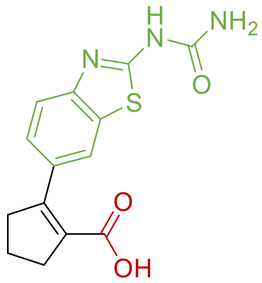

26d

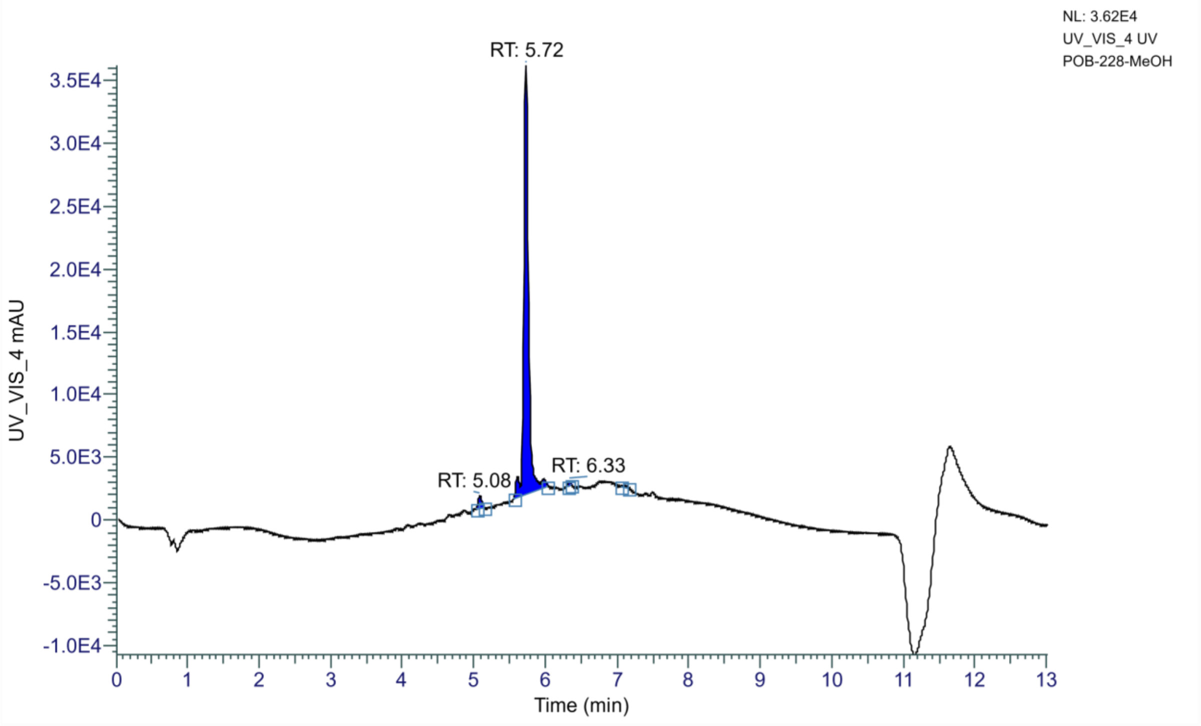

RT (Min)	% Area	Integration Method
5.08	1.96	Automated - ICIS
5.72	97.37	Automated - ICIS
6.33	0.41	Automated - ICIS
7.1	0.26	Automated - ICIS

2 Proof of Stereochemistry

As stated in the paper, the *cis*-configuration from the hydrogenation reactions to give acids *cis*-**17a-f** was confirmed by X-ray crystallography of dihydrofuran *cis*-**17e** (CCDC 2482170). The configurations of cyclopentyl heteroaryl carboxylic acids *cis*-**17a-d** and *cis*-**17f** were assigned by analogy.

Acids *cis*-**17a-f** had characteristic differences in δ_H values for the signals due to the CHAr and CHCO protons. This is summarised in Table S1. Apart from *cis*-**17c**, which was an outlier, the difference in δ_H values were ~0.25-0.4 ppm. This difference is smaller than that observed in the corresponding *trans*-diastereomers (see Table S3).

Table S1 NMR spectroscopic data for acids *cis*-**17**

**Compound**	δ_H value (C*H*Ar) / ppm	δ_H value (C*H*CO) / ppm	Solvent	Difference in δ_H values (separation) / ppm
cis-**17a**	3.71-3.62	3.34-3.26	MeOD-d_4	~0.35
cis-**17b**	3.72-3.61	3.29-3.21	MeOD-d_4	~0.4
cis-**17c**	3.92-3.77	3.28-3.24	MeOD-d_4	~0.55
cis-**17d**	3.64-3.55	3.24-3.15	MeOD-d_4	~0.4
cis-**17e**	3.36	3.15-3.04	$CDCl_3$	~0.25
cis-**17f**	3.49-3.40	3.17-3.09	MeOD-d_4	~0.35

Ester *cis*-**22b** was prepared by hydrogenation of **19c** (Scheme S1) and the product *cis*-**22b** had different ^{1}H and ^{13}C NMR spectra to *trans*-**22b** prepared by the photoredox route (*vide infra*).

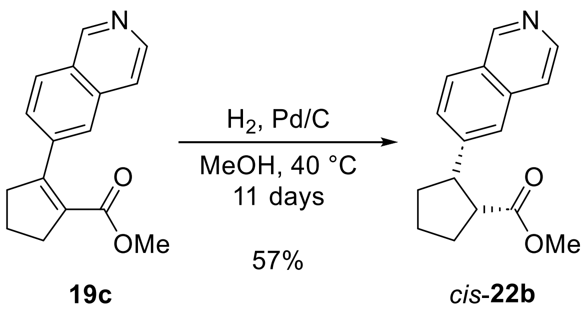

Scheme S1

Esters *cis*-**22a-e** had characteristic differences in δ_H values for the signals due to the CHAr and CHCO protons. This is summarised in Table S2. The difference in δ_H values was ~0.3 ppm in all cases. This difference is smaller than that observed in the corresponding *trans*-diastereomers (see Table S4).

Table S2 NMR spectroscopic data for esters *cis*-**22**

**Compound**	δ_H value (C*H*Ar) / ppm	δ_H value (C*H*CO) / ppm	Solvent	Difference in δ_H values (separation) / ppm
cis-**22a**	3.56-3.46	3.26-3.21	$CDCl_3$	~0.3
cis-**22b**	3.58	3.29	$CDCl_3$	~0.3
cis-**22c**	3.62-3.51	3.30-3.25	$CDCl_3$	~0.3
cis-**22d**	3.58-3.46	3.25-3.19	$CDCl_3$	~0.3
cis-**22e**	3.67-3.53	3.31-3.25	$CDCl_3$	~0.3

As stated in the paper, the *trans*-configuration of acid *trans*-**17i** was confirmed by X-ray crystallography (CCDC 2482171). The configurations of cyclopentyl heteroaryl carboxylic acids *trans*-**17a** and *trans*-**17g-h** were assigned by analogy. In addition, ester hydrolysis and epimerisation of the Barluenga reaction products, diastereomeric mixtures of *trans*-/*cis*-mixtures **22a** and **22c**, gave acids *trans*-**17g** and *trans*-**17i** (identical by ^{1}H NMR spectroscopy to those obtained via the photoredox-ester hydrolysis route) respectively. The *trans*-configurations of acids *trans*-**17b** and *trans*-**17j** were assigned by analogy. In addition, the *trans*-configuraiton of acid *trans*-**32** was assured as it was synthesised from *trans*-**22a** of known configuration.

Acids *trans*-**17a-b**, **g-j** and *trans*-**32** had characteristic differences in δ_H values for the signals due to the CHAr and CHCO protons. This is summarised in Table S3. The difference in δ_H values was ~0.5-0.7 ppm. This difference is larger than that observed in the corresponding *cis*-diastereomers (see Table S1).

Table S3 NMR spectroscopic data for acids *trans*-**17a-b**, **g-j** and *trans*-**32**

**Compound**	δ_H value (C*H*Ar) / ppm	δ_H value (C*H*CO) / ppm	Solvent	Difference in δ_H values (separation) / ppm
trans-**17a**	3.53	2.91	DMSO-d_6	~0.6
trans-**17b**	3.41	2.91	DMSO-d_6	~0.5
trans-**17g**	3.50	2.94	DMSO-d_6	~0.55
trans-**17h**	3.64	3.02	$CDCl_3$	~0.6
trans-**17i**	3.55	2.97	$CDCl_3$	~0.6
trans-**17j**	3.76	3.05	$CDCl_3$	~0.7
trans-**32**	3.12-3.05	2.64-2.57	MeOD-d_4	~0.5

As outlined in Scheme S1, the configuration of ester *cis*-**22b** is confirmed by the hydrogenation route and comparison with other *cis*-acids and *cis*-esters. This, in turn, confirms the *trans* configuration of ester *trans*-**22b**. The *trans*-configurations of esters *trans*-**22a** and *trans*-**22c-e** were assigned by analogy.

Esters *trans*-**22a-e** had characteristic differences in δ_H values for the signals due to the CHAr and CHCO protons. This is summarised in Table S4. The difference in δ_H values was ~0.6 ppm. This difference is larger than that observed in the corresponding *cis*-diastereomers (see Table S2).

Table S4 NMR spectroscopic data for esters *trans*-**22**

**Compound**	δ_H value (C*H*Ar) / ppm	δ_H value (C*H*CO) / ppm	Solvent	Difference in δ_H values (separation) / ppm
trans-**22a**	3.56-3.46	2.90	$CDCl_3$	~0.6
trans-**22b**	3.53	2.91	$CDCl_3$	~0.6
trans-**22c**	3.62-3.51	2.95	$CDCl_3$	~0.6
trans-**22d**	3.58-3.46	2.91	$CDCl_3$	~0.6
trans-**22e**	3.67-3.53	2.98	$CDCl_3$	~0.6

Methyl -2-(isoquinolin-6-yl)cyclopentane-1-carboxylate *cis*-22b

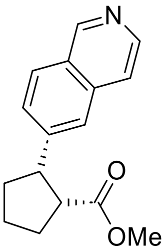

cis-**22b**

Using general procedure B, alkene **19c** (80 mg, 0.32 mmol, 1.0 eq.) and 10% Pd/C (8 mg) in MeOH (4 mL) at 40 °C for 11 days gave the crude product. Purification by prep-TLC on silica with 75:25 *n*-hexane-EtOAc eluent gave ester *cis*-**22b** (46 mg, 57%) as a white solid, mp 45 - 48 °C; R_F (5:1 *n*-hexane-EtOAc) 0.10, IR (ATR) 2950, 2872, 1728 (C=O), 1630, 1491, 1434, 1369, 1197, 1169 cm^{-1}; ^{1}H NMR (400 MHz, $CDCl_3$) 9.19 (s, 1H, Ar), 8.48 (d, J = 6.0 Hz, 1H, Ar), 7.87 (d, J = 8.5 Hz, 1H, Ar), 7.62 (d, J = 1.5 Hz, 1H, Ar), 7.59 (d, J = 6.0 Hz, 1H, Ar), 7.47 (dd, J = 8.5, 1.5 Hz, 1H, Ar), 3.62-3.55 (m, 1H, CHAr), 3.32-3.26 (m, 1H, CHCO), 3.14 (s, 1H, Me), 2.27 – 2.02 (m, 5H, CH), 1.84 – 1.76 (m, 1H, CH); ^{13}C NMR (100.6 MHz, $CDCl_3$) δ 174.6 (C=O), 152.2 (*ipso*-Ar), 144.6 (Ar), 143.3 (Ar), 135.9 (*ipso*-Ar), 128.4 (Ar), 127.7 (*ipso*-Ar), 127.2 (Ar), 124.6 (Ar), 120.5 (Ar), 51.1 (*C*HCO), 49.8 (*C*HAr), 31.2 (CH_2), 28.9 (CH_2), 24.9 (CH_2); HRMS (ESI) *m/z* calcd for $C_{16}H_{17}NO_2$ $(M + H)^+$ 256.1334, found 256.1332 (–0.8 ppm error).

Lab book reference XW-002-074.

3 Initial Molecular Modelling of Mac1 Inhibitors

The structures of the priority 1 and priority 2 compounds are shown in Figure S1 and the docked poses in Tables S5 and S6.

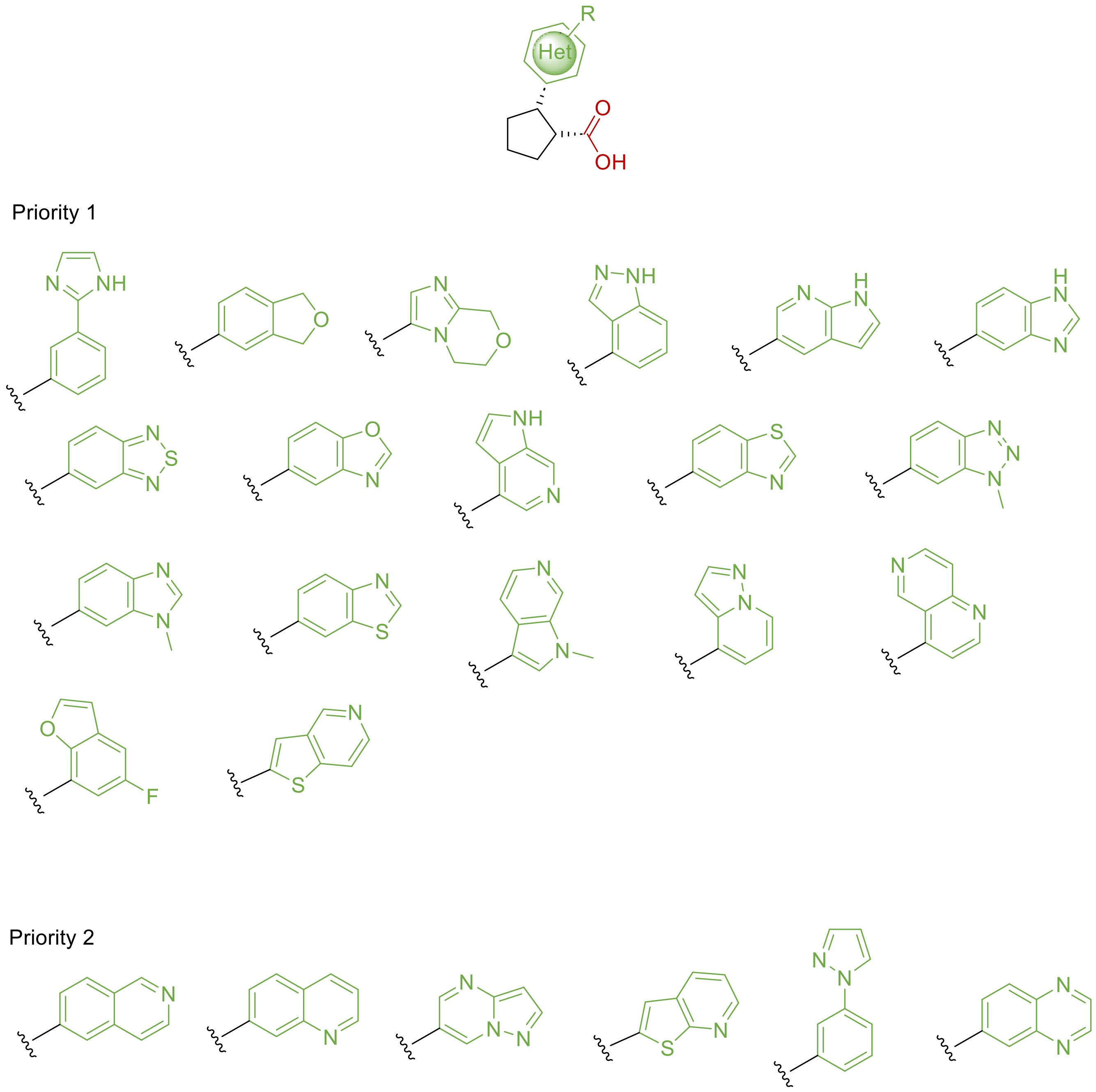

Figure S1

Table S5. Priority 1 modelling results

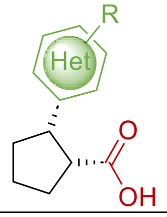

Structure of aromatic group	Pose
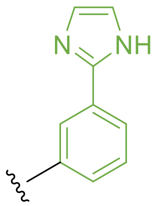	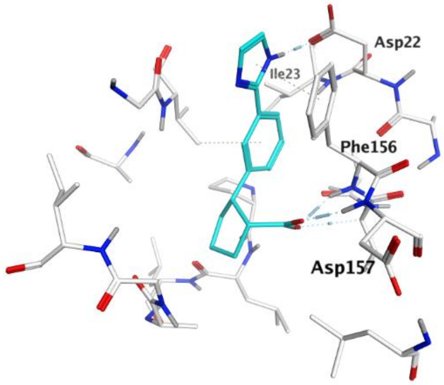
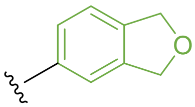	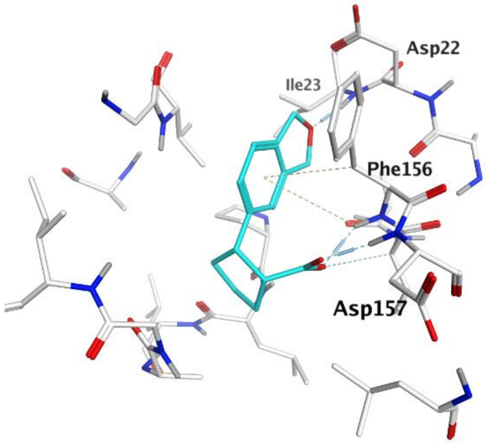
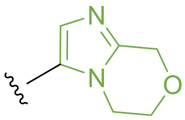	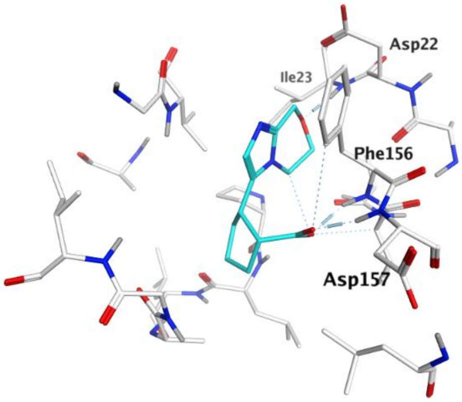

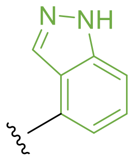 N<br>NH	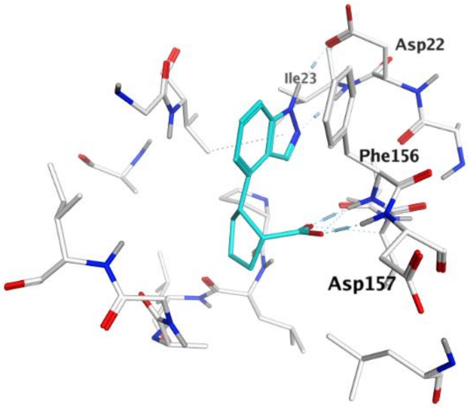 Asp22<br>Ile23<br>Phe156<br>Asp157
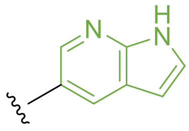 N<br>H<br>N	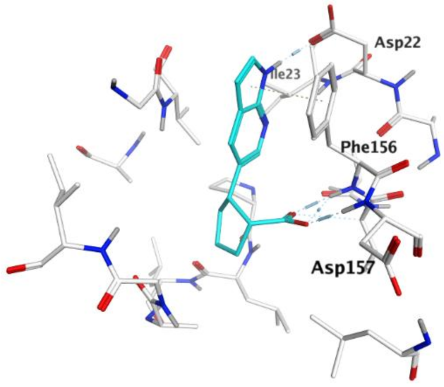 Asp22<br>Ile23<br>Phe156<br>Asp157
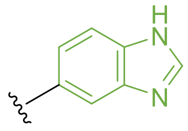 H<br>N<br>N	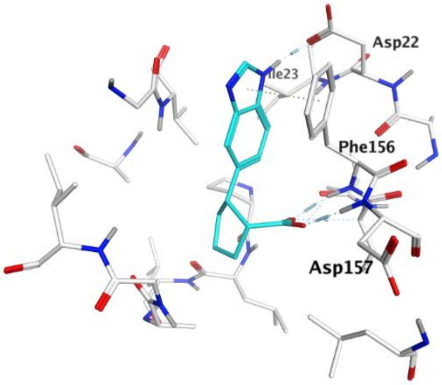 Asp22<br>Ile23<br>Phe156<br>Asp157

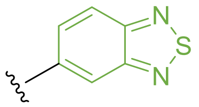 N<br>S<br>N	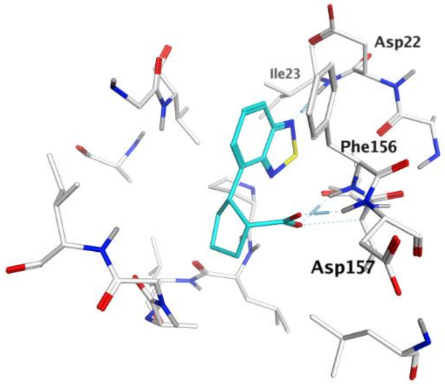 Asp22<br>Ile23<br>Phe156<br>Asp157
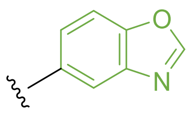 O<br>N	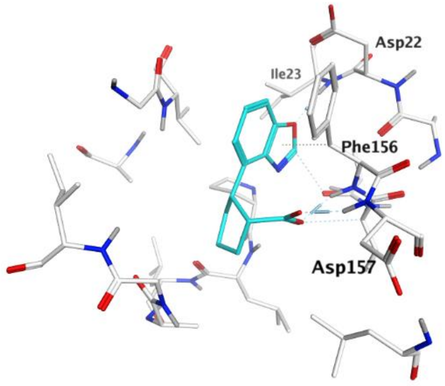 Asp22<br>Ile23<br>Phe156<br>Asp157
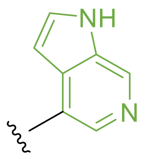 NH<br>N	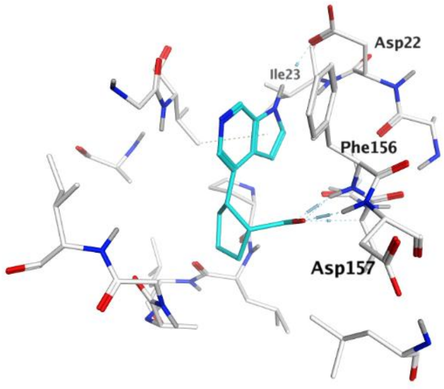 Asp22<br>Ile23<br>Phe156<br>Asp157

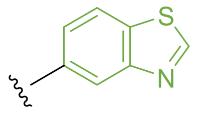

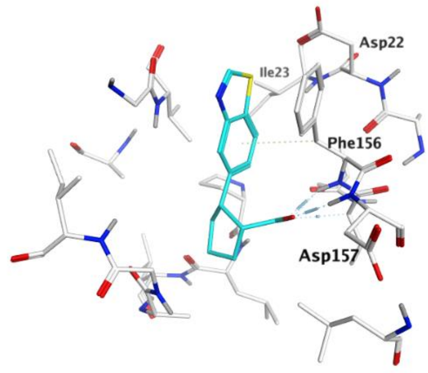
Asp22
Ile23
Phe156
Asp157

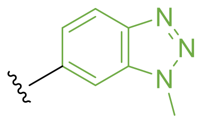

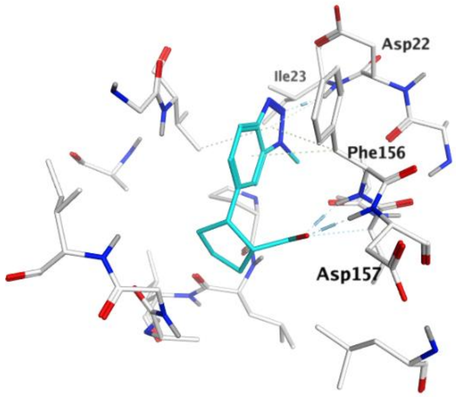
Asp22
Ile23
Phe156
Asp157

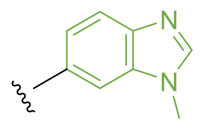

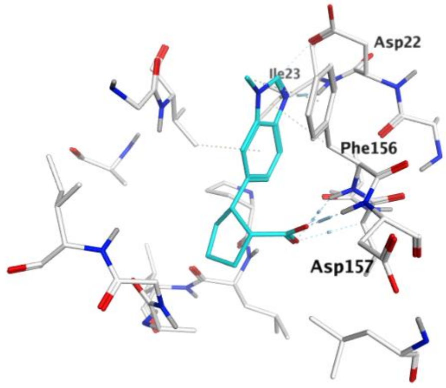
Asp22
Ile23
Phe156
Asp157

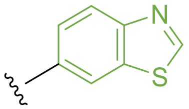	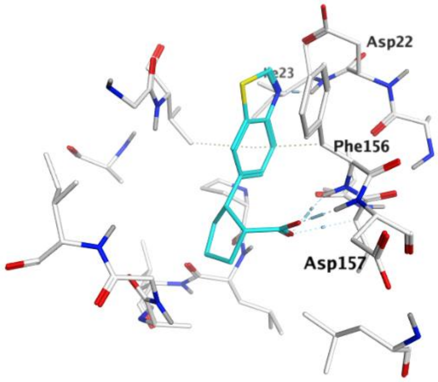 Asp22 Ile23 Phe156 Asp157
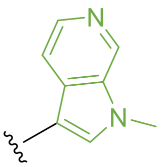	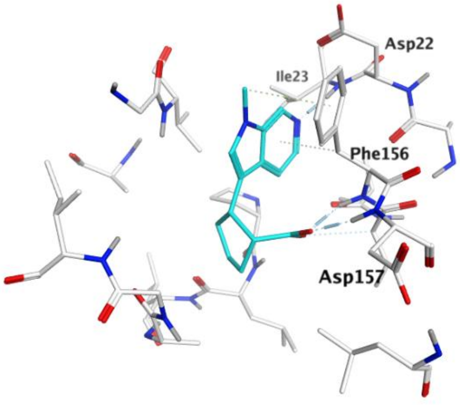 Asp22 Ile23 Phe156 Asp157
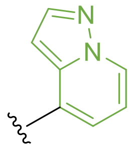	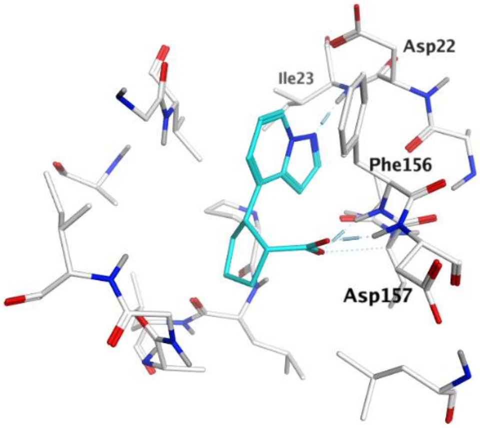 Asp22 Ile23 Phe156 Asp157

<table>
<tr>
<td>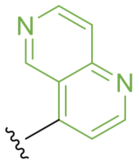
</td>
<td>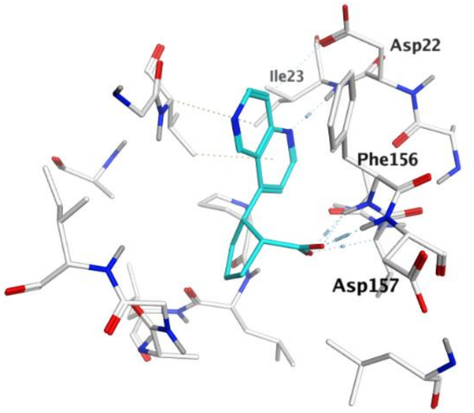
</td>
</tr>
<tr>
<td>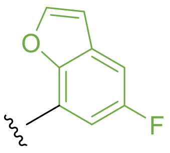
</td>
<td>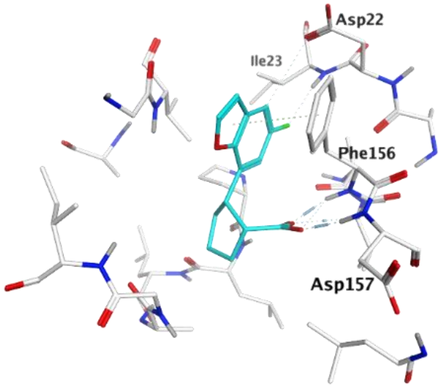
</td>
</tr>
<tr>
<td>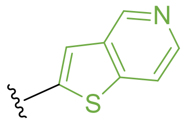
</td>
<td>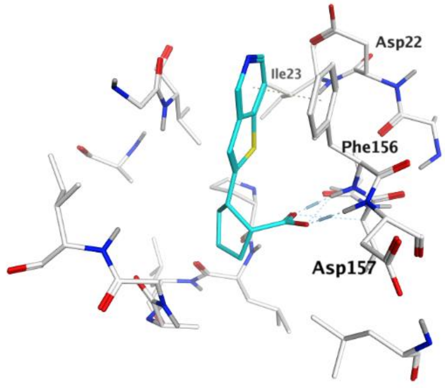
</td>
</tr>
</table>

Table S6. Priority 2 modelling results

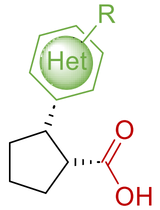

Structure of aromatic group	Pose
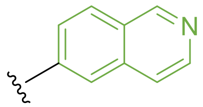	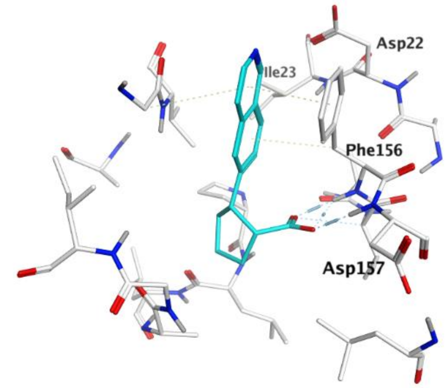
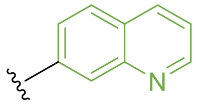	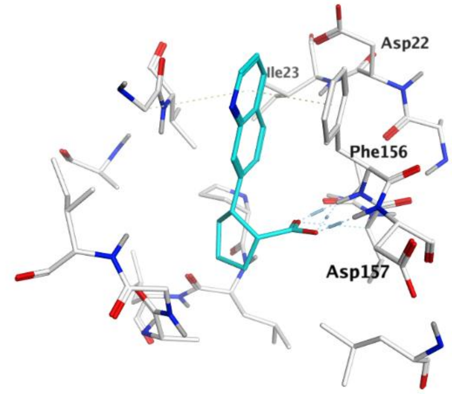
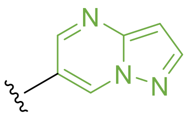	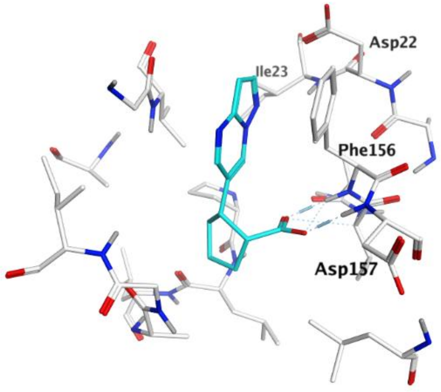

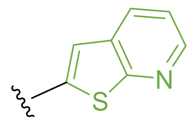	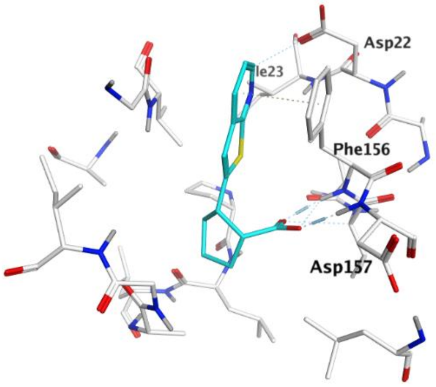Asp22<br>Ile23<br>Phe156<br>Asp157
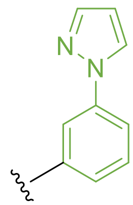	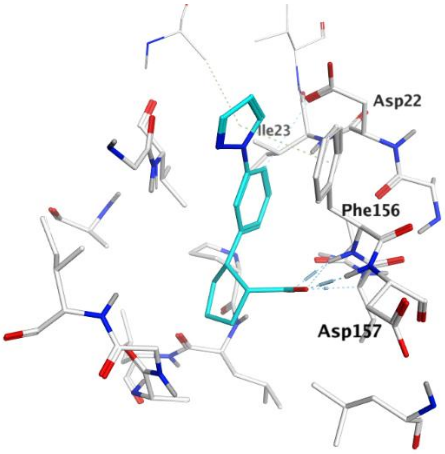Asp22<br>Ile23<br>Phe156<br>Asp157
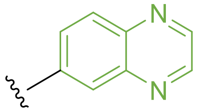	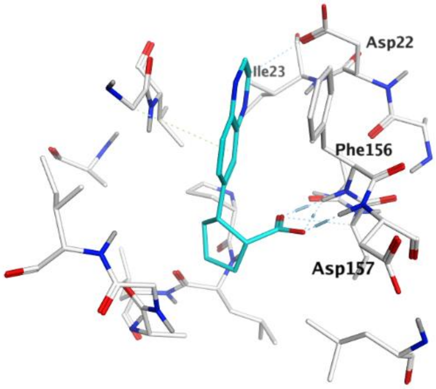Asp22<br>Ile23<br>Phe156<br>Asp157

4 HTRF Assay of Mac1 Inhibitors

The IC_{50} curves of fragments bound to Mac1 are shown in Figures S2-S5.

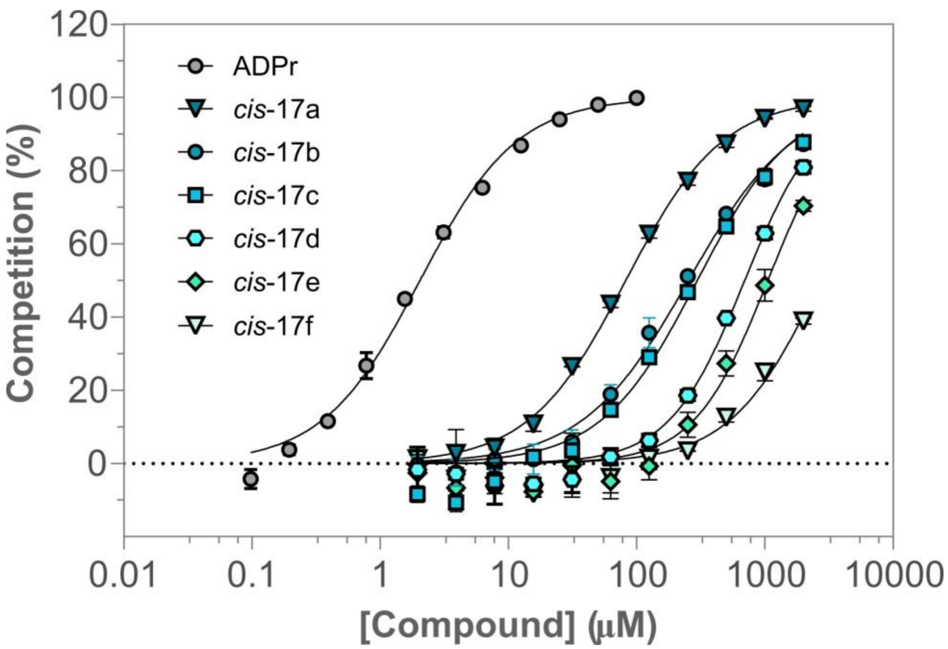

Figure S2. Plot of % Competition *vs* Concentration for *cis*-**17a-f**

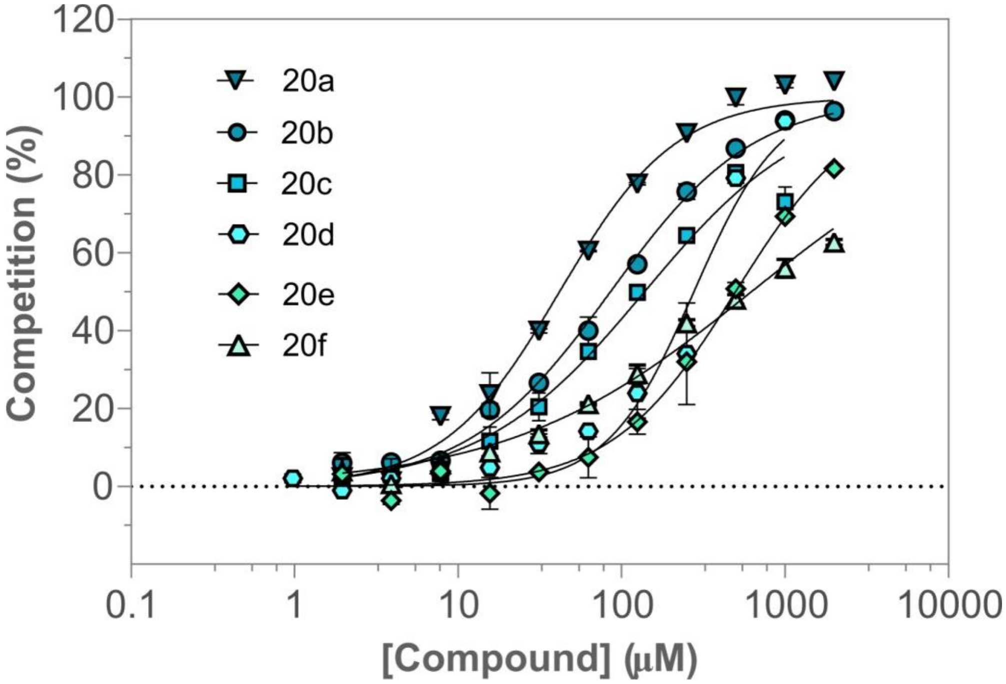

Figure S3. Plot of % Competition *vs* Concentration for **20a-f**

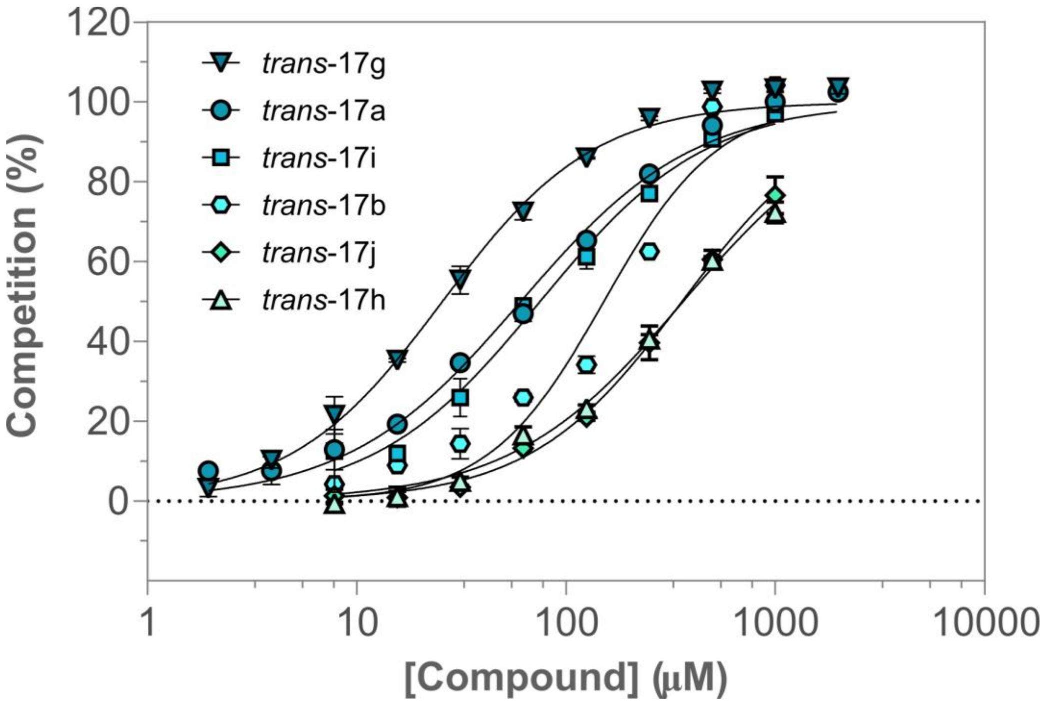

Figure S4. Plot of % Competition *vs* Concentration for *trans*-**17a-b** and *trans*-**17g-j**

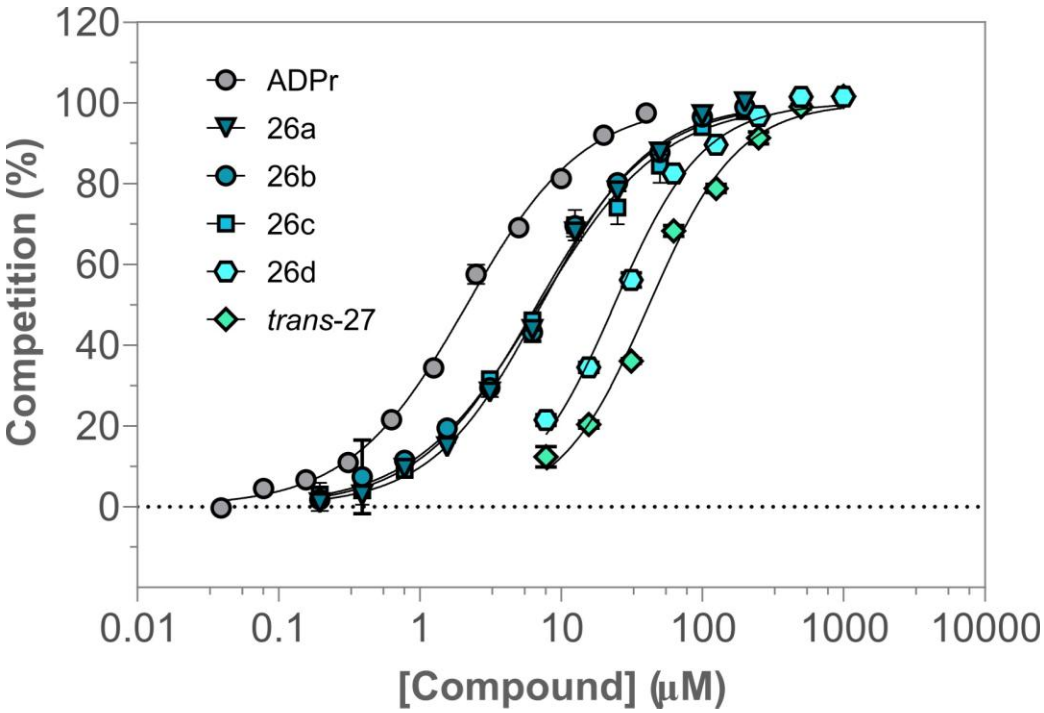

Figure S5. Plot of % Competition *vs* Concentration for **26a-d** and *trans*-**27**

5 X-ray Crystallography of Mac1-inhibitor Co-crystals

Data collection and refinement statistics are summarised in Table S7.

Table S7. Data collection and refinement statistics for protein X-ray crystallography

PDB ID	7IJN	7IJO	7IJP	7IJQ
**Dataset**	Mac1-x10181	Mac1-x10183	Mac1-x10184	Mac1-x10199
**Ligand**	**POB0175**	**POB0177**	**POB0179**	**POB0176**
**Wavelength**	0.9179	0.9179	0.9179	0.9179
**Resolution range**	88.55 - 1.22 (1.25 - 1.22)	88.75 - 1.18 (1.24 - 1.18)	88.71 - 1.22 (1.26 - 1.22)	88.61 - 1.21 (1.24 - 1.21)
**Space group**	P 43	P 43	P 43	P 43
**Cell (a b c)**	88.55 88.55 39.62	88.75 88.75 39.54	88.71 88.71 39.55	88.61 88.61 39.39
**Cell (alpha beta gamma)**	90.00 90.00 90.00	90.00 90.00 90.00	90.00 90.00 90.00	90.00 90.00 90.00
**Total reflections**	396079 (1119)	400576 (222)	397194 (1173)	396431 (830)
**Unique reflections**	79818 (1074)	78423 (215)	81580 (1123)	78572 (807)
**Multiplicity**	5.00 (1.00)	5.10 (1.00)	4.90 (1.00)	5.00 (1.00)
**Completeness (%)**	87.20 (23.00)	77.50 (4.30)	88.70 (24.20)	84.20 (17.60)
**Mean I/sigma(I)**	28.60 (-)	38.40 (-)	7.50 (-)	25.70 (-)
**R-merge**	0.041 (0.122)	0.029 (0.565)	0.106 (0.881)	0.040 (0.417)
**R-rim**	0.018 (0.122)	0.012 (0.565)	0.045 (0.881)	0.017 (0.417)
**CC-half**	0.998 (0.984)	0.999 (0.739)	0.994 (0.604)	0.999 (0.725)
**R-factor**	0.145 (0.167)	0.140 (0.217)	0.174 (0.416)	0.153 (0.241)
**R-free**	0.169 (0.176)	0.169 (0.249)	0.206 (0.473)	0.174 (0.247)
**Number of total atoms**	3050	3106	3062	3030
**atoms for ligands**	38	18	26	17
**atoms for waters**	452	502	484	450
**Number of polymer residues**	334	335	334	334
**Wilson B-factor**	11.9	13.9	14.0	13.5
**Average B-factor**	16.8	18.8	20.6	18.9
**B-factor for ligands**	23.2	15.9	17.7	17.9
**B-factor for solvent**	28.4	31.0	33.1	31.0
**RMS(bonds)**	0.013	0.014	0.013	0.013
**RMS(bond angles)**	1.21	1.25	1.20	1.27

**RMS(dihedral angles)**	5.63	5.57	4.78	5.34

Table S7 continued. Data collection and refinement statistics for protein X-ray crystallography

**PDB ID**	**7IJR**	**7IJS**	**7IJT**	**7IJU**
**Dataset**	Mac1-x10313	Mac1-x10314	Mac1-x10331	Mac1-x10390
**Ligand**	**POB0208**	**POB0209**	**POB0185**	**POB0206**
**Wavelength**	0.9212	0.9212	0.9212	0.9212
**Resolution range**	13.76 - 1.21 (1.25 - 1.21)	16.81 - 1.16 (1.19 - 1.16)	16.54 - 1.15 (1.19 - 1.15)	20.00 - 1.29 (1.39 - 1.29)
**Space group**	P 43	P 43	P 43	P 1 21 1
**Cell (a b c)**	89.22 89.22 39.83	88.98 88.98 39.78	88.97 88.97 39.87	37.54 33.52 60.41
**Cell (alpha beta gamma)**	90.00 90.00 90.00	90.00 90.00 90.00	90.00 90.00 90.00	90.00 96.68 90.00
**Total reflections**	886196 (3892)	884635 (1093)	892137 (510)	199621 (2539)
**Unique reflections**	91734 (2360)	92993 (1021)	94301 (498)	37415 (1172)
**Multiplicity**	9.70 (1.60)	9.50 (1.10)	9.50 (1.00)	5.30 (2.20)
**Completeness (%)**	93.60 (49.20)	85.90 (19.00)	83.90 (8.80)	94.20 (57.60)
**Mean I/sigma(I)**	15.10 (-)	26.60 (-)	17.50 (-)	7.80 (-)
**R-merge**	0.052 (1.398)	0.042 (0.319)	0.058 (0.632)	0.068 (4.218)
**R-rim**	0.015 (1.225)	0.013 (0.319)	0.017 (0.632)	0.030 (3.723)
**CC-half**	0.996 (0.374)	0.999 (0.883)	0.999 (0.844)	0.997 (0.241)
**R-factor**	0.185 (0.535)	0.144 (0.303)	0.150 (0.425)	0.187 (0.534)
**R-free**	0.219 (0.571)	0.164 (0.308)	0.172 (0.377)	0.259 (0.553)
**Number of total atoms**	3020	3168	3065	1470
**atoms for ligands**	22	33	21	18
**atoms for waters**	436	534	460	190
**Number of polymer residues**	333	335	332	166
**Wilson B-factor**	17.4	11.2	12.9	19.3
**Average B-factor**	27.3	18.9	20.6	26.5
**B-factor for ligands**	33.1	20.4	18.6	24.8
**B-factor for solvent**	40.0	31.5	34.7	39.3
**RMS(bonds)**	0.013	0.013	0.014	0.012

**RMS(bond angles)**	1.16	1.18	1.19	1.15
**RMS(dihedral angles)**	4.73	5.37	5.26	4.22

Table S7 continued. Data collection and refinement statistics for protein X-ray crystallography

**PDB ID**	**7IJV**	**7IJW**	**7IJX**	**7IJY**
**Dataset**	Mac1-x10395	Mac1-x10399	Mac1-x10400	Mac1-x10407
**Ligand**	**POB0207**	**POB0208**	**POB0209**	**POB0185**
**Wavelength**	0.9212	0.9212	0.9212	0.9212
**Resolution range**	17.28 - 1.27 (1.30 - 1.27)	20.27 - 1.61 (1.65 - 1.61)	15.36 - 1.38 (1.46 - 1.38)	37.38 - 1.48 (1.50 - 1.48)
**Space group**	P 1 21 1	P 1 21 1	P 1 21 1	P 1 21 1
**Cell (a b c)**	37.64 33.57 60.95	37.62 33.44 61.27	37.62 33.32 60.88	37.65 33.52 60.69
**Cell (alpha beta gamma)**	90.00 97.34 90.00	90.00 97.10 90.00	90.00 96.54 90.00	90.00 96.86 90.00
**Total reflections**	209055 (2650)	134065 (5767)	182643 (6316)	164523 (6207)
**Unique reflections**	37254 (1096)	19954 (915)	30727 (1604)	25356 (1239)
**Multiplicity**	5.60 (2.40)	6.70 (6.30)	5.90 (3.90)	6.50 (5.00)
**Completeness (%)**	92.60 (52.40)	99.60 (91.90)	98.60 (96.10)	100.00 (99.40)
**Mean I/sigma(I)**	10.30 (-)	5.60 (-)	6.30 (-)	8.80 (-)
**R-merge**	0.065 (1.671)	0.122 (2.524)	0.105 (2.382)	0.099 (2.541)
**R-rim**	0.028 (1.340)	0.051 (1.091)	0.045 (1.356)	0.042 (1.261)
**CC-half**	0.996 (0.362)	0.989 (0.249)	0.996 (0.337)	0.997 (0.212)
**R-factor**	0.190 (0.471)	0.234 (0.439)	0.226 (0.475)	0.208 (0.460)
**R-free**	0.251 (0.461)	0.285 (0.340)	0.268 (0.444)	0.251 (0.442)
**Number of total atoms**	1451	1399	1466	1435
**atoms for ligands**	22	18	17	17
**atoms for waters**	156	114	165	162
**Number of polymer residues**	166	166	166	166
**Wilson B-factor**	19.3	28.8	21.7	23.8
**Average B-factor**	28.5	38.2	28.6	28.7
**B-factor for ligands**	41.3	40.5	26.0	27.9
**B-factor for solvent**	41.5	44.2	36.6	38.3

**RMS(bonds)**	0.013	0.010	0.011	0.011
**RMS(bond angles)**	1.16	1.05	1.07	1.03
**RMS(dihedral angles)**	4.56	3.65	3.65	3.84

Table S7 continued. Data collection and refinement statistics for protein X-ray crystallography

**PDB ID**	**7IJZ2**	**7IK0**	**7IK1**	**7IK2**
**Dataset**	Mac1-x10516	Mac1-x10525	Mac1-x10529	Mac1-x10558
**Ligand**	**POB0221**	**POB0211**	**POB0215**	**POB0228**
**Wavelength**	0.9221	0.9221	0.9221	0.922
**Resolution range**	37.13 - 1.32 (1.33 - 1.32)	36.96 - 1.37 (1.38 - 1.37)	37.16 - 1.34 (1.35 - 1.34)	13.26 - 1.15 (1.21 - 1.15)
**Space group**	P 1 21 1	P 1 21 1	P 1 21 1	P 1 21 1
**Cell (a b c)**	37.31 33.78 60.08	37.14 33.59 60.22	37.36 33.61 60.40	37.33 33.73 60.23
**Cell (alpha beta gamma)**	90.00 95.67 90.00	90.00 95.53 90.00	90.00 95.83 90.00	90.00 95.93 90.00
**Total reflections**	199392 (3734)	189651 (5210)	196676 (4268)	212018 (152)
**Unique reflections**	34338 (1236)	31278 (1416)	33029 (1325)	40866 (150)
**Multiplicity**	5.80 (3.00)	6.10 (3.70)	6.00 (3.20)	5.20 (1.00)
**Completeness (%)**	97.30 (71.70)	99.50 (91.60)	97.90 (80.50)	76.90 (5.70)
**Mean I/sigma(I)**	12.00 (-)	12.50 (-)	10.30 (-)	18.70 (-)
**R-merge**	0.081 (1.431)	0.093 (1.747)	0.098 (1.594)	0.045 (0.253)
**R-rim**	0.035 (0.983)	0.040 (1.040)	0.042 (1.033)	0.020 (0.253)
**CC-half**	0.998 (0.305)	0.997 (0.314)	0.992 (0.332)	0.998 (-)
**R-factor**	0.174 (0.378)	0.198 (0.379)	0.200 (0.406)	0.159 (0.396)
**R-free**	0.235 (0.472)	0.244 (0.477)	0.235 (0.343)	0.206 (0.479)
**Number of total atoms**	1503	1477	1494	1508
**atoms for ligands**	18	24	30	21
**atoms for waters**	210	177	185	213
**Number of polymer residues**	166	166	165	166
**Wilson B-factor**	17.6	19.4	18.4	14.6
**Average B-factor**	24.8	26.5	24.6	24.4
**B-factor for ligands**	24.0	31.2	31.3	22.6

**B-factor for solvent**	38.4	37.3	36.4	39.6
**RMS(bonds)**	0.012	0.012	0.012	0.014
**RMS(bond angles)**	1.13	1.09	1.10	1.21
**RMS(dihedral angles)**	4.55	4.35	4.43	5.01

Table S7 continued. Data collection and refinement statistics for protein X-ray crystallography

PDB ID	7IK3	7IK4	7IK5	7IK6
**Dataset**	Mac1-x10580	Mac1-x10581	Mac1-x10584	Mac1-x10585
**Ligand**	**POB0212**	**POB0213**	**POB0216**	**POB0217**
**Wavelength**	0.922	0.922	0.922	0.922
**Resolution range**	33.09 - 1.30 (1.41 - 1.30)	12.94 - 1.15 (1.21 - 1.15)	17.04 - 1.20 (1.24 - 1.20)	37.08 - 1.30 (1.37 - 1.30)
**Space group**	P 1 21 1	P 1 21 1	P 1 21 1	P 1 21 1
**Cell (a b c)**	37.32 33.63 60.37	37.14 33.65 60.24	37.04 34.08 59.25	37.23 34.05 59.62
**Cell (alpha beta gamma)**	90.00 95.69 90.00	90.00 95.75 90.00	90.00 94.82 90.00	90.00 95.15 90.00
**Total reflections**	201635 (2661)	209179 (145)	207633 (844)	200350 (3223)
**Unique reflections**	35712 (981)	38405 (144)	38898 (587)	35698 (1274)
**Multiplicity**	5.60 (2.70)	5.40 (1.00)	5.30 (1.40)	5.60 (2.50)
**Completeness (%)**	94.10 (53.70)	72.70 (5.60)	84.00 (26.50)	97.00 (70.30)
**Mean I/sigma(I)**	6.10 (-)	25.30 (-)	8.80 (-)	5.90 (-)
**R-merge**	0.088 (1.539)	0.038 (0.000)	0.086 (0.975)	0.101 (1.435)
**R-rim**	0.038 (1.115)	0.016 (0.000)	0.038 (0.927)	0.044 (1.110)
**CC-half**	0.998 (0.190)	0.998 (0.000)	0.993 (0.273)	0.997 (0.248)
**R-factor**	0.180 (0.414)	0.169 (0.338)	0.170 (0.419)	0.209 (0.455)
**R-free**	0.254 (0.427)	0.210 (0.376)	0.224 (0.374)	0.251 (0.454)
**Number of total atoms**	1471	1465	1503	1458
**atoms for ligands**	30	26	22	19
**atoms for waters**	167	183	216	185
**Number of polymer residues**	166	166	166	165
**Wilson B-factor**	20.6	15.7	13.3	18.0
**Average B-factor**	28.2	26.8	19.3	24.3

**B-factor for ligands**	30.9	41.0	28.5	23.9
**B-factor for solvent**	43.1	39.8	33.0	36.6
**RMS(bonds)**	0.012	0.014	0.013	0.012
**RMS(bond angles)**	1.12	1.22	1.17	1.10
**RMS(dihedral angles)**	4.12	5.02	4.63	4.30

Table S7 continued. Data collection and refinement statistics for protein X-ray crystallography

**PDB ID**	**7IK7**	**7IK8**
**Dataset**	Mac1-x10586	Mac1-x10590
**Ligand**	**POB0218**	**POB0222**
**Wavelength**	0.922	0.922
**Resolution range**	16.89 - 1.23 (1.26 - 1.23)	16.84 - 1.15 (1.21 - 1.15)
**Space group**	P 1 21 1	P 1 21 1
**Cell (a b c)**	37.31 33.71 60.15	37.25 33.95 59.71
**Cell (alpha beta gamma)**	90.00 95.70 90.00	90.00 95.32 90.00
**Total reflections**	208481 (1518)	210238 (150)
**Unique reflections**	37391 (779)	40702 (148)
**Multiplicity**	5.60 (1.90)	5.20 (1.00)
**Completeness (%)**	86.00 (36.20)	76.90 (5.60)
**Mean I/sigma(I)**	9.70 (-)	24.00 (-)
**R-merge**	0.068 (1.497)	0.057 (0.065)
**R-rim**	0.030 (1.281)	0.025 (0.065)
**CC-half**	0.998 (0.333)	0.996 (0.000)
**R-factor**	0.174 (0.436)	0.153 (0.255)
**R-free**	0.233 (0.459)	0.192 (0.260)
**Number of total atoms**	1508	1528
**atoms for ligands**	24	18
**atoms for waters**	191	245
**Number of polymer residues**	166	166
**Wilson B-factor**	16.4	11.2

**Average B-factor**	25.8	19.4
**B-factor for ligands**	45.6	17.1
**B-factor for solvent**	39.2	33.2
**RMS(bonds)**	0.013	0.014
**RMS(bond angles)**	1.17	1.21
**RMS(dihedral angles)**	4.78	5.38

6 WaterMap Modelling of Mac1 Inhibitors

WaterMap calculations* were performed on crystal structures 5S3T, 7IJN, 7IJX, 7IJY, and 7IK2 using default parameters. Two runs of WaterMap were conducted: one with the co-crystallised ligands retained in the pocket, and one with the ligands removed. The co-crystallised water molecules were treated as solvent during the calculations.

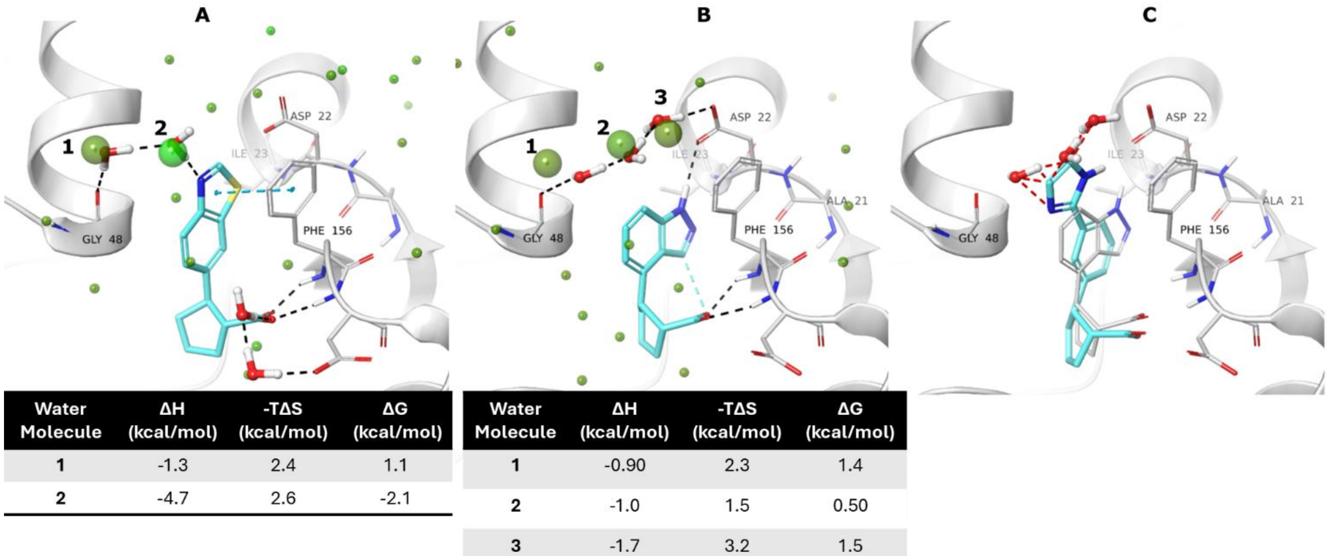

Water Molecule	ΔH (kcal/mol)	-TΔS (kcal/mol)	ΔG (kcal/mol)
1	-1.3	2.4	1.1
2	-4.7	2.6	-2.1

Water Molecule	ΔH (kcal/mol)	-TΔS (kcal/mol)	ΔG (kcal/mol)
1	-0.90	2.3	1.4
2	-1.0	1.5	0.50
3	-1.7	3.2	1.5

Figure S6. A) WaterMap results for *trans*-**17g** (7IJX). B) WaterMap results for *cis*-**17c** (7IJN). C) Superimposition of the docked pose of *cis*-**17f** in 7IJN.
Steric clashes with the co-crystallised water molecules are shown with red, dashed lines.

Water map reference:

* Schrödinger Release 2024-4: WaterMap, Schrödinger, LLC, New York, NY, 2024.

In terms of SAR, one benzothiazole regioisomer, **20a** (IC_{50} 42 μM), considerably outperformed the other, **20e** (IC_{50} 498 μM), in terms of its ability to inhibit Mac1. As outlined above, we analysed the crystal structure of **20a** (PDB: 7IJY) and found that the benzothiazole group interacts with Gly48 *via* two stable water molecules, as suggested by the above WaterMap calculations. In addition, the polarised CH of the benzothiazole appears to form a non-canonical hydrogen bond with the side chain of Asp22. We therefore tentatively ascribe the improved inhibitory potency of **20a** over **20e** to the network of interactions that link Asp22 with Gly48 and are mediated only by one regioisomeric

benzothiazole, namely **20a**. A similar extended water-network contact between Asp22 and Gly48 was observed in the X-ray structure of *cis*-**17c** (PDB: 7IJN). In this case, three water molecules bridge the two amino acids at the opposite sides of the binding pocket, suggesting that this interaction could be involved in retaining inhibitory activity to Mac1. Accordingly, we infer that compounds such as *cis*-**17f**, which would project substituents into a region to disrupt this water network should be inactive, as was observed (IC_{50} >2000 μM).

Analysis of the crystal structure of *trans*-**17g** also indicated interaction of the benzothiazole group with Gly48 *via* two stable water molecules (see above WaterMap calculations).

We note that the above analysis provides some tentative suggestions for some of the differences in activity. However, further work would be required to fully confirm our proposals.

7 *In vitro* de-ADP-ribosylation activity assay using AMP-Glo luminescence detection

Figure S7 shows the results of the *in vitro* de-ADP-ribosylation assay.

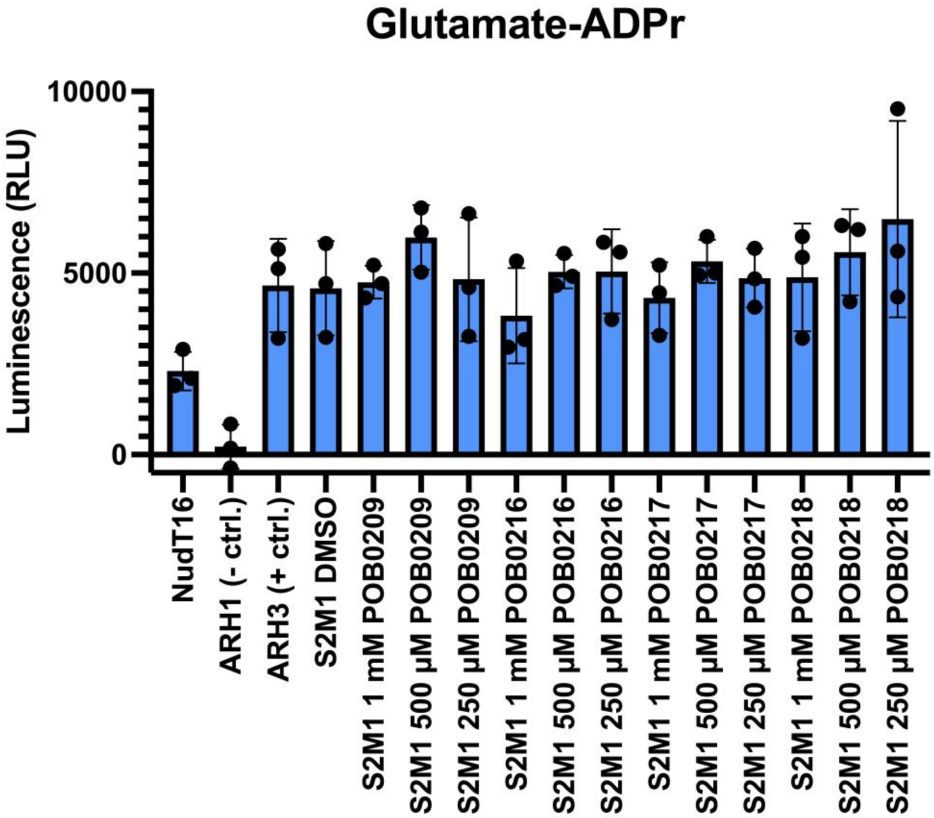

Figure S7. Hydrolytic activity of SARS-CoV-2 Mac1 (S2M1) in the presence of titrated inhibitors (1 mM, 500 µM, and 250 µM) or DMSO vehicle control, using chemically synthesised glutamate-ADPr as substrate. Hydrolytic activity was assessed by monitoring AMP production either directly using NudT16 as a technical positive control or indirectly through Nudix hydrolase 5 (NudT5)-mediated conversion of hydrolase-released ADP-ribose into AMP.† Human ARH1 and ARH3 served as hydrolytic negative (− ctrl.) and positive (+ ctrl.) controls, respectively.‡ NudT16 = Nudix hydrolase 16; ARH1 = ADP-ribosylhydrolase 1; ARH3 = ADP-ribosylhydrolase 3; SM21 = SARS-CoV-2 Mac1; POB0209 = *trans*-**17g**; POB0216 = **26a**; POB0217 = **26b**; POB0218 = **26c**.

† Palazzo, L.; Thomas, B.; Jemth, A.-S.; Colby, T.; Leidecker, O.; Feijs, K. L. H.; Zaja, R.; Loseva, O.; Puigvert, J. C.; Matic, I.; Helledy, T.; Ahel, I. Processing of protein ADP-ribosylation by Nudix hydrolases. *Biochem. J.* **2015**, *468*, 293.
‡ Tashiro, K.; Wijngaarden, S.; Mohapatra, J.; Rack, J. G. M.; Ahel, I.; Filippov, D. V.; Liszczak, G. Chemoenzymatic and Synthetic Approaches To Investigate Aspartate- and Glutamate-ADP-Ribosylation. *J. Am. Chem. Soc.* **2023**, *14*, 14000.

8 ^{1}H and ^{13}C NMR Spectra

400 MHz ^{1}H NMR spectrum; 100.6 MHz ^{13}C NMR spectrum; $CDCl_3$ of **33**

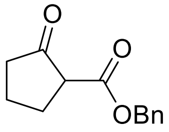

33

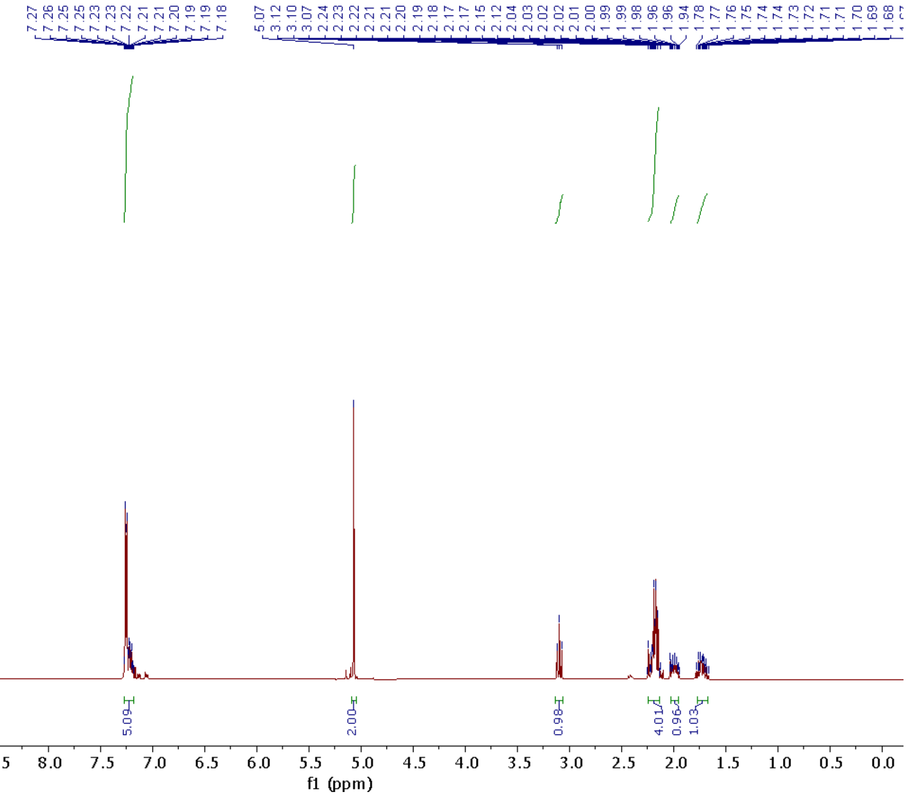

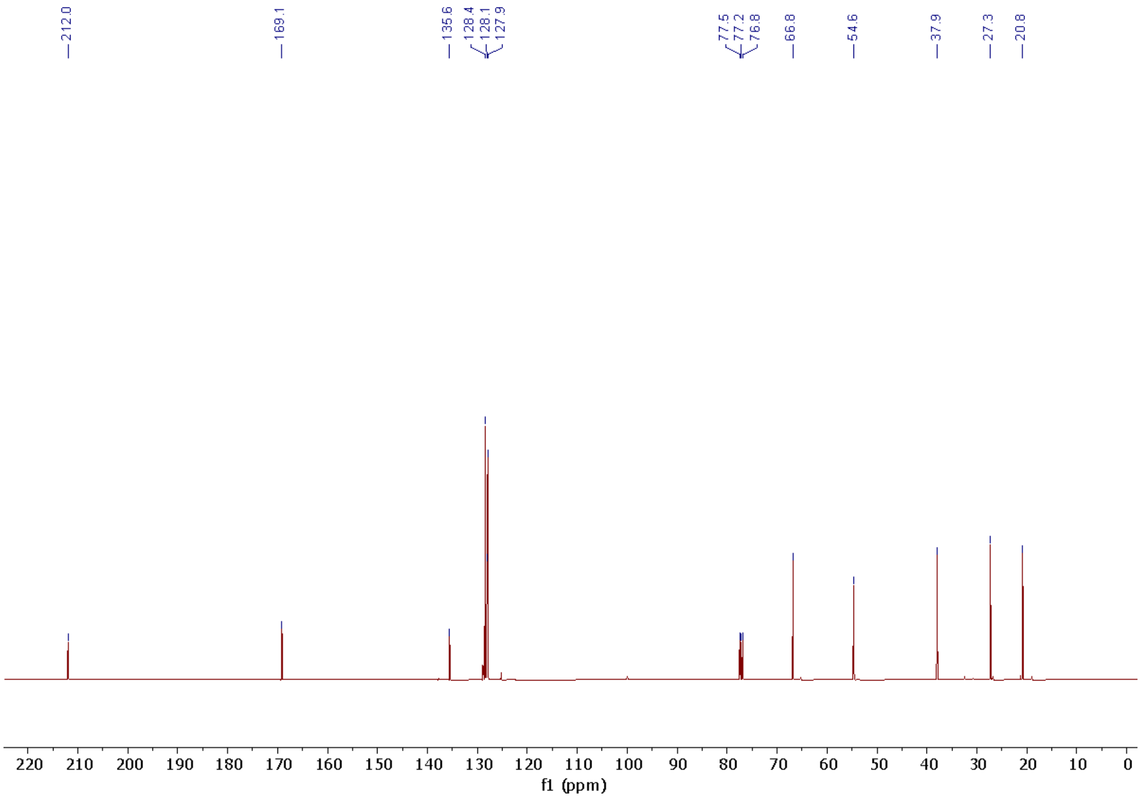

400 MHz ^{1}H NMR spectrum; 100.6 MHz ^{13}C NMR spectrum; 378.5 MHz ^{19}F NMR spectrum; $CDCl_3$ of **15**

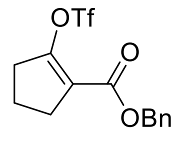

15

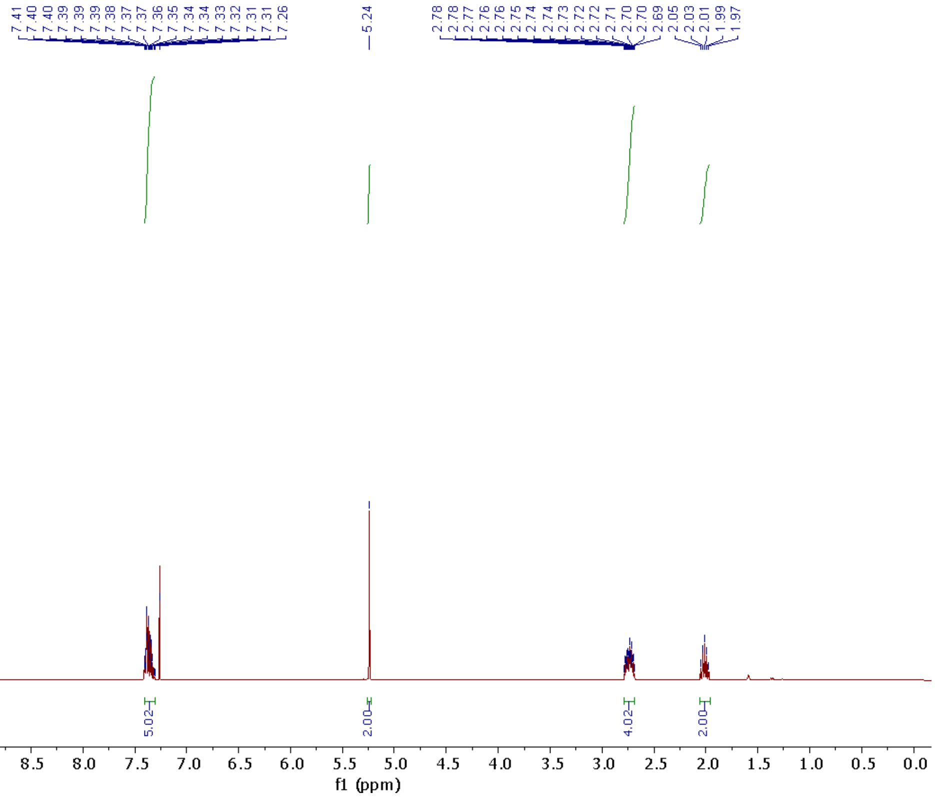

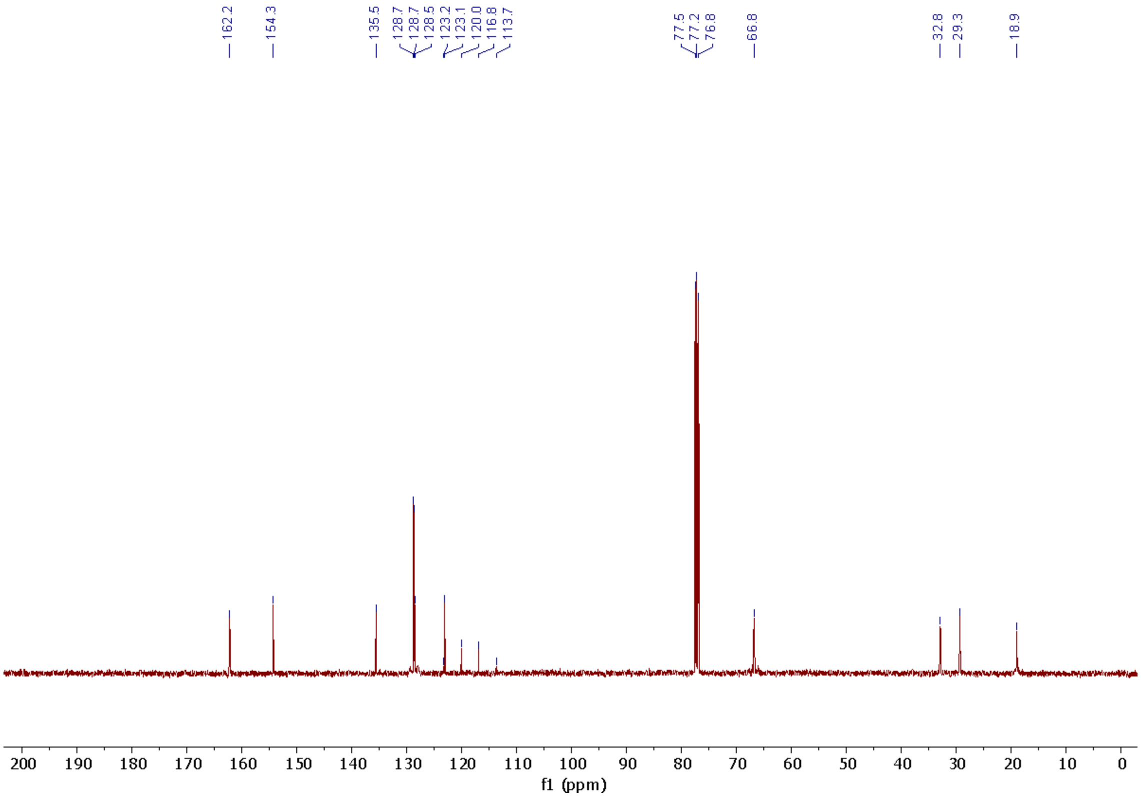

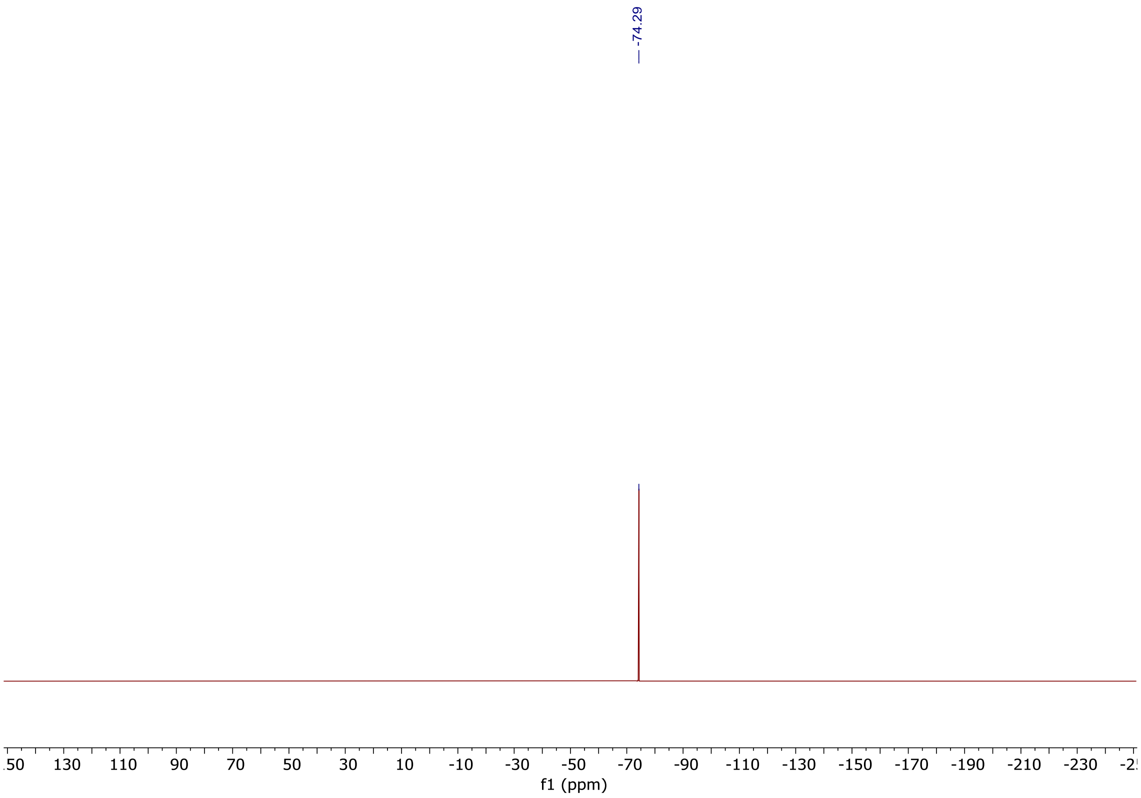
-74.29
150
130
110
90
70
50
30
10
-10
-30
-50
-70
-90
-110
-130
-150
-170
-190
-210
-230
-250
f1 (ppm)

400 MHz ^{1}H NMR spectrum; 100.6 MHz ^{13}C NMR spectrum; $CDCl_3$ of **16a**

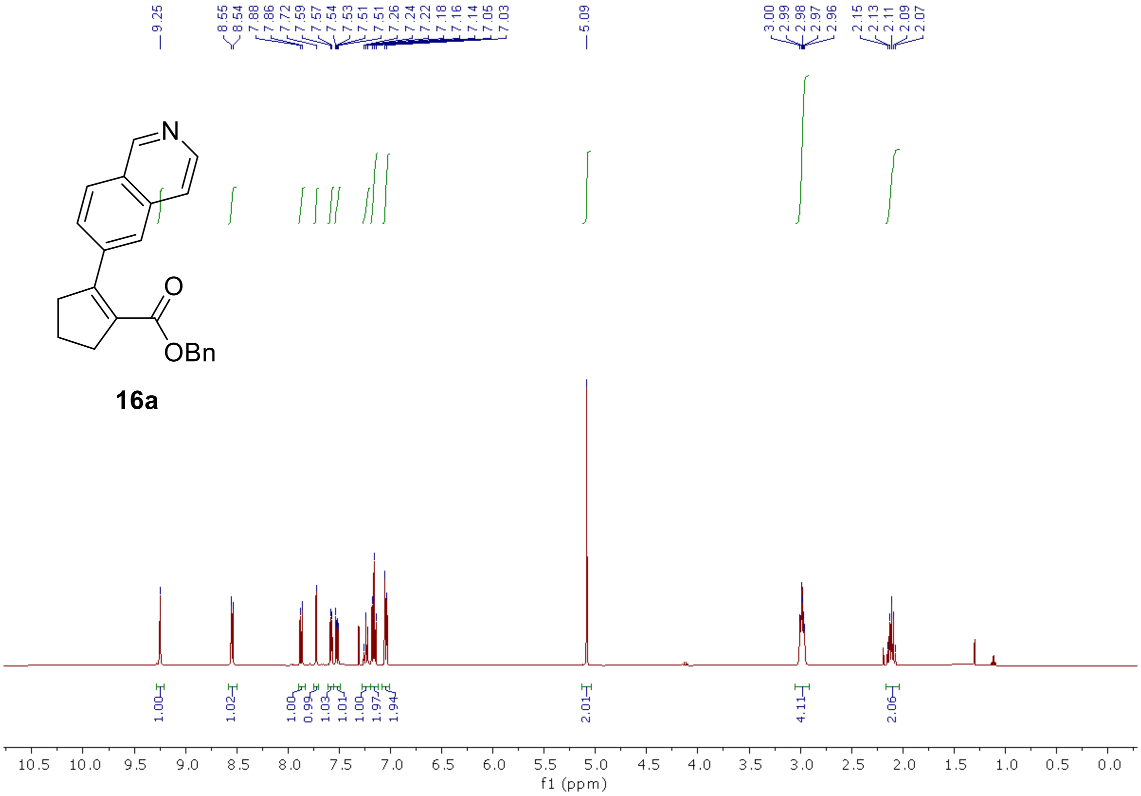

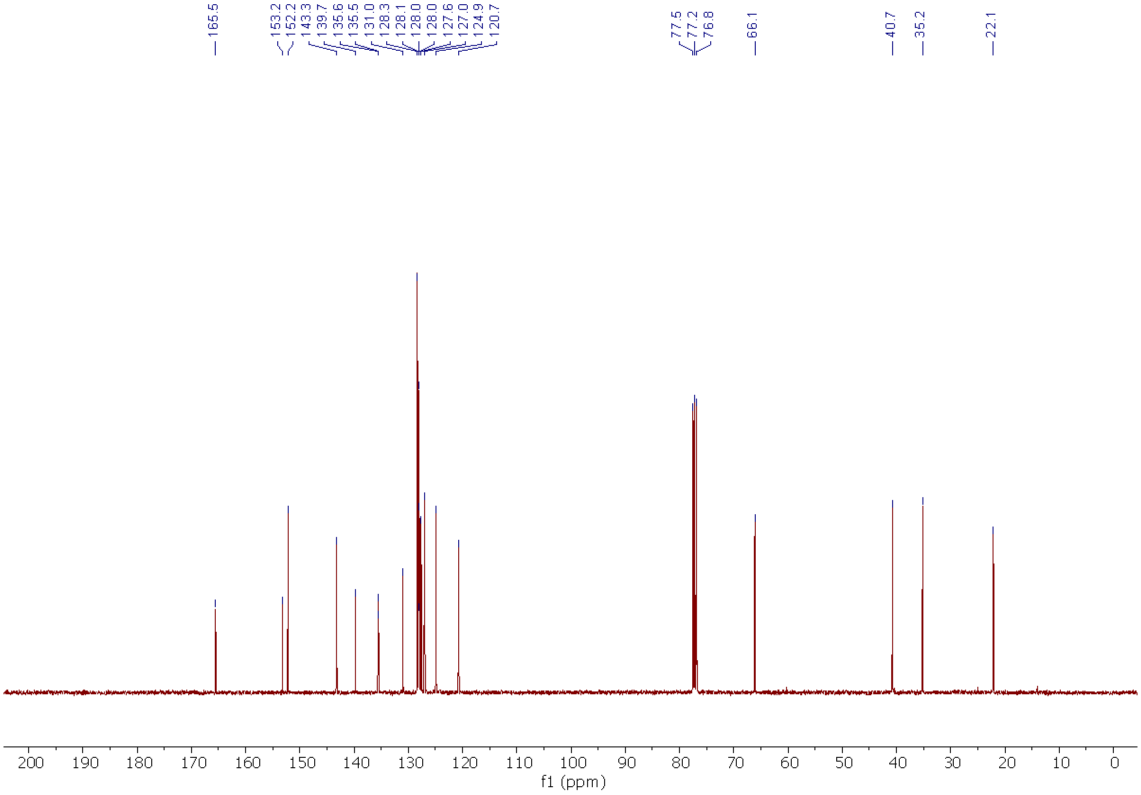

400 MHz ^{1}H NMR spectrum; 100.6 MHz ^{13}C NMR spectrum; $CDCl_3$ of **16b**

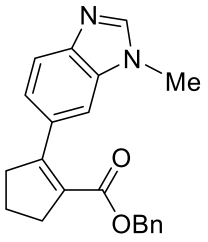

16b

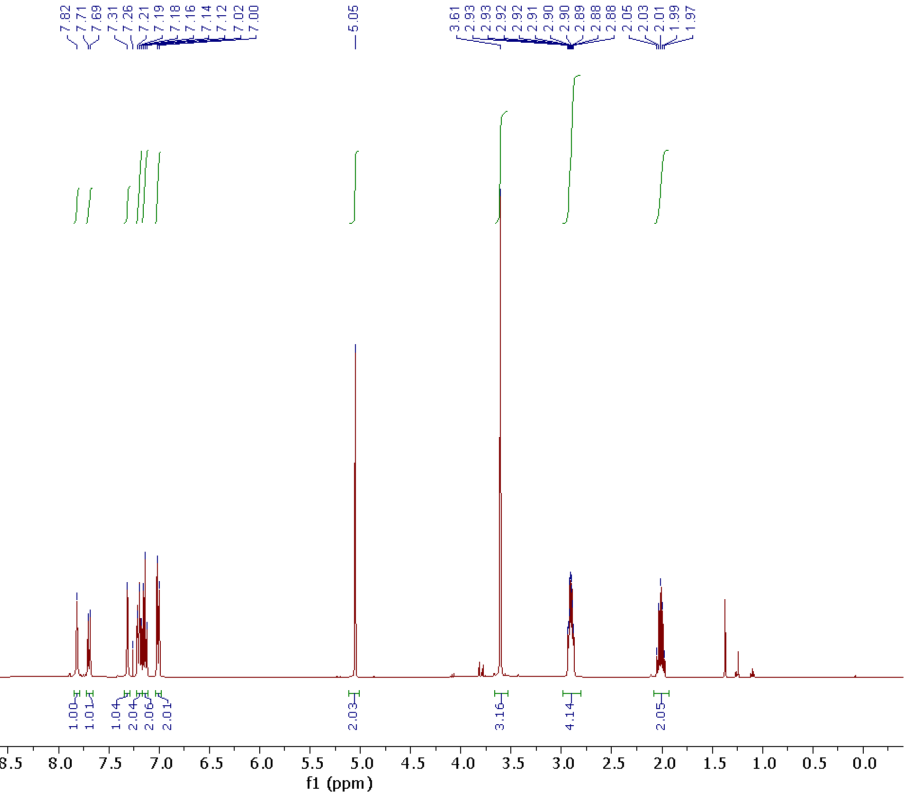

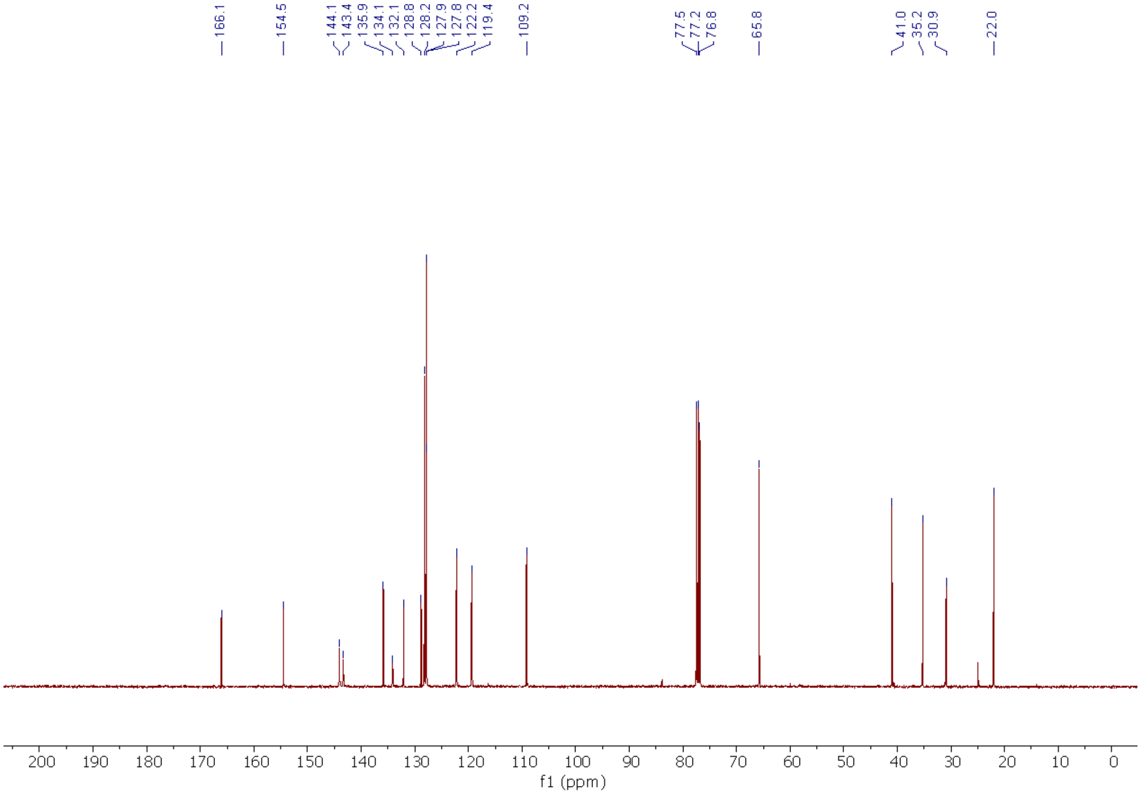

400 MHz ^{1}H NMR spectrum; 100.6 MHz ^{13}C NMR spectrum; $CDCl_3$ of **34**

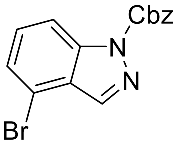

34

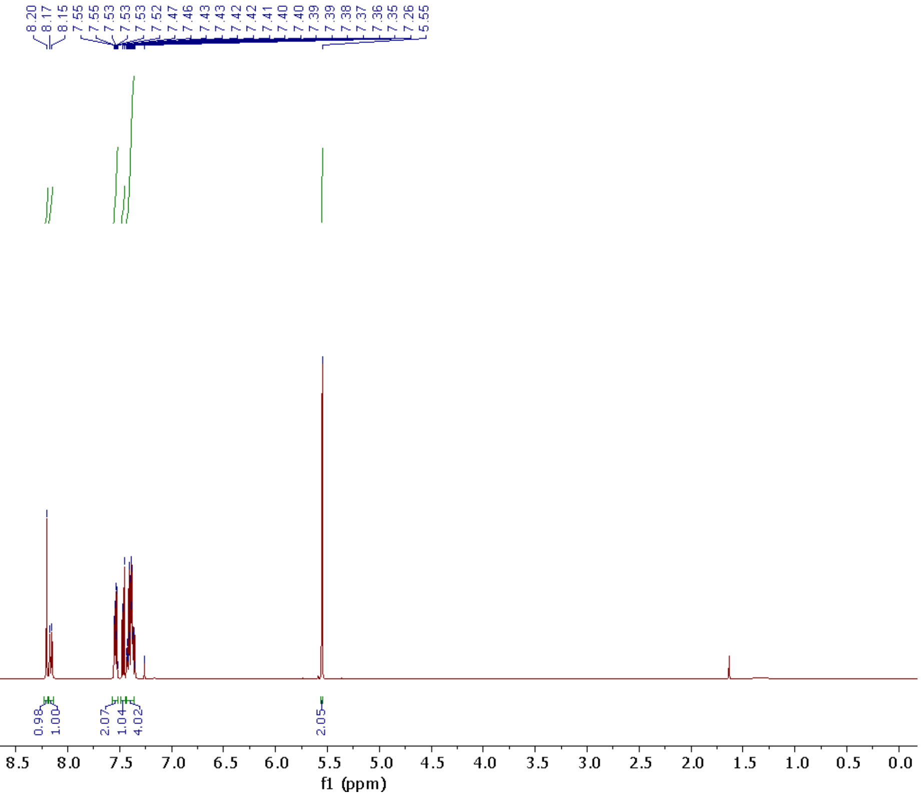

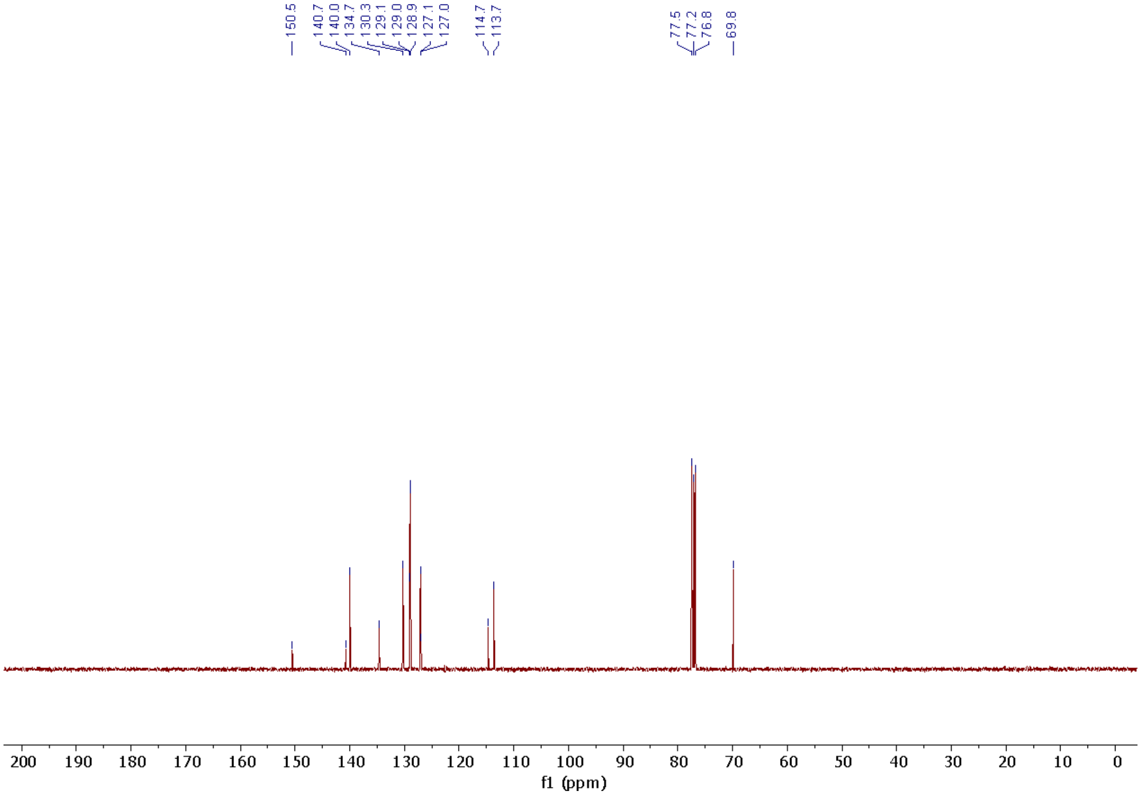

400 MHz ^{1}H NMR spectrum; 100.6 MHz ^{13}C NMR spectrum; $CDCl_3$ of **16c**

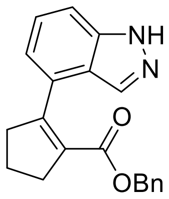

16c

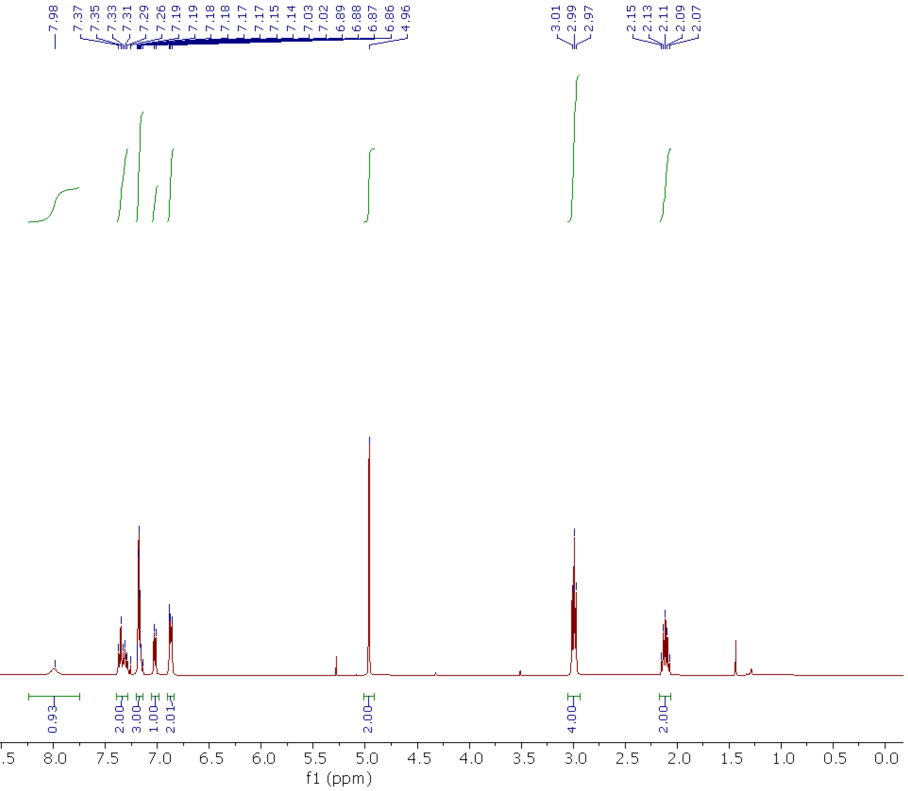

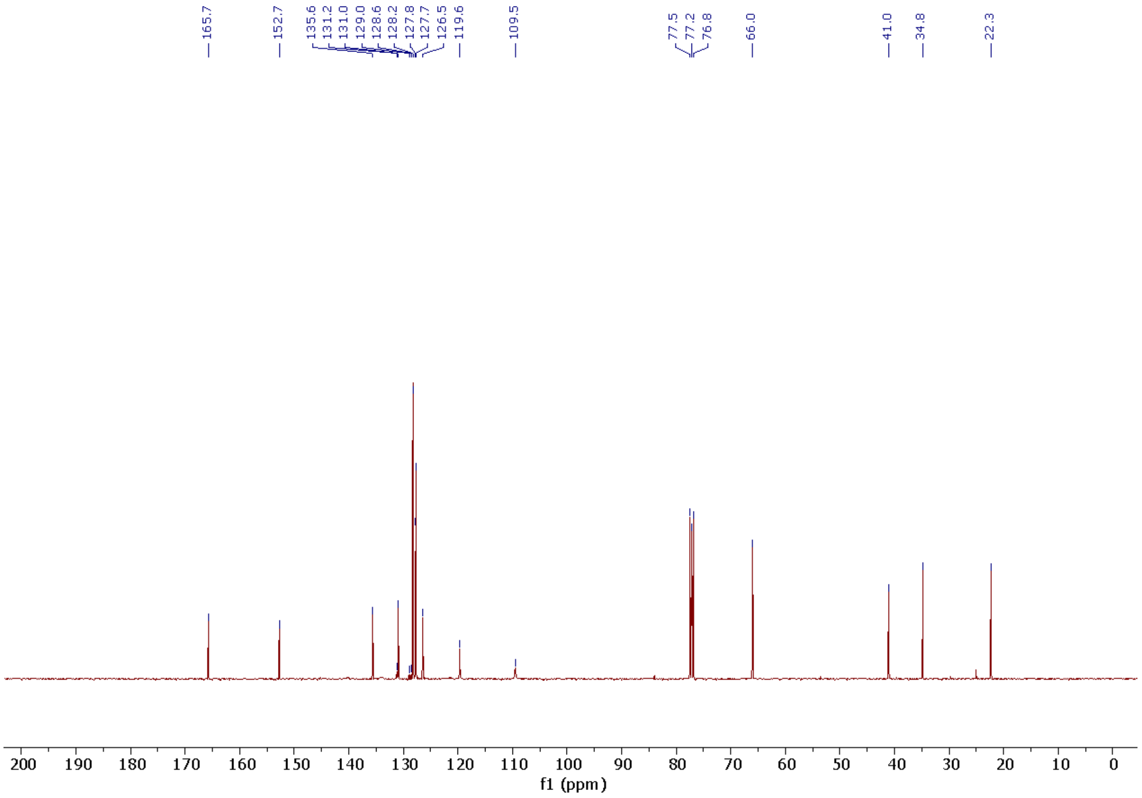

400 MHz ^{1}H NMR spectrum; 100.6 MHz ^{13}C NMR spectrum; $CDCl_3$ of **16d**

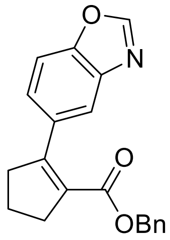

16d

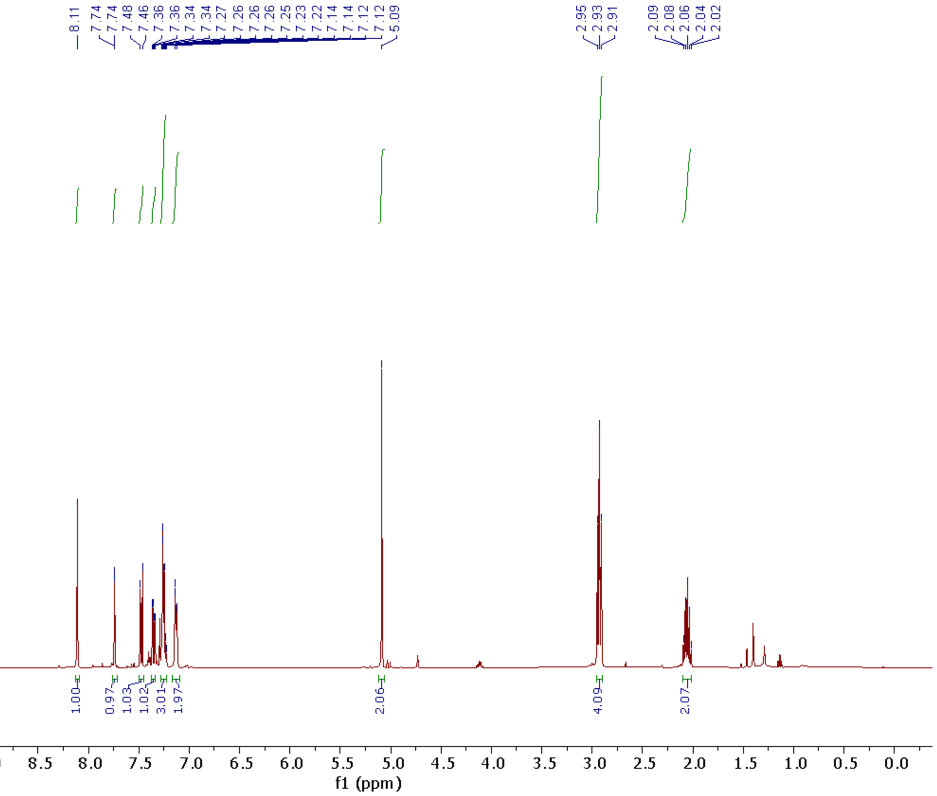

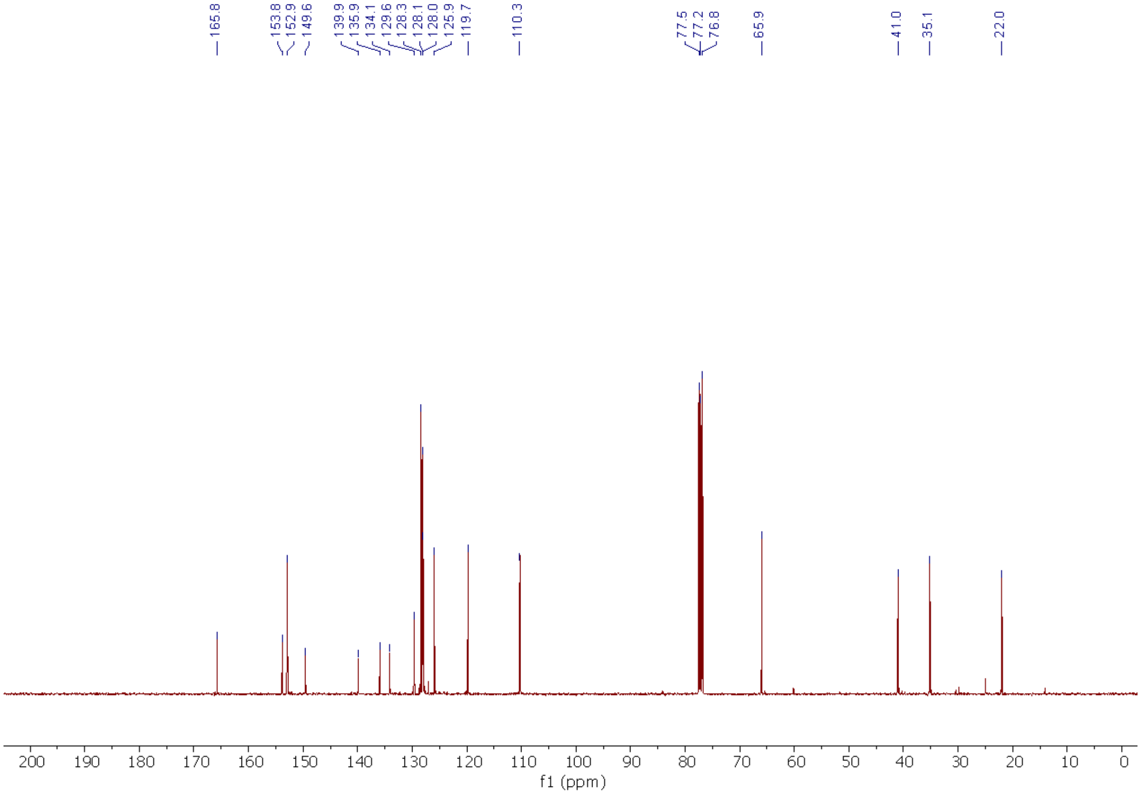

400 MHz ^{1}H NMR spectrum; 100.6 MHz ^{13}C NMR spectrum; 378.5 MHz ^{19}F NMR spectrum; $CDCl_3$ of **16e**

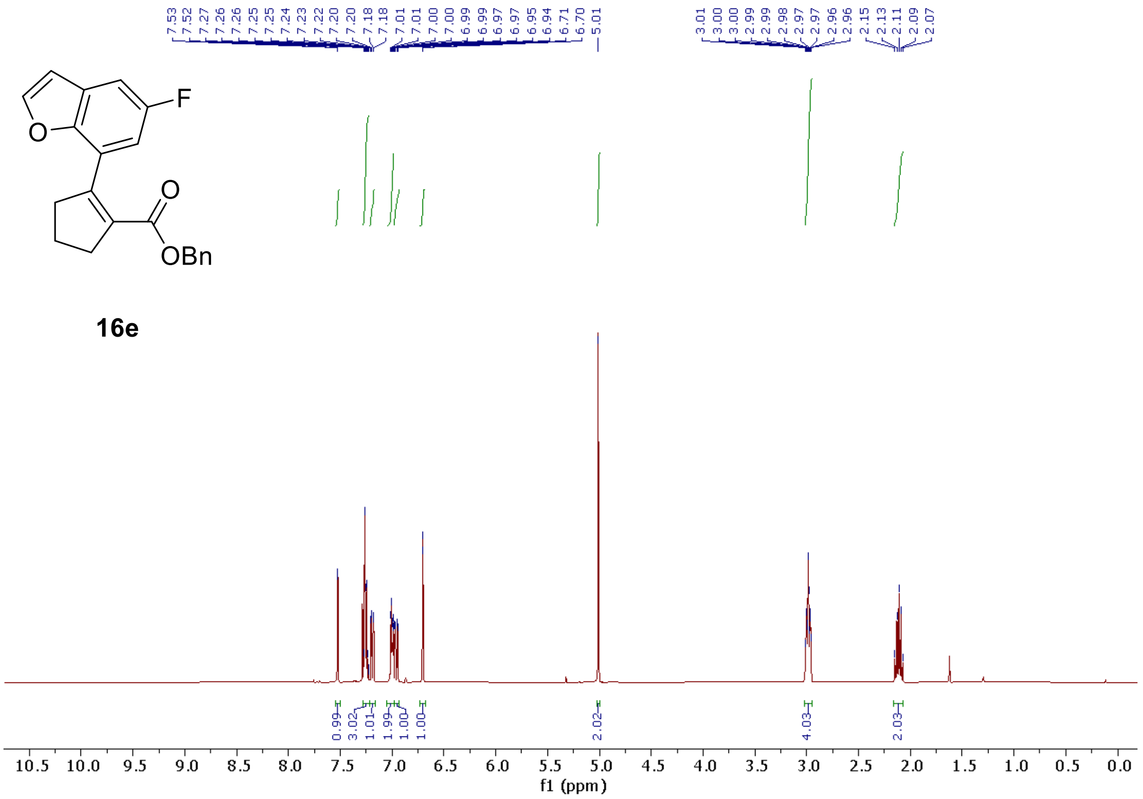

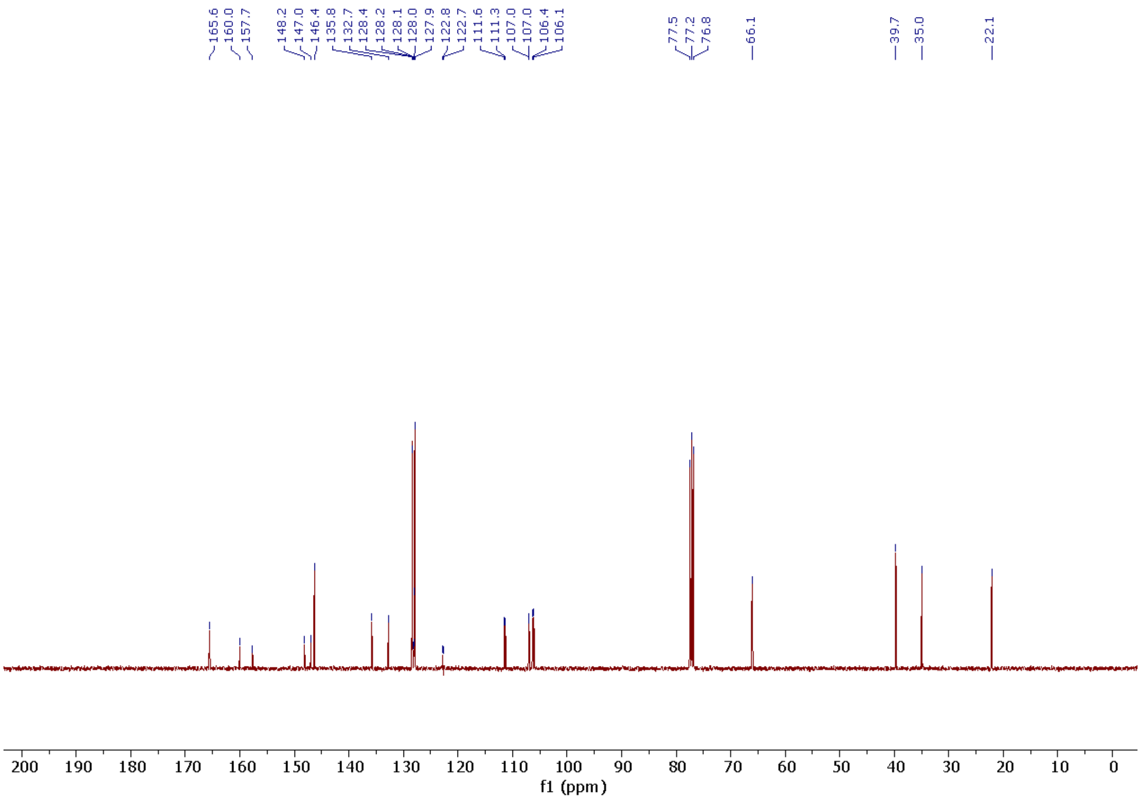

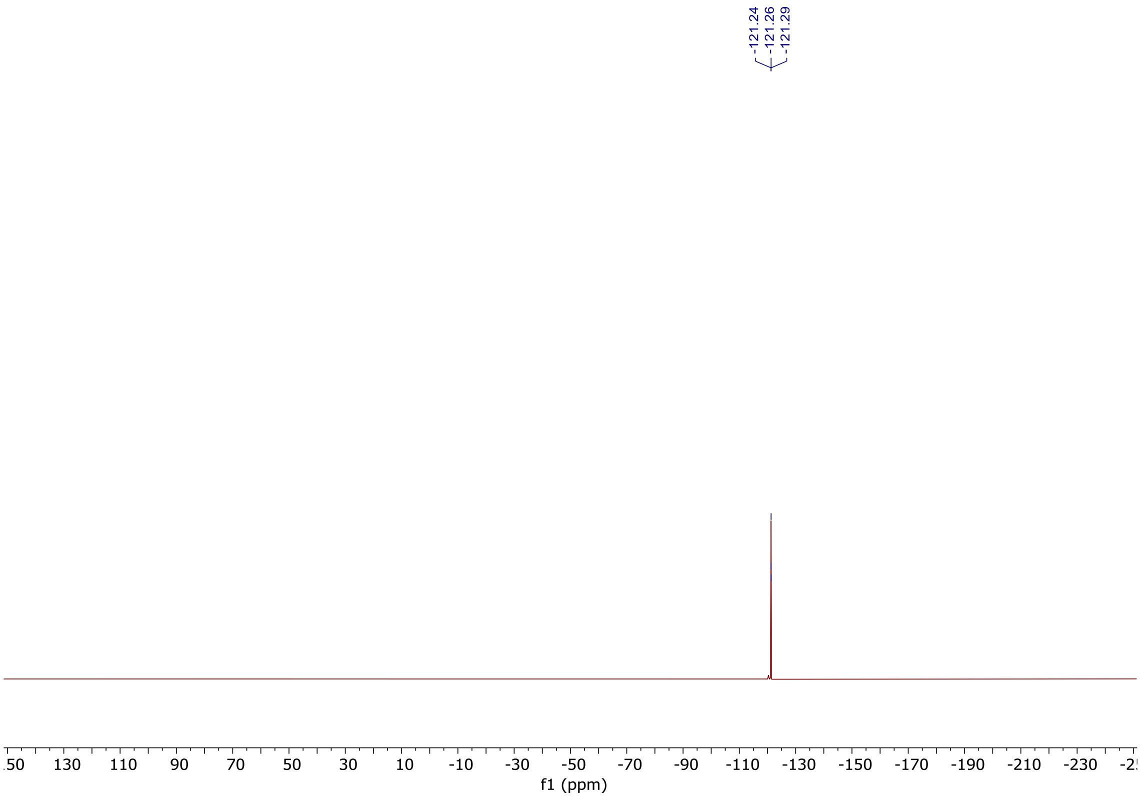
-121.24
-121.26
-121.29
150
130
110
90
70
50
30
10
-10
-30
-50
-70
-90
-110
-130
-150
-170
-190
-210
-230
-25
f1 (ppm)

400 MHz ^{1}H NMR spectrum; 100.6 MHz ^{13}C NMR spectrum; $CDCl_3$ of **35**

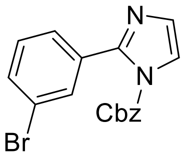

35

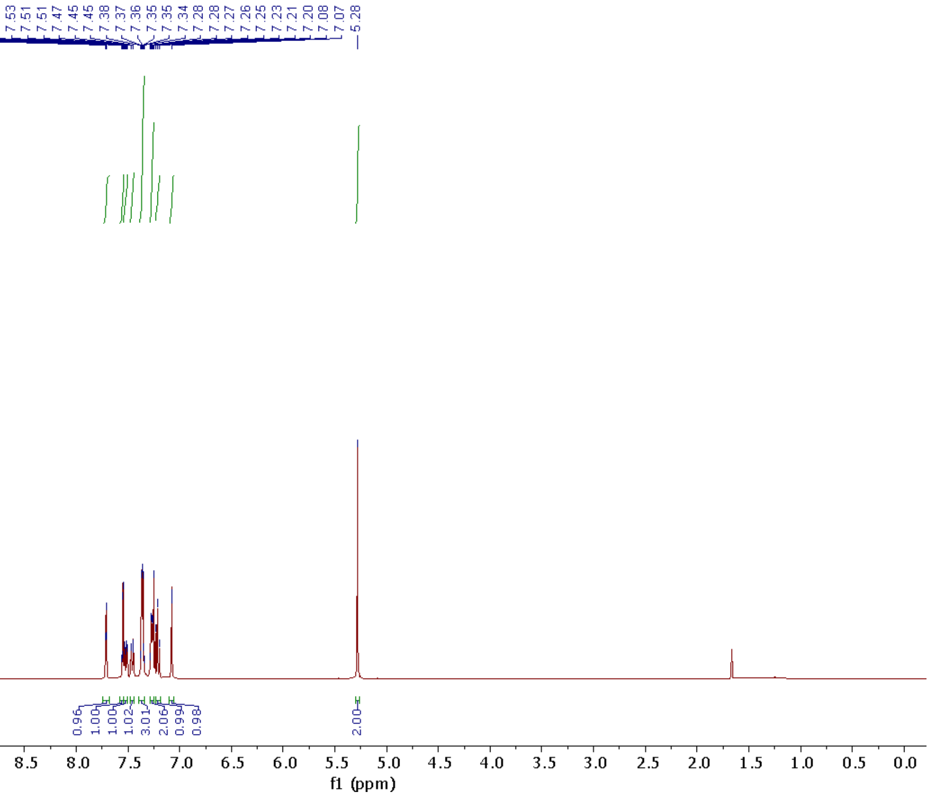

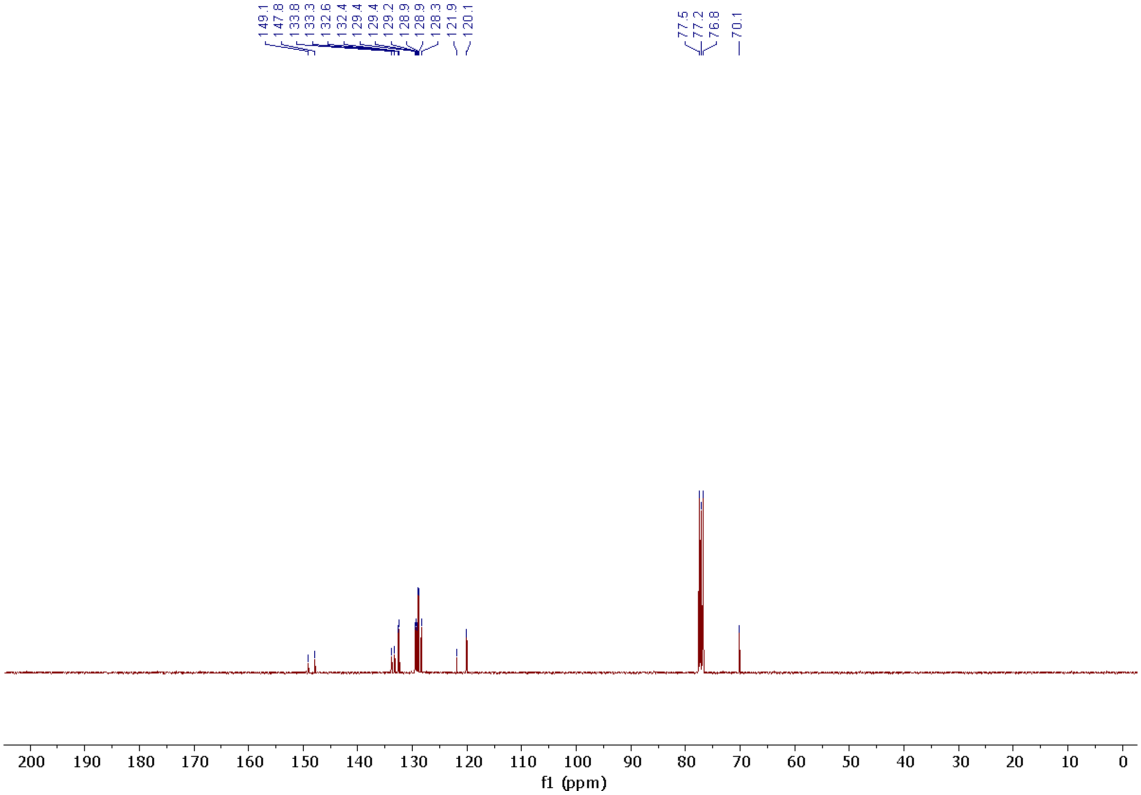

400 MHz ^{1}H NMR spectrum; 100.6 MHz ^{13}C NMR spectrum; $CDCl_3$ of **16f**

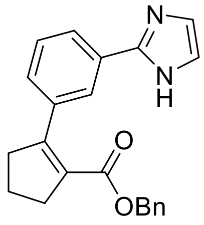

16f

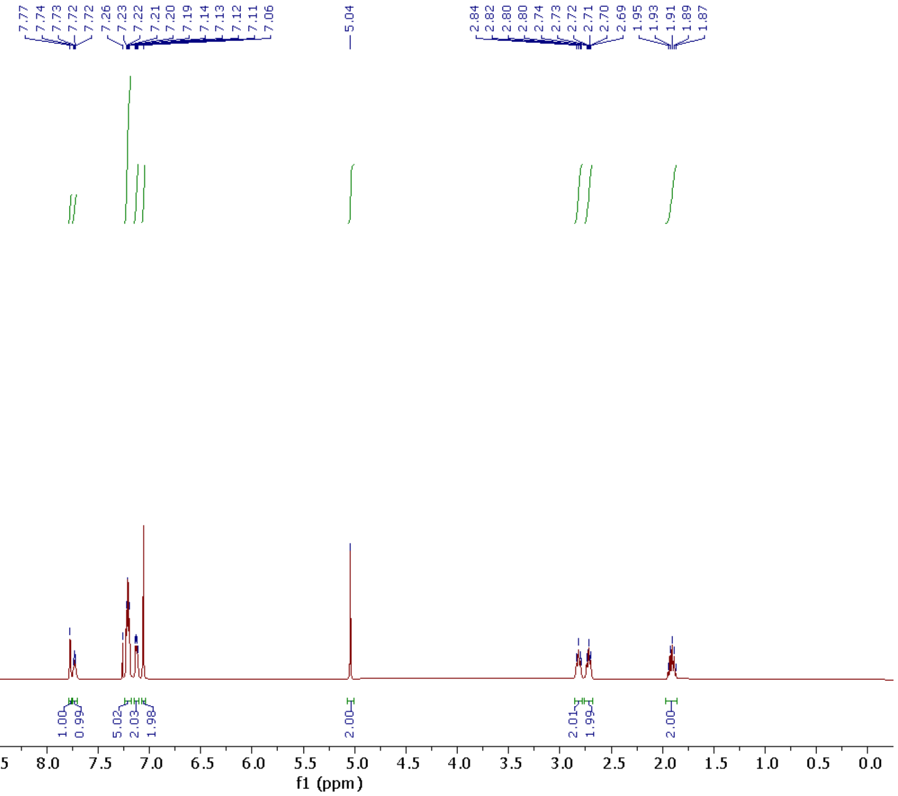

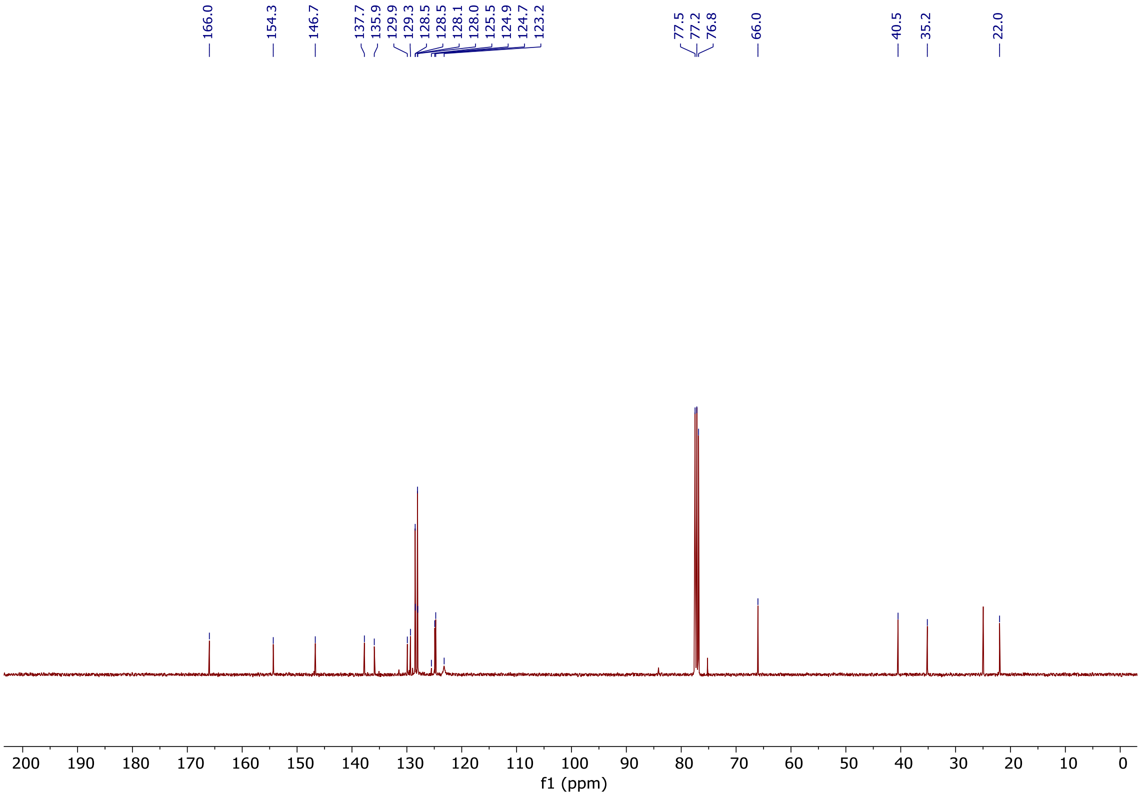

400 MHz ^{1}H NMR spectrum; 100.6 MHz ^{13}C NMR spectrum; $CDCl_3$ of **16g**

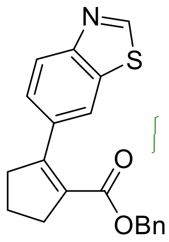

16g

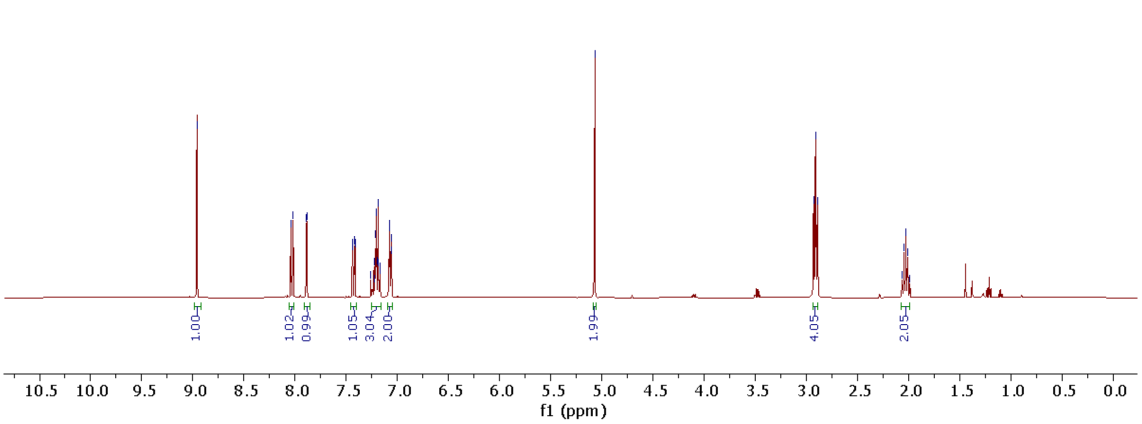

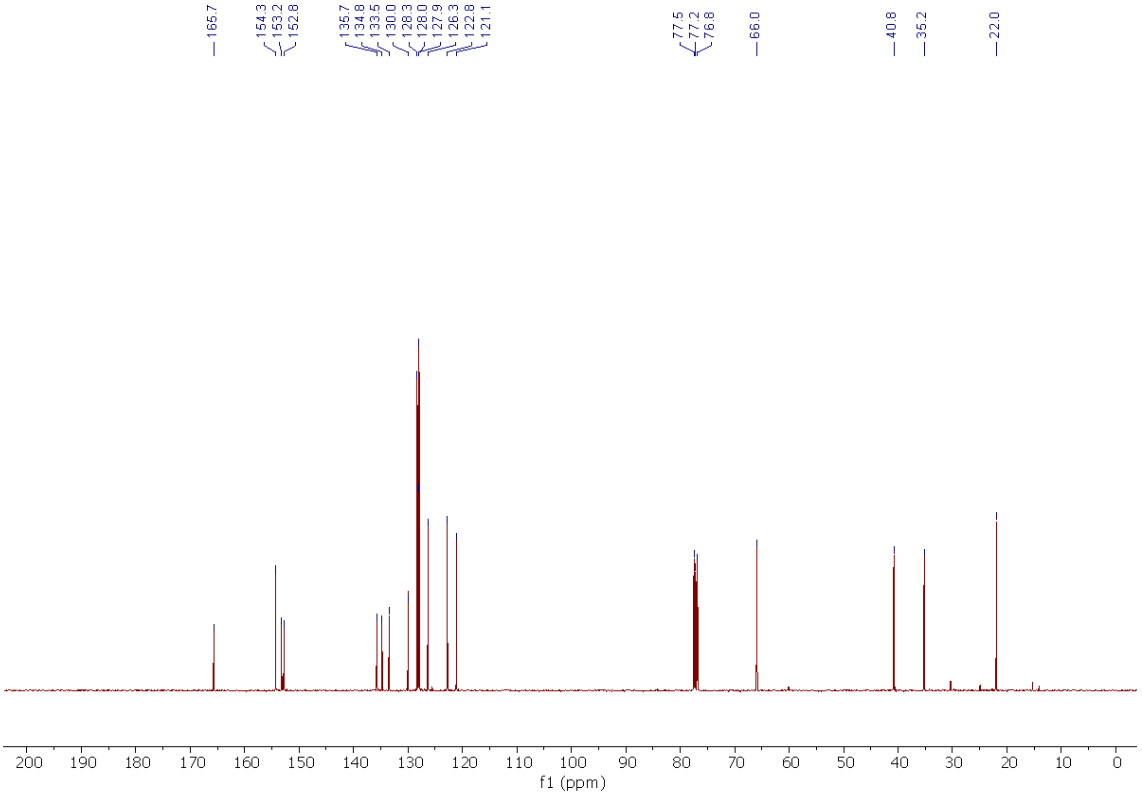

400 MHz ^{1}H NMR spectrum; 100.6 MHz ^{13}C NMR spectrum; $CDCl_3$ of **16h**

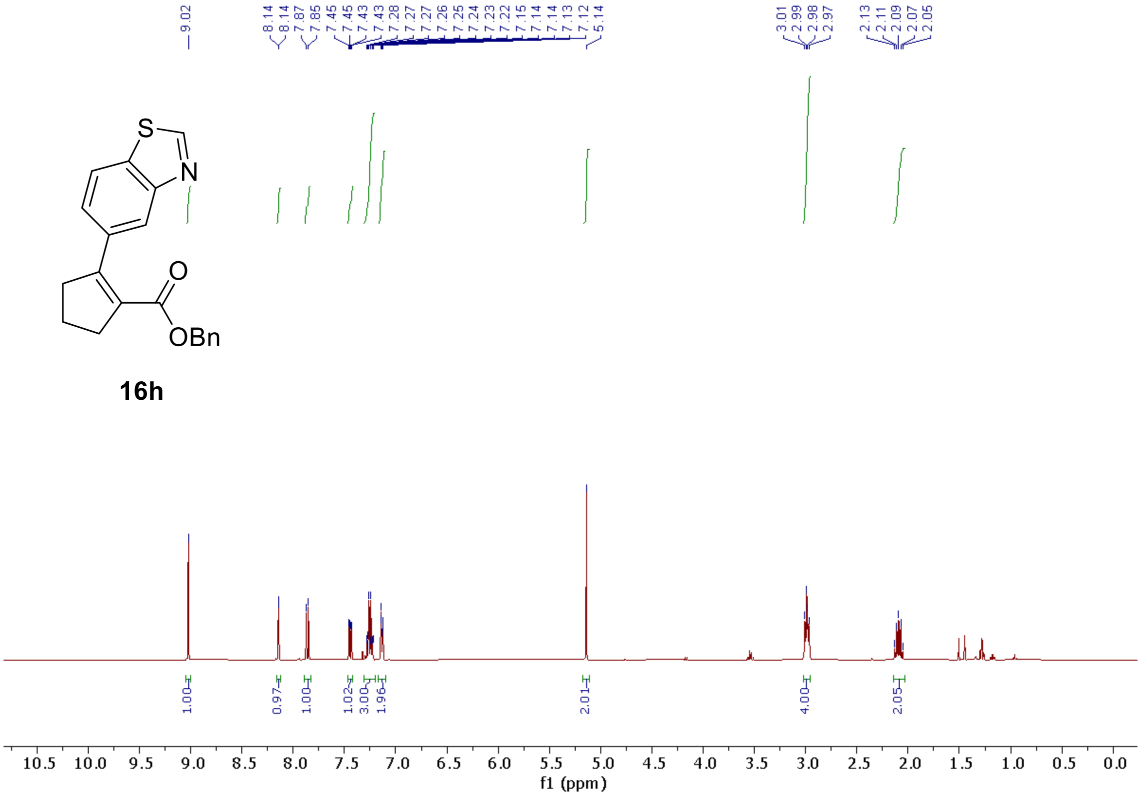

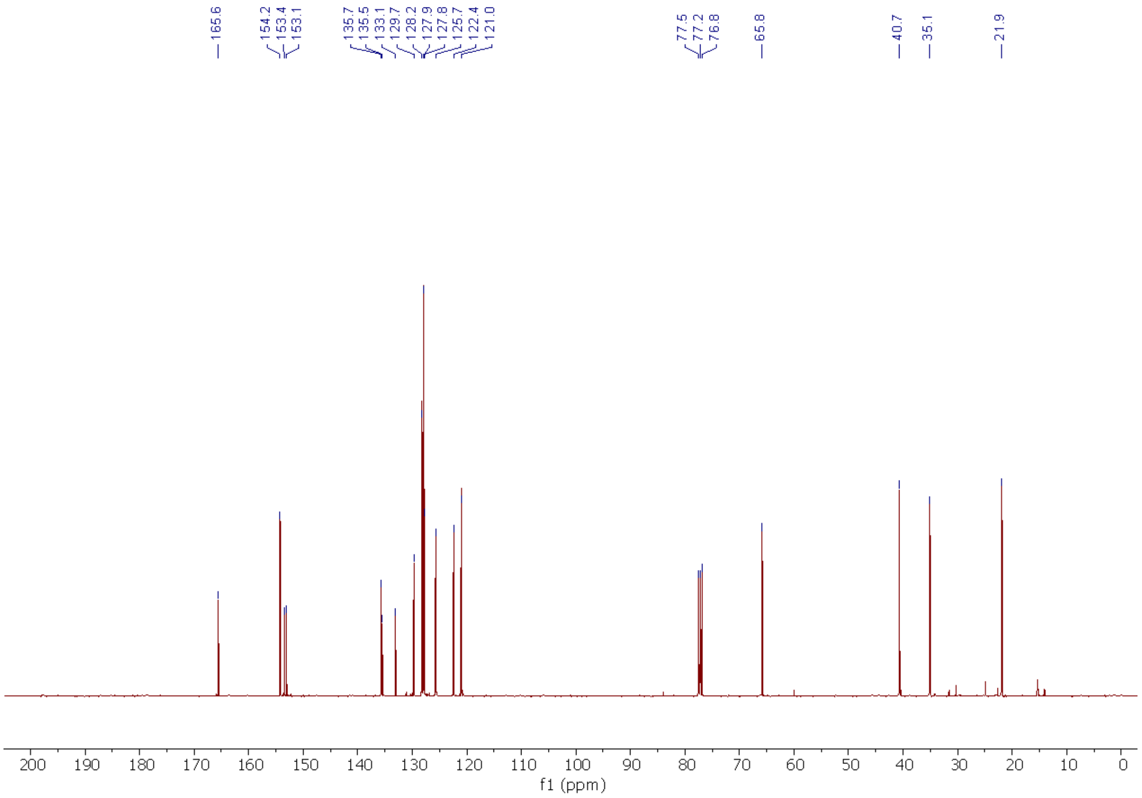

400 MHz ^{1}H NMR spectrum; 100.6 MHz ^{13}C NMR spectrum; $CDCl_3$ of **16i**

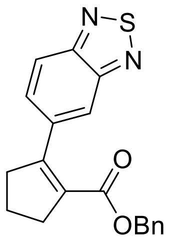

16i

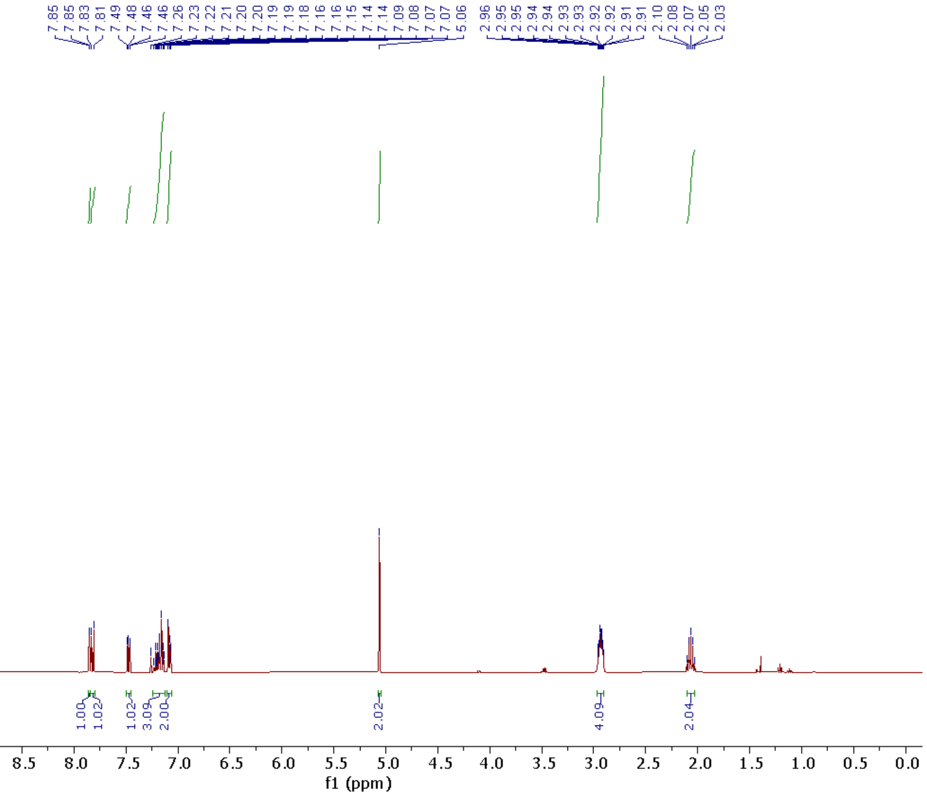

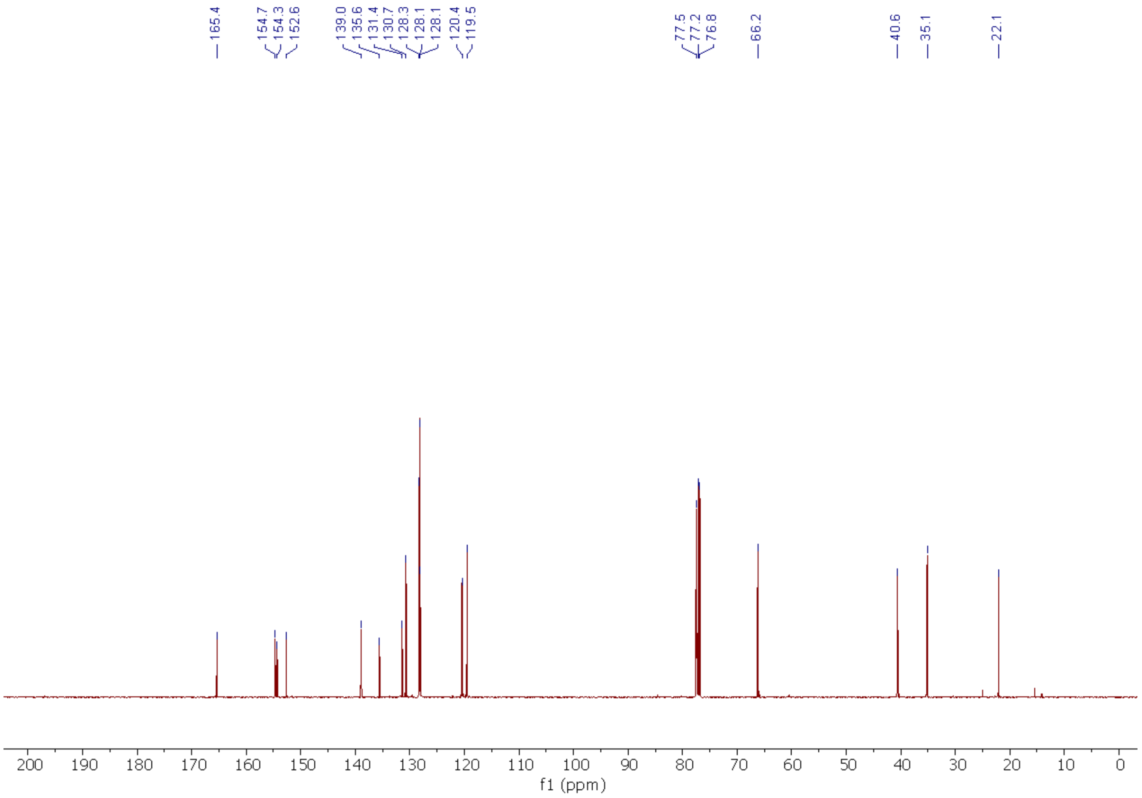

400 MHz ^{1}H NMR spectrum; 100.6 MHz ^{13}C NMR spectrum; CD_3OD of *cis*-**17a**

cis-**17a**

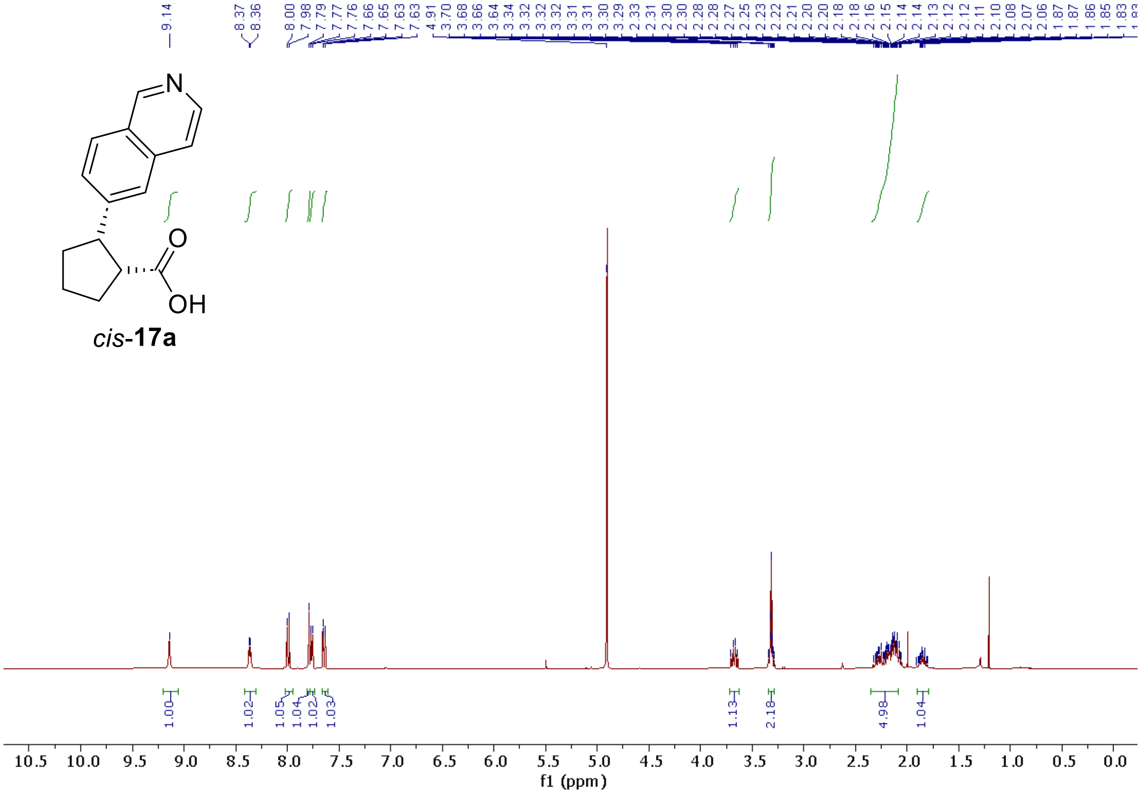

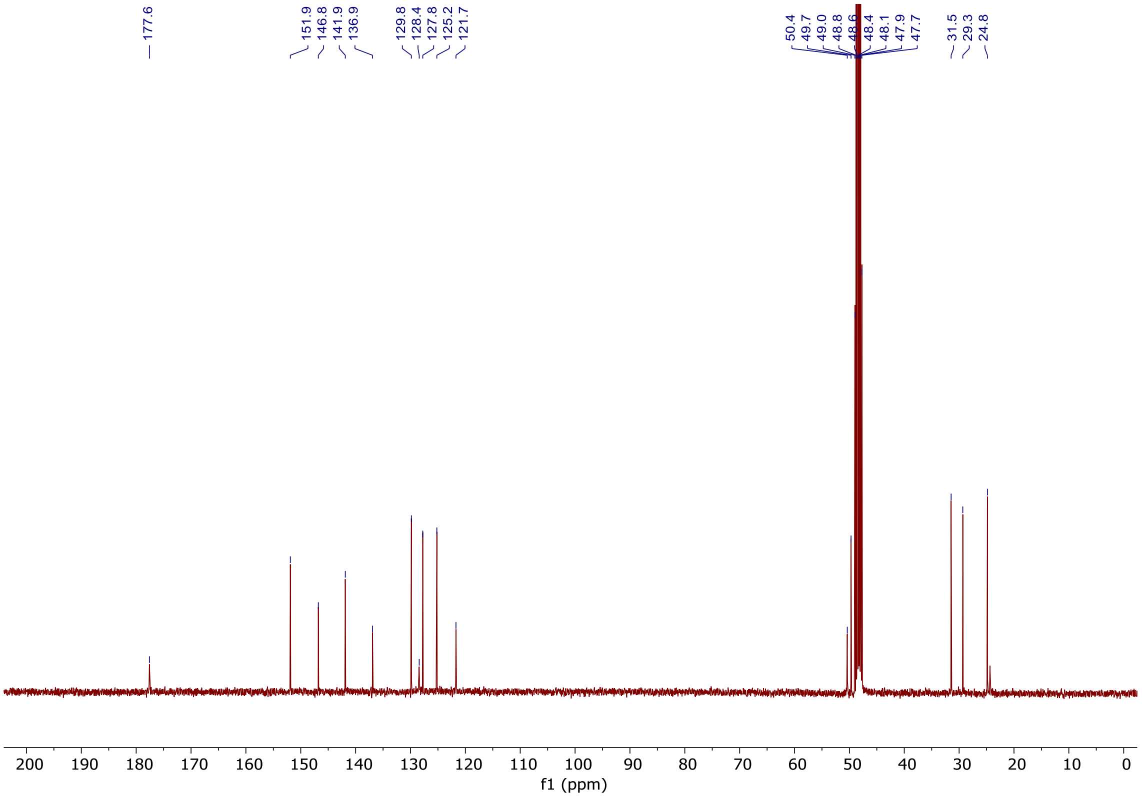

400 MHz ^{1}H NMR spectrum; 100.6 MHz ^{13}C NMR spectrum; CD_3OD of *cis*-**17b**

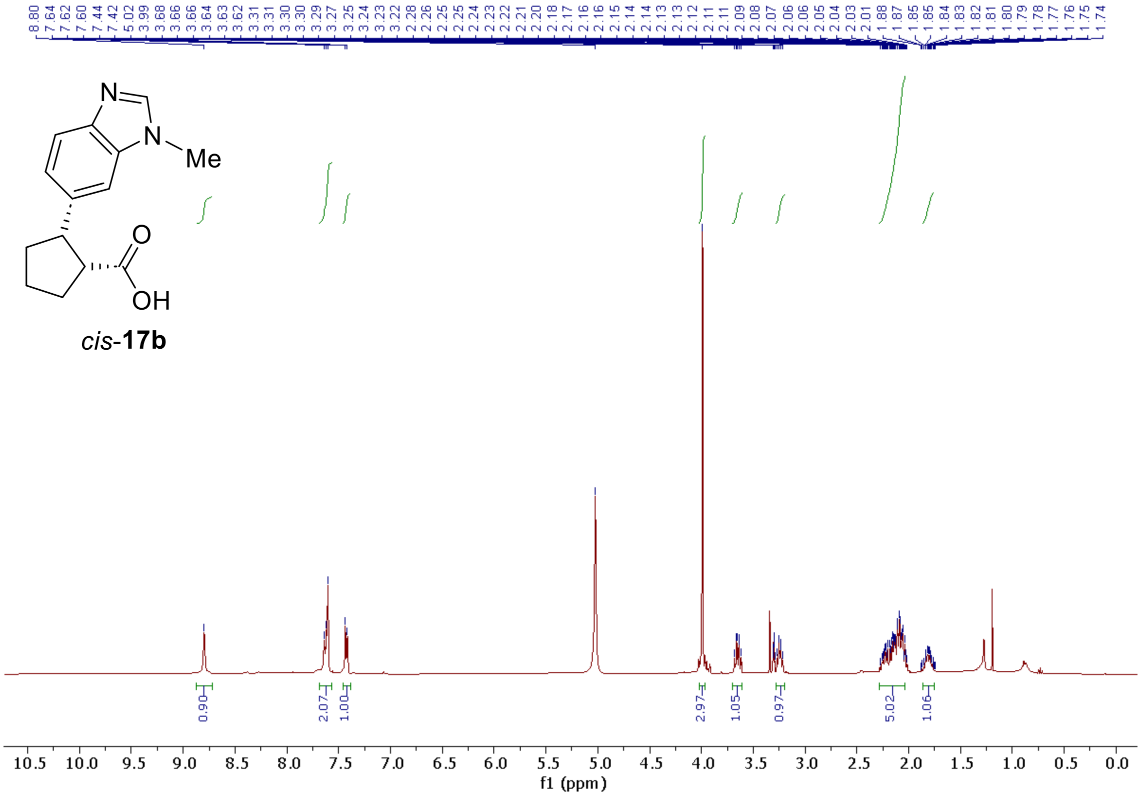

cis-**17b**

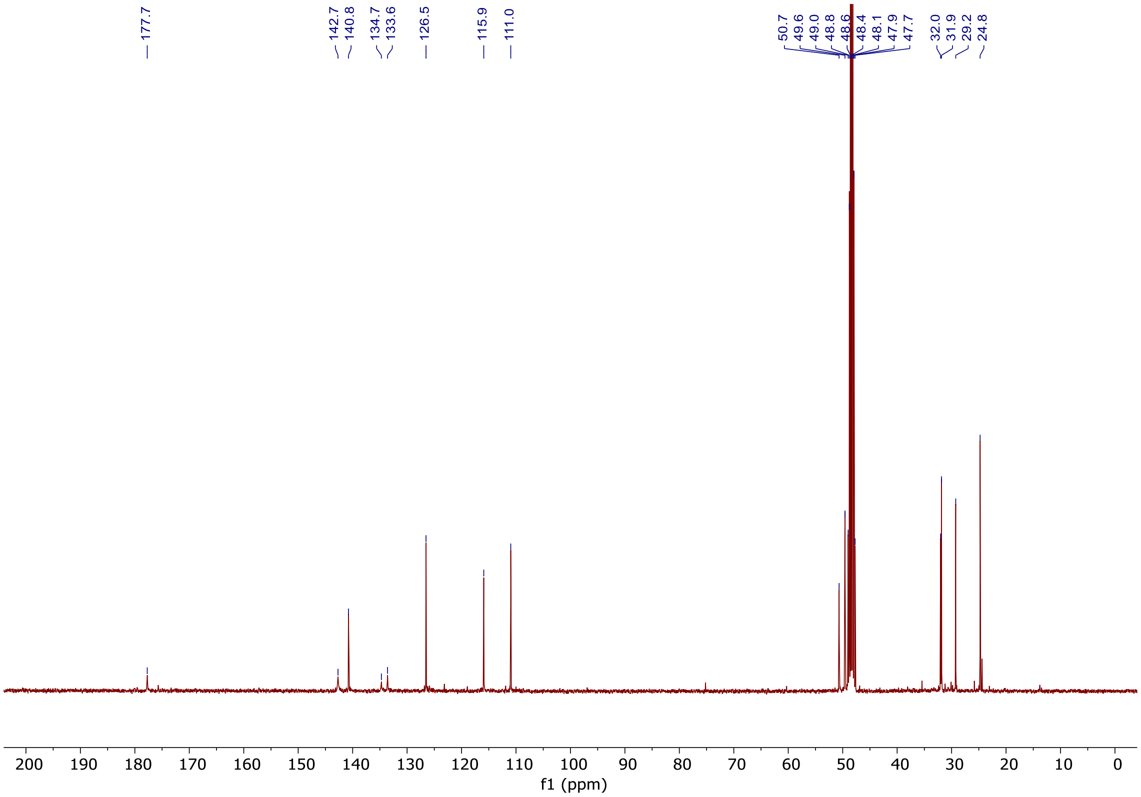

400 MHz ^{1}H NMR spectrum; 100.6 MHz ^{13}C NMR spectrum; CD_3OD of *cis*-**17c**

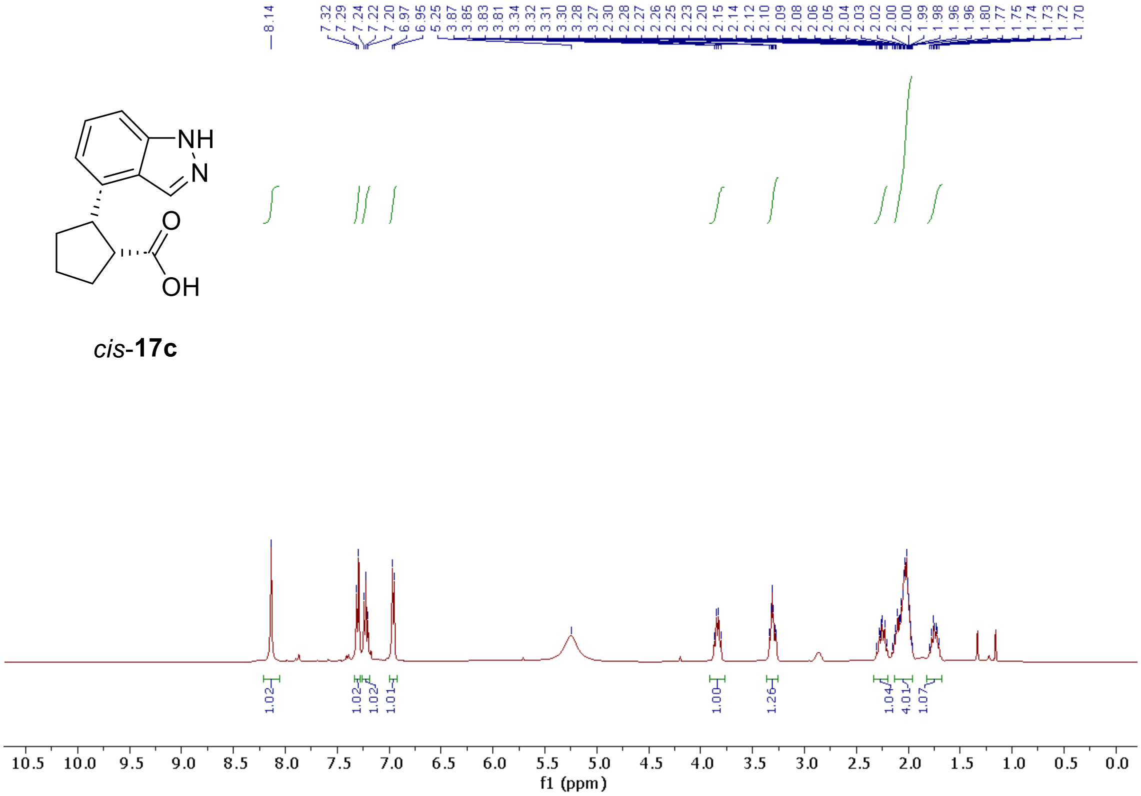

cis-**17c**

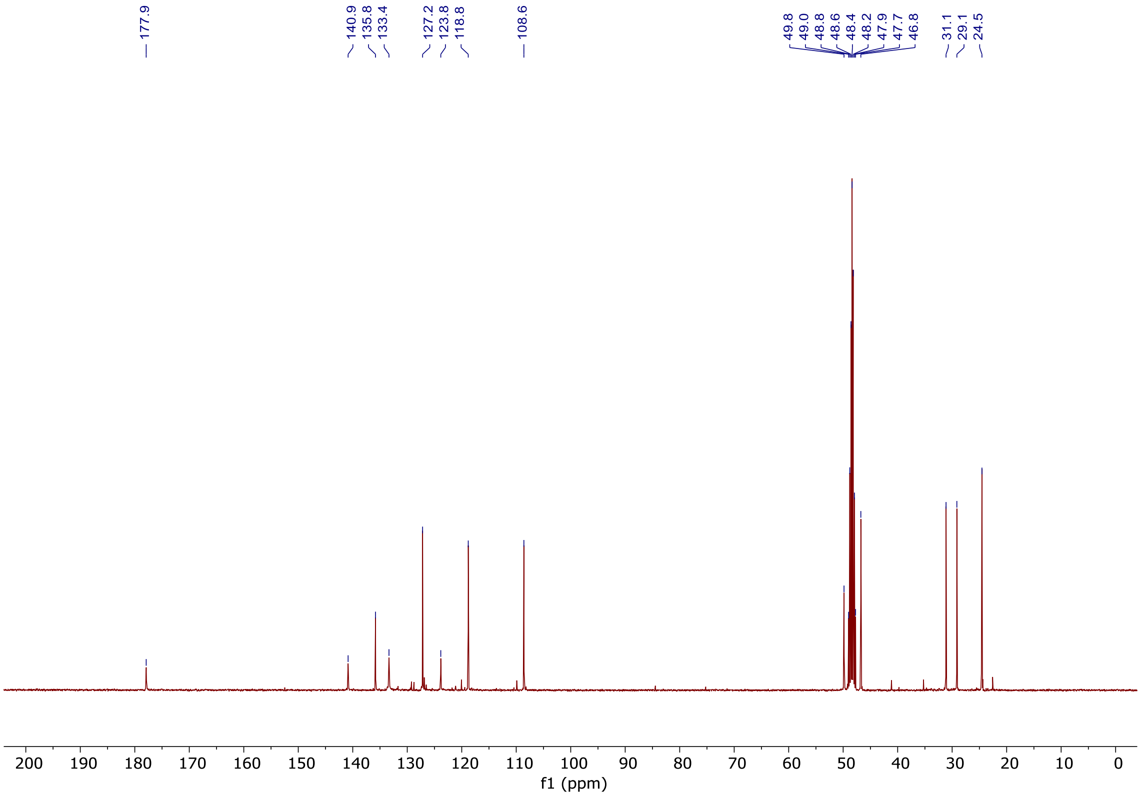

400 MHz ^{1}H NMR spectrum; 100.6 MHz ^{13}C NMR spectrum; CD_3OD of *cis*-**17d**

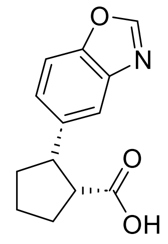

cis-**17d**

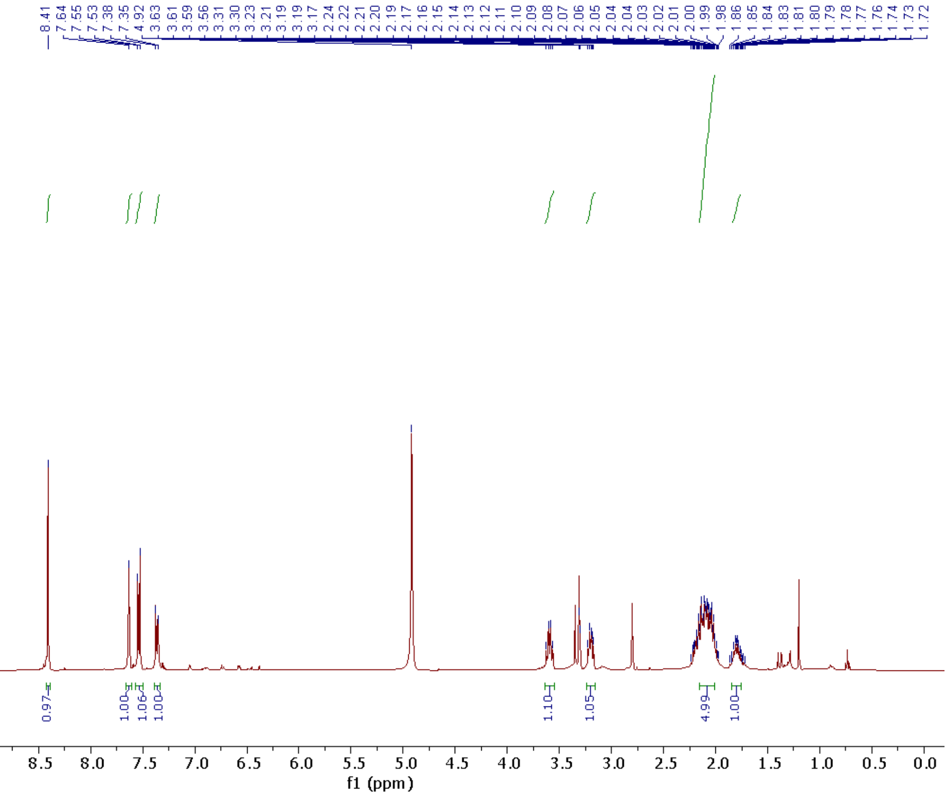

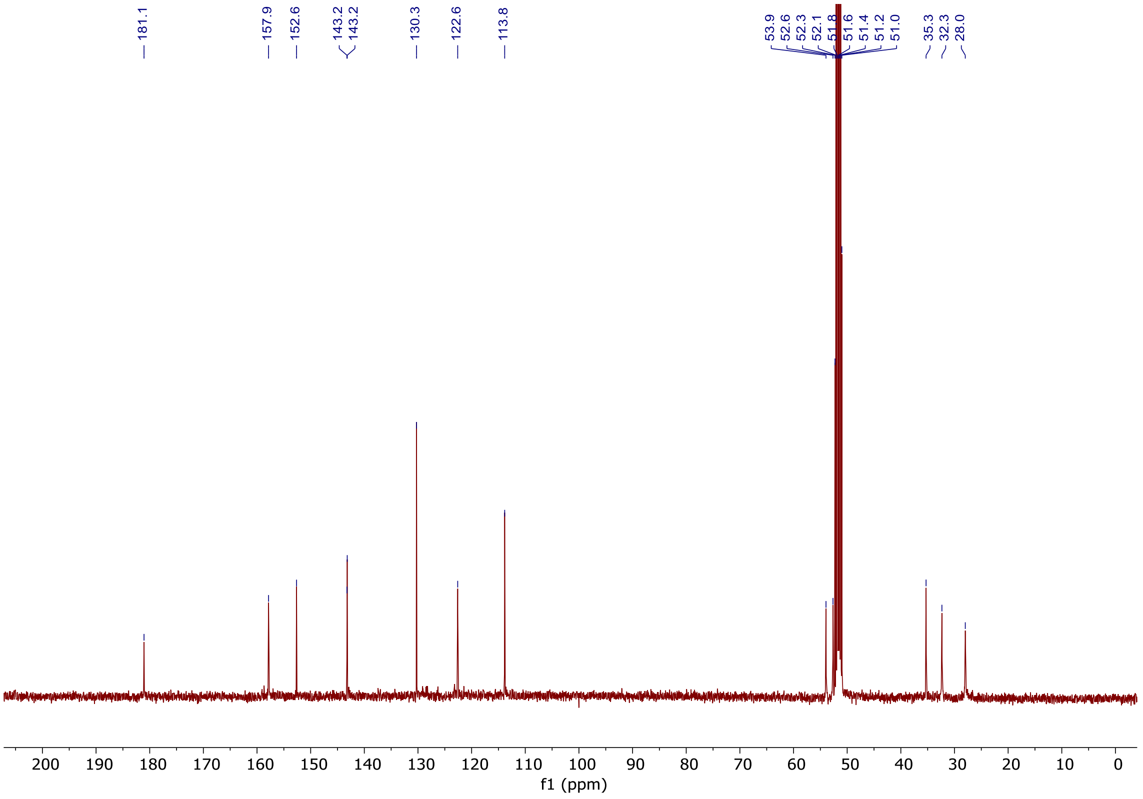

400 MHz ^{1}H NMR spectrum; 100.6 MHz ^{13}C NMR spectrum; 378.5 MHz ^{19}F NMR spectrum; $CDCl_3$ of *cis*-**17e**

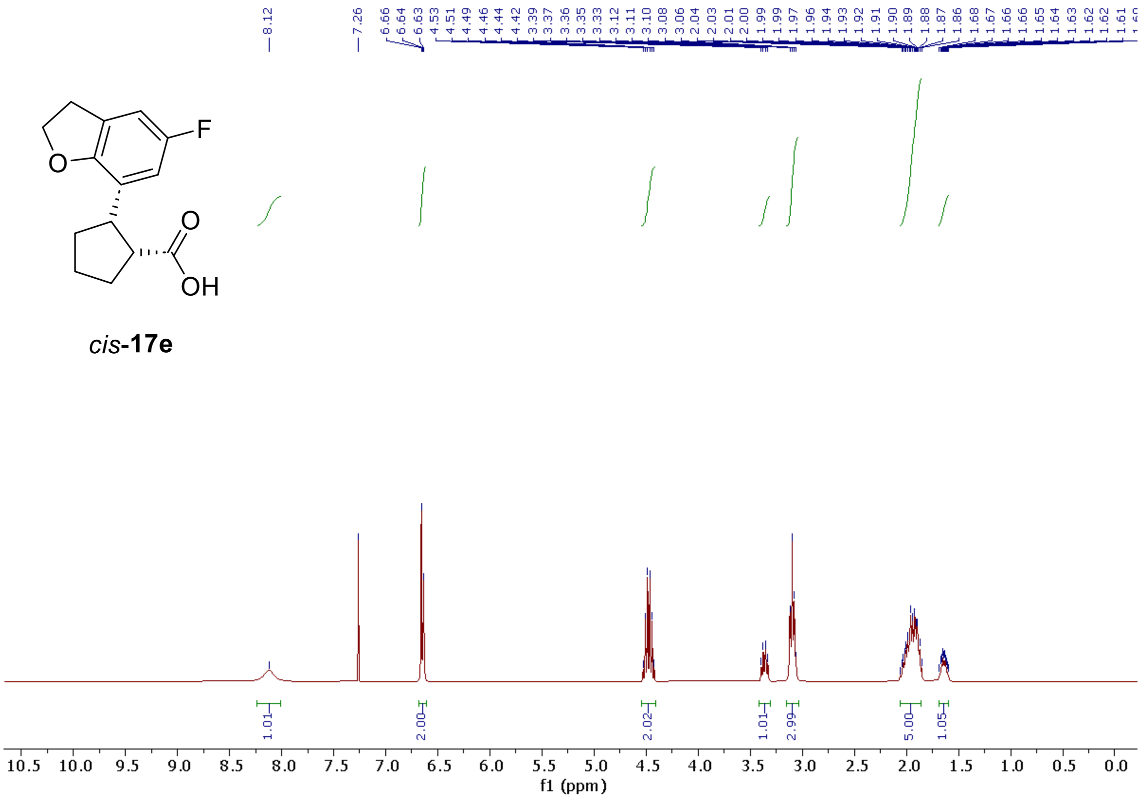

cis-**17e**

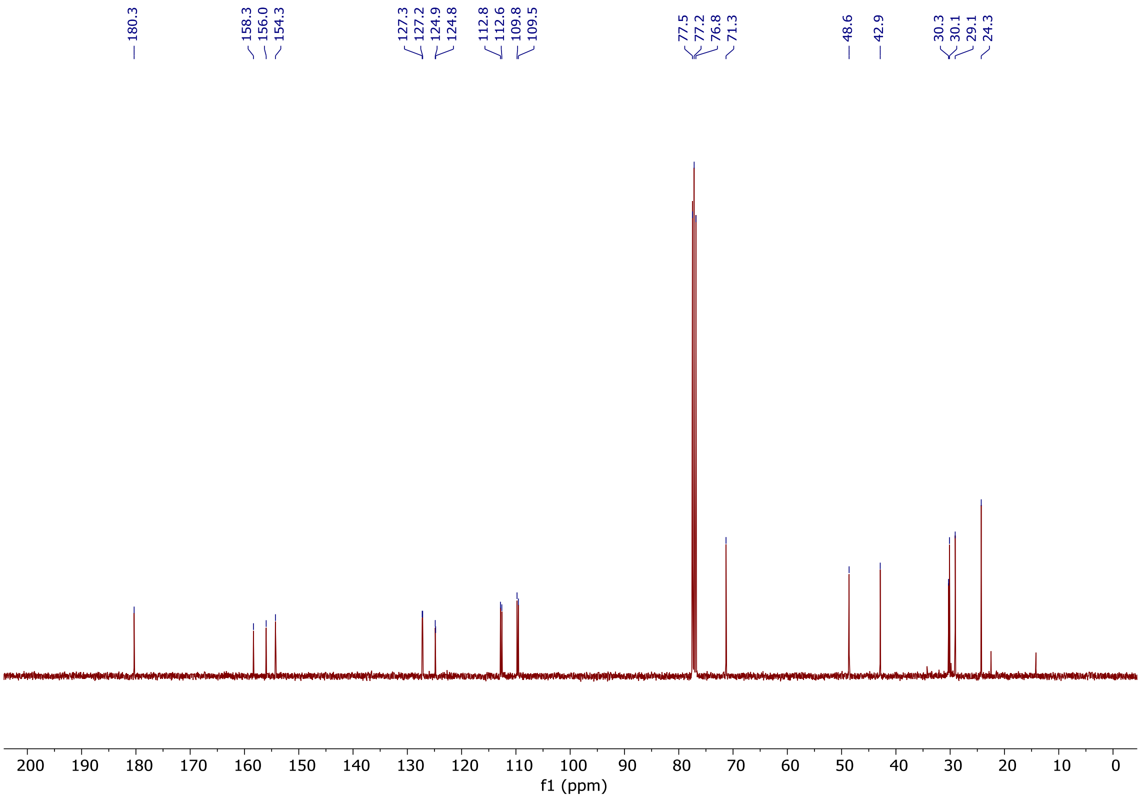

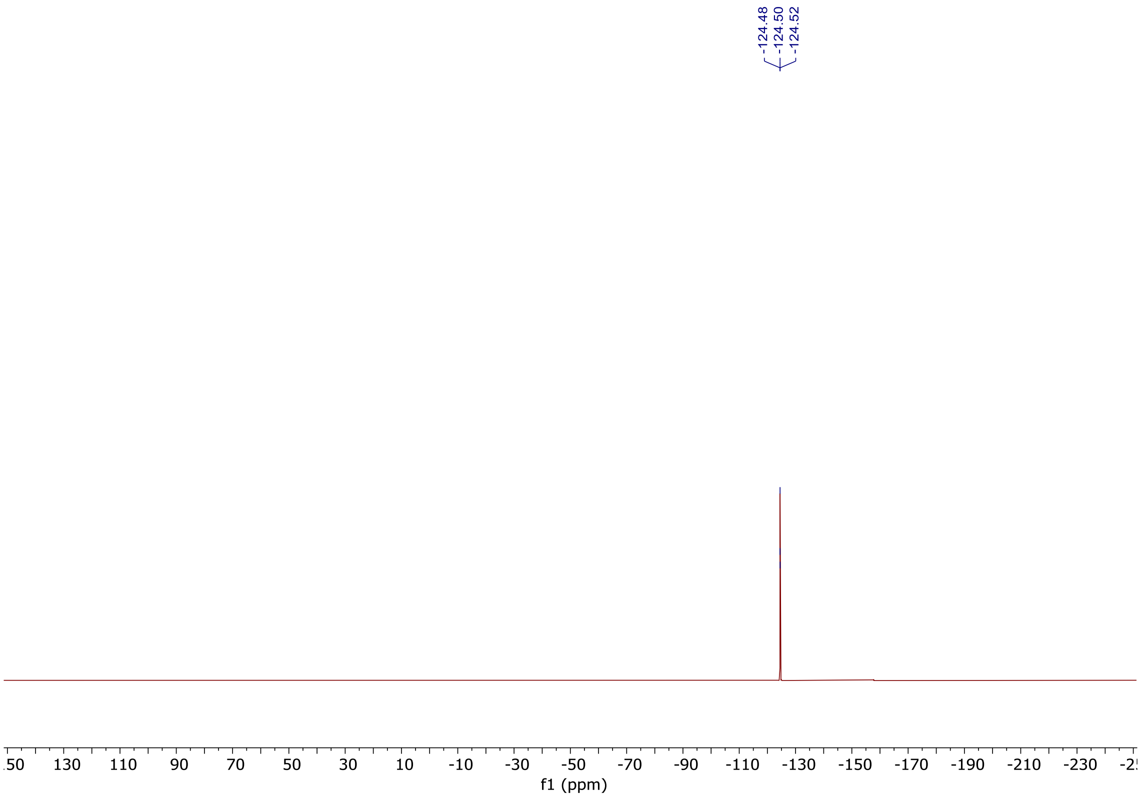
-124.48
-124.50
-124.52
.50 130 110 90 70 50 30 10 -10 -30 -50 -70 -90 -110 -130 -150 -170 -190 -210 -230
f1 (ppm)

400 MHz ^{1}H NMR spectrum; 100.6 MHz ^{13}C NMR spectrum; CD_3OD of *cis*-**17f**

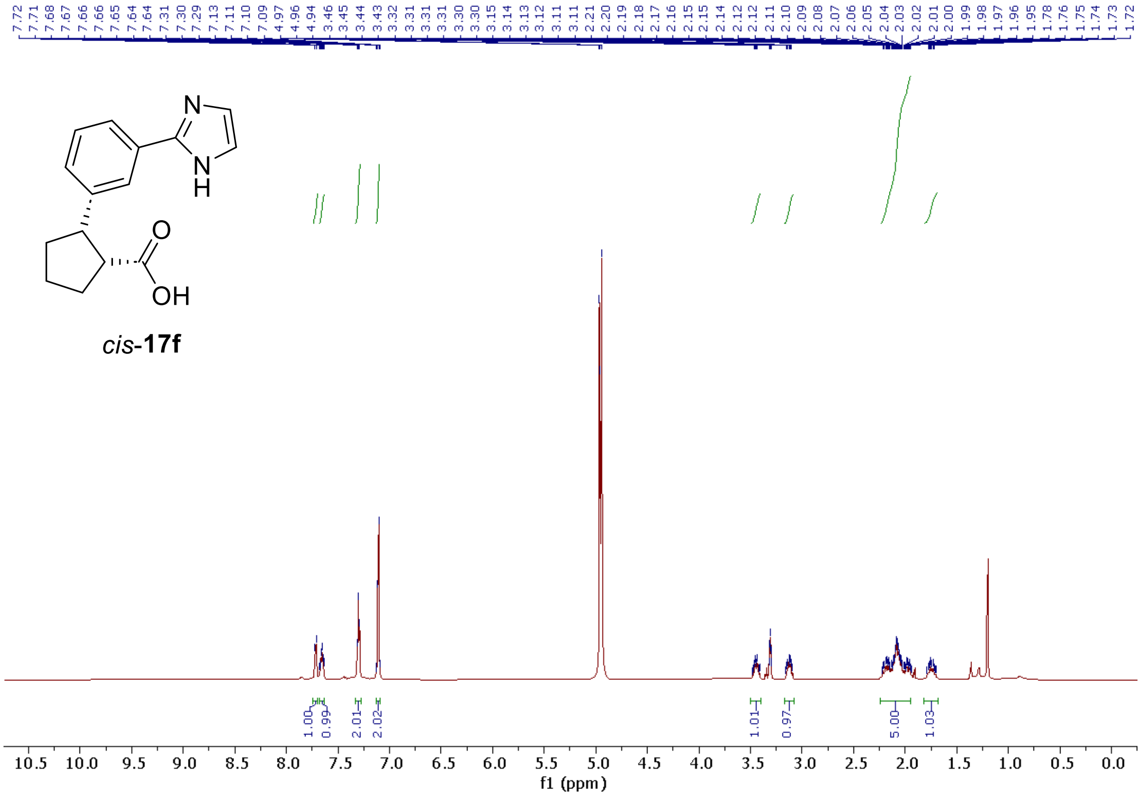

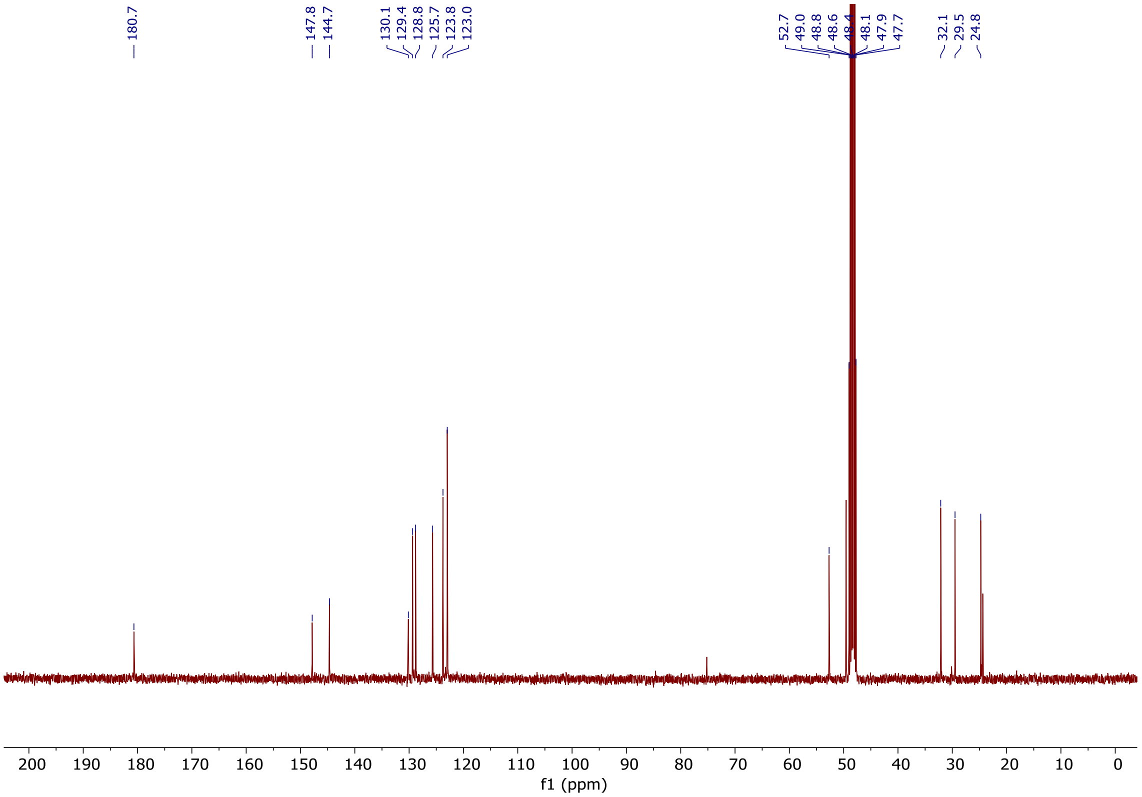

400 MHz ^{1}H NMR spectrum; 100.6 MHz ^{13}C NMR spectrum; 378.5 MHz ^{19}F NMR spectrum; $CDCl_3$ of **18**

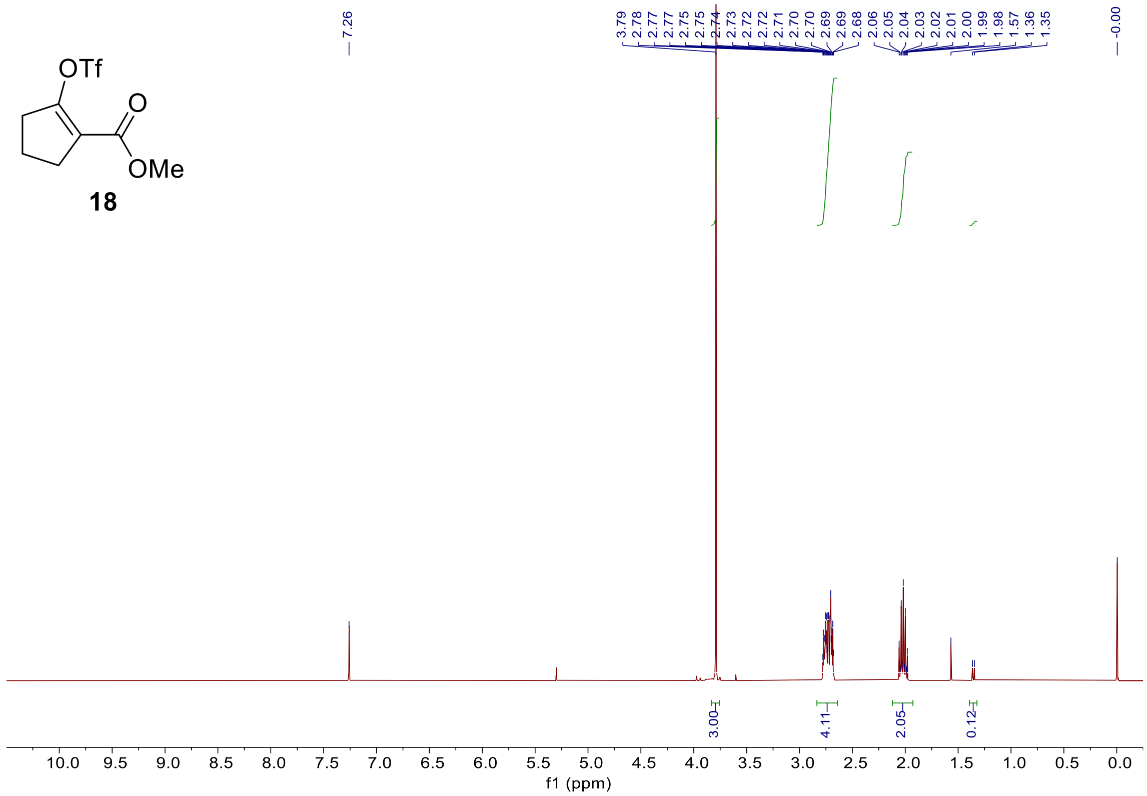

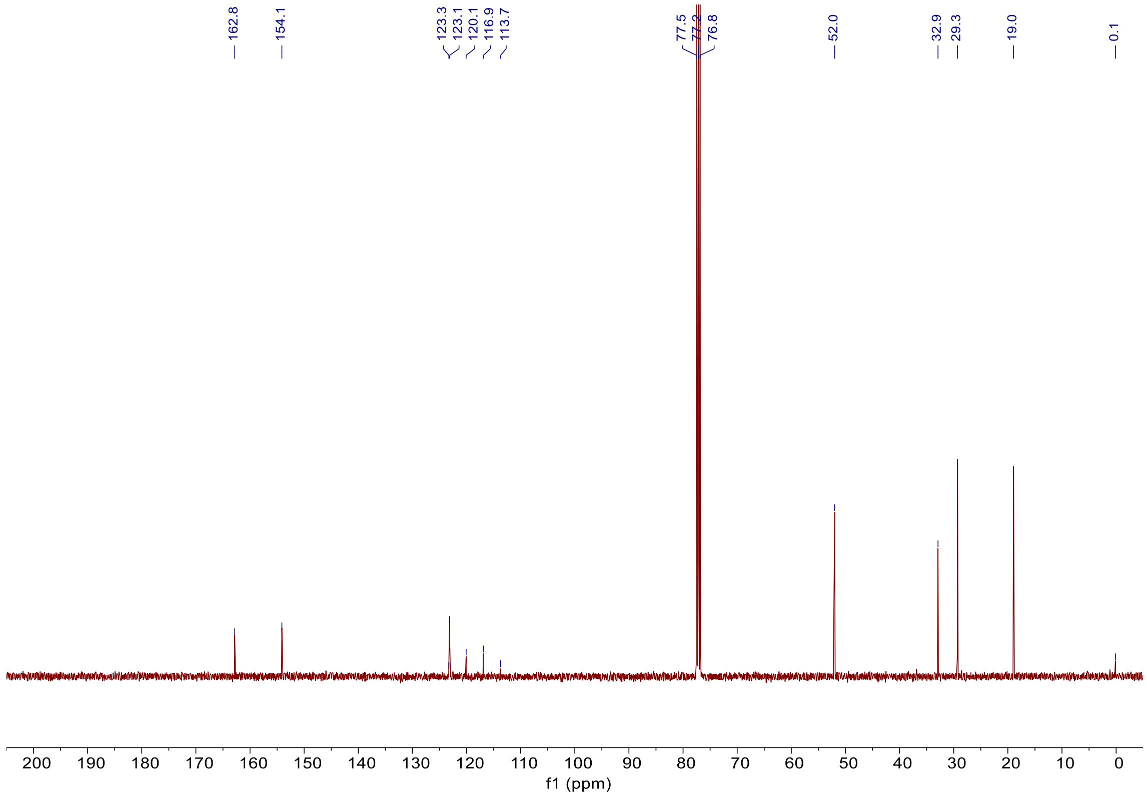

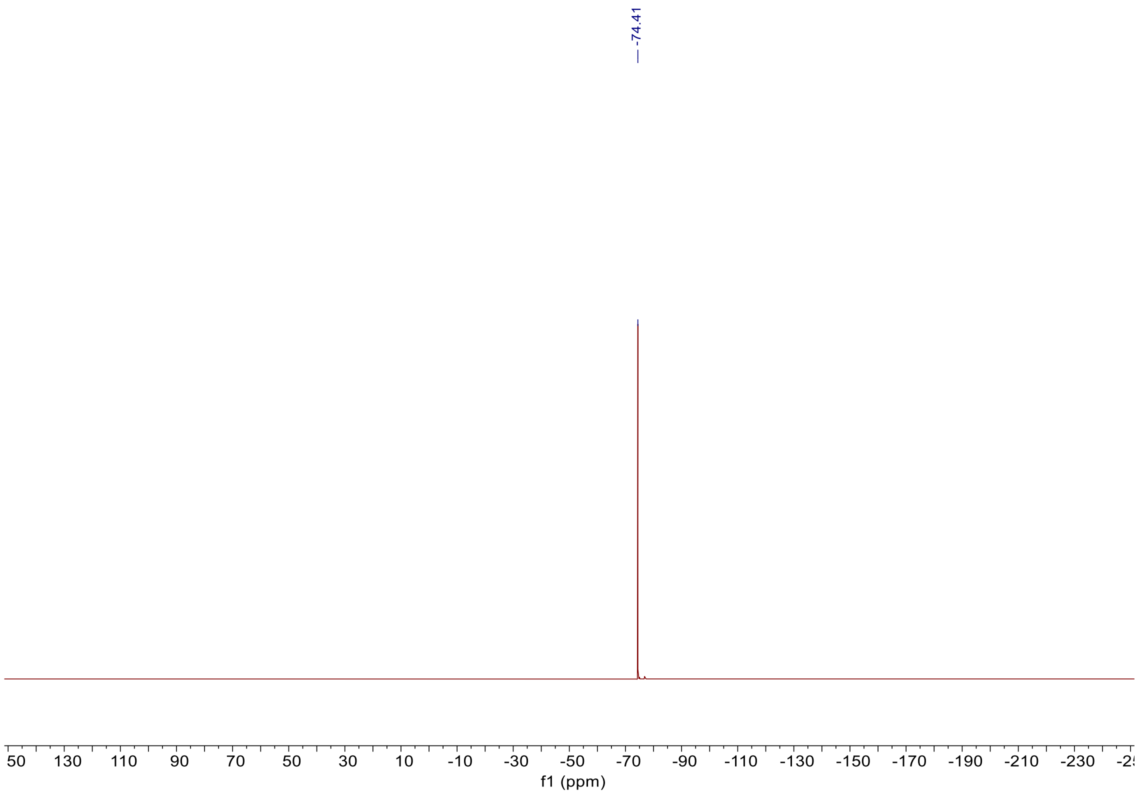
-74.41
50
130
110
90
70
50
30
10
-10
-30
-50
-70
-90
-110
-130
-150
-170
-190
-210
-230
f1 (ppm)

400 MHz ^{1}H NMR spectrum; 100.6 MHz ^{13}C NMR spectrum; $CDCl_3$ of **19a**

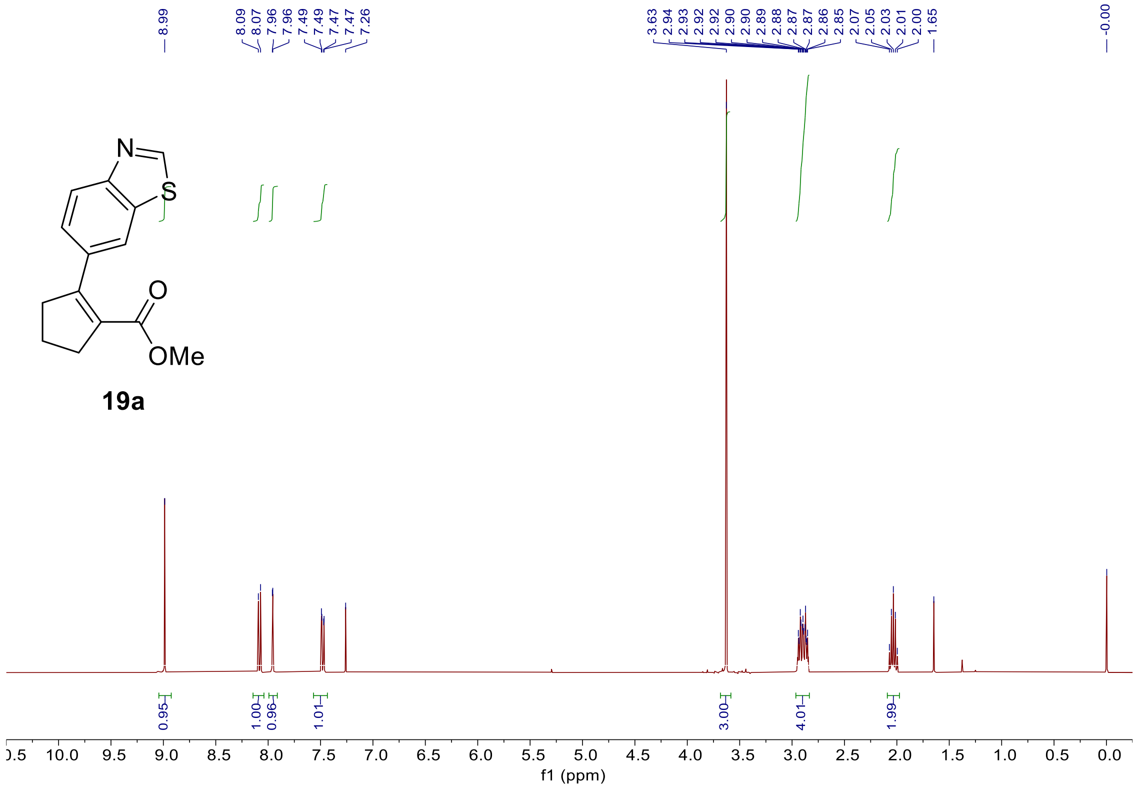

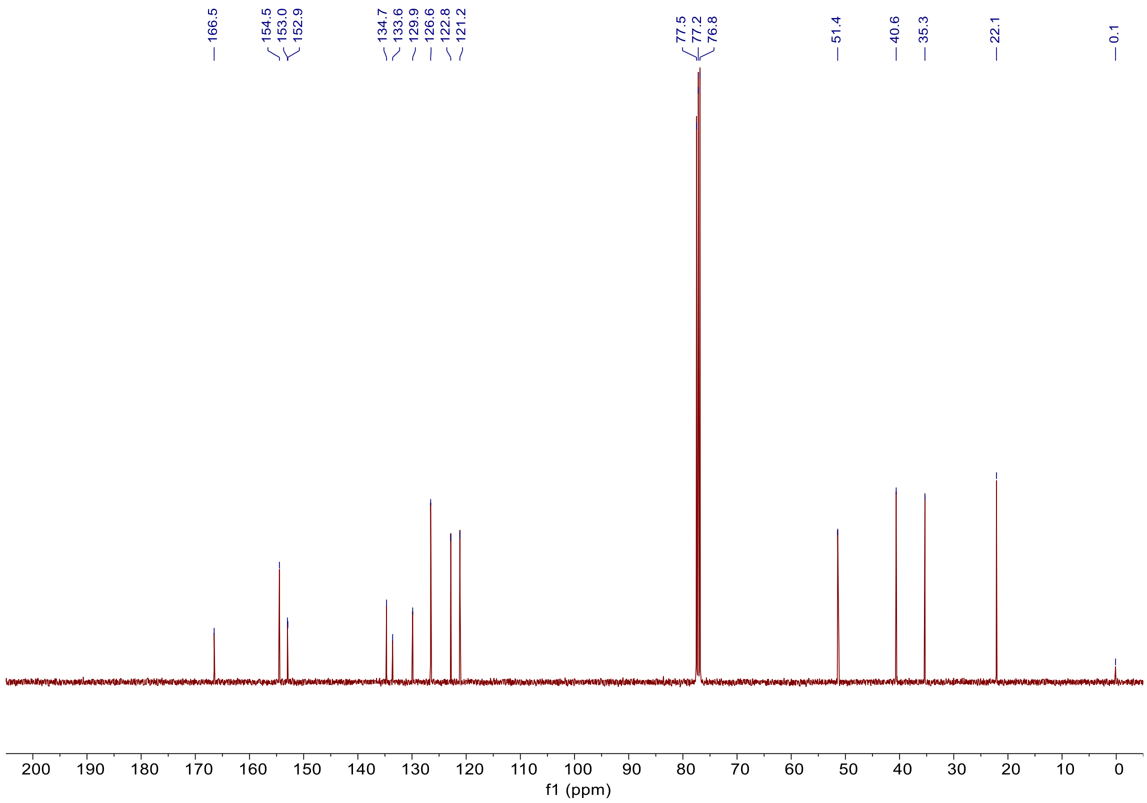

400 MHz ^{1}H NMR spectrum; 100.6 MHz ^{13}C NMR spectrum; $CDCl_3$ of **19b**

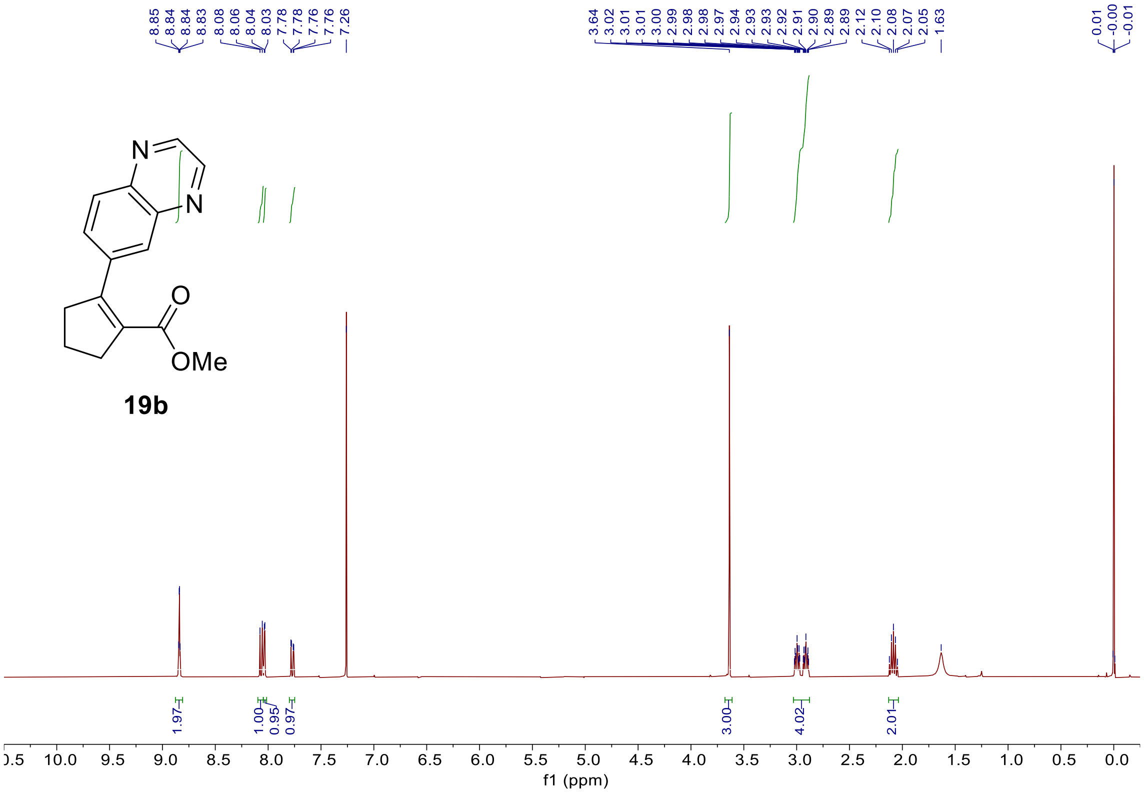

19b

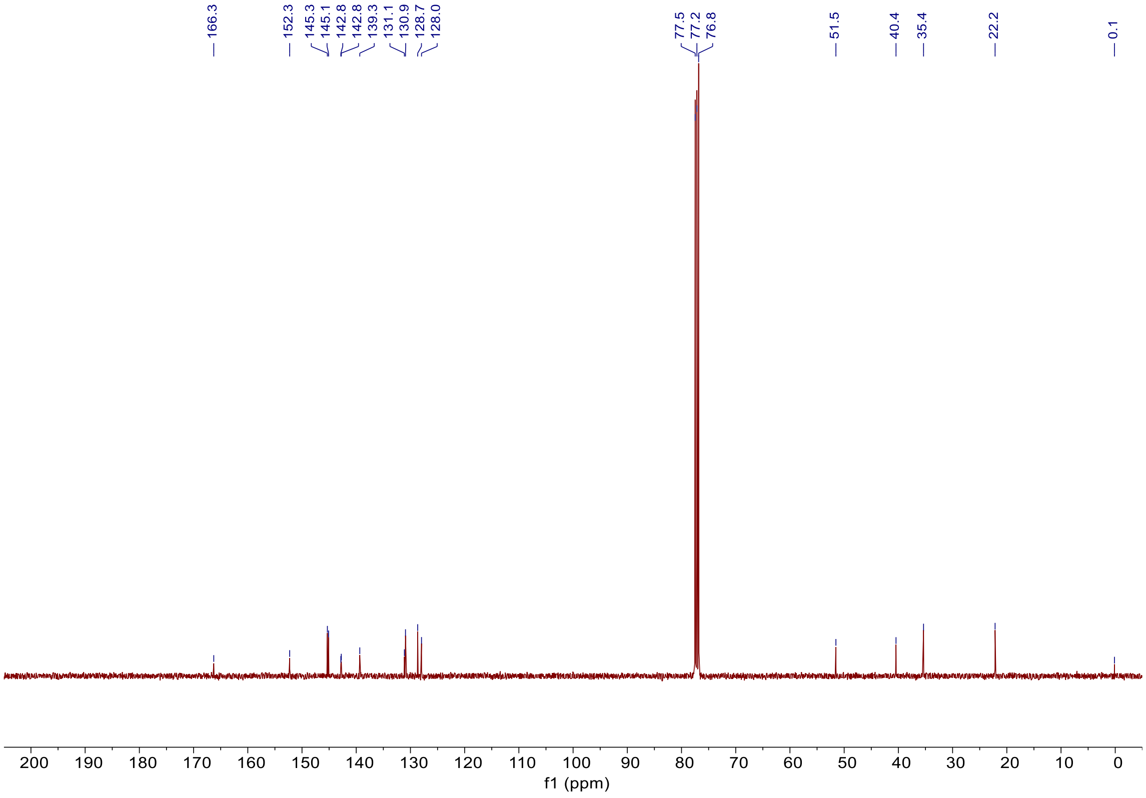

400 MHz ^{1}H NMR spectrum; 100.6 MHz ^{13}C NMR spectrum; $CDCl_3$ of **19c**

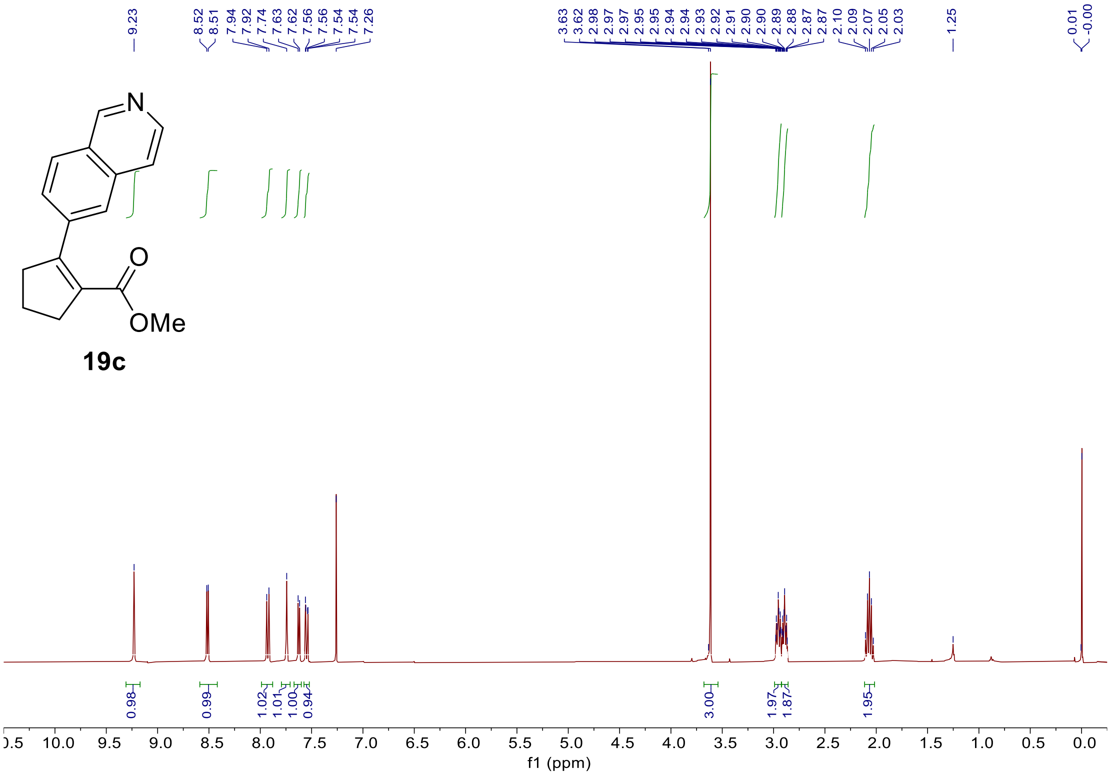

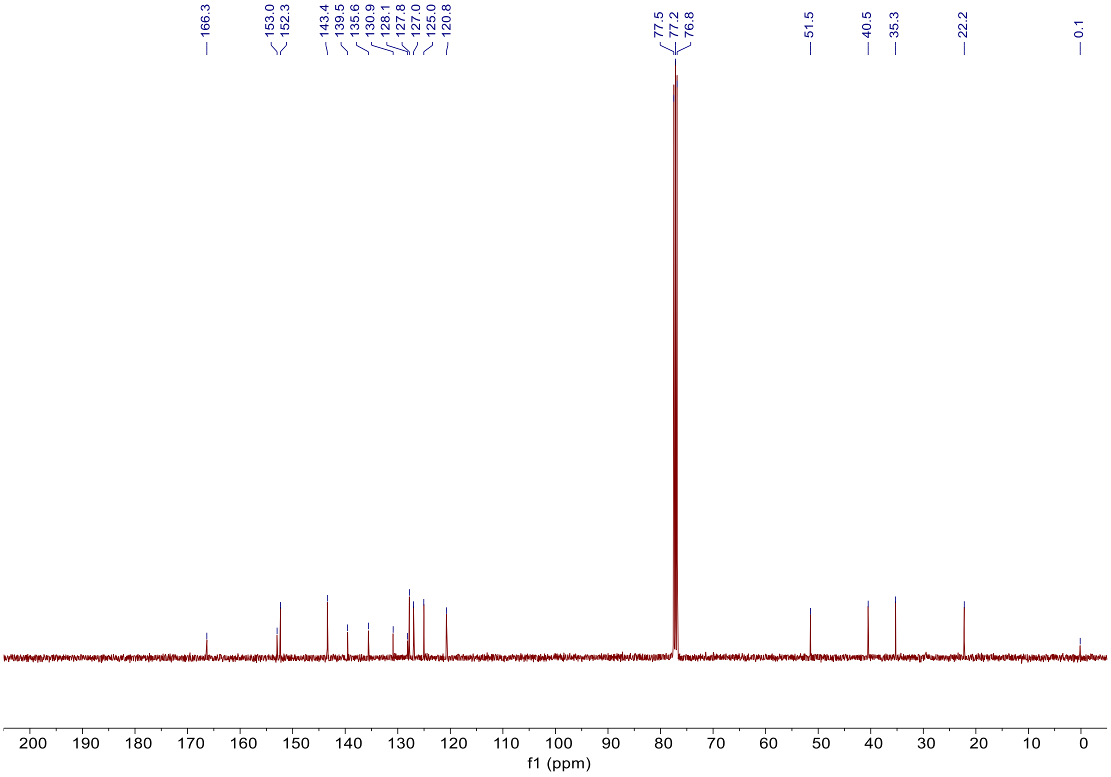

400 MHz ^{1}H NMR spectrum; 100.6 MHz ^{13}C NMR spectrum; $CDCl_3$ of **19d**

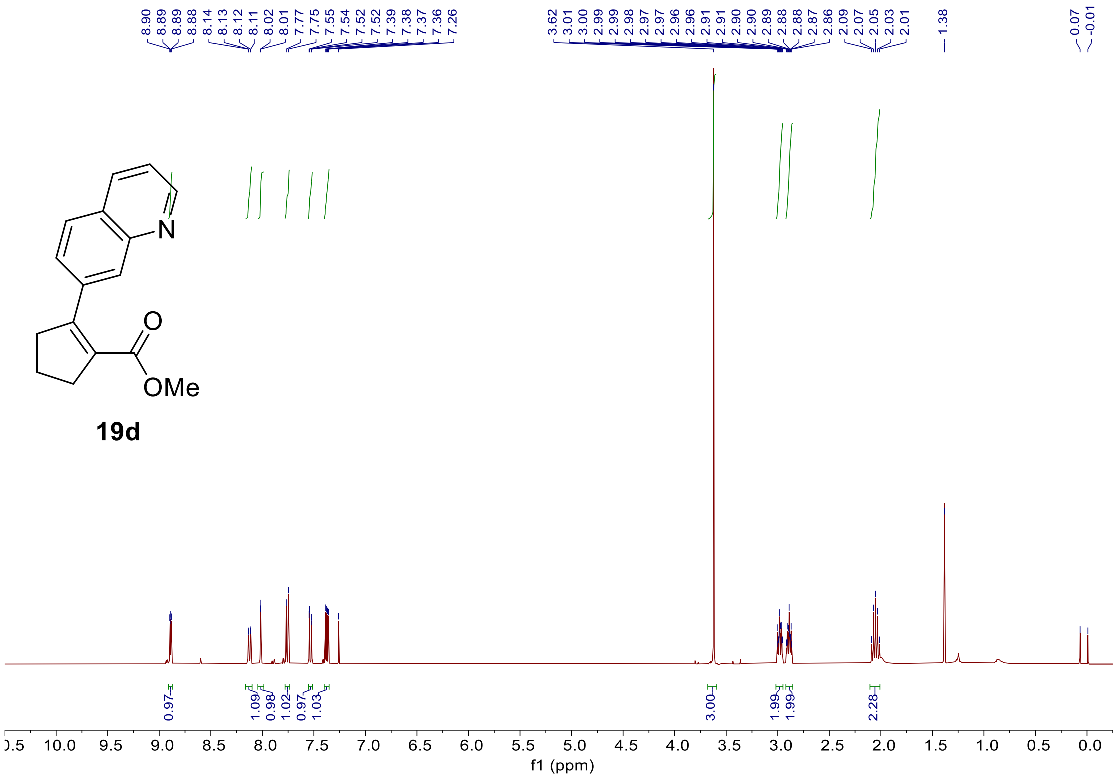

19d

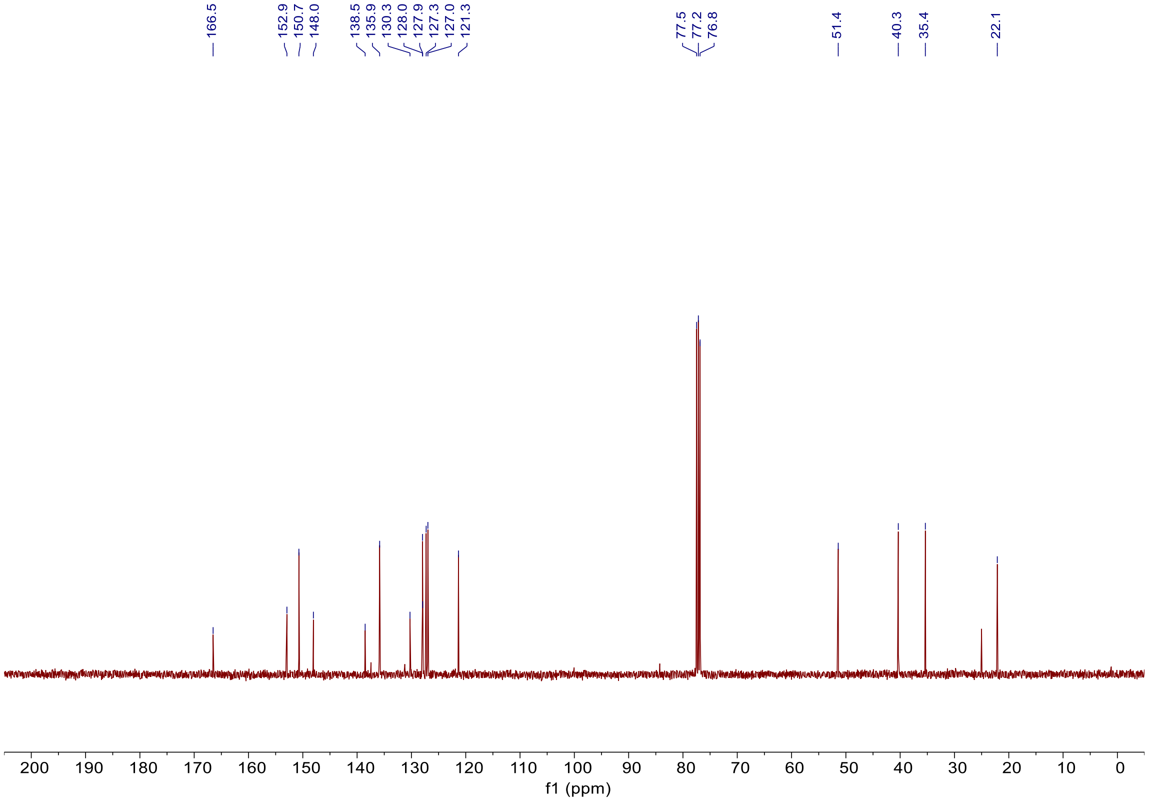

400 MHz ^{1}H NMR spectrum; 100.6 MHz ^{13}C NMR spectrum; $CDCl_3$ of **19e**

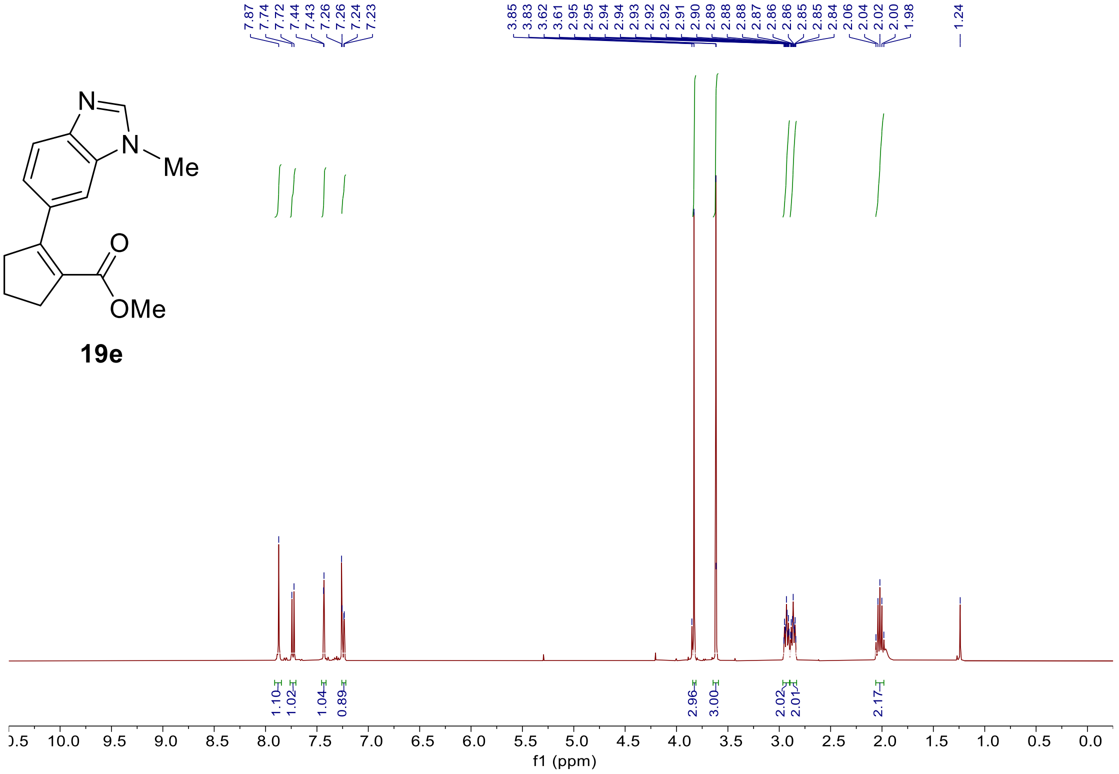

19e

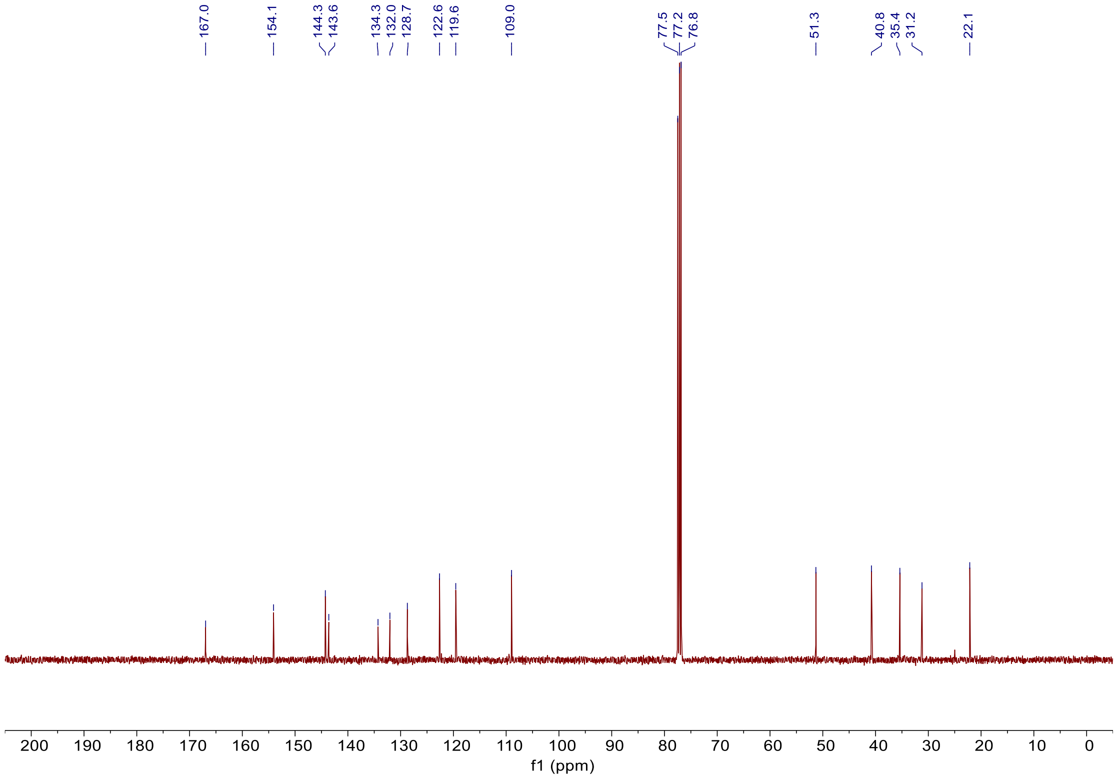

400 MHz ^{1}H NMR spectrum; 100.6 MHz ^{13}C NMR spectrum; $CDCl_3$ of **19f**

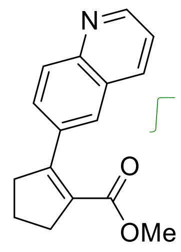

19f

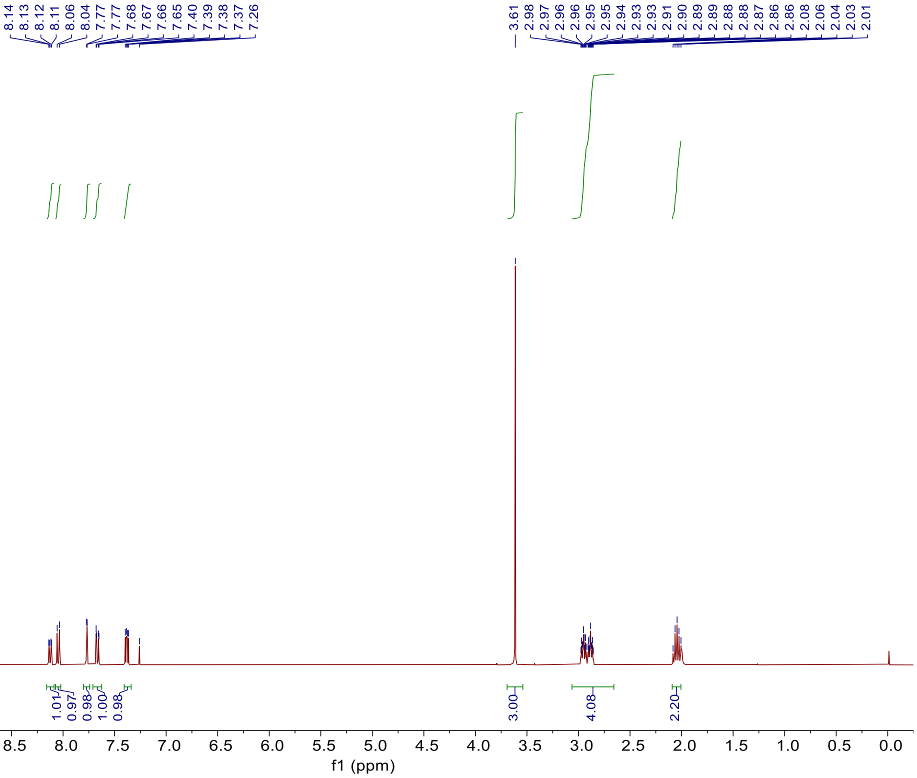

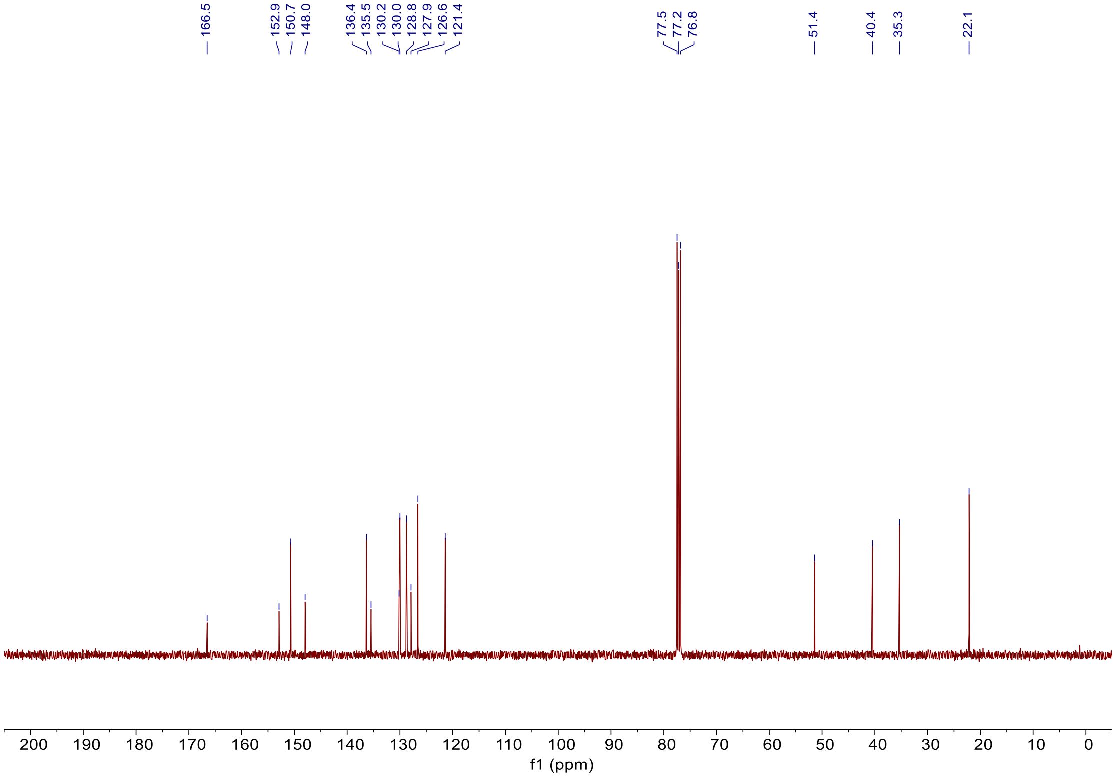

400 MHz ^{1}H NMR spectrum; 100.6 MHz ^{13}C NMR spectrum; DMSO-d_6 of **20a**

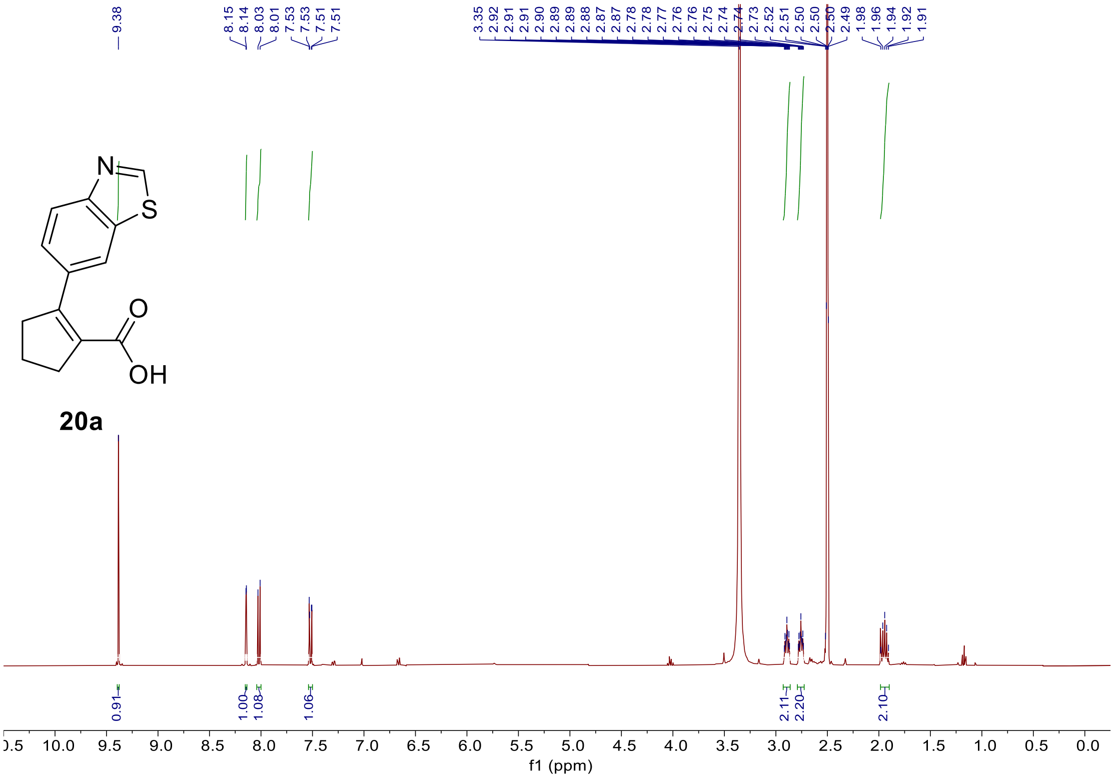

20a

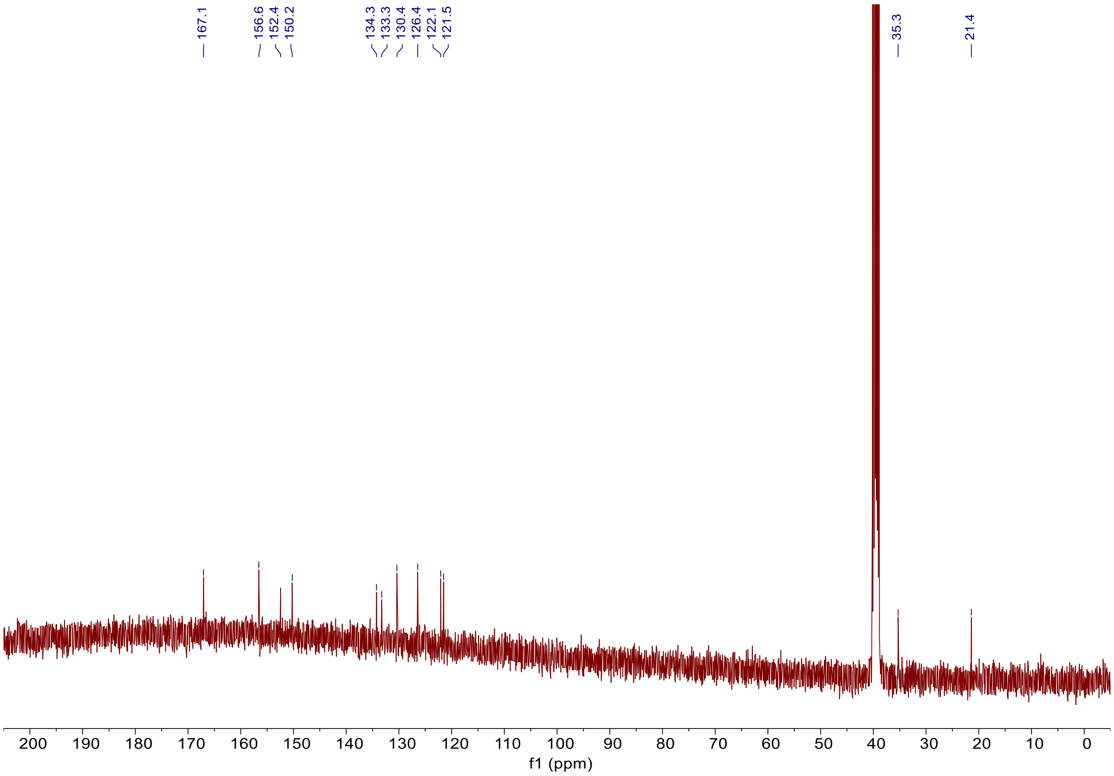

400 MHz ^{1}H NMR spectrum; 100.6 MHz ^{13}C NMR spectrum; DMSO-d_6 of **20b**

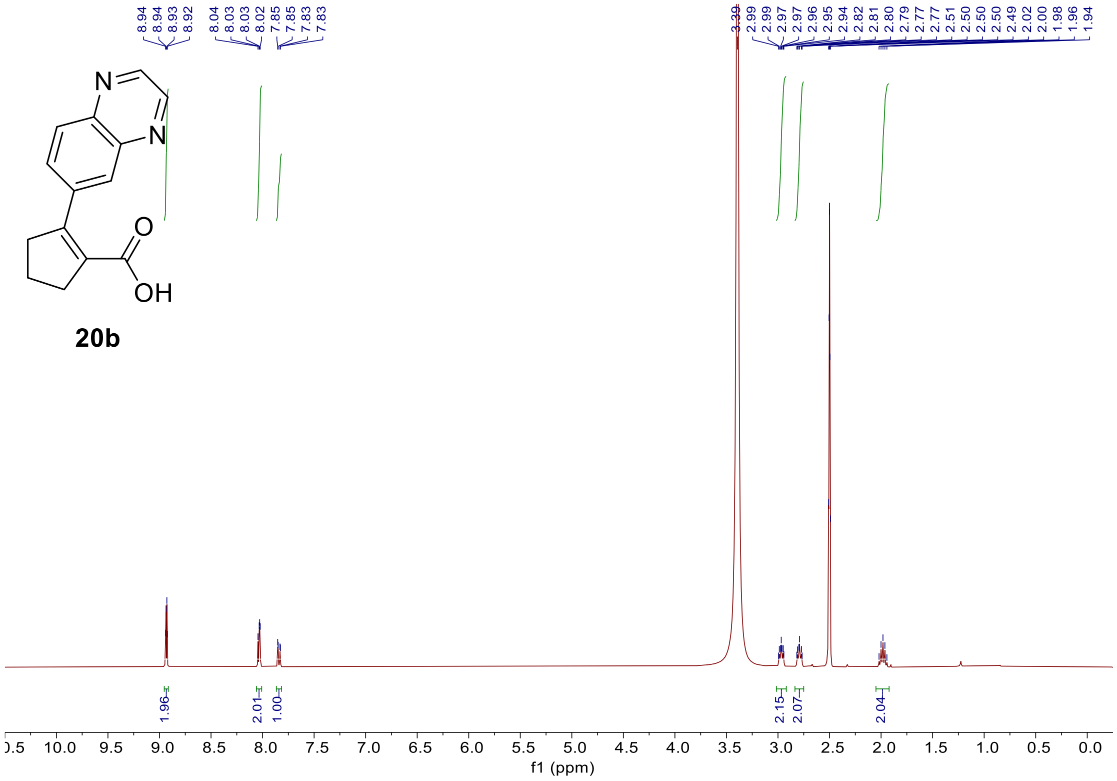

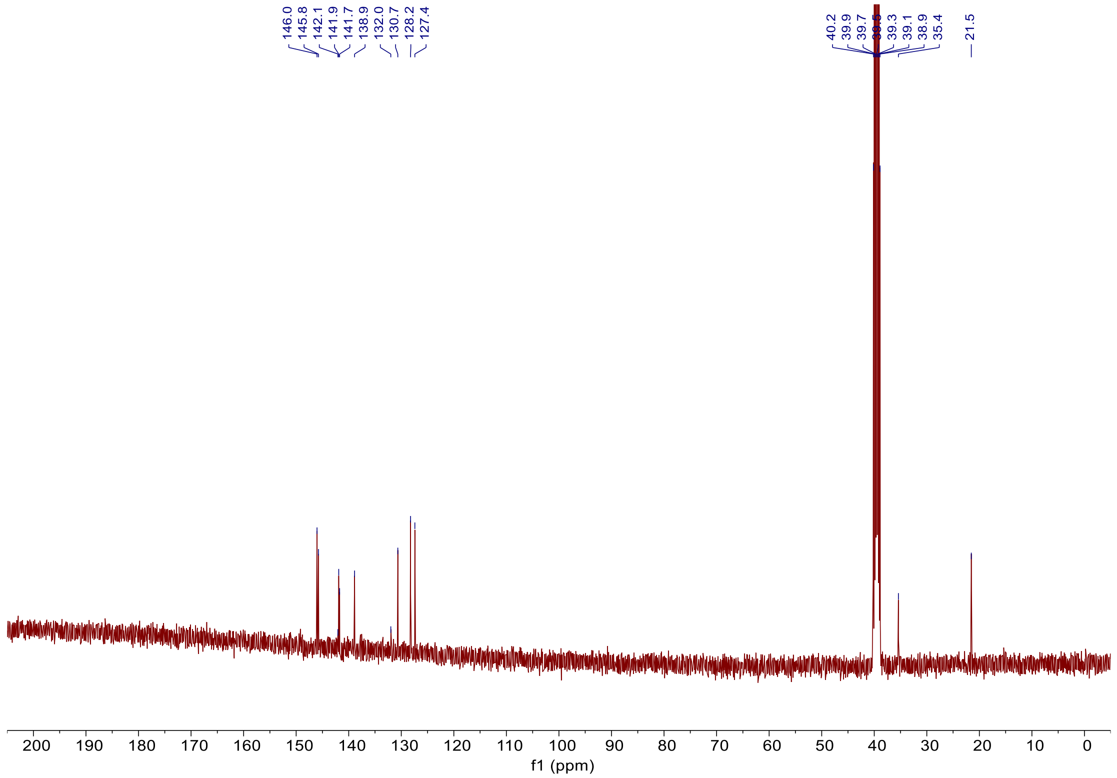

400 MHz ^{1}H NMR spectrum; 100.6 MHz ^{13}C NMR spectrum; DMSO-d_6 of **20c**

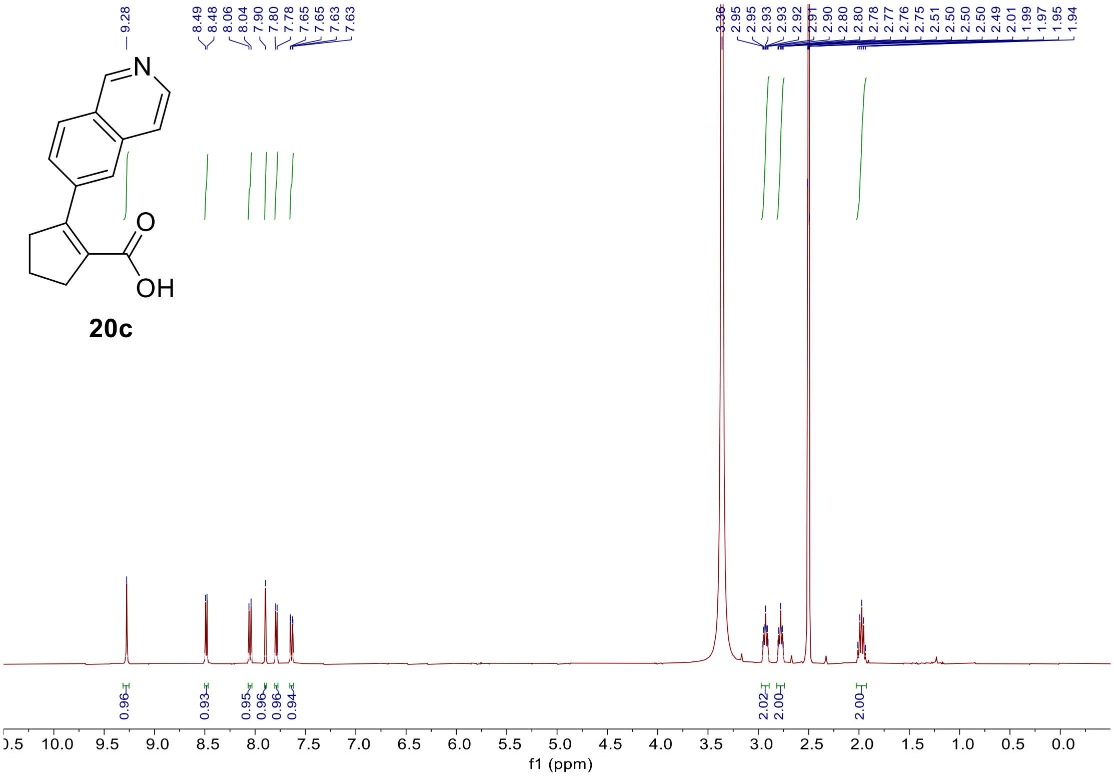

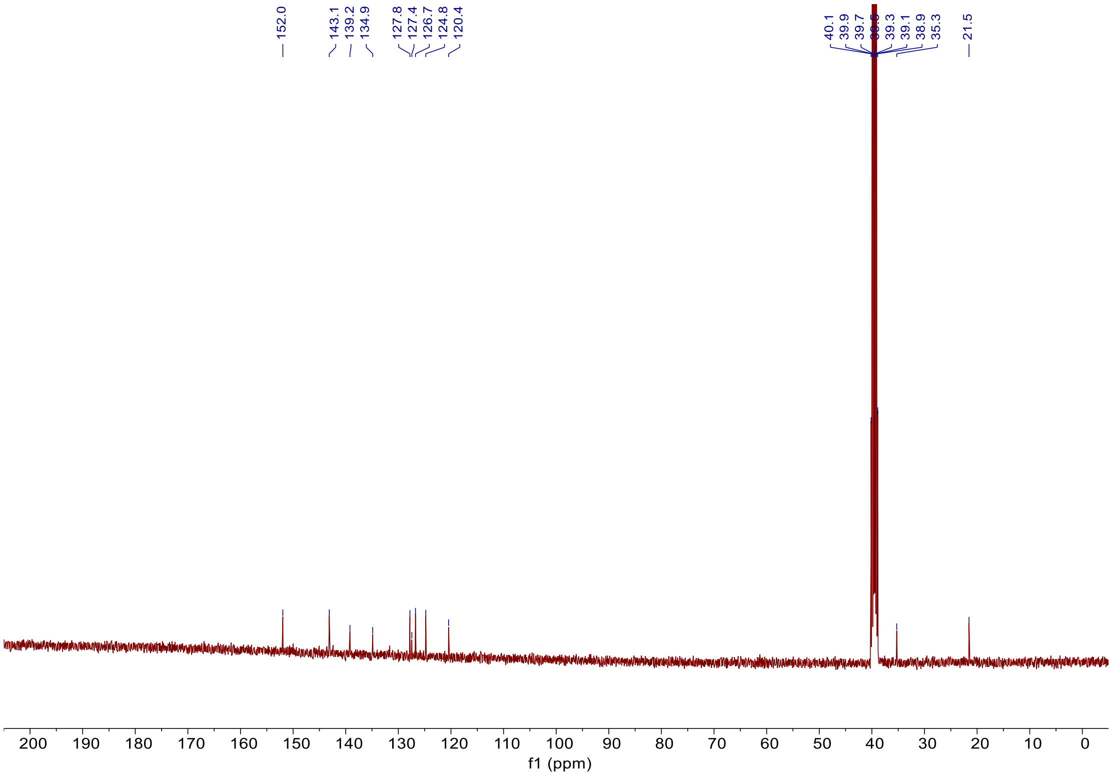

400 MHz ^{1}H NMR spectrum; 100.6 MHz ^{13}C NMR spectrum; DMSO-d_6 of **20d**

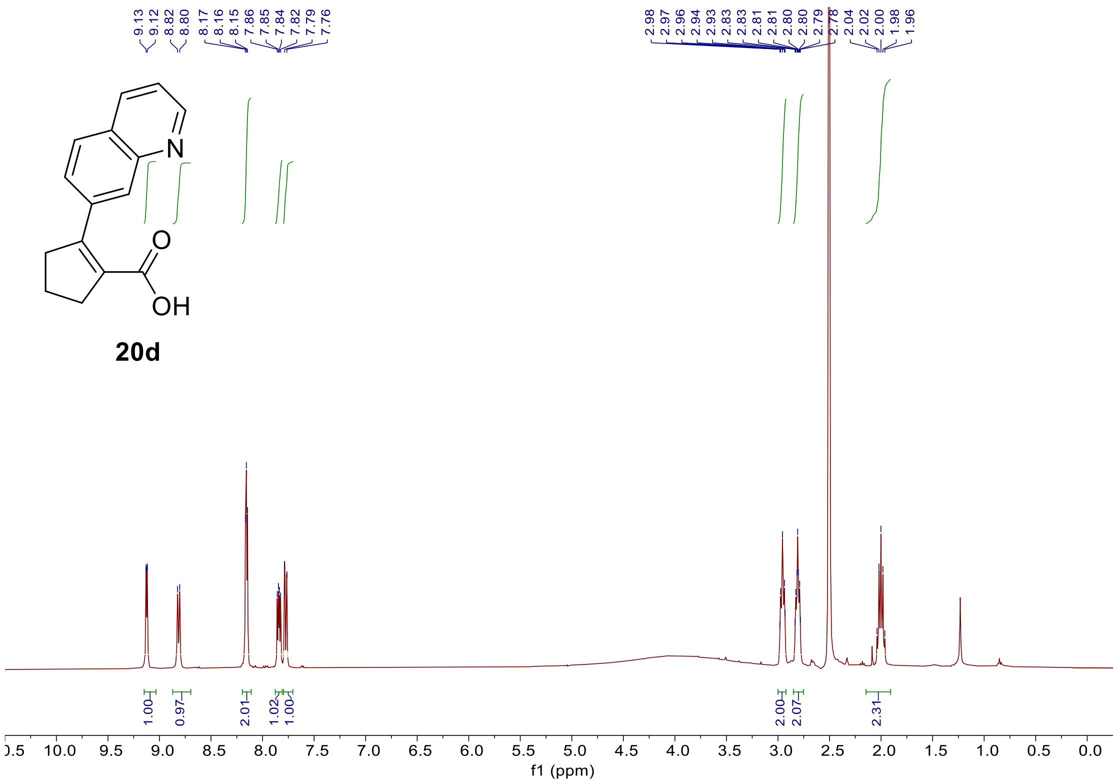

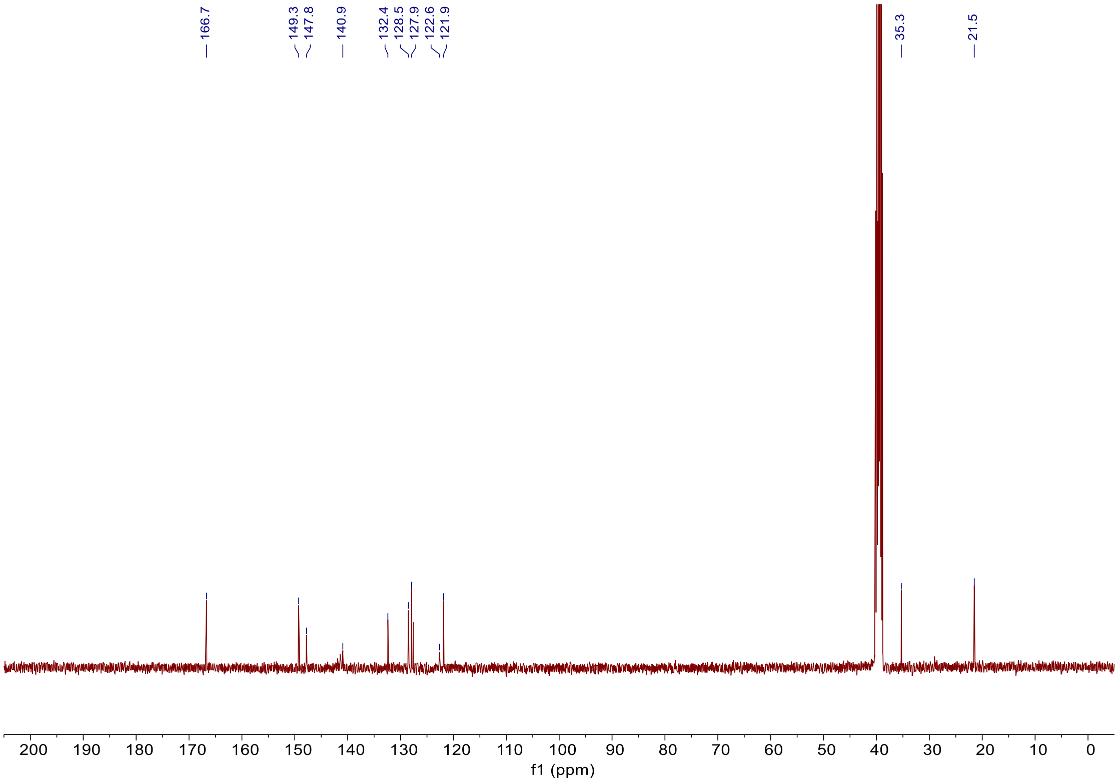

400 MHz ^{1}H NMR spectrum; 100.6 MHz ^{13}C NMR spectrum; $CDCl_3$ of **20e**

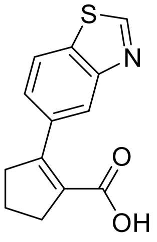

20e

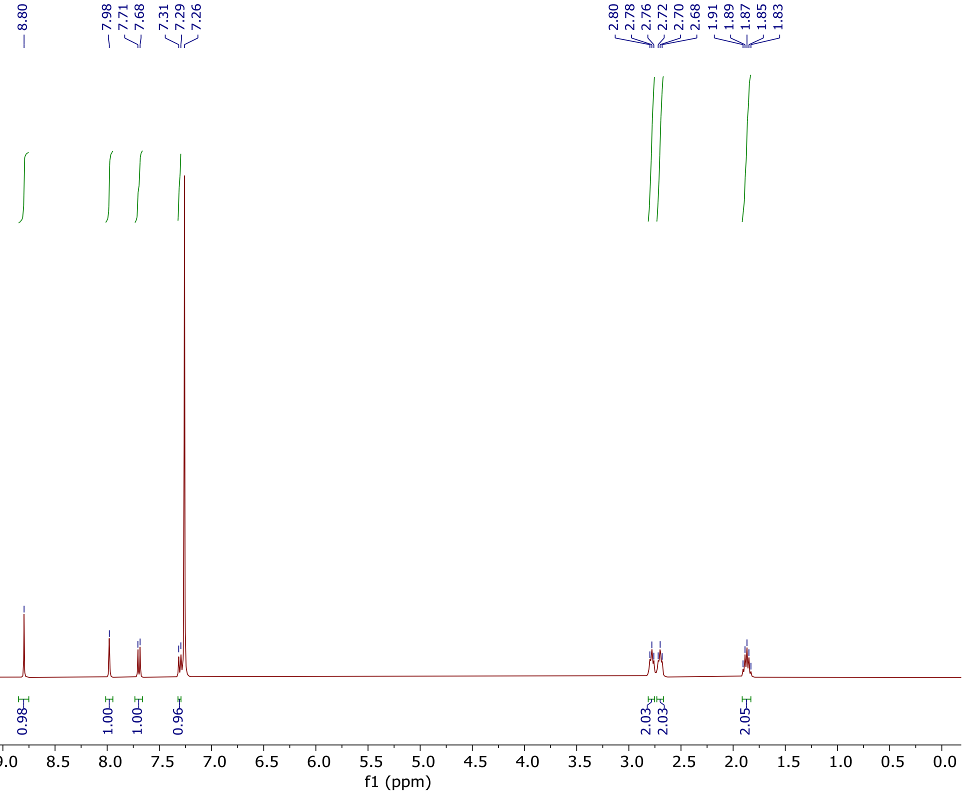

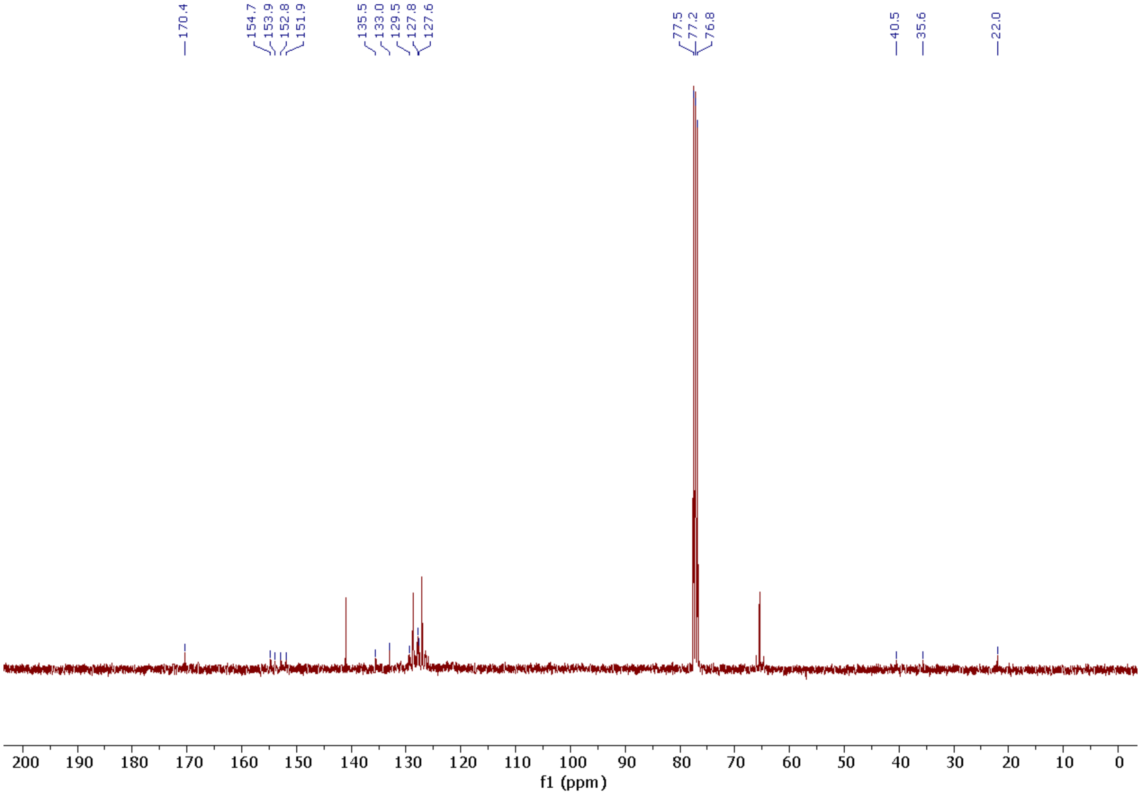

400 MHz ^{1}H NMR spectrum; 100.6 MHz ^{13}C NMR spectrum; $CDCl_3$ of **20f**

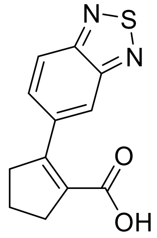

20f

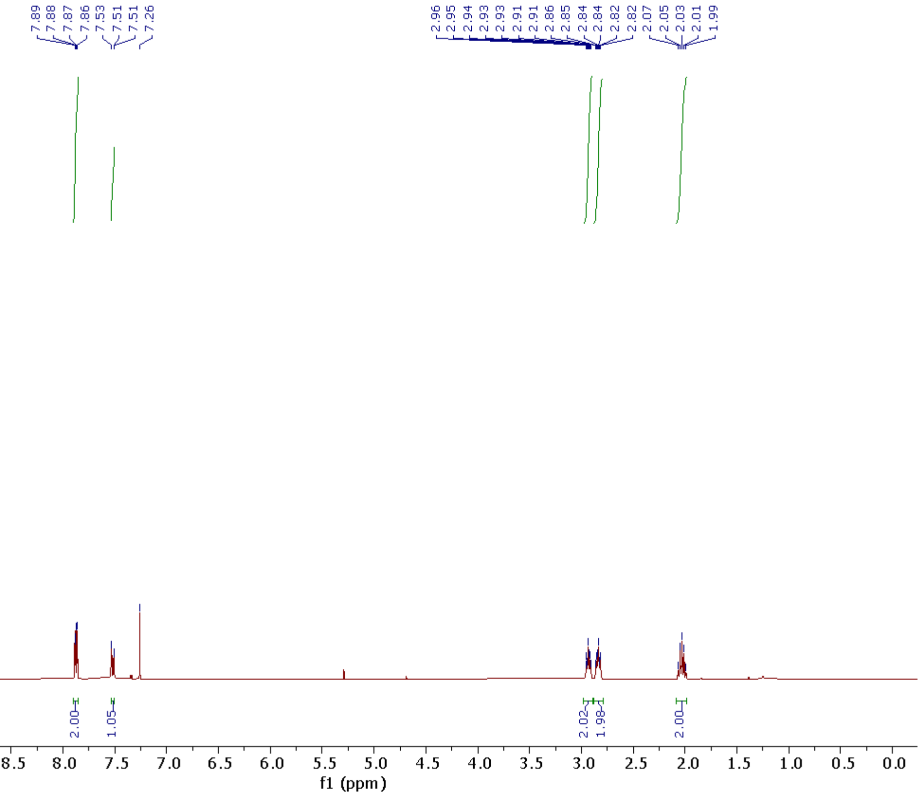

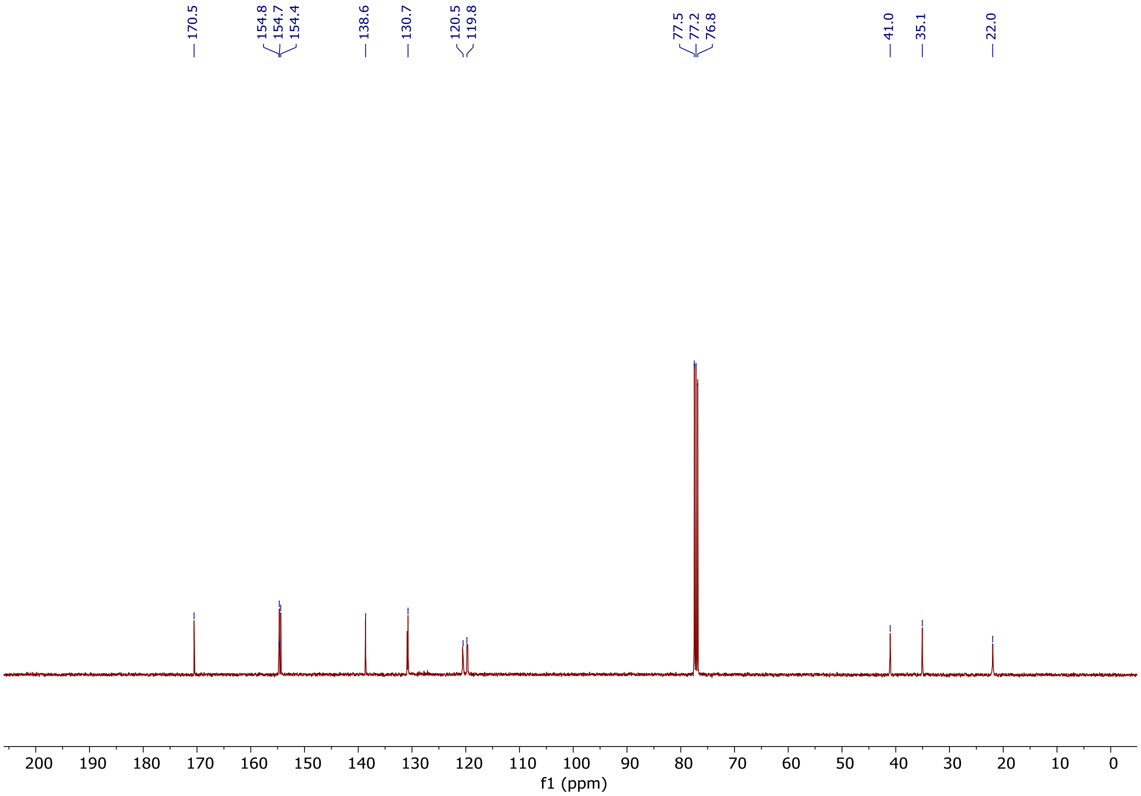

400 MHz ^{1}H NMR spectrum; 100.6 MHz ^{13}C NMR spectrum; DMSO-d_6 of **20g**

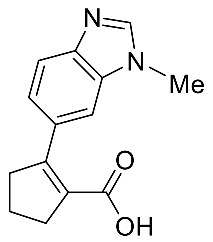

20g

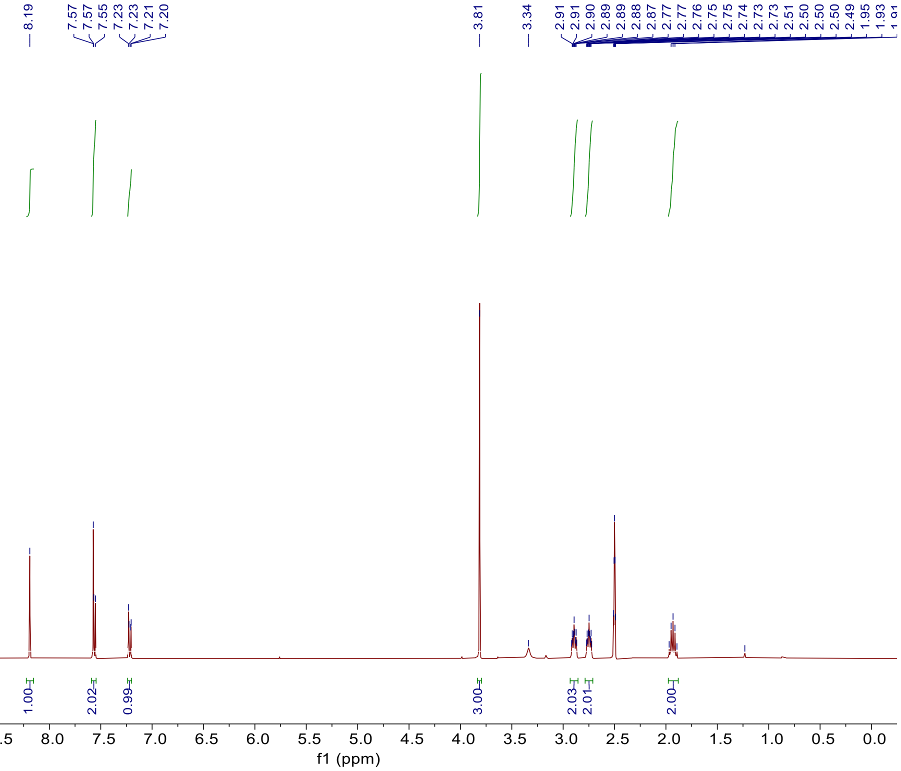

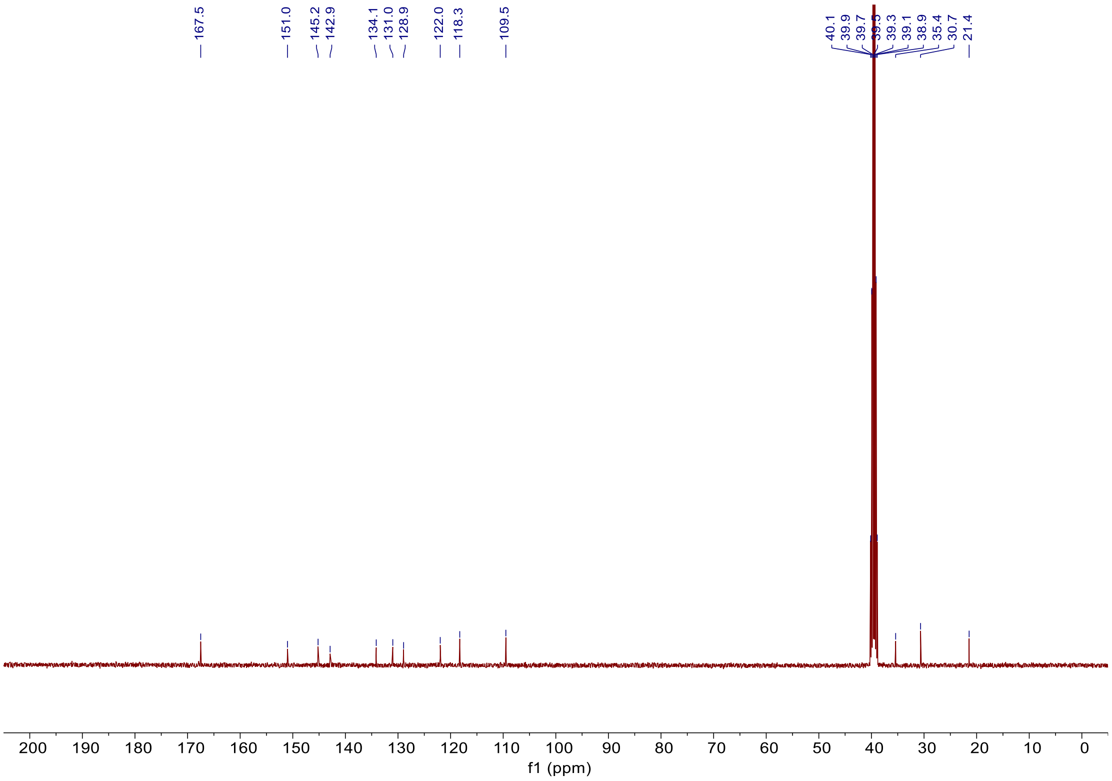

400 MHz ^{1}H NMR spectrum; 100.6 MHz ^{13}C NMR spectrum; DMSO-d_6 of **20h**

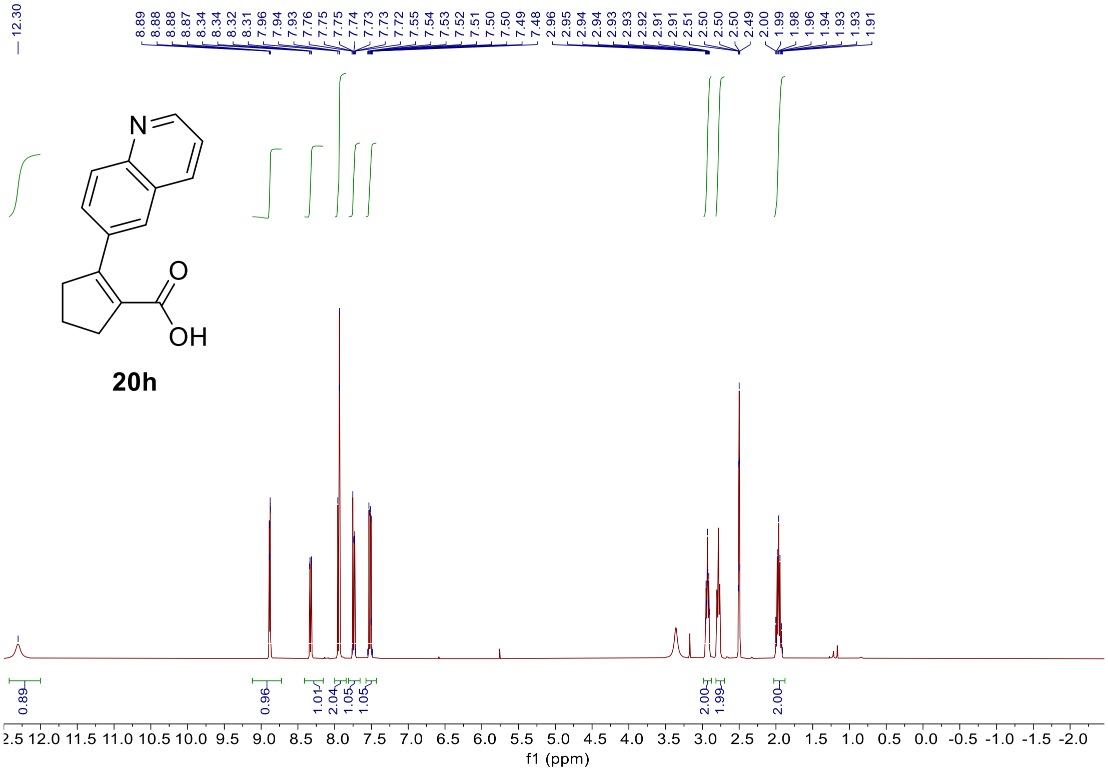

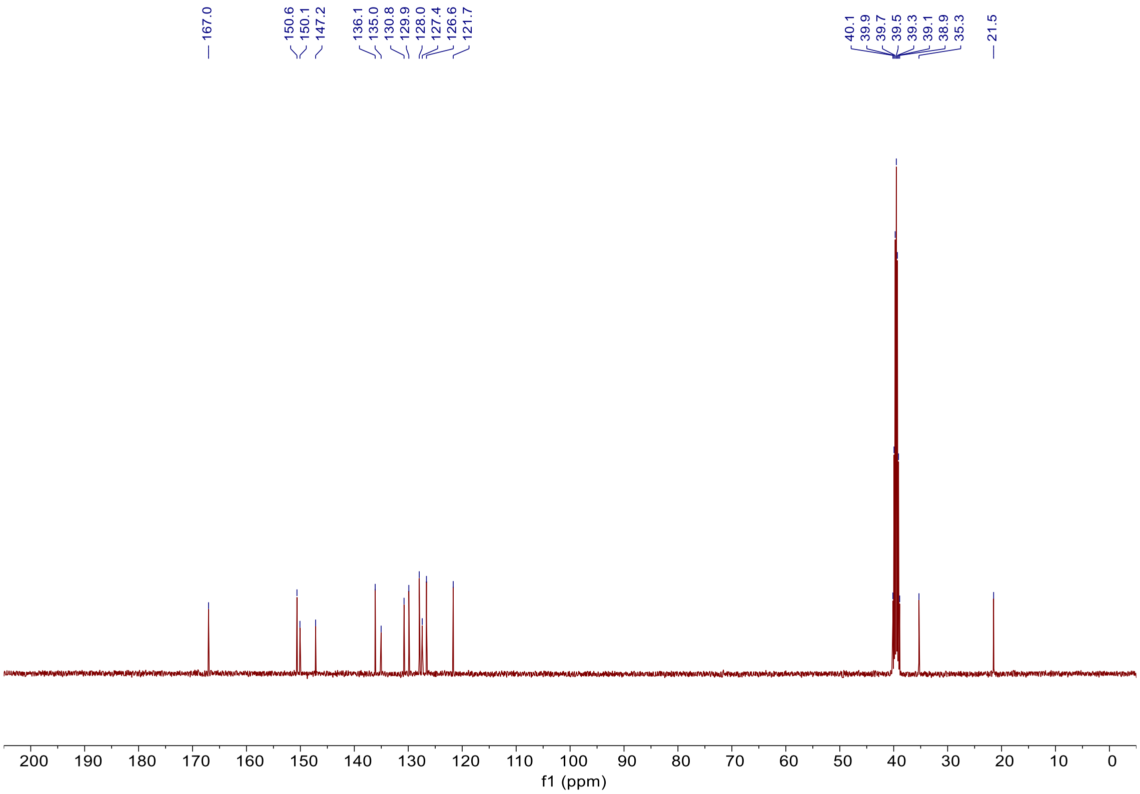

400 MHz ^{1}H NMR spectrum; 100.6 MHz ^{13}C NMR spectrum; $CDCl_3$of *cis*-**21**

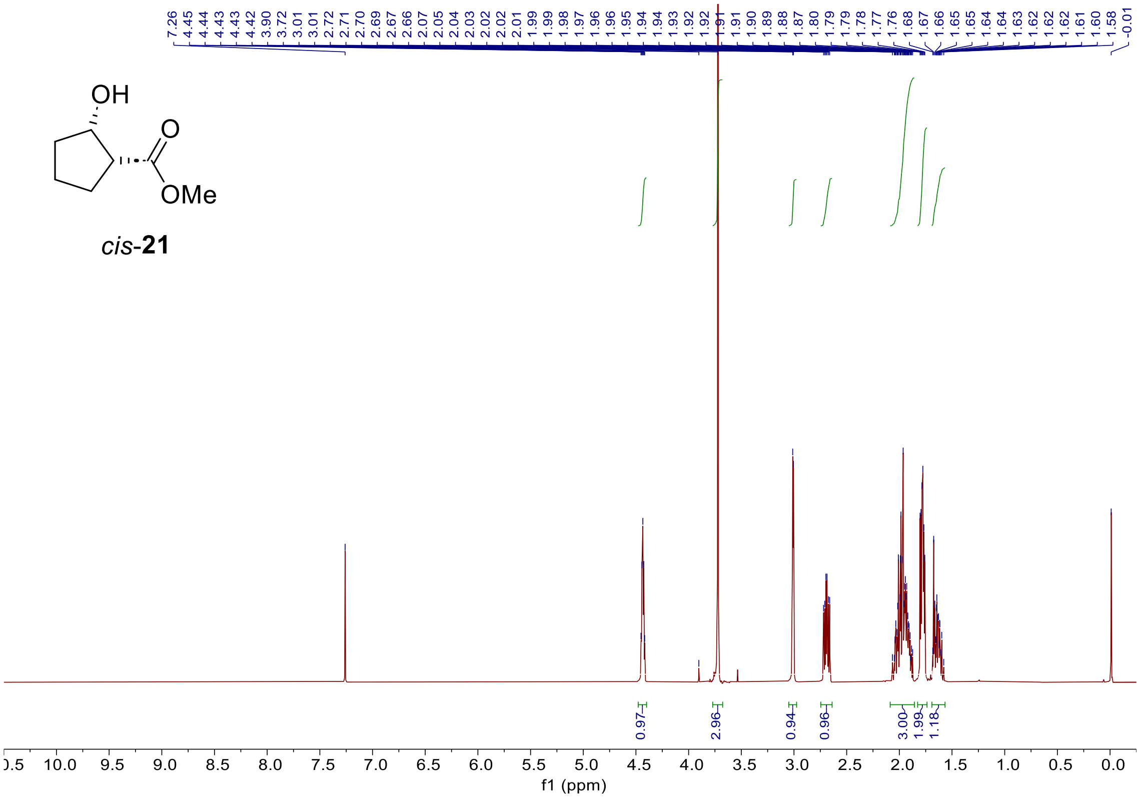

cis-**21**

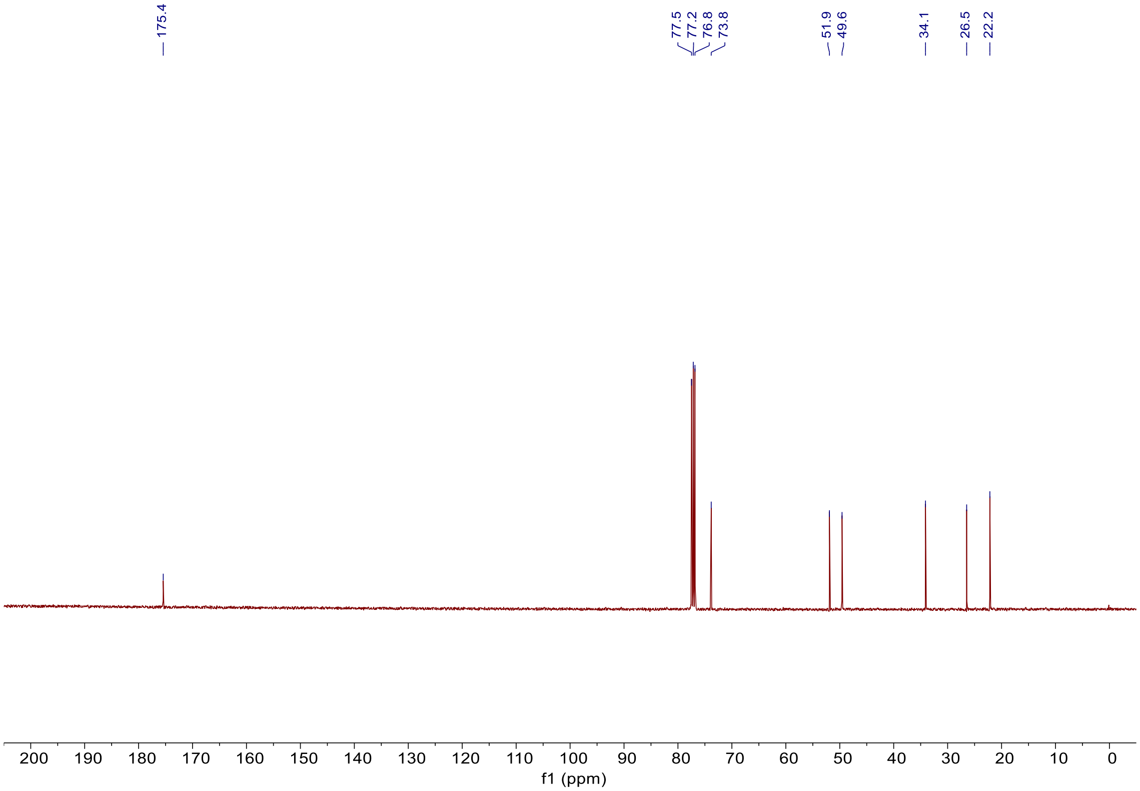

400 MHz ^{1}H NMR spectrum; 100.6 MHz ^{13}C NMR spectrum; $CDCl_3$of *trans*-**21**

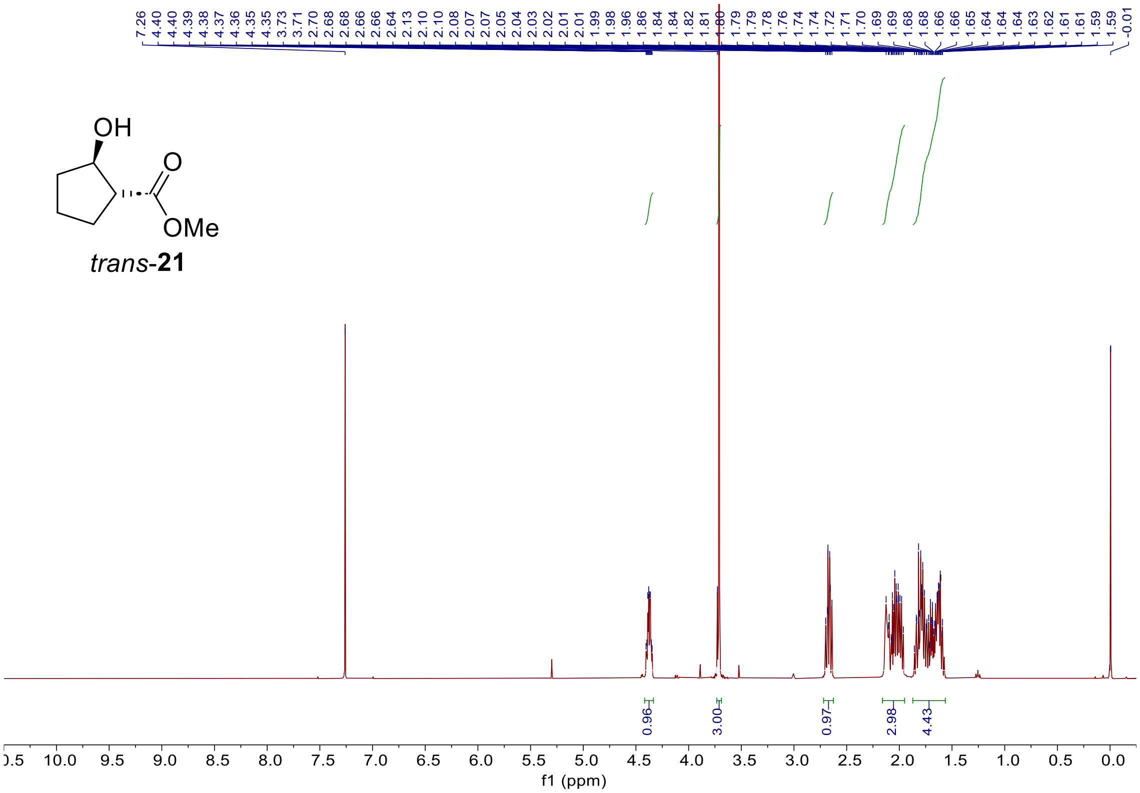

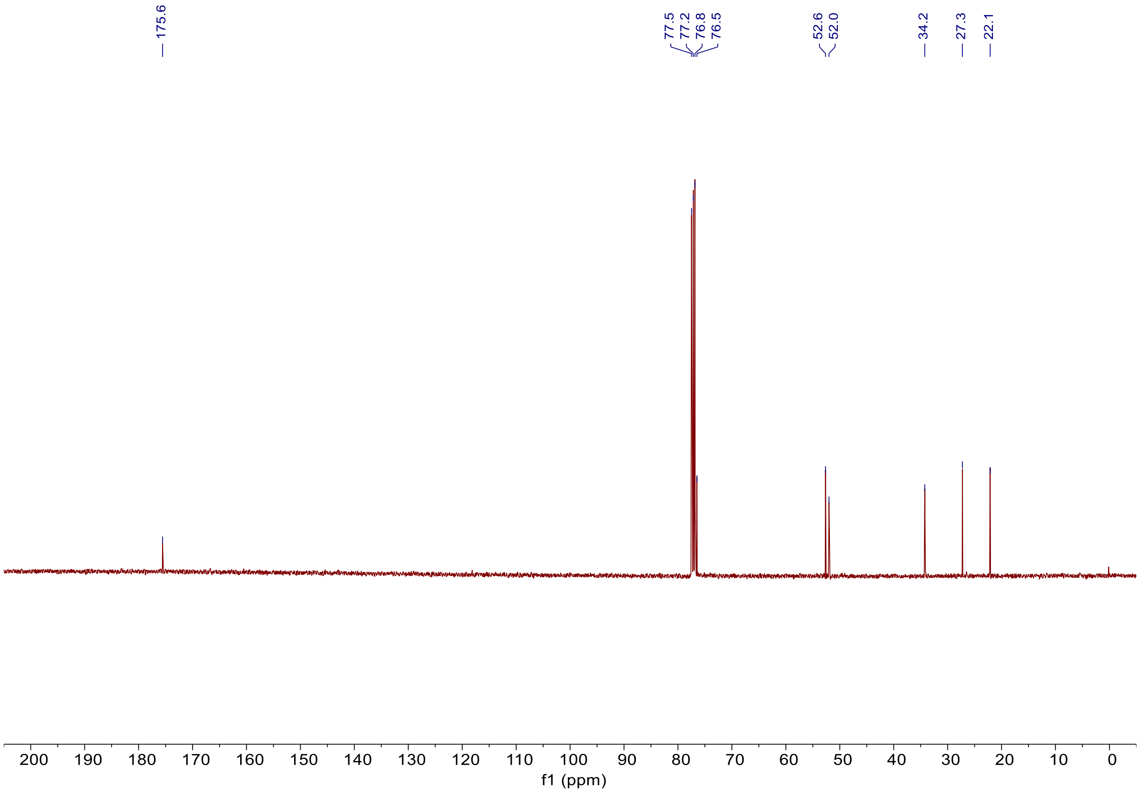

400 MHz ^{1}H NMR spectrum; $CDCl_3$of *cis*-**21** & *trans*-**21**(*trans*-**21** : *cis*-**21** = 56:44)

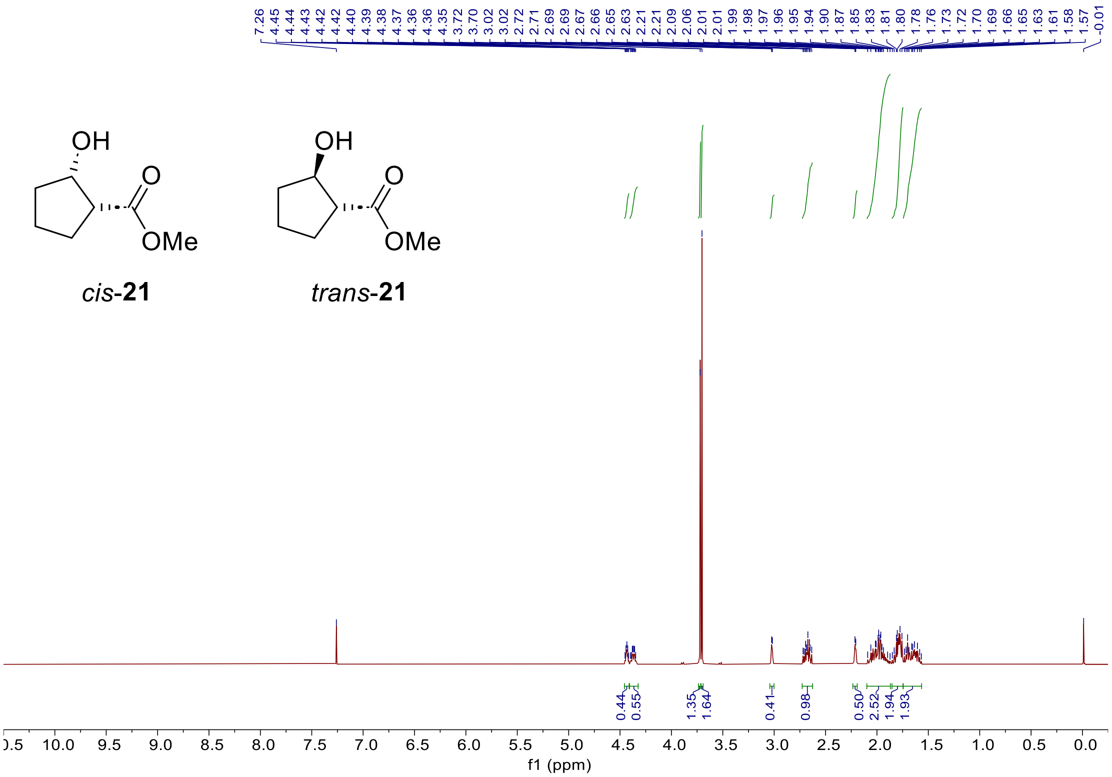

cis-**21** *trans*-**21**

400 MHz ^{1}H NMR spectrum; 100.6 MHz ^{13}C NMR spectrum; $CDCl_3$of *trans*-**22a**

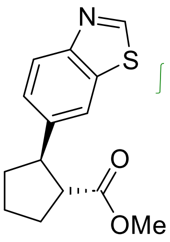

trans-**22a**

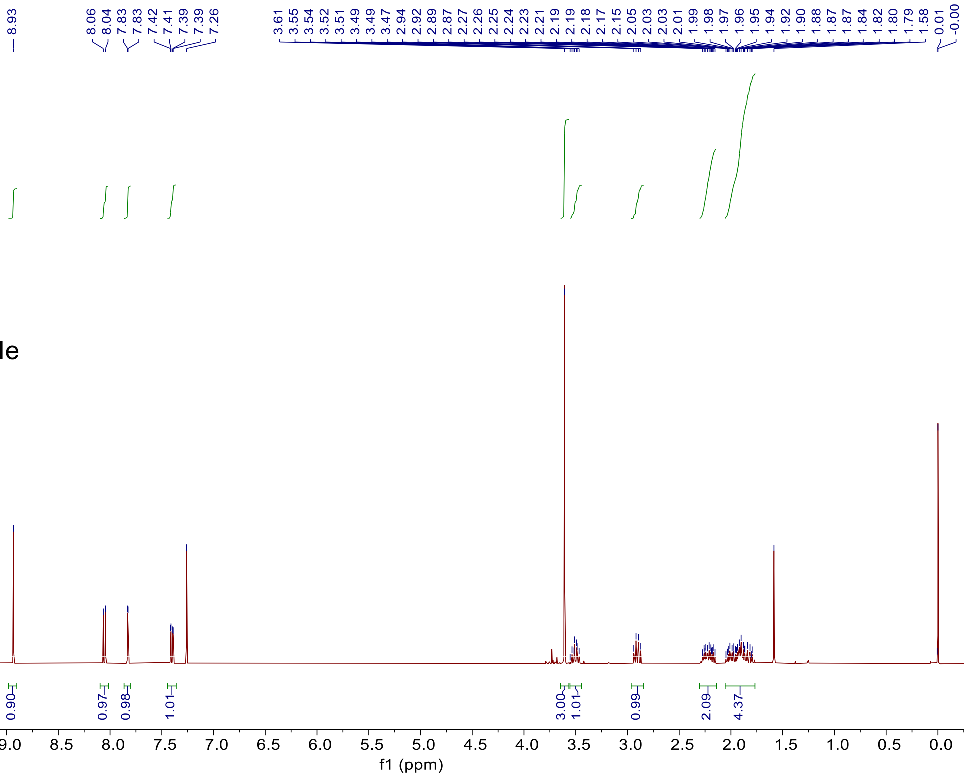

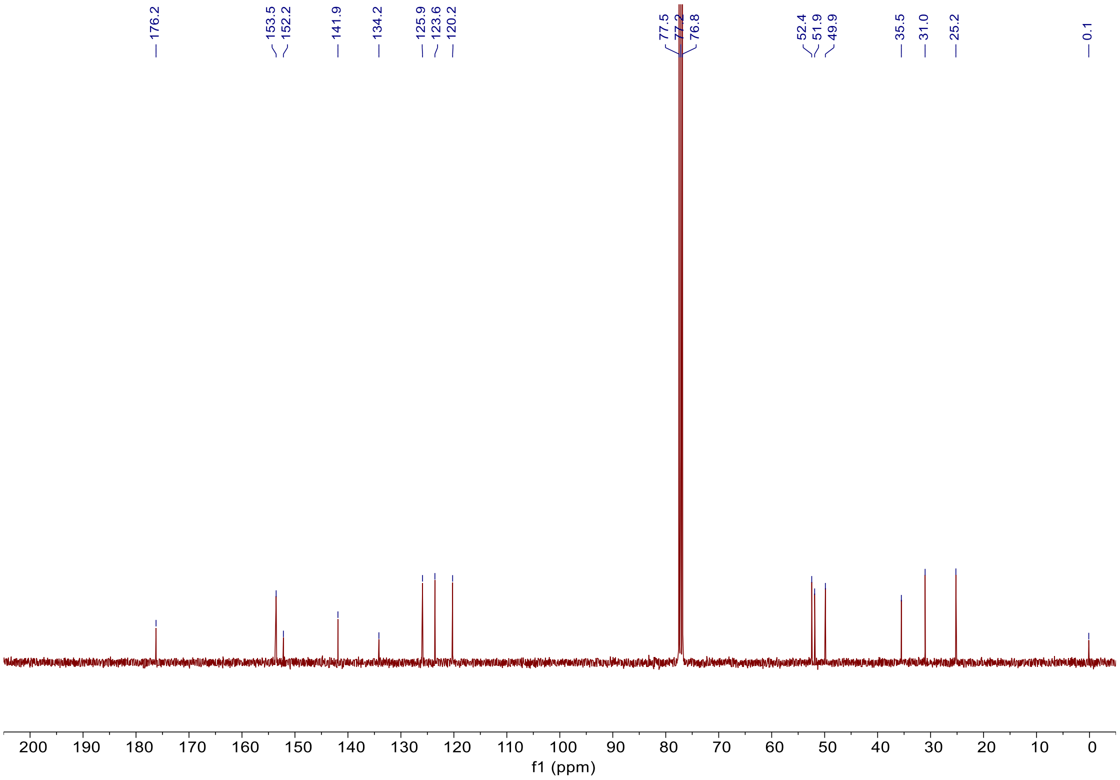

400 MHz ^{1}H NMR spectrum; 100.6 MHz ^{13}C NMR spectrum; $CDCl_3$of *trans*-**22b**

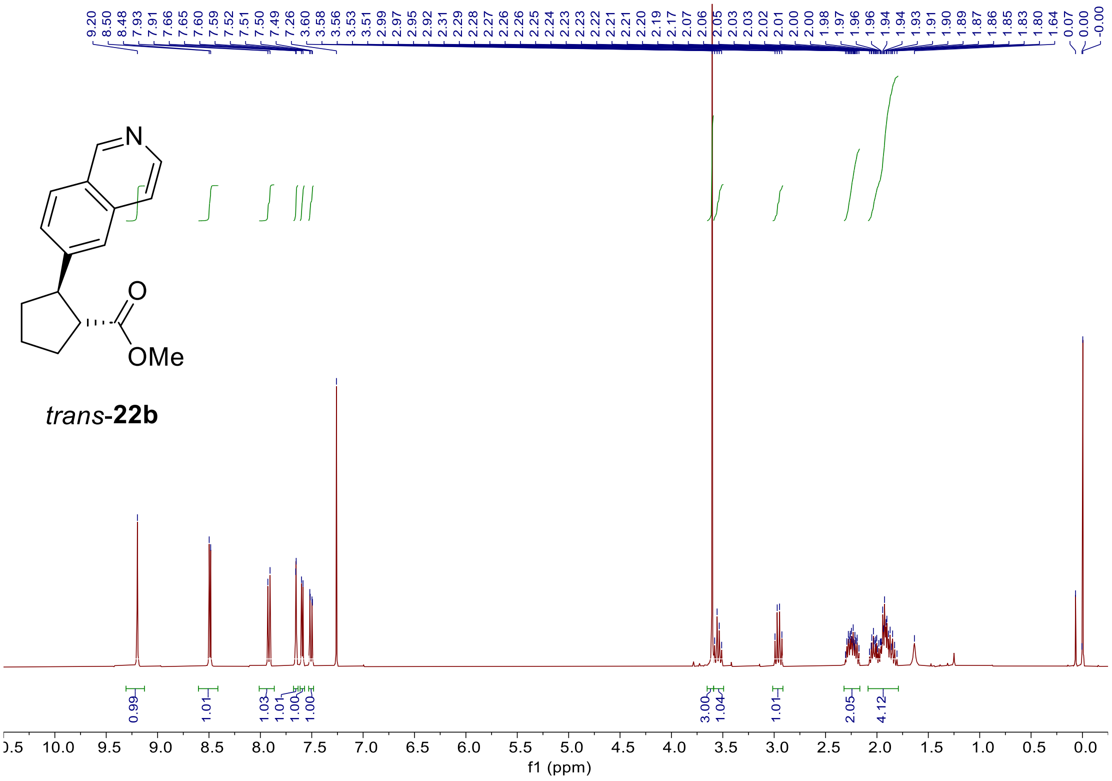

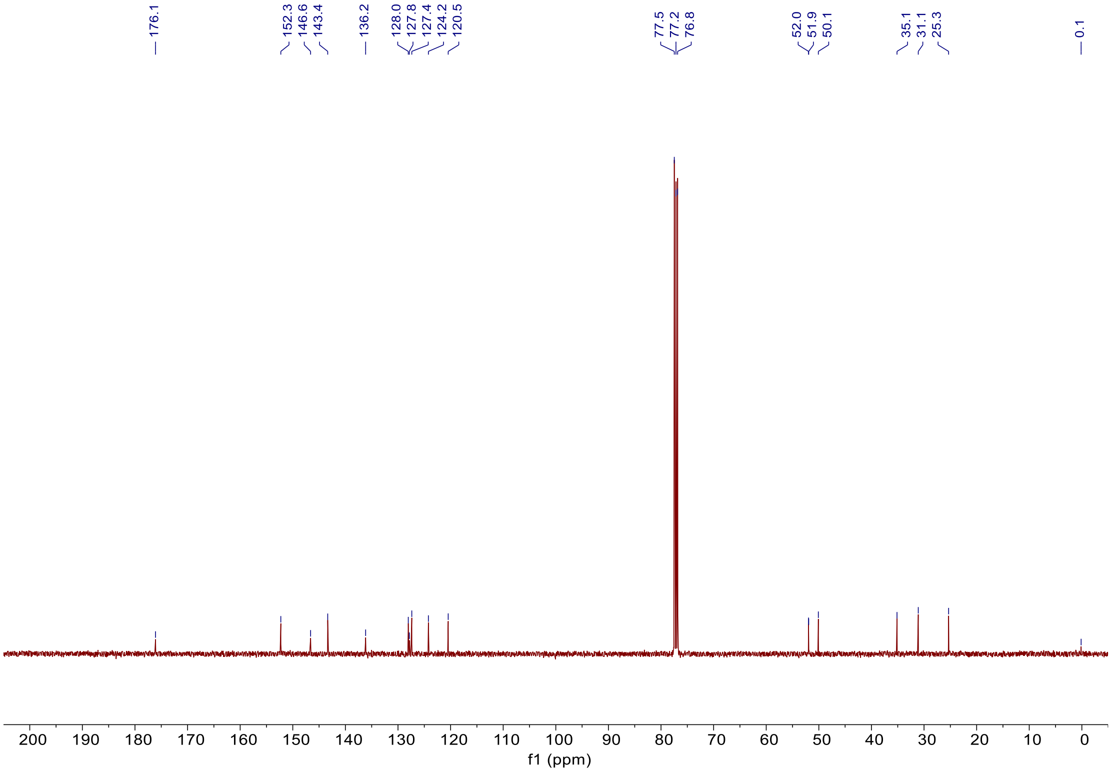

400 MHz ^{1}H NMR spectrum; 100.6 MHz ^{13}C NMR spectrum; $CDCl_3$of *trans*-**22c**

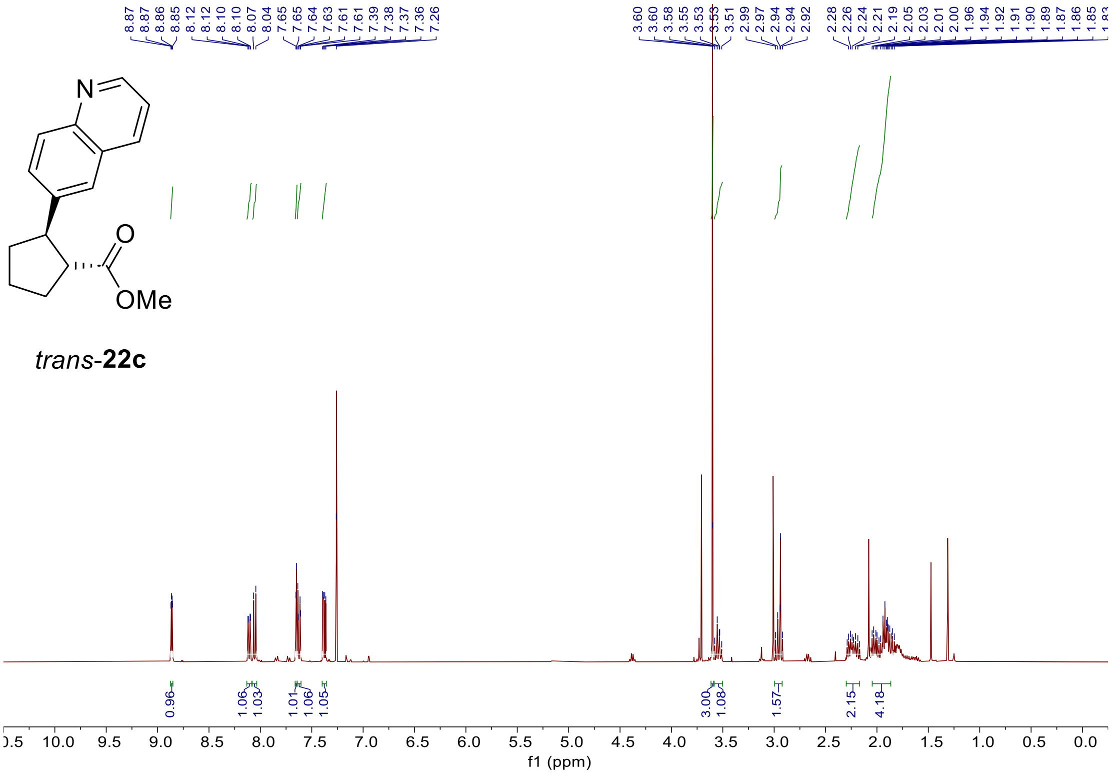

trans-**22c**

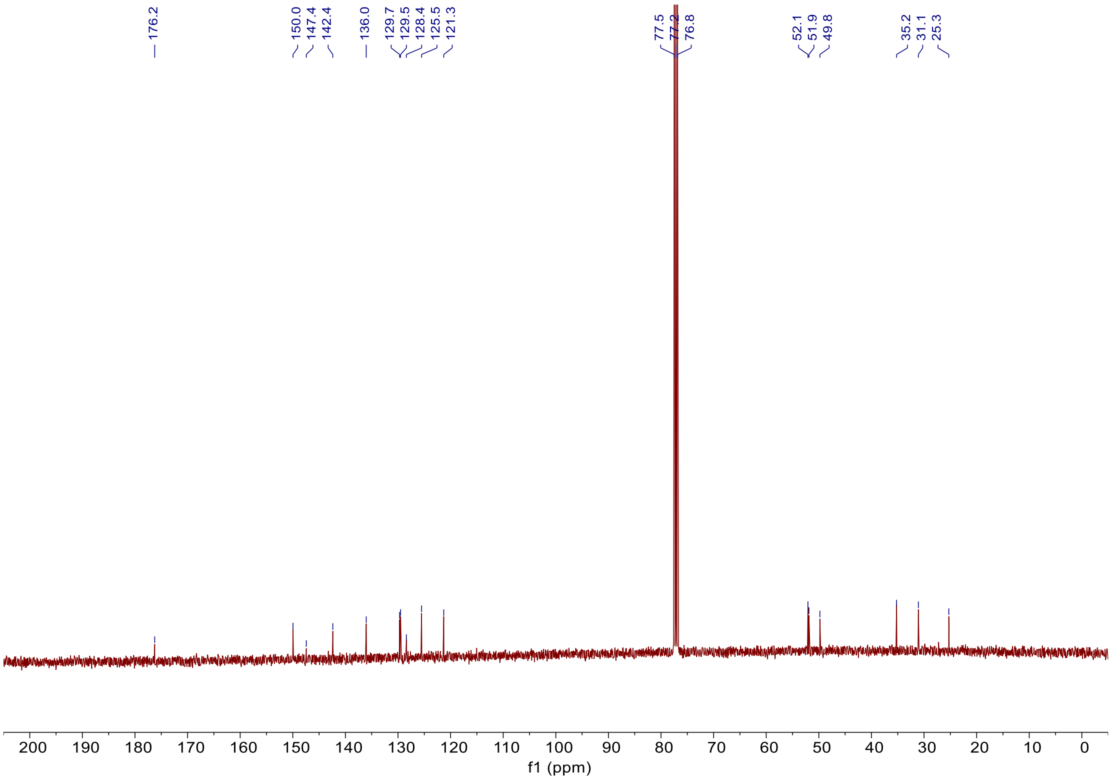

400 MHz ^{1}H NMR spectrum; 100.6 MHz ^{13}C NMR spectrum; $CDCl_3$of *trans*-**22d**

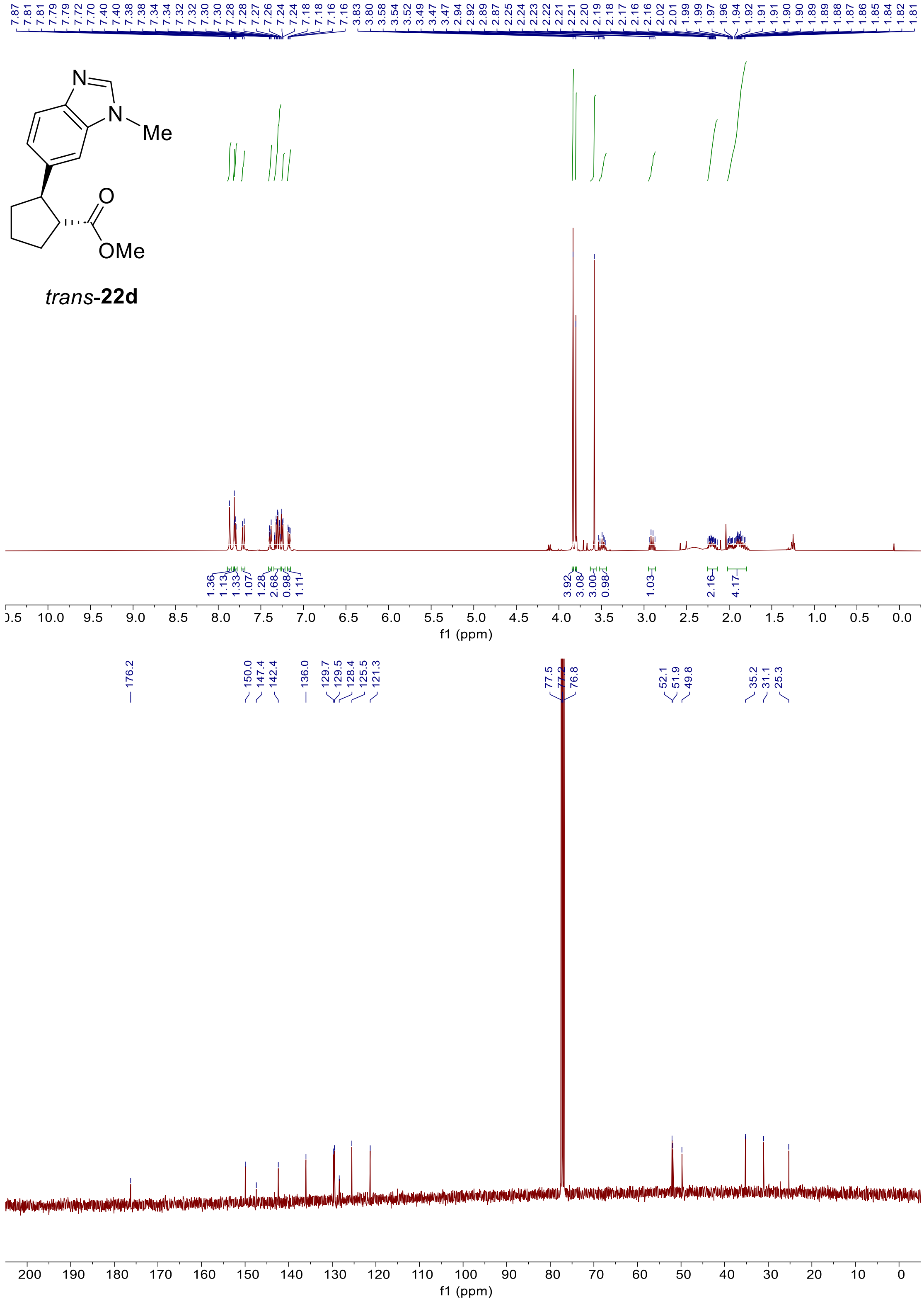

400 MHz ^{1}H NMR spectrum; 100.6 MHz ^{13}C NMR spectrum; $CDCl_3$of *trans*-**22e**

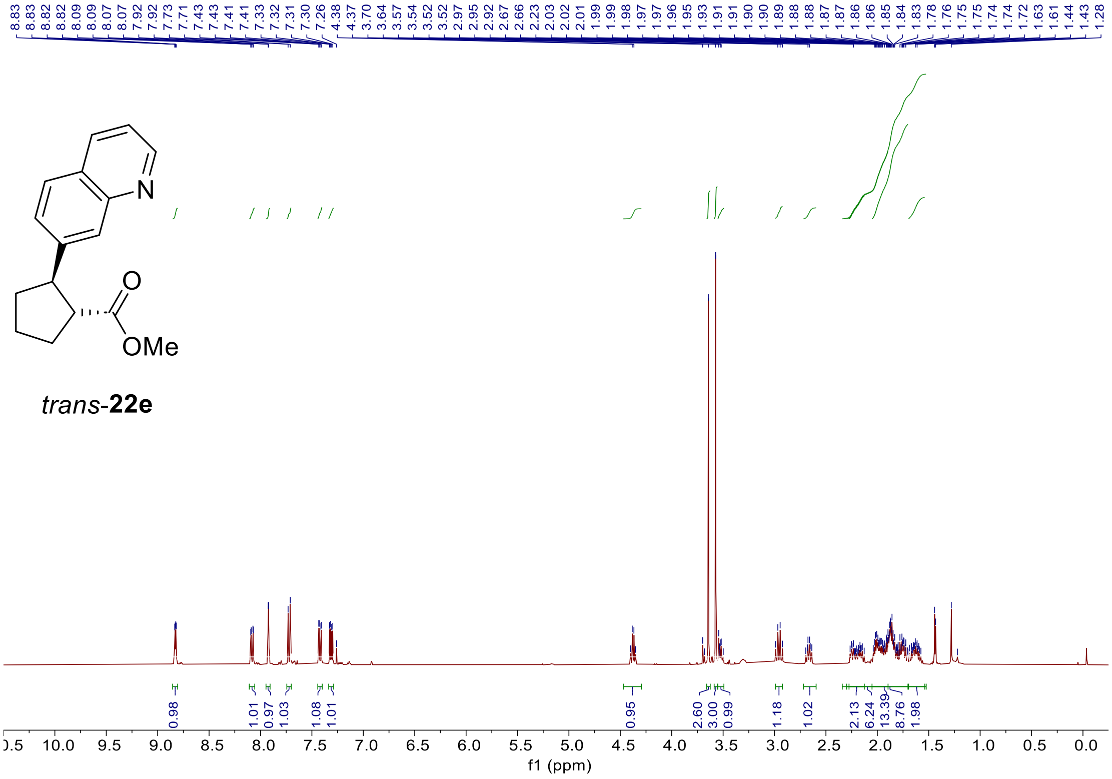

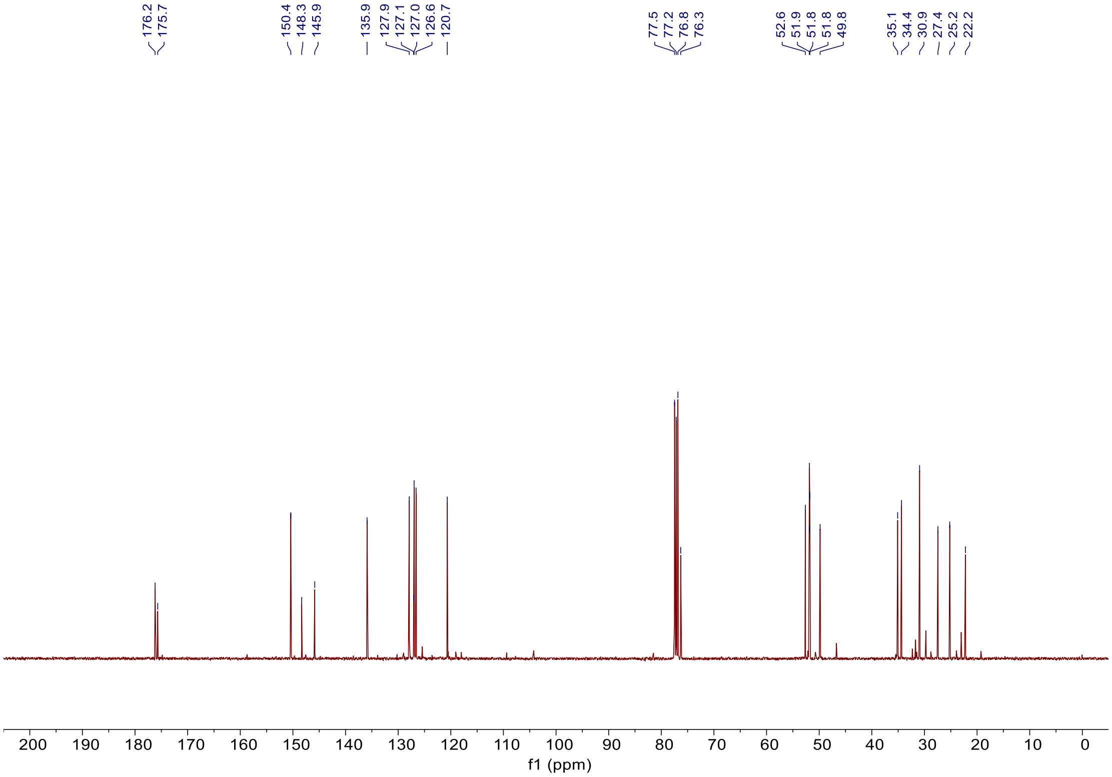

400 MHz ^{1}H NMR spectrum; 100.6 MHz ^{13}C NMR spectrum; $CDCl_3$of *trans*-**22f**

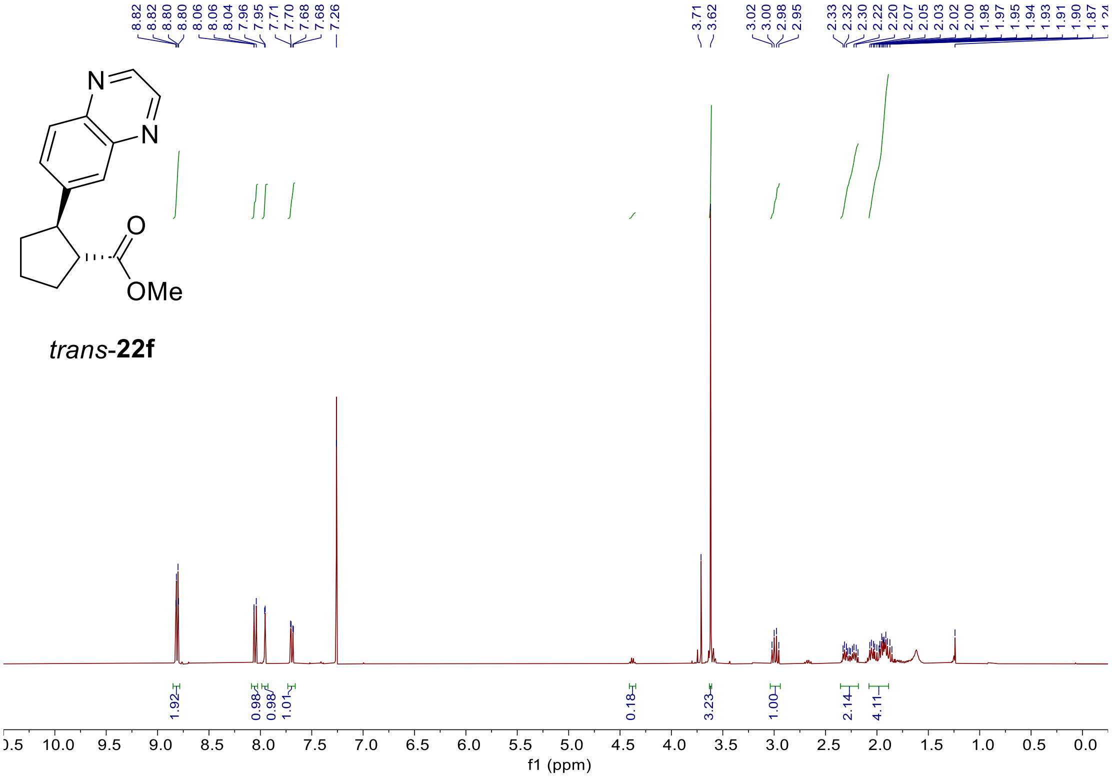

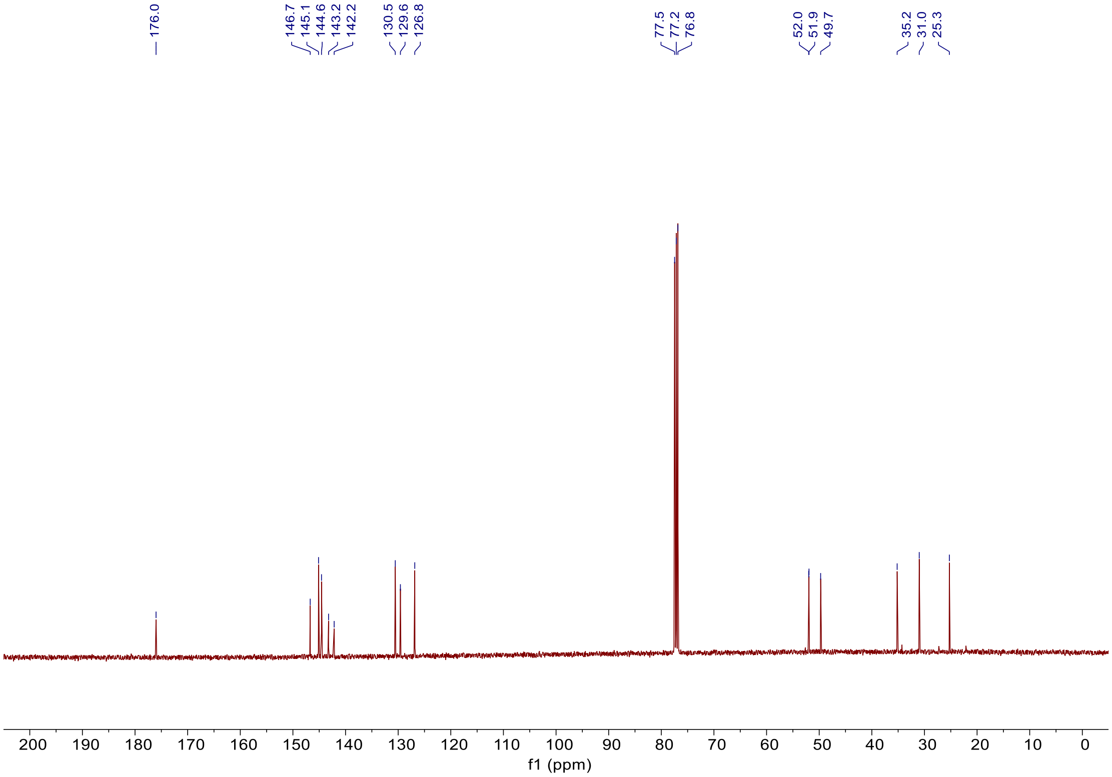

400 MHz ^{1}H NMR spectrum; 100.6 MHz ^{13}C NMR spectrum; $CDCl_3$of **23**

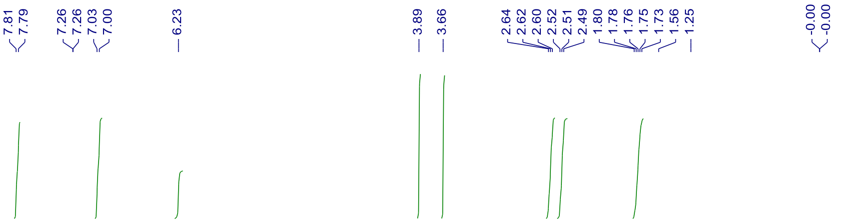

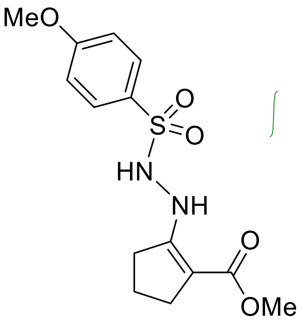

23

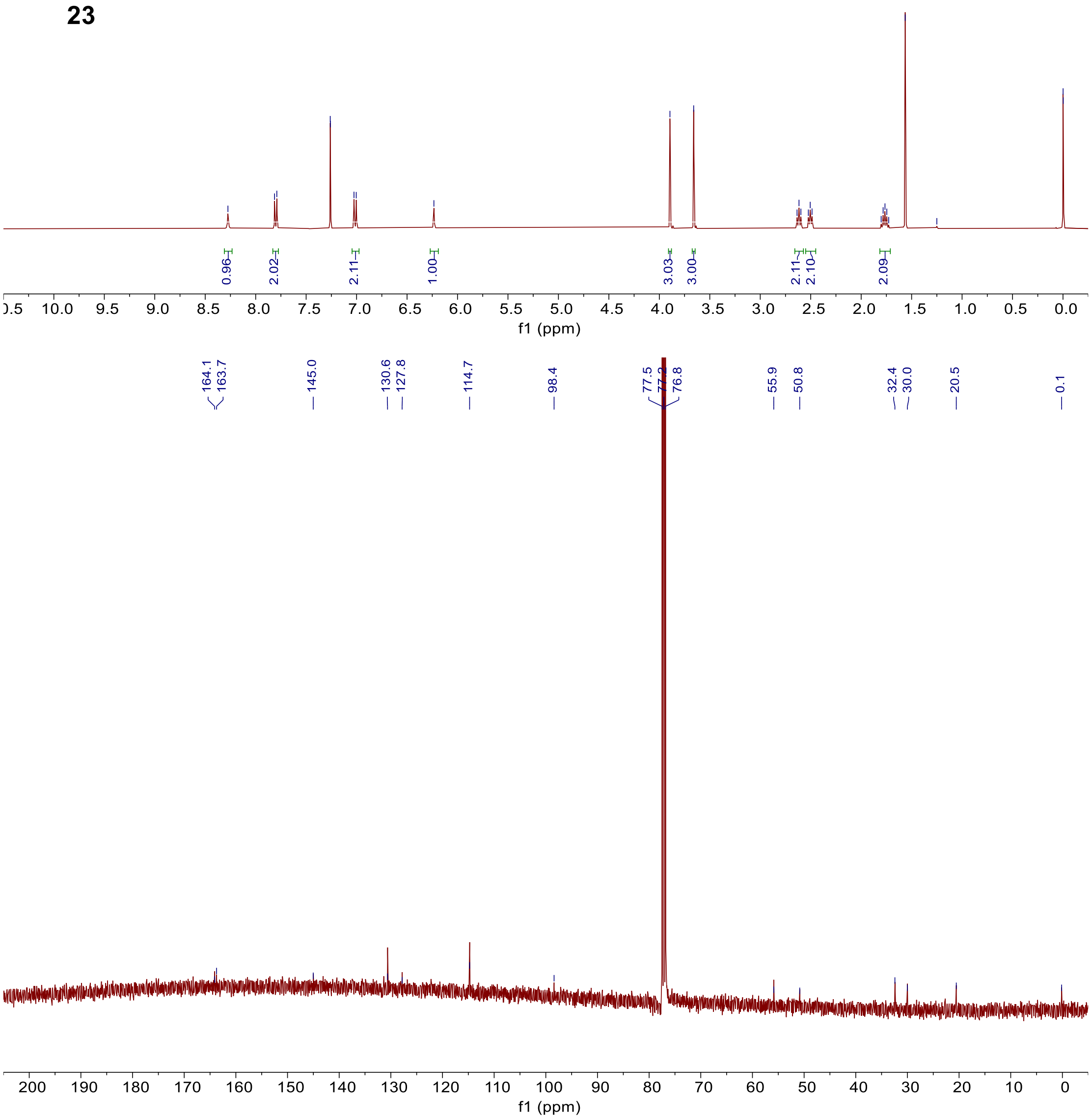

400 MHz ^{1}H NMR spectrum; $CDCl_3$of **23** & *Z*-**24** & *E*-**24** (**23** : *Z*-**24**:*E*-**24** = 24:33:33)

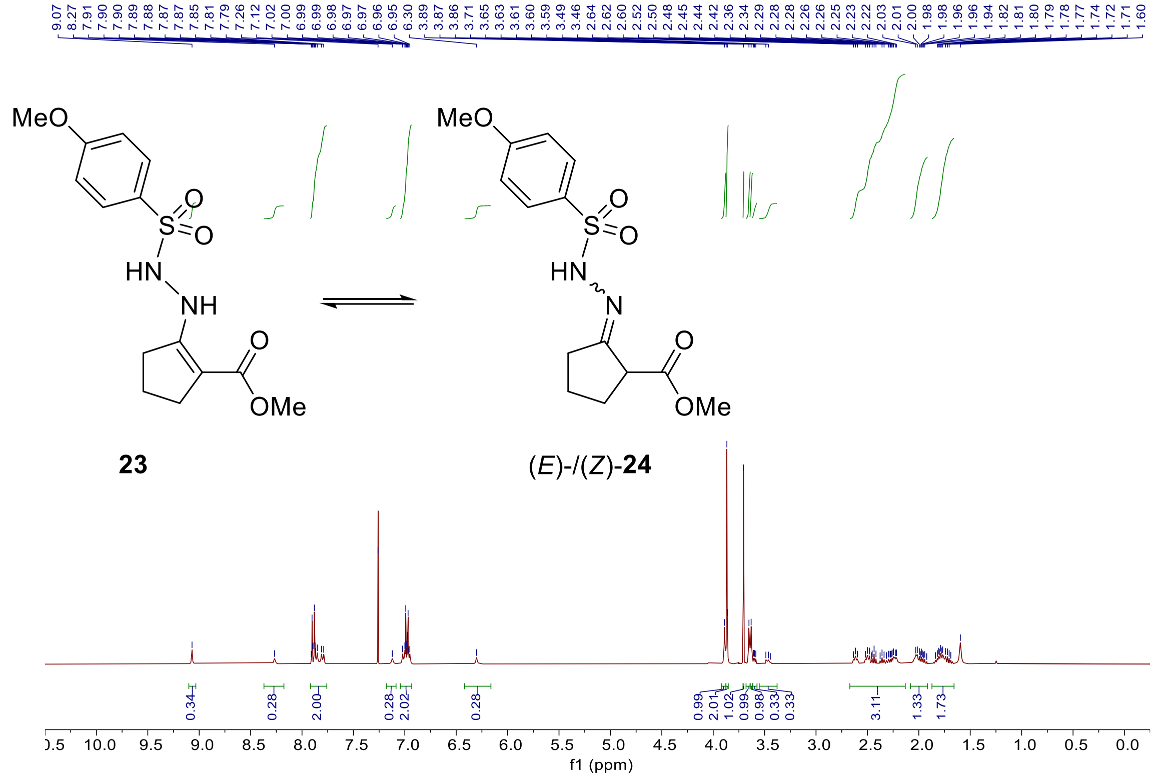

400 MHz ^{1}H NMR spectrum; 100.6 MHz ^{13}C NMR spectrum; 128 MHz ^{11}B NMR spectrum; $CDCl_3$of **36a**

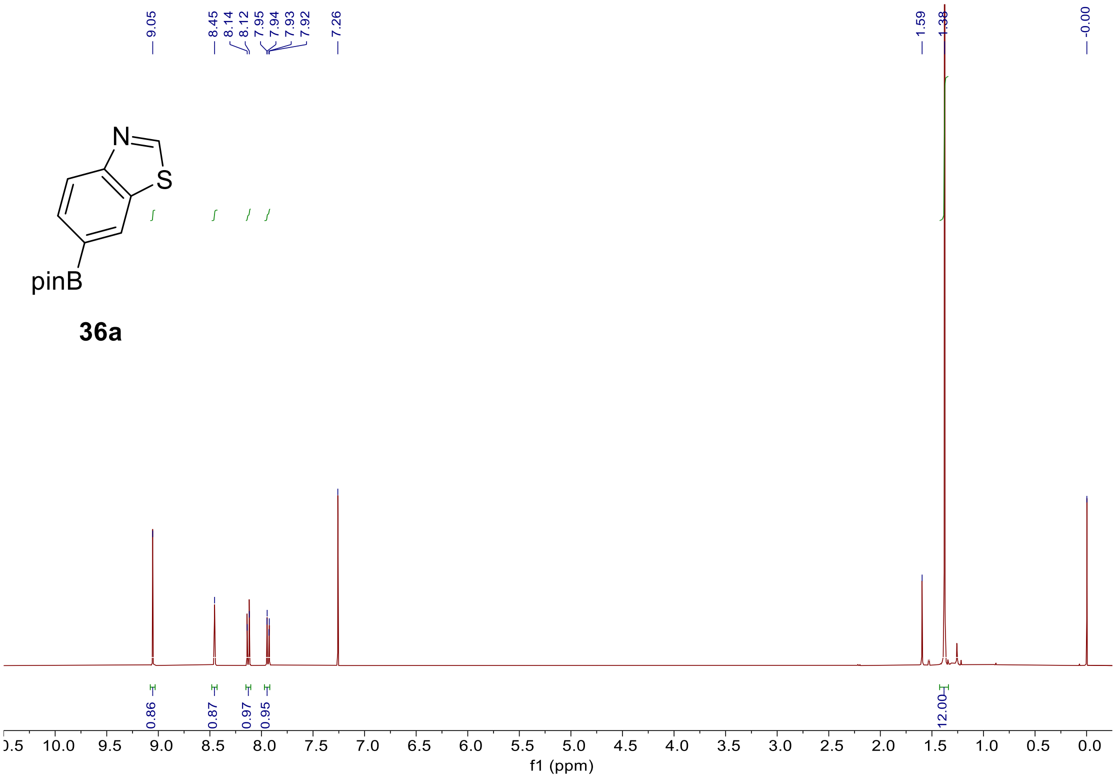

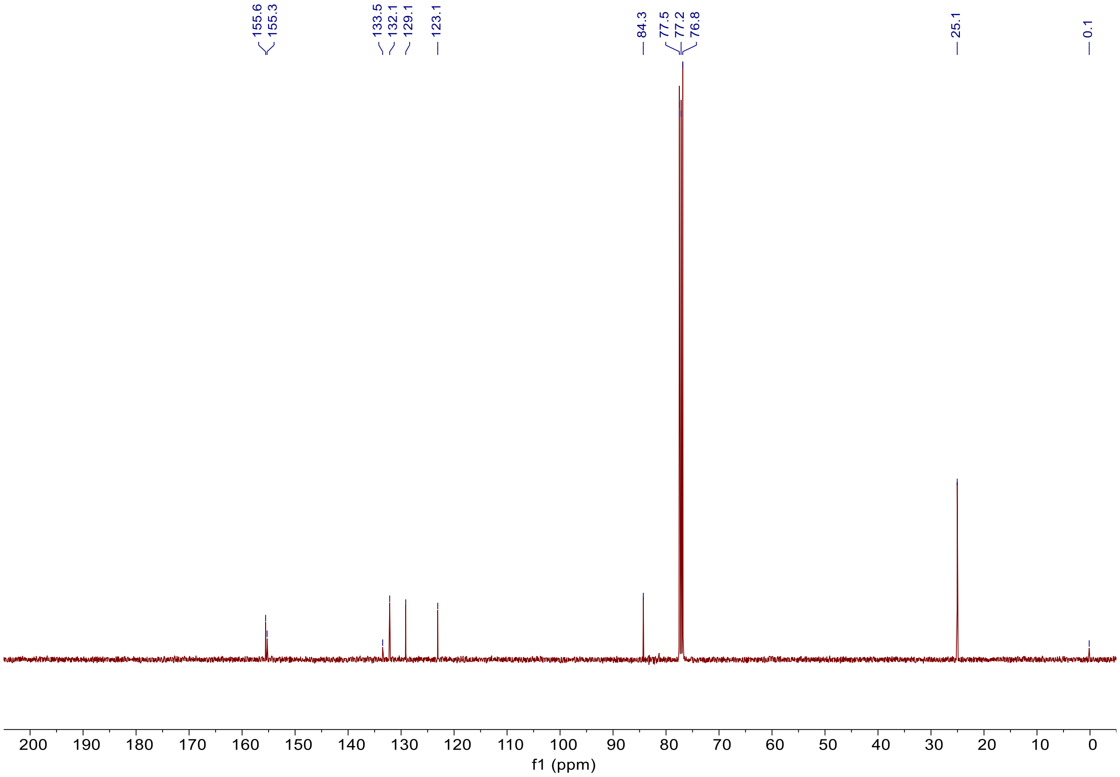

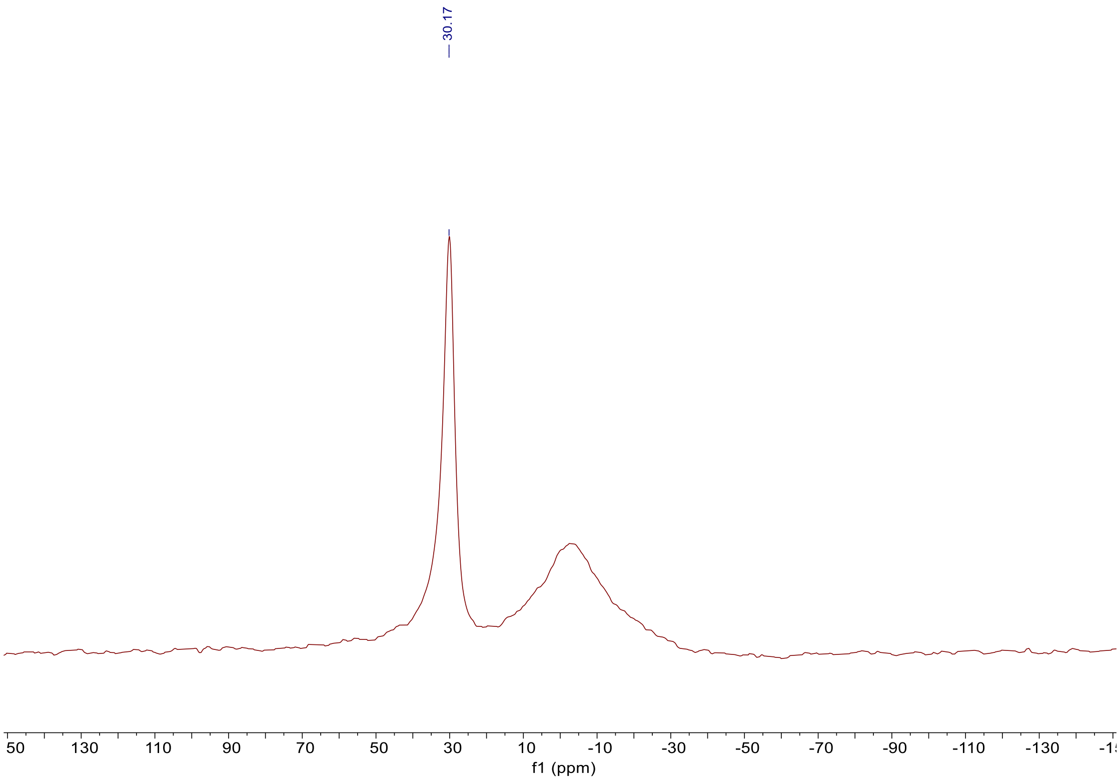
30.17
50
130
110
90
70
50
30
10
-10
-30
-50
-70
-90
-110
-130
f1 (ppm)

400 MHz ^{1}H NMR spectrum; 100.6 MHz ^{13}C NMR spectrum; 128 MHz ^{11}B NMR spectrum; DMSO-d_6 of **37a•HCl**

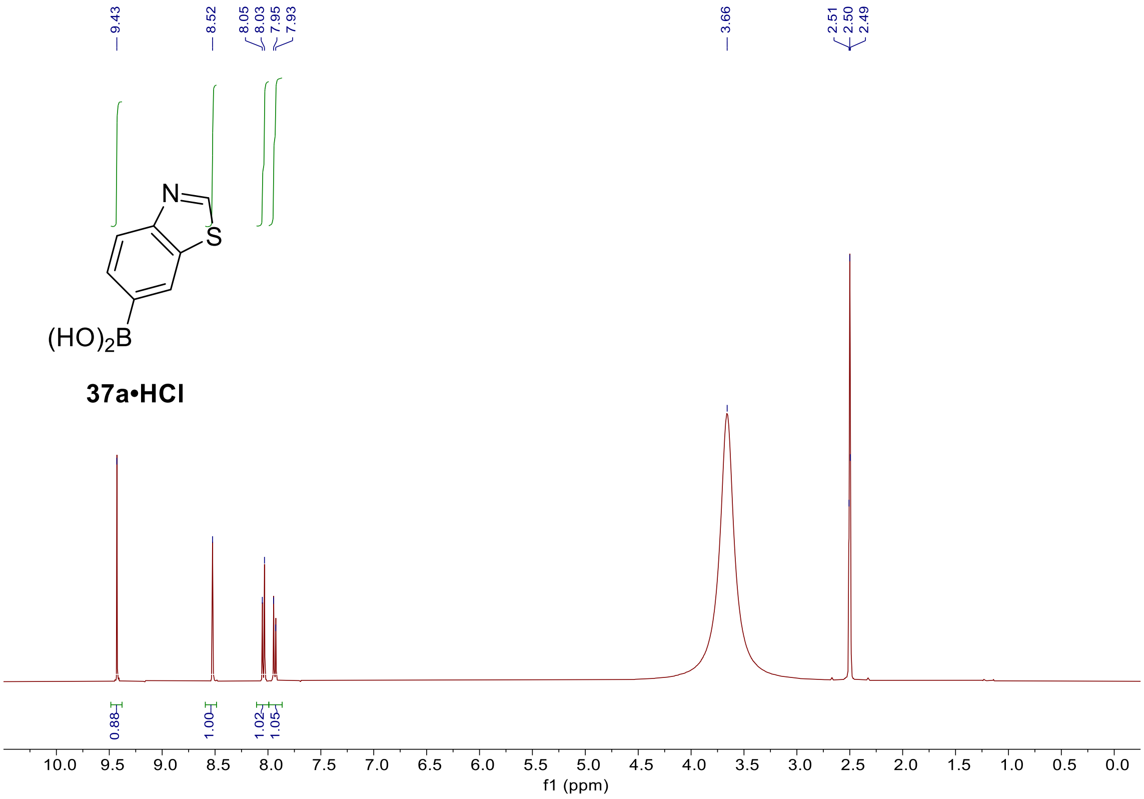

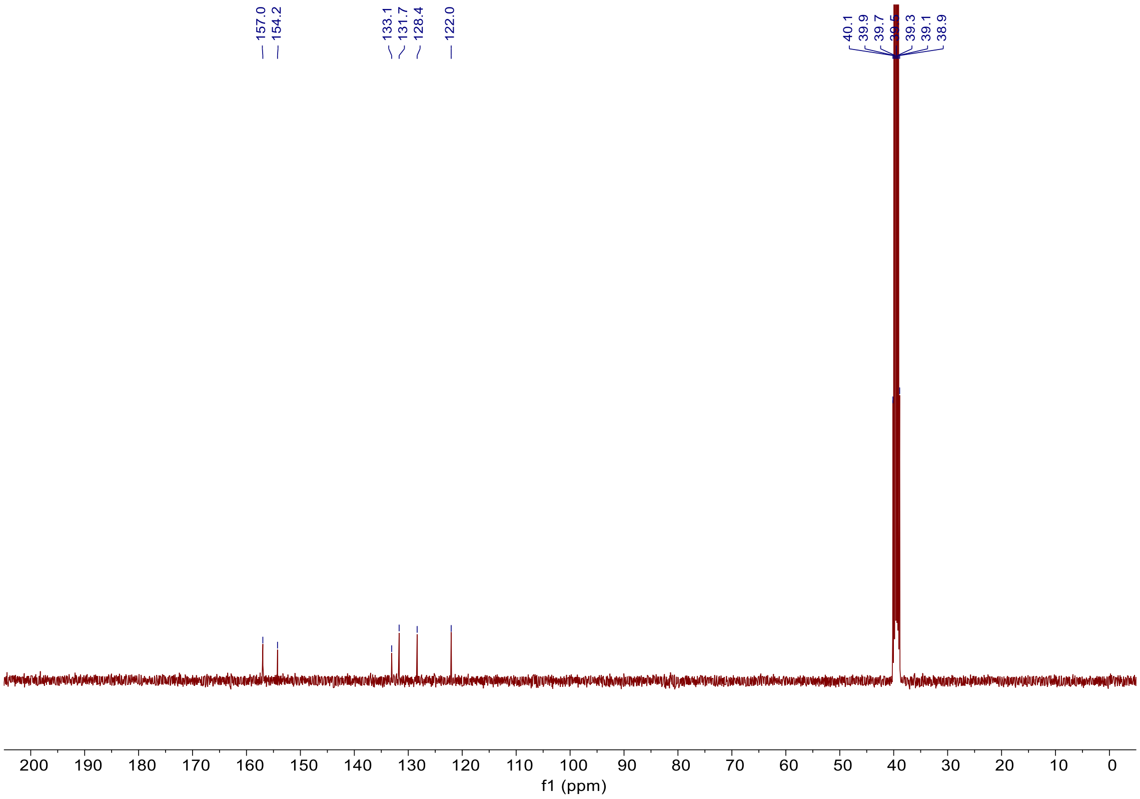

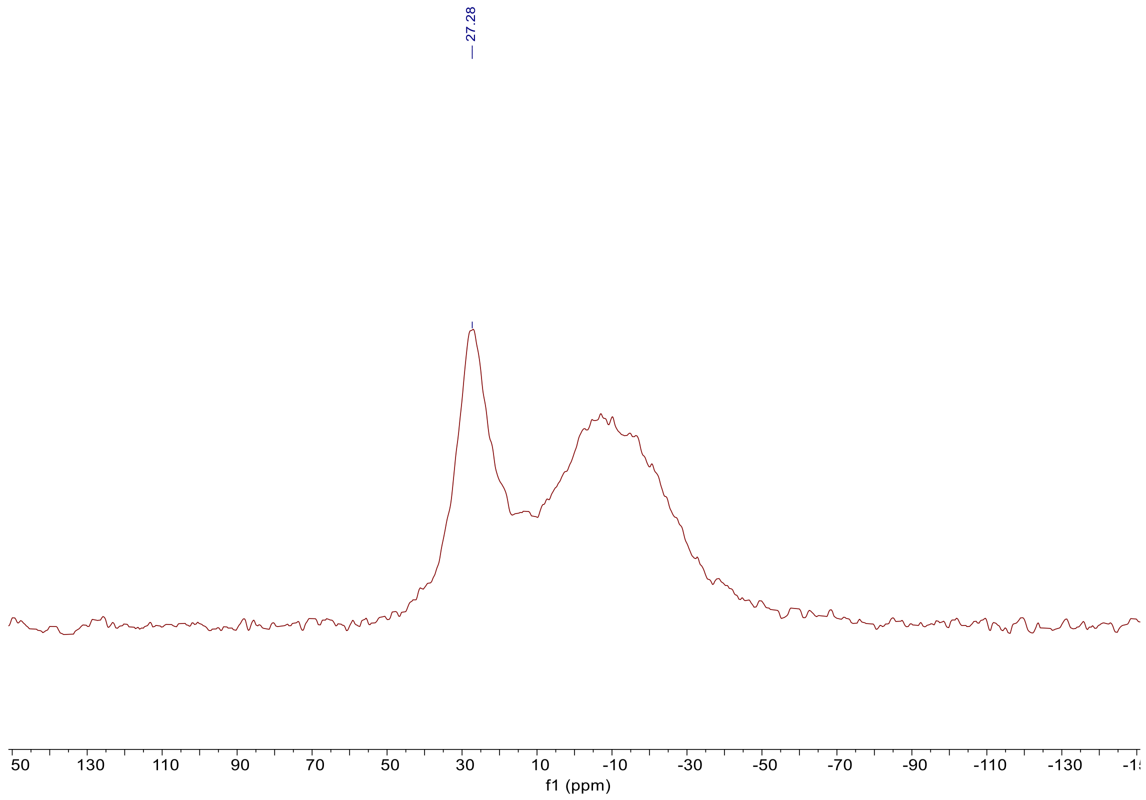
27.28
50
130
110
90
70
50
30
10
-10
-30
-50
-70
-90
-110
-130
f1 (ppm)

400 MHz ^{1}H NMR spectrum; $CDCl_3$ of *trans*-**22a** (*trans*-**22a** : *cis*-**22a** = 76:24)

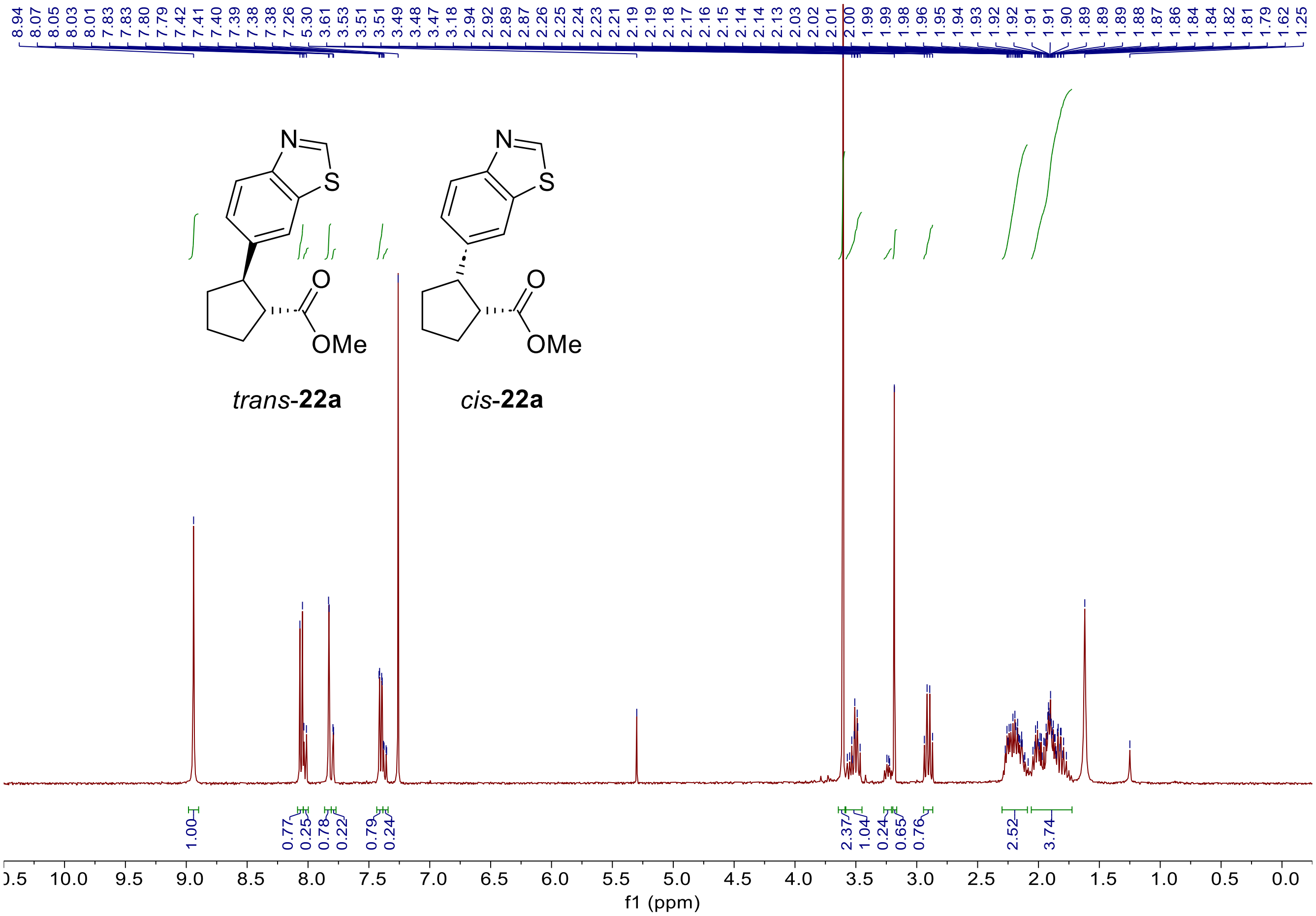

400 MHz ^{1}H NMR spectrum; 100.6 MHz ^{13}C NMR spectrum; 128 MHz ^{11}B NMR spectrum; $CDCl_3$ of **36b**

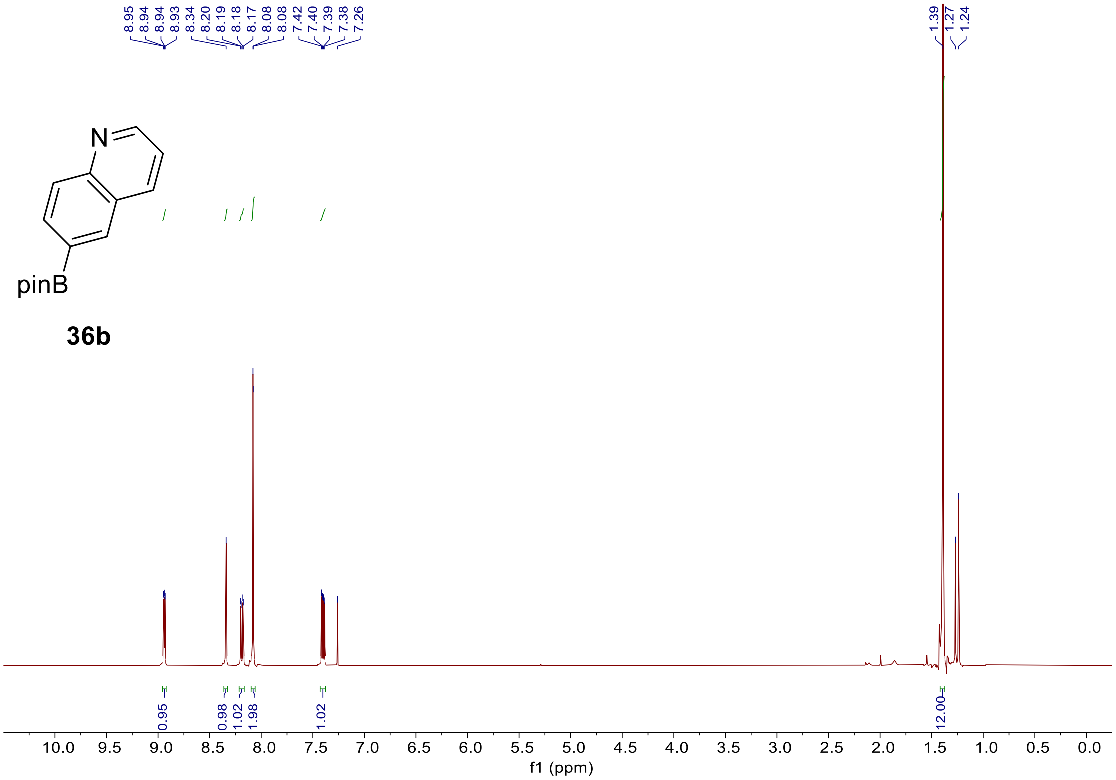

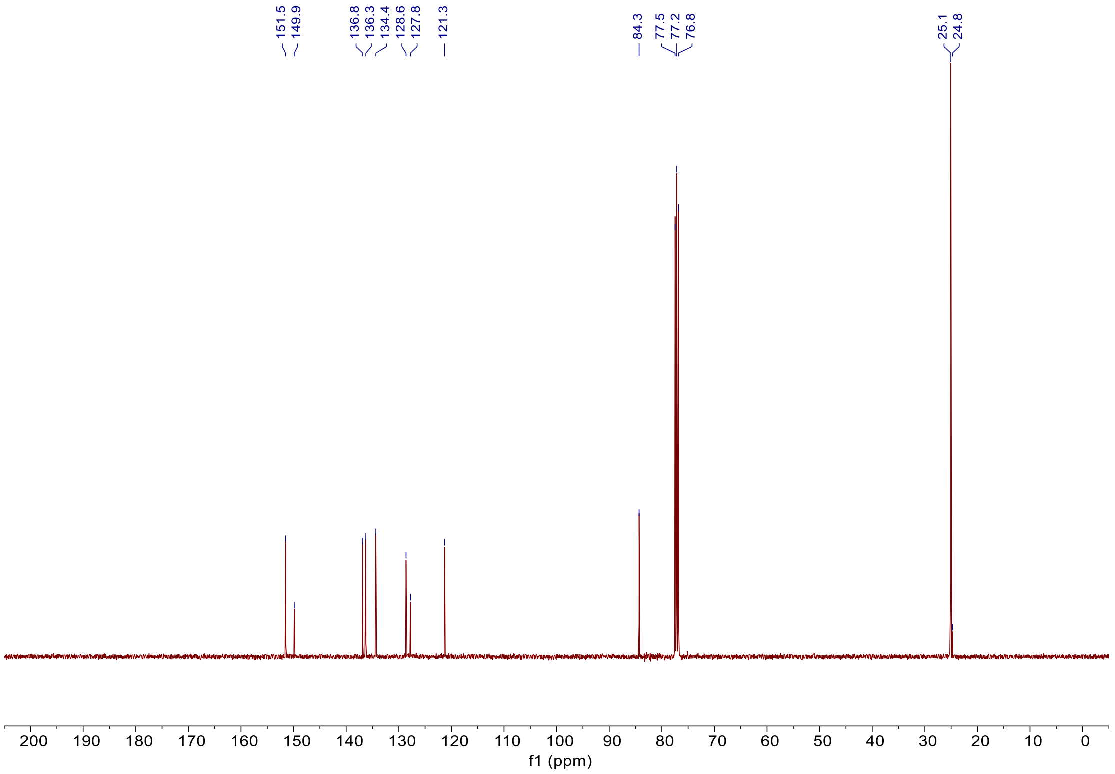

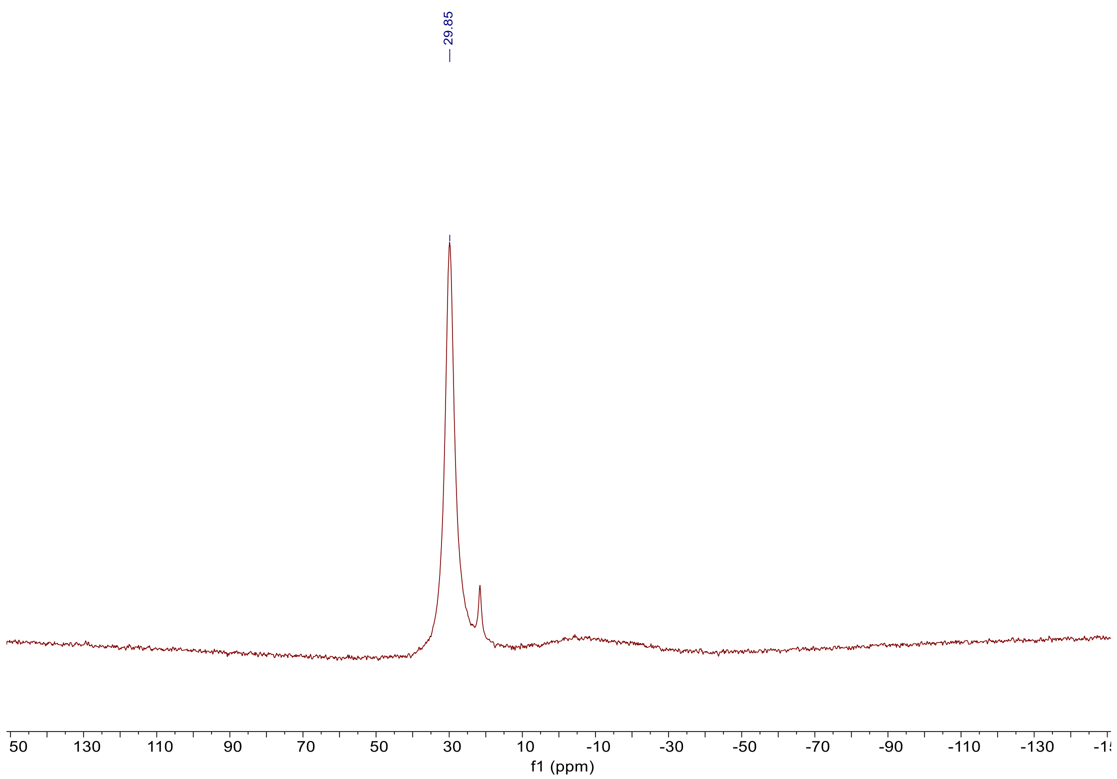
29.85
50
130
110
90
70
50
30
10
-10
-30
-50
-70
-90
-110
-130
f1 (ppm)

400 MHz ^{1}H NMR spectrum; 100.6 MHz ^{13}C NMR spectrum; 128 MHz ^{11}B NMR spectrum; DMSO-d_6 of **37b•HCl**

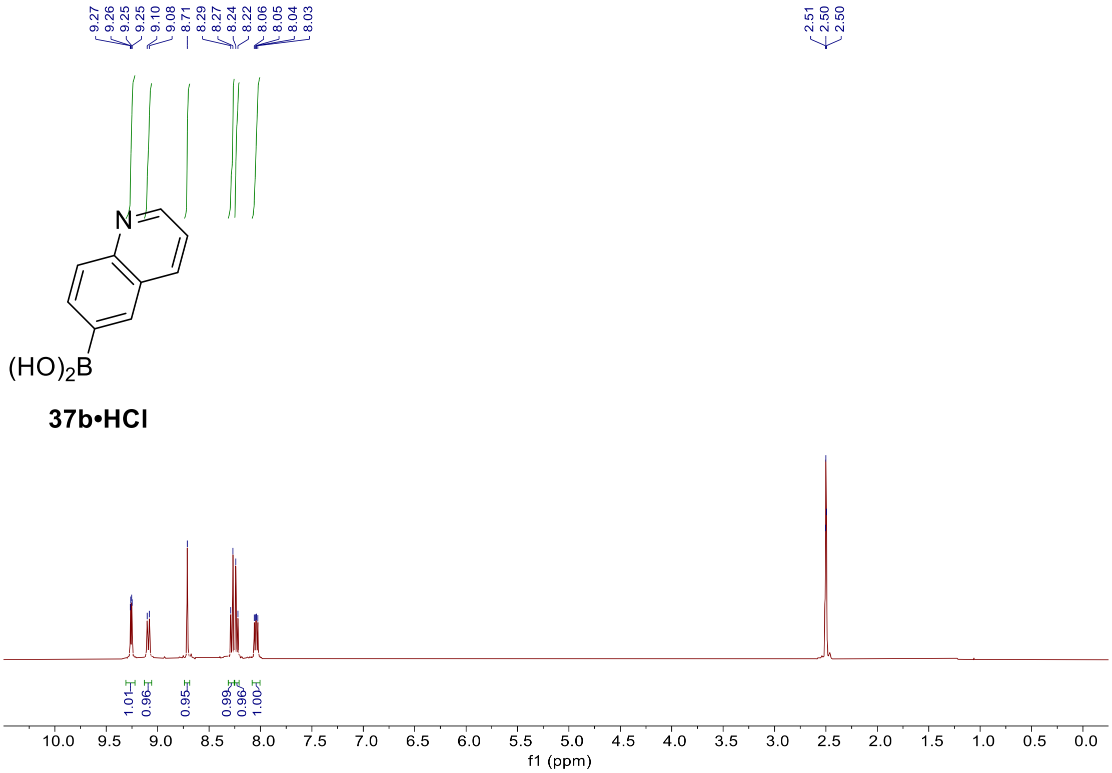

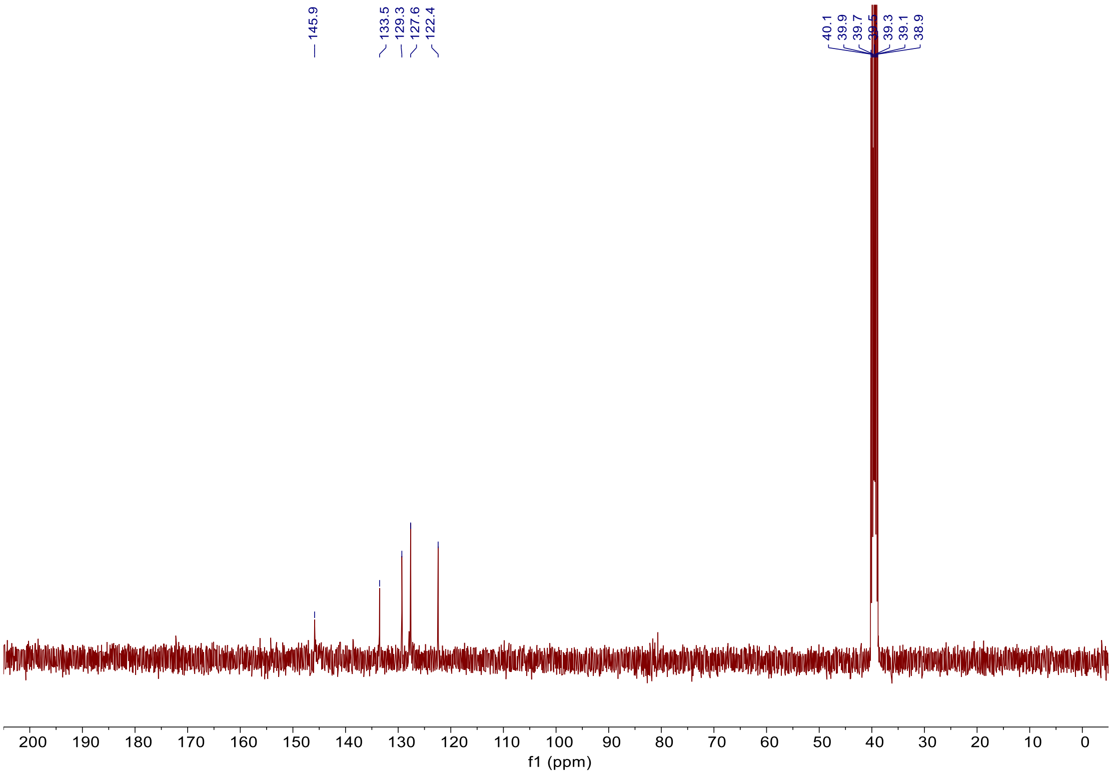

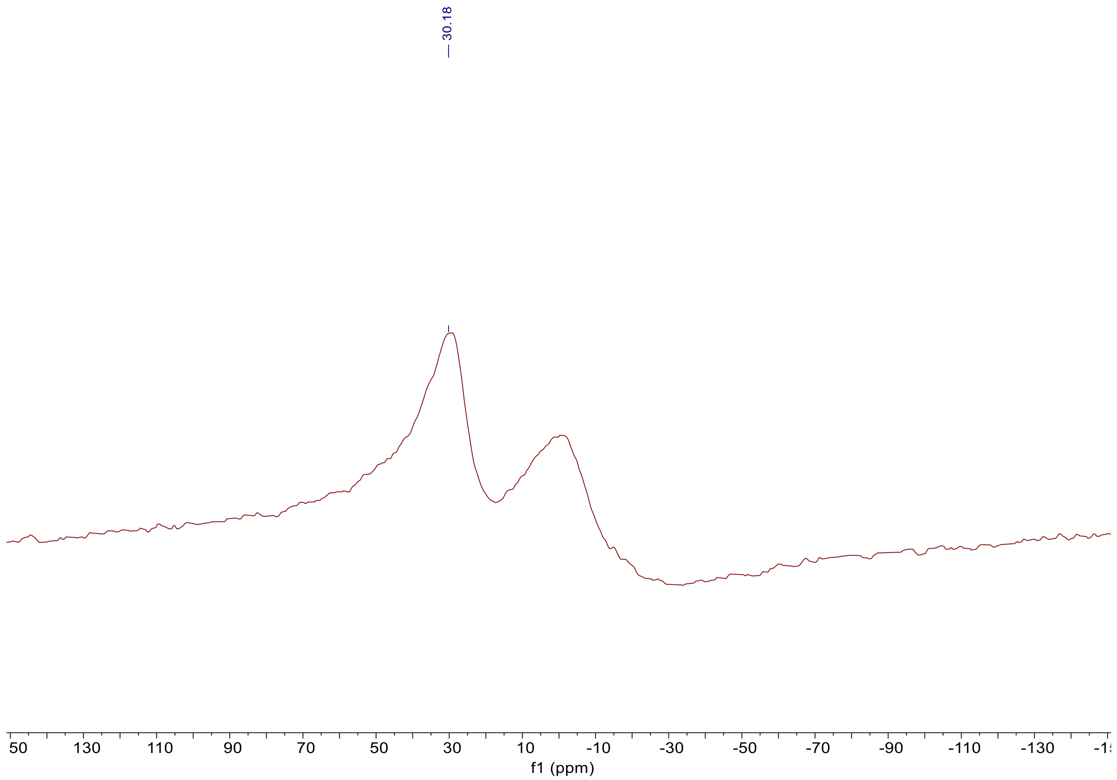
30.18
50
130
110
90
70
50
30
10
-10
-30
-50
-70
-90
-110
-130
f1 (ppm)

400 MHz ^{1}H NMR spectrum; $CDCl_3$ of *trans*-**22c** (*trans*-**22c** : *cis*-**22c** = 65:35)

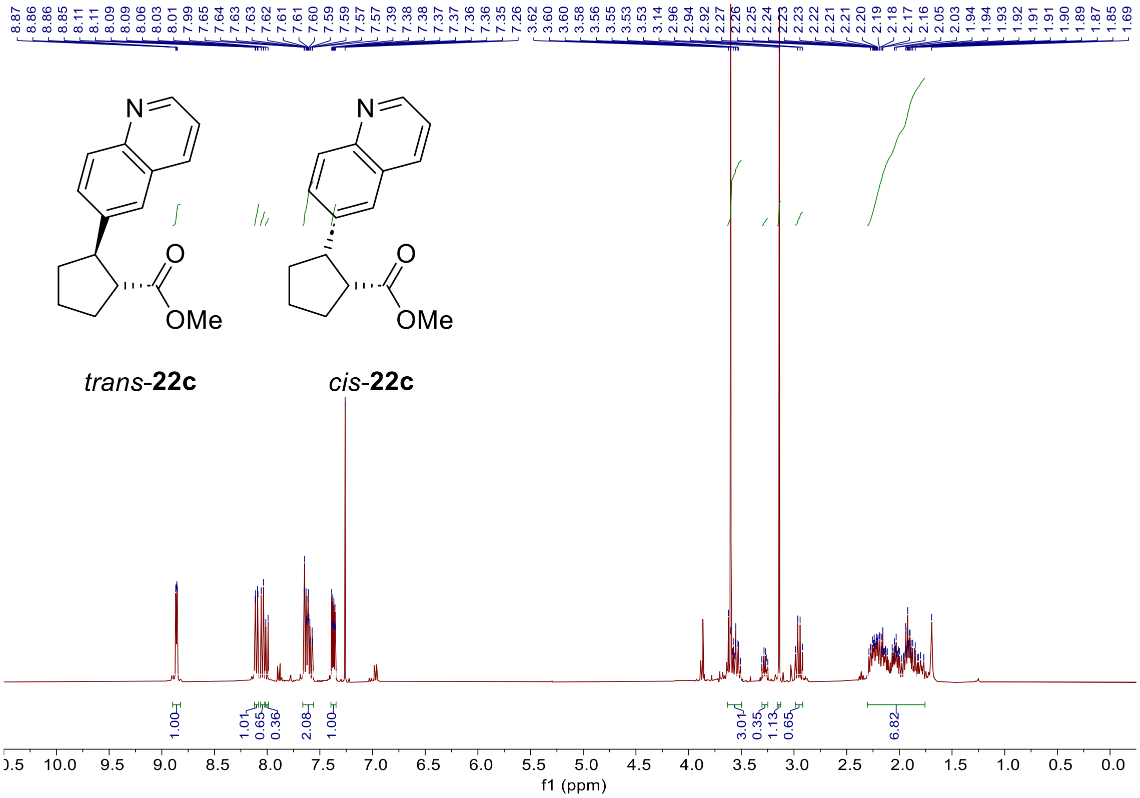

400 MHz ^{1}H NMR spectrum; 100.6 MHz ^{13}C NMR spectrum; 128 MHz ^{11}B NMR spectrum; $CDCl_3$ of **36c**

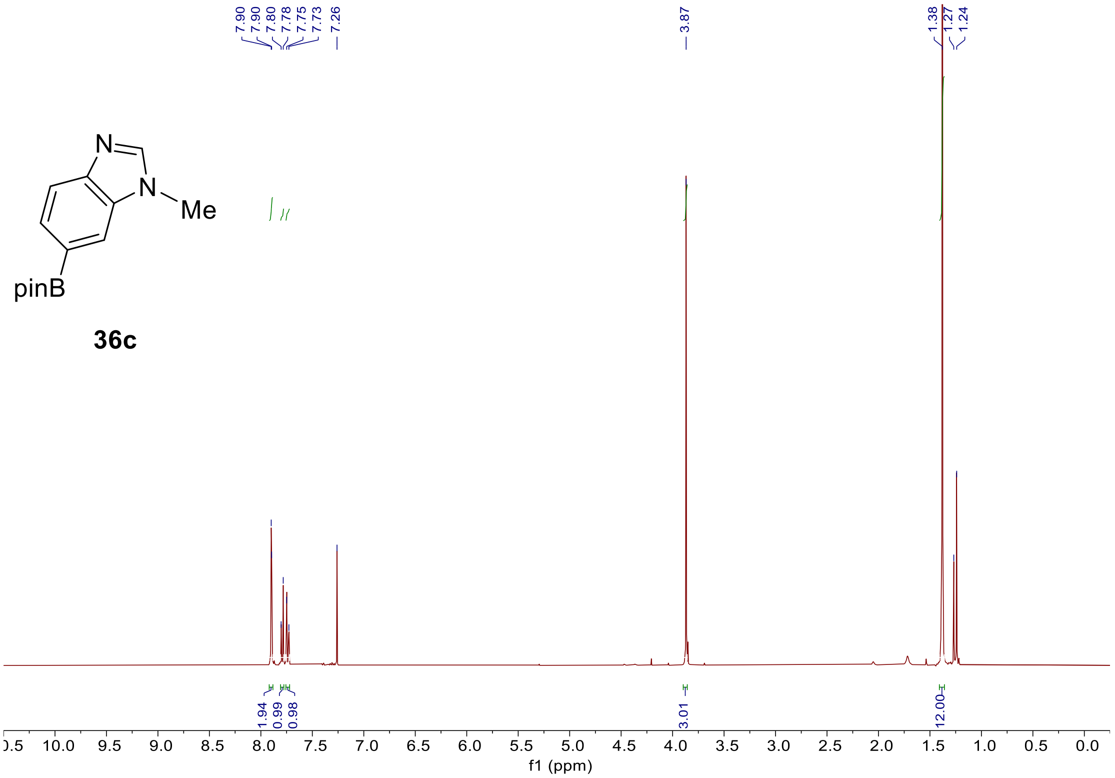

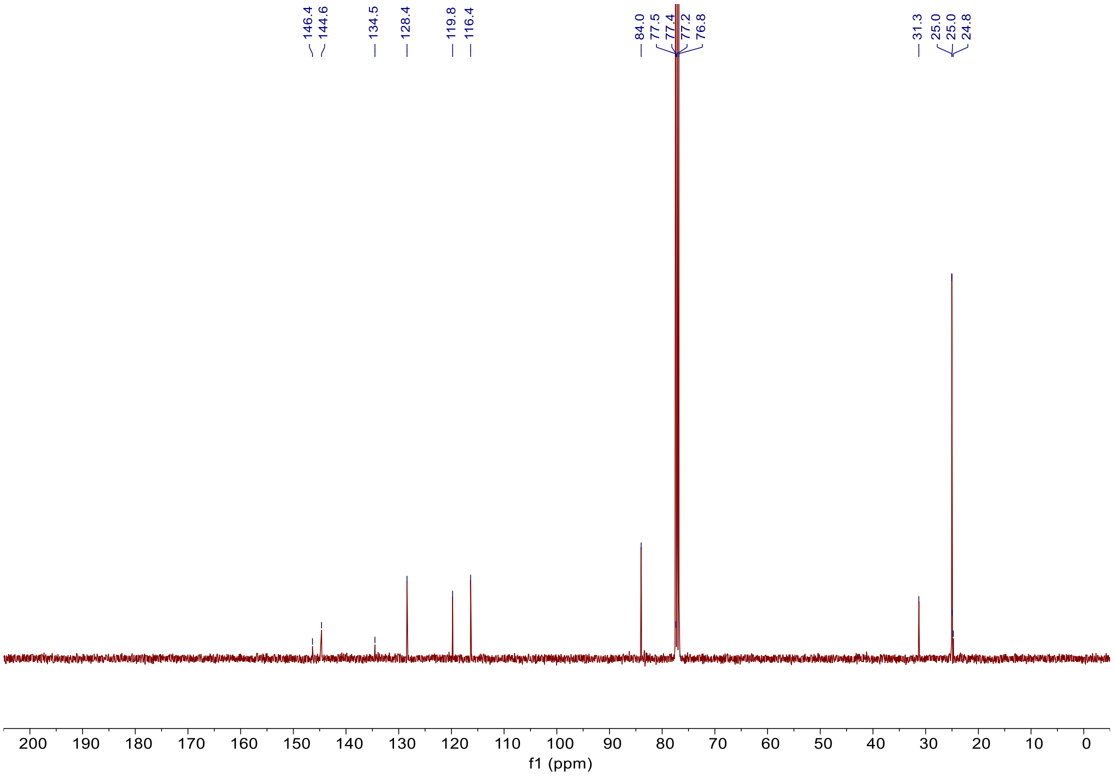

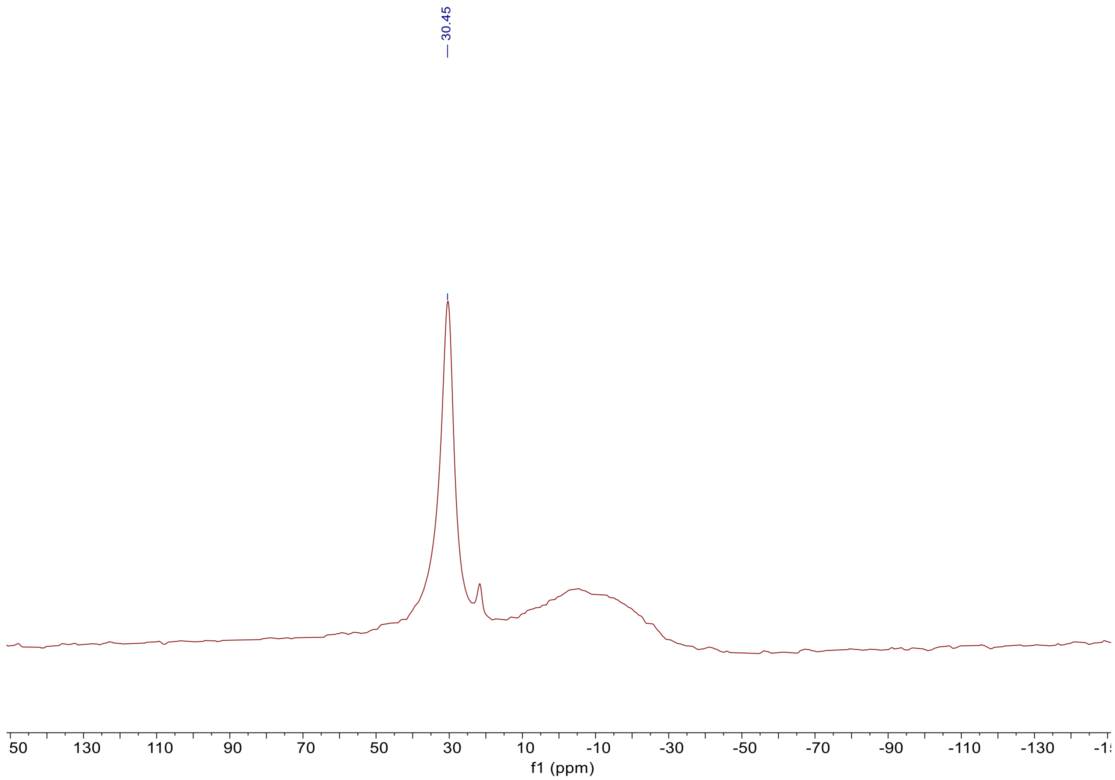

30.45
50
130
110
90
70
50
30
10
-10
-30
-50
-70
-90
-110
-130
f1 (ppm)

400 MHz ^{1}H NMR spectrum; 100.6 MHz ^{13}C NMR spectrum; 128 MHz ^{11}B NMR spectrum; DMSO-d_6 of **37c•HCl**

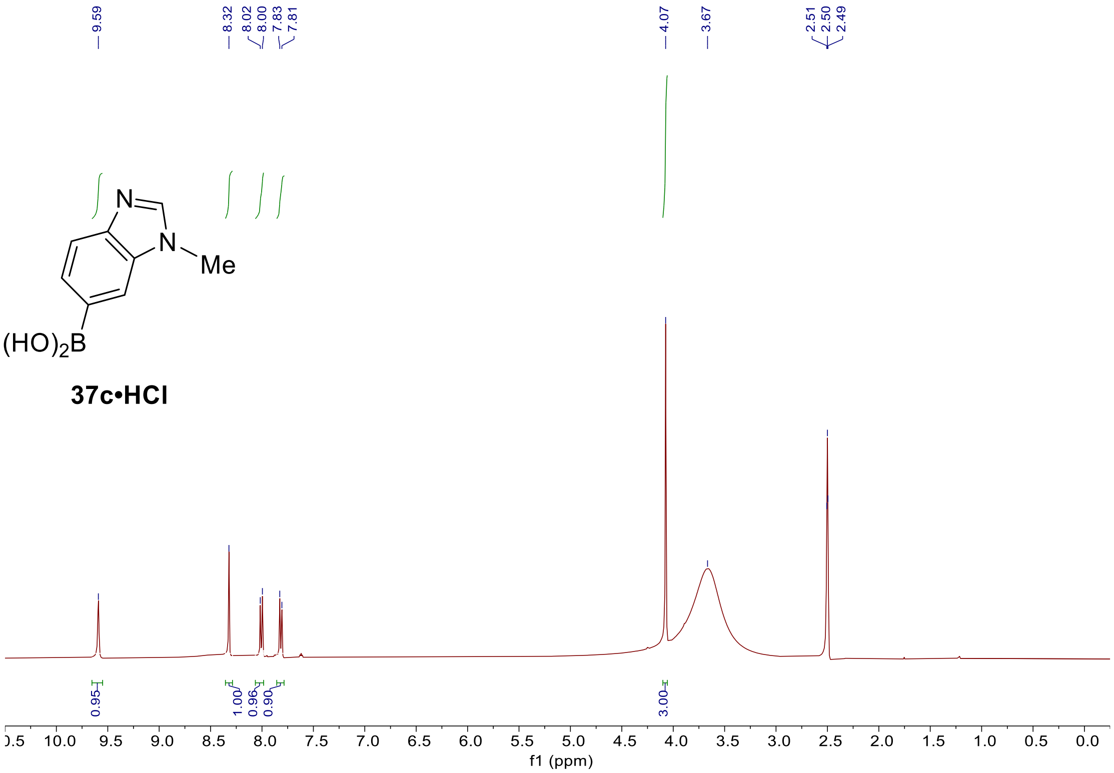

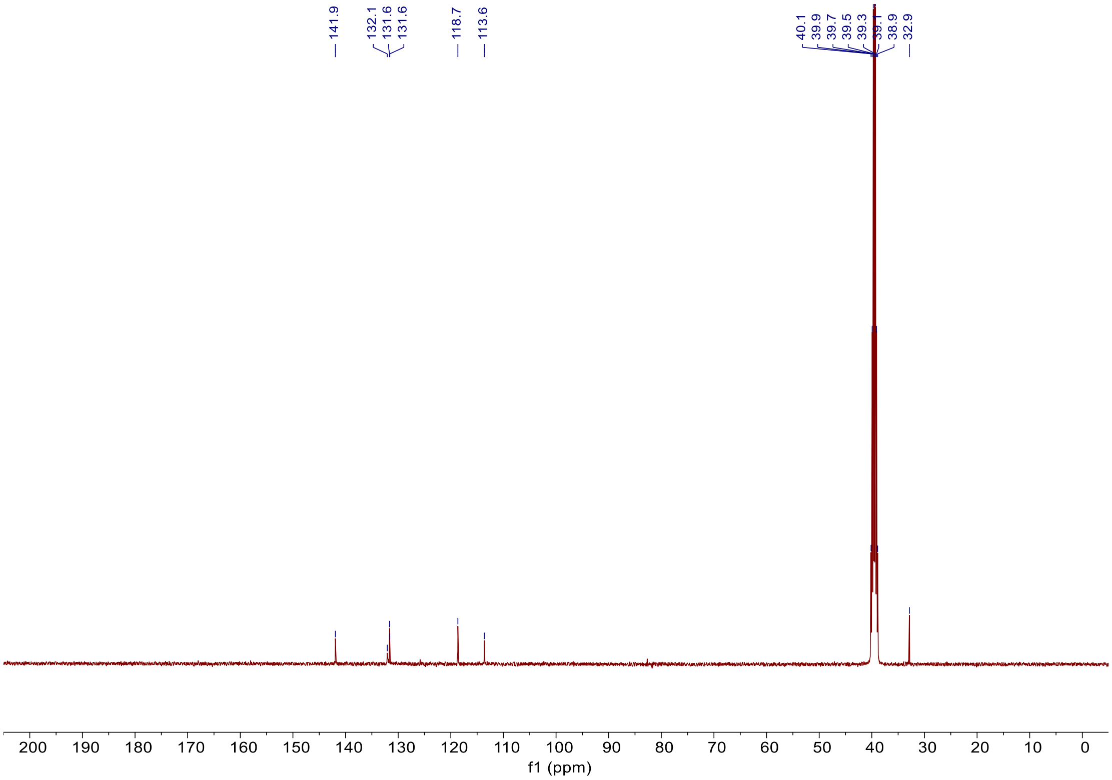

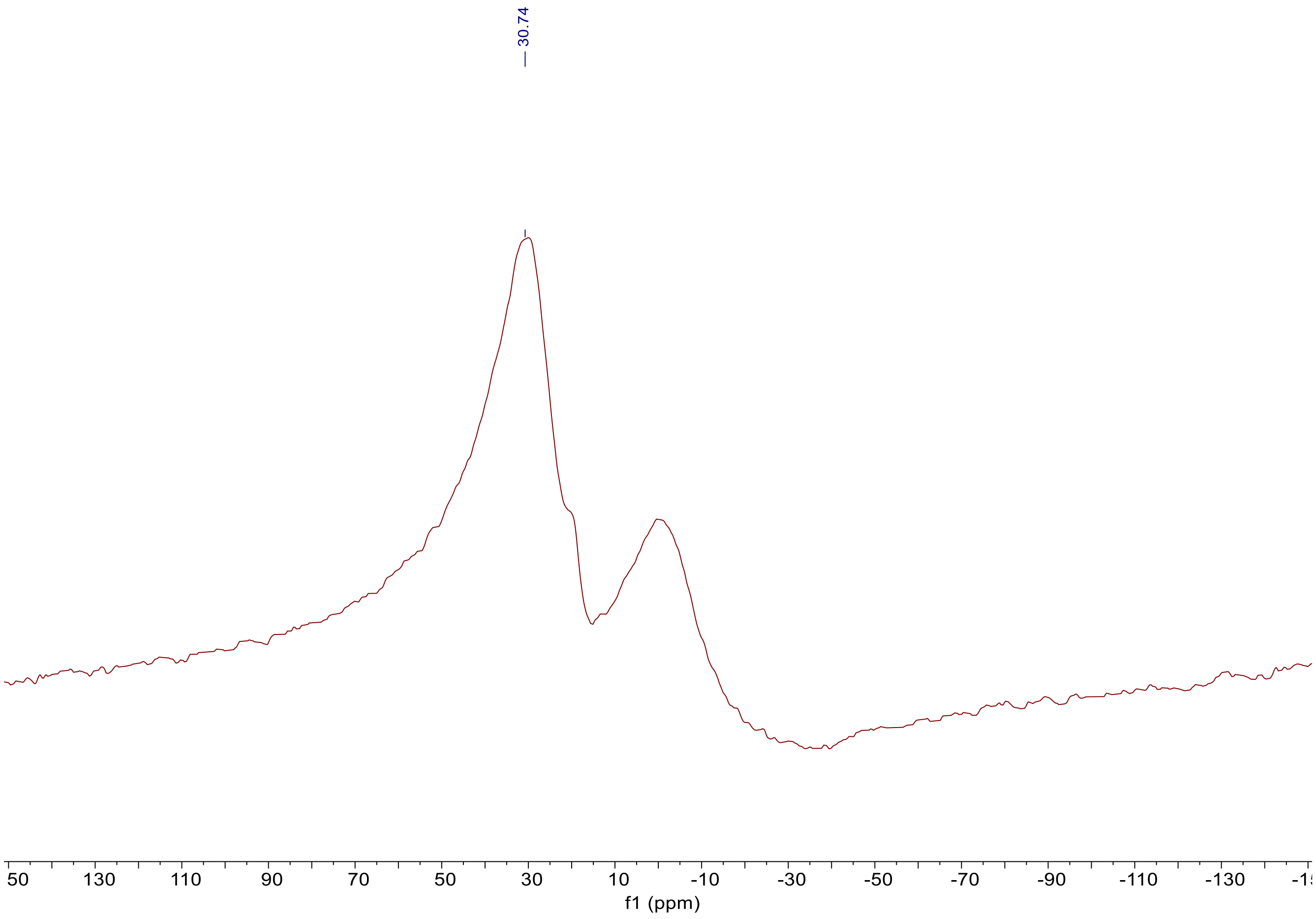
30.74
50
130
110
90
70
50
30
10
-10
-30
-50
-70
-90
-110
-130
f1 (ppm)

400 MHz ^{1}H NMR spectrum; $CDCl_3$ of *trans*-**22d** (1-methyl-*1H*-benzo[*d*]imidazole: *trans*-**22d** : *cis*-**22d** = 73:22:5)

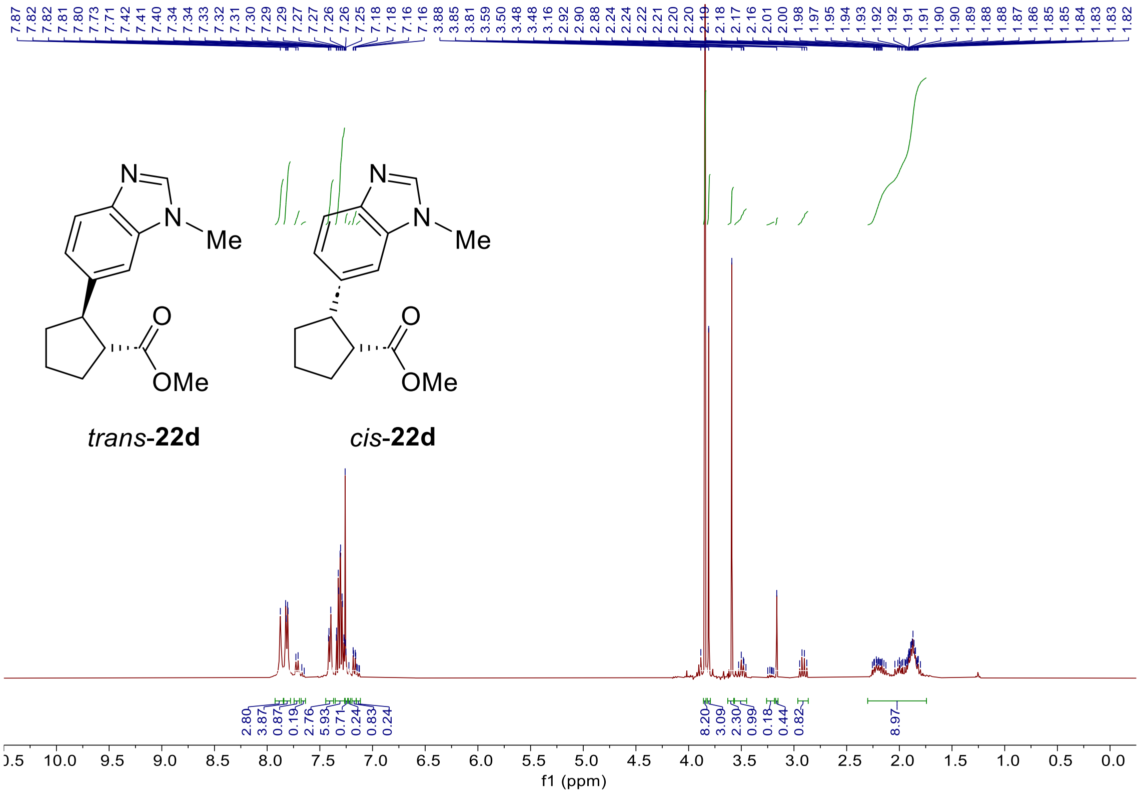

400 MHz ^{1}H NMR spectrum; 100.6 MHz ^{13}C NMR spectrum; 128 MHz ^{11}B NMR spectrum; $CDCl_3$ of **36d**

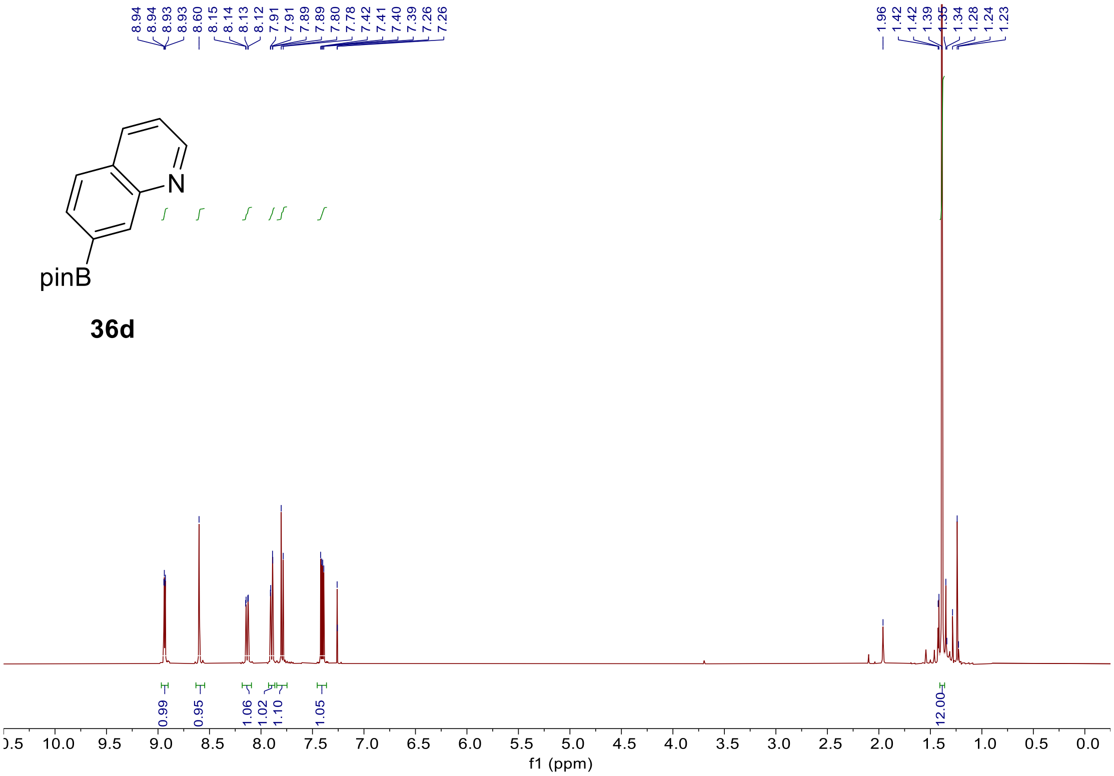

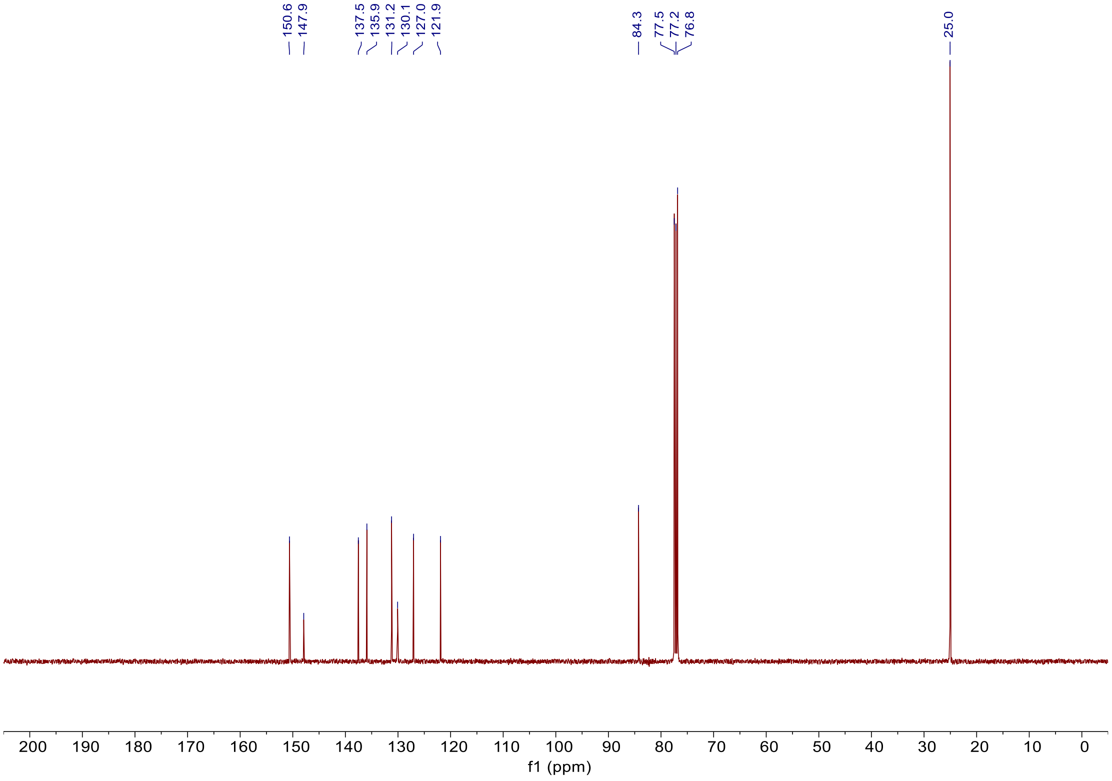

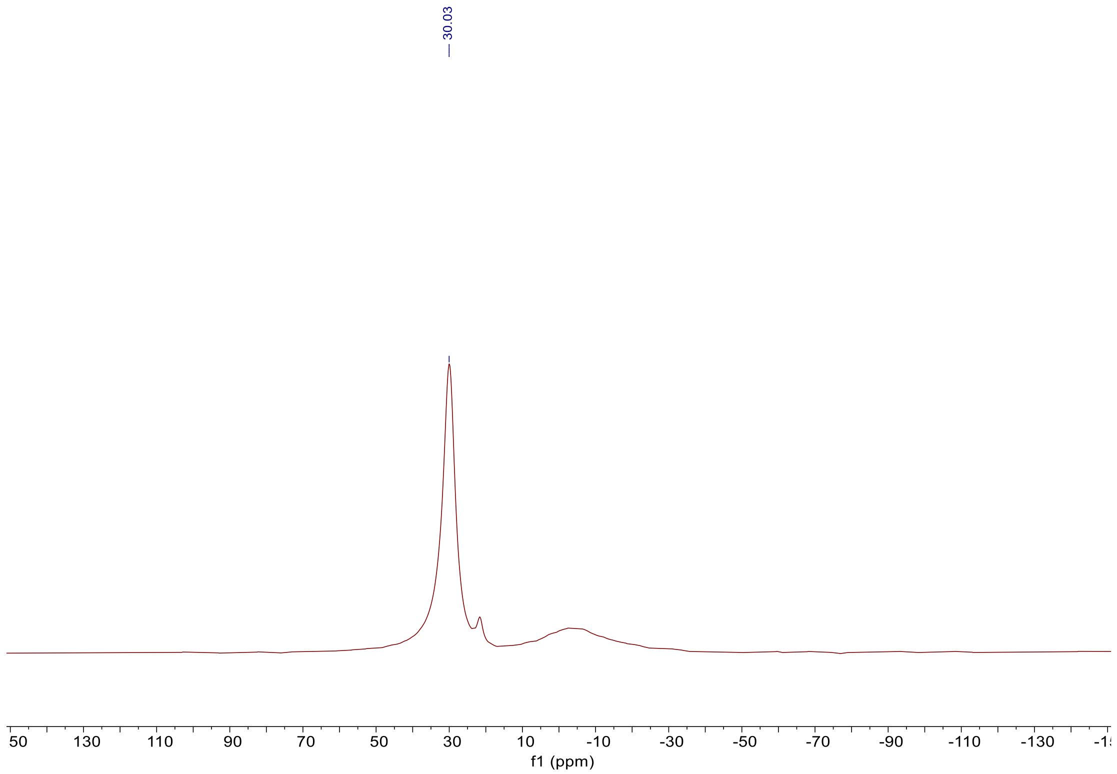
30.03
50
130
110
90
70
50
30
10
-10
-30
-50
-70
-90
-110
-130
f1 (ppm)

400 MHz ^{1}H NMR spectrum; 100.6 MHz ^{13}C NMR spectrum; 128 MHz ^{11}B NMR spectrum; DMSO-d_6 of **37d•HCl**

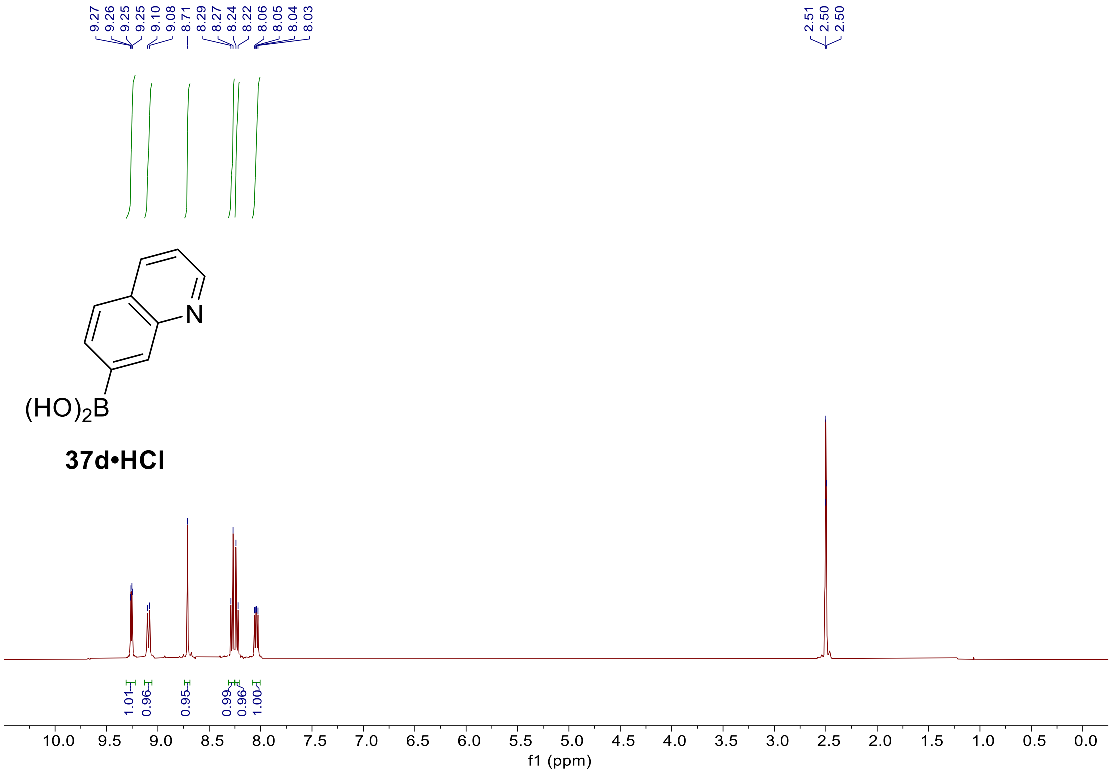

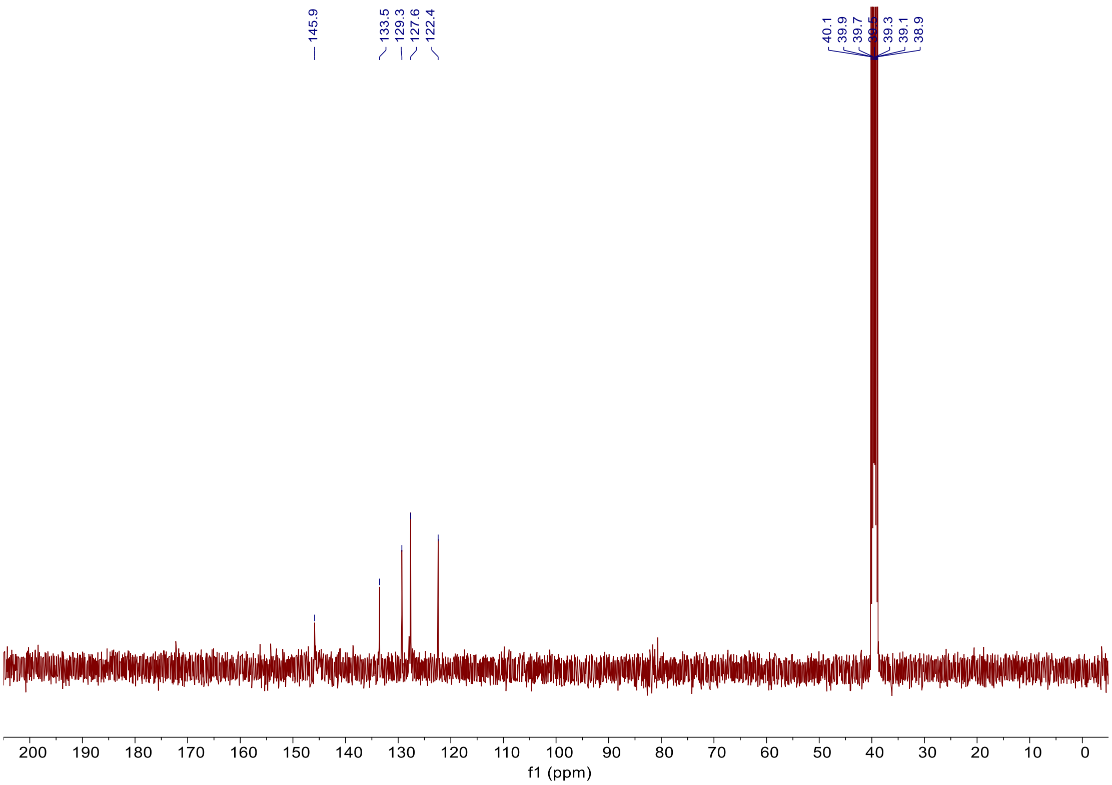

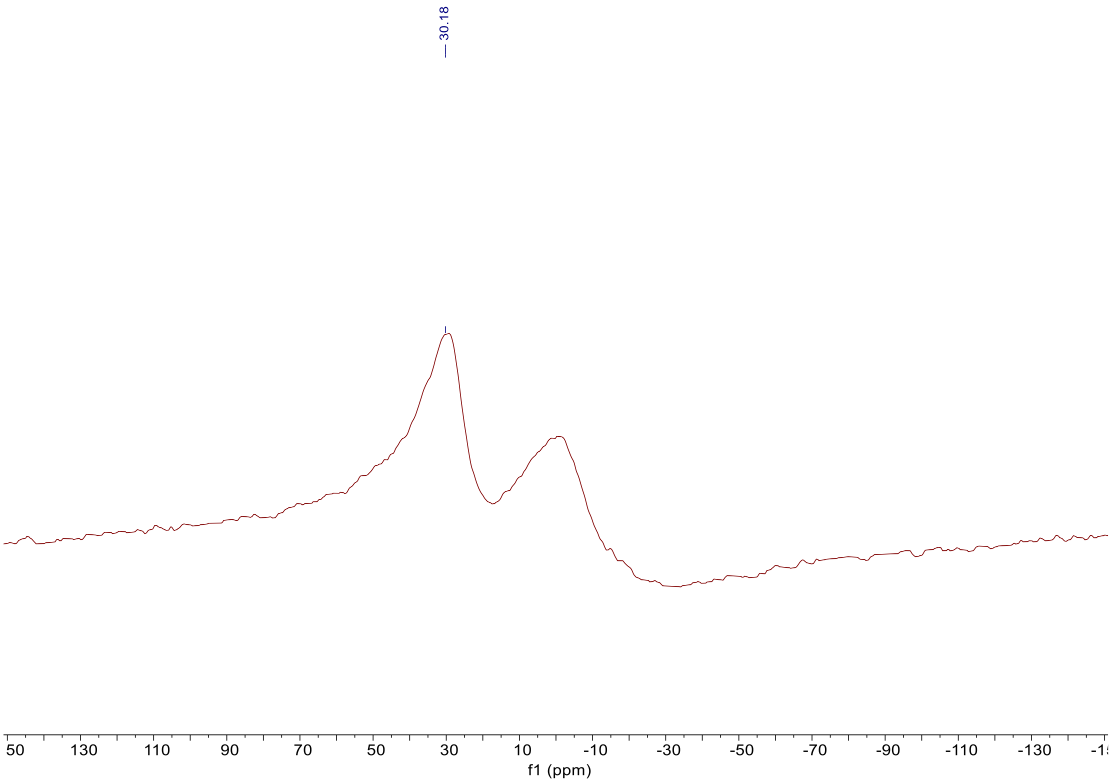
30.18
50
130
110
90
70
50
30
10
-10
-30
-50
-70
-90
-110
-130
f1 (ppm)

400 MHz ^{1}H NMR spectrum; $CDCl_3$ of *cis*-**22e** (*trans*-**22e** : *cis*-**22e** = 35:65)

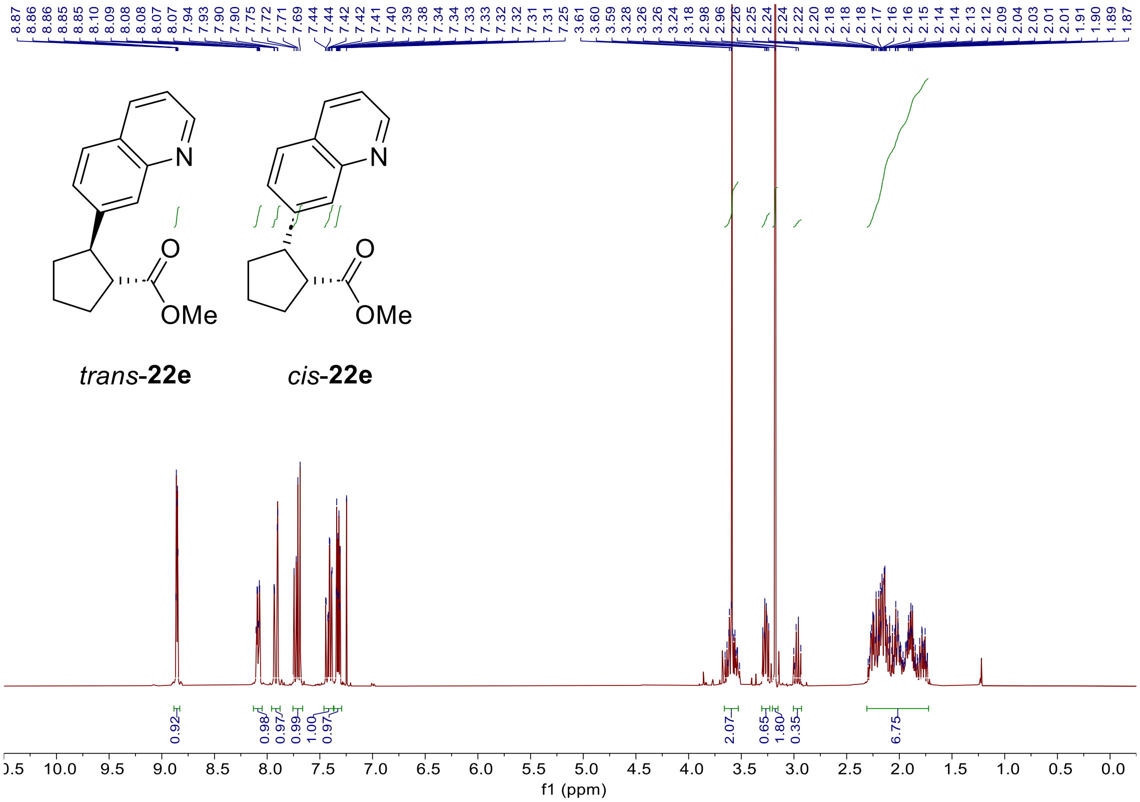

400 MHz ^{1}H NMR spectrum; 100.6 MHz ^{13}C NMR spectrum; $CDCl_3$of *trans*-**17a**

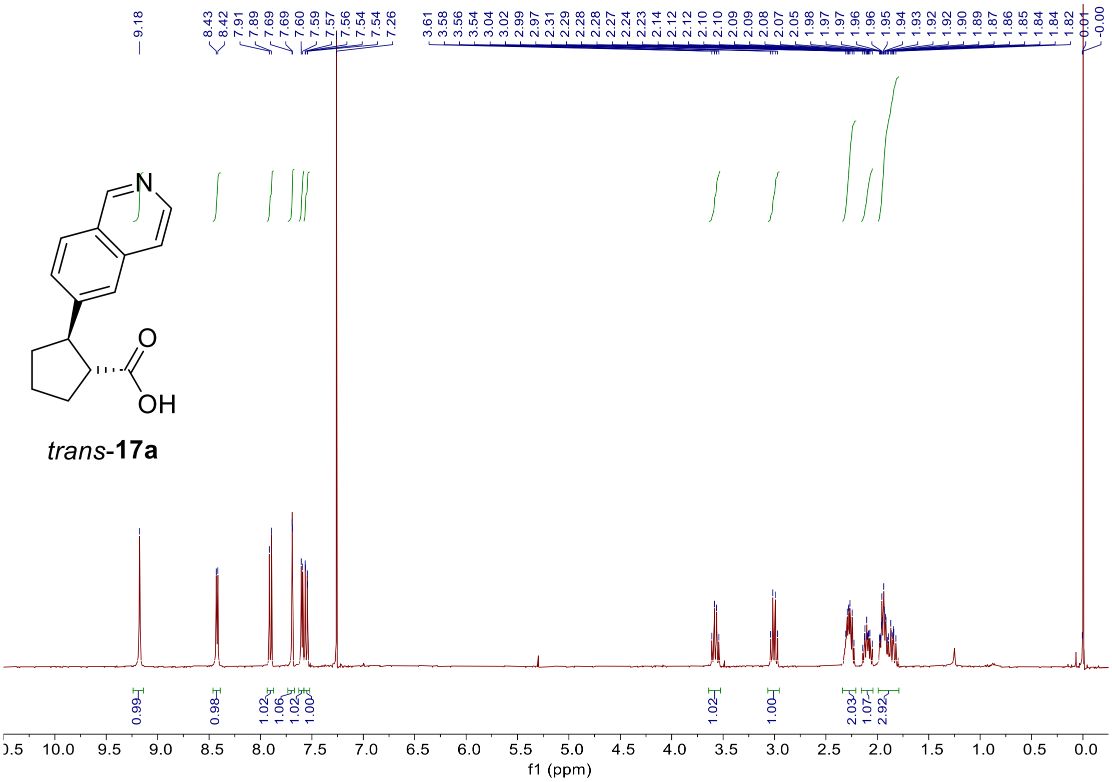

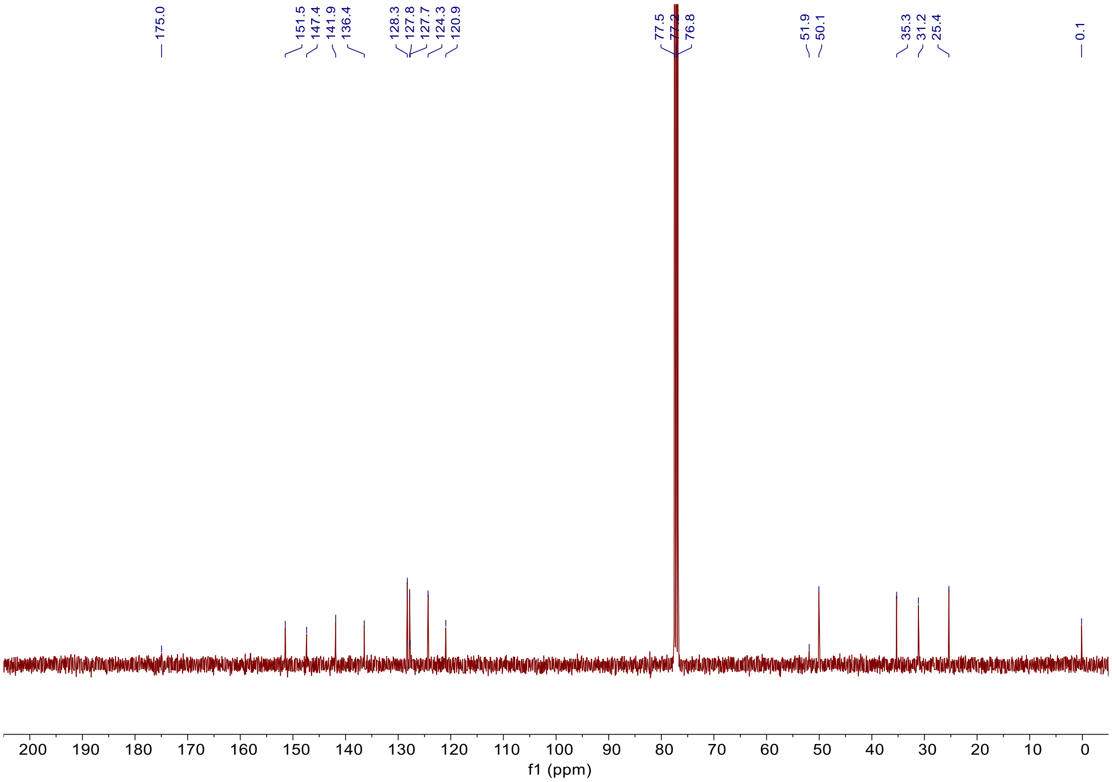

400 MHz ^{1}H NMR spectrum; 100.6 MHz ^{13}C NMR spectrum; DMSO-d_6 of *trans*-**17b**

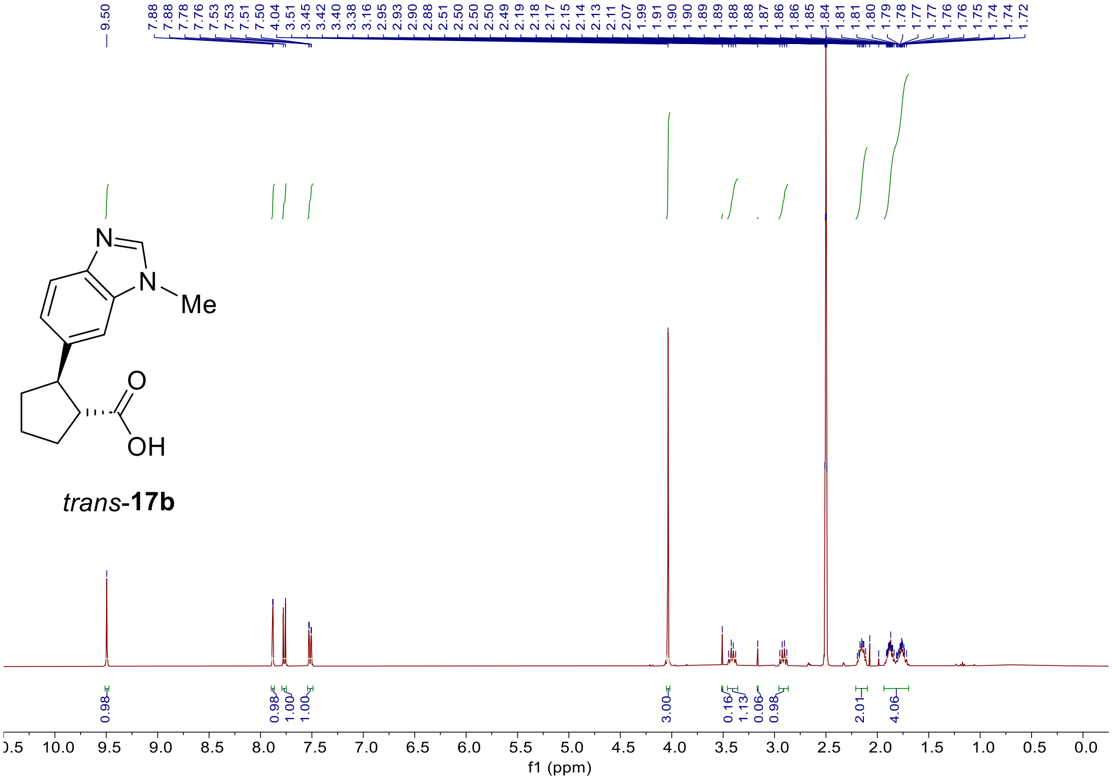

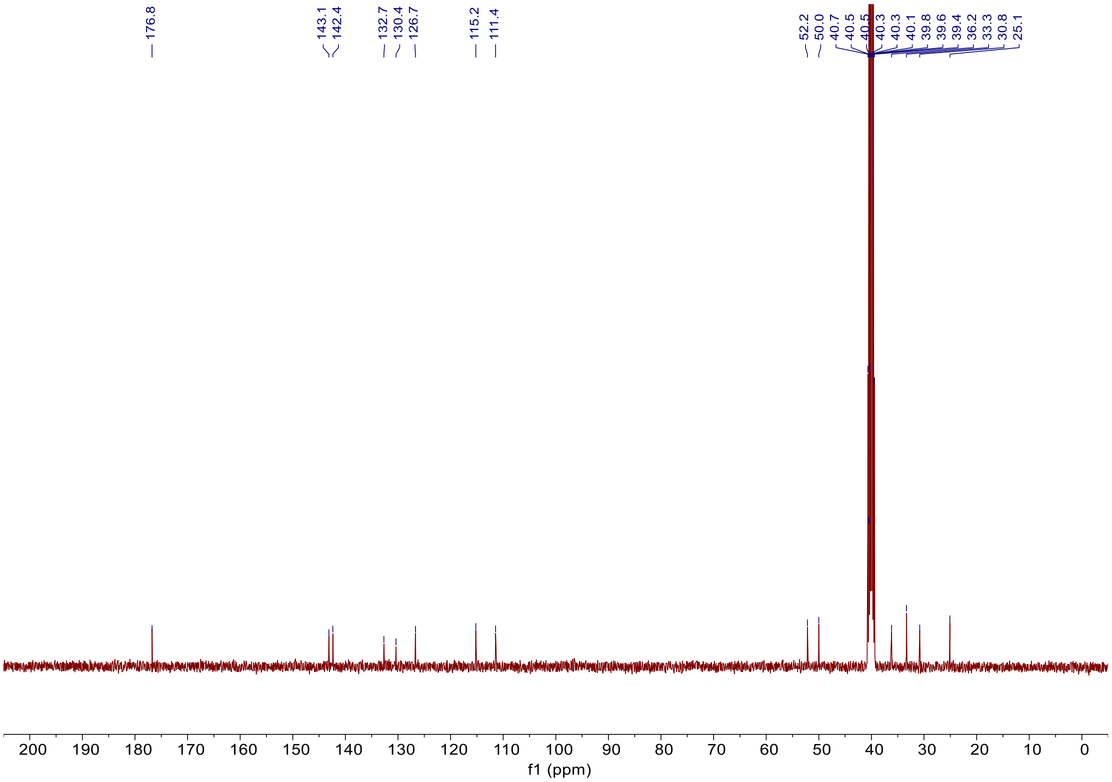

400 MHz ^{1}H NMR spectrum; 100.6 MHz ^{13}C NMR spectrum; $CDCl_3$ of *trans*-**17g**

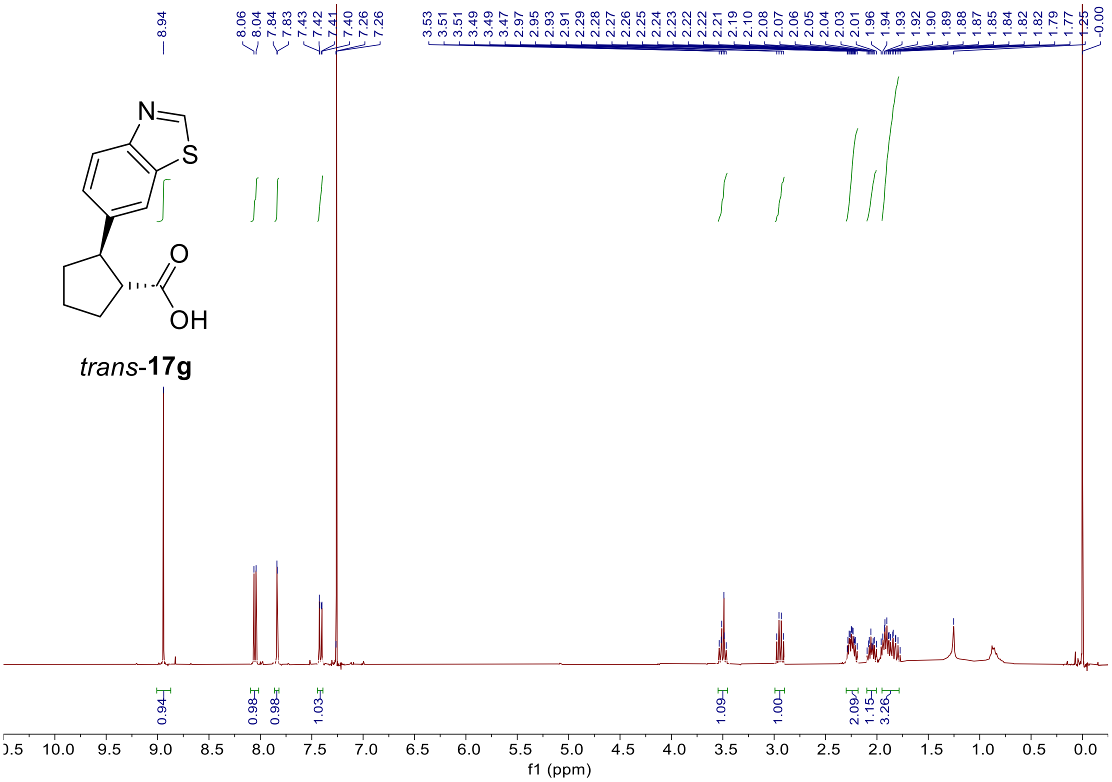

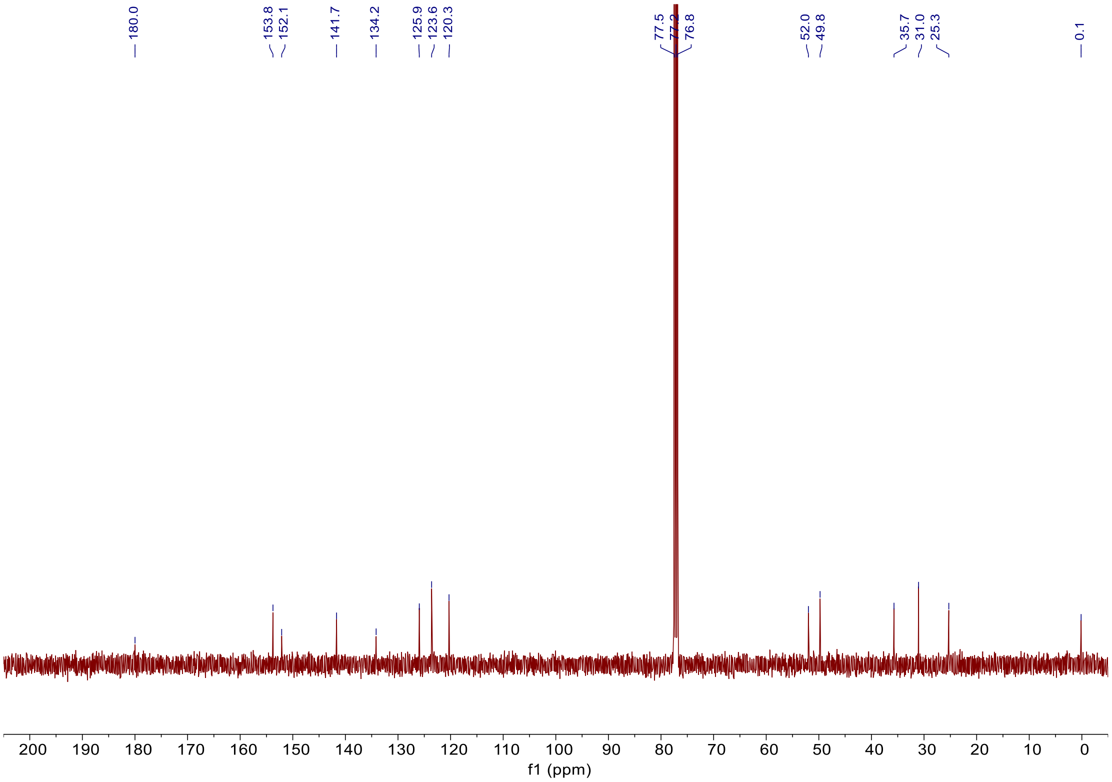

400 MHz ^{1}H NMR spectrum; 100.6 MHz ^{13}C NMR spectrum; $CDCl_3$ of *trans*-**17h**

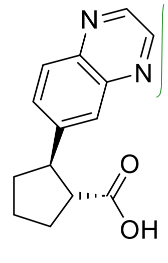

trans-**17h**

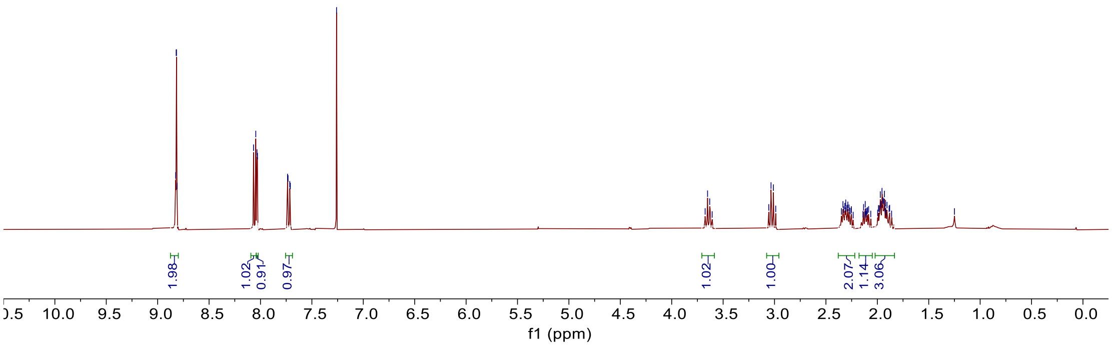

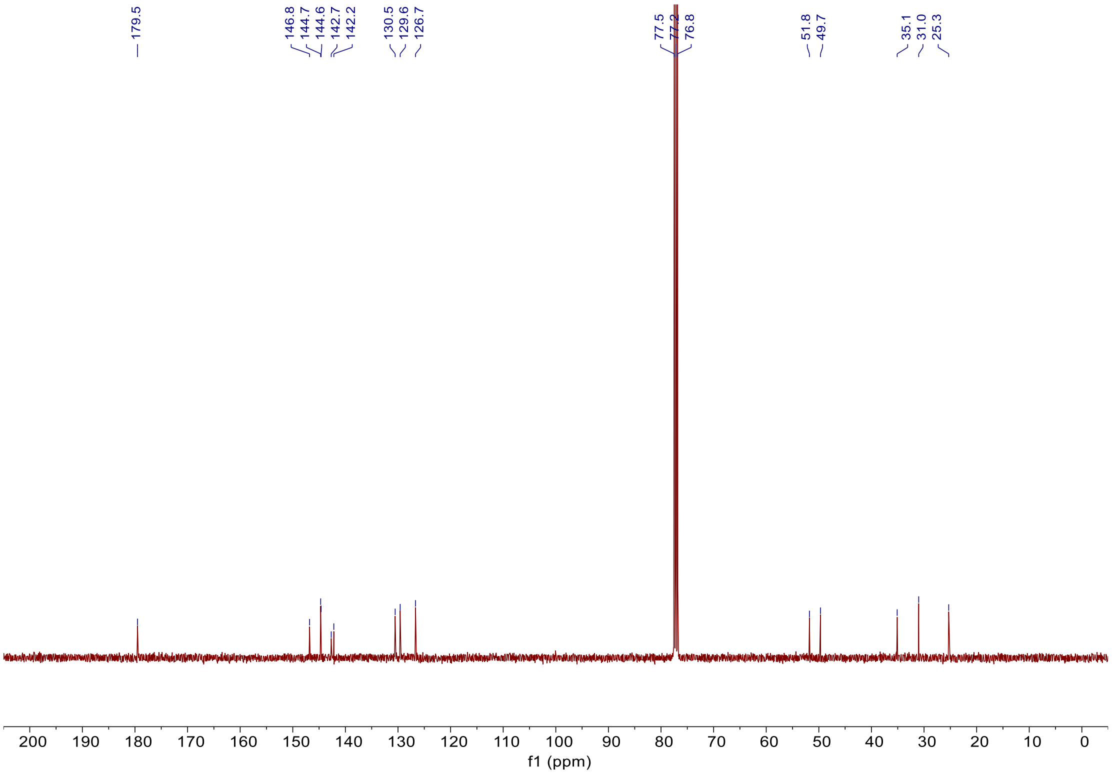

400 MHz ^{1}H NMR spectrum; 100.6 MHz ^{13}C NMR spectrum; $CDCl_3$ of *trans*-**17i**

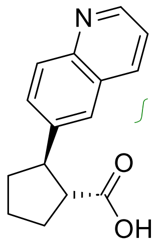

trans-**17i**

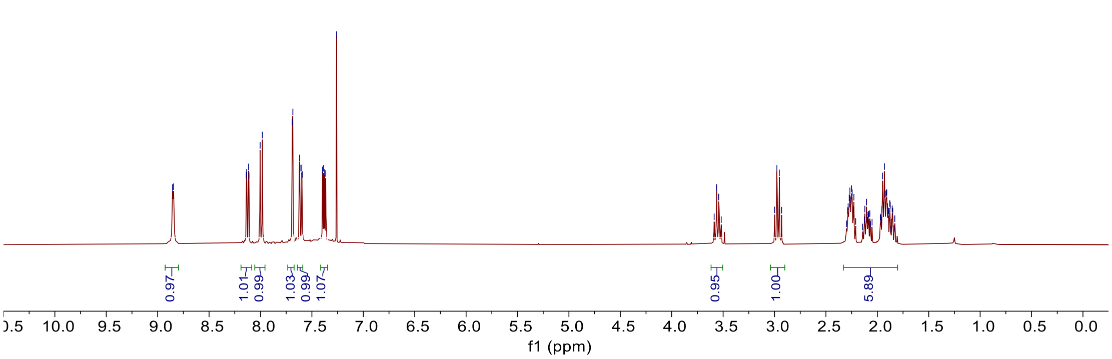

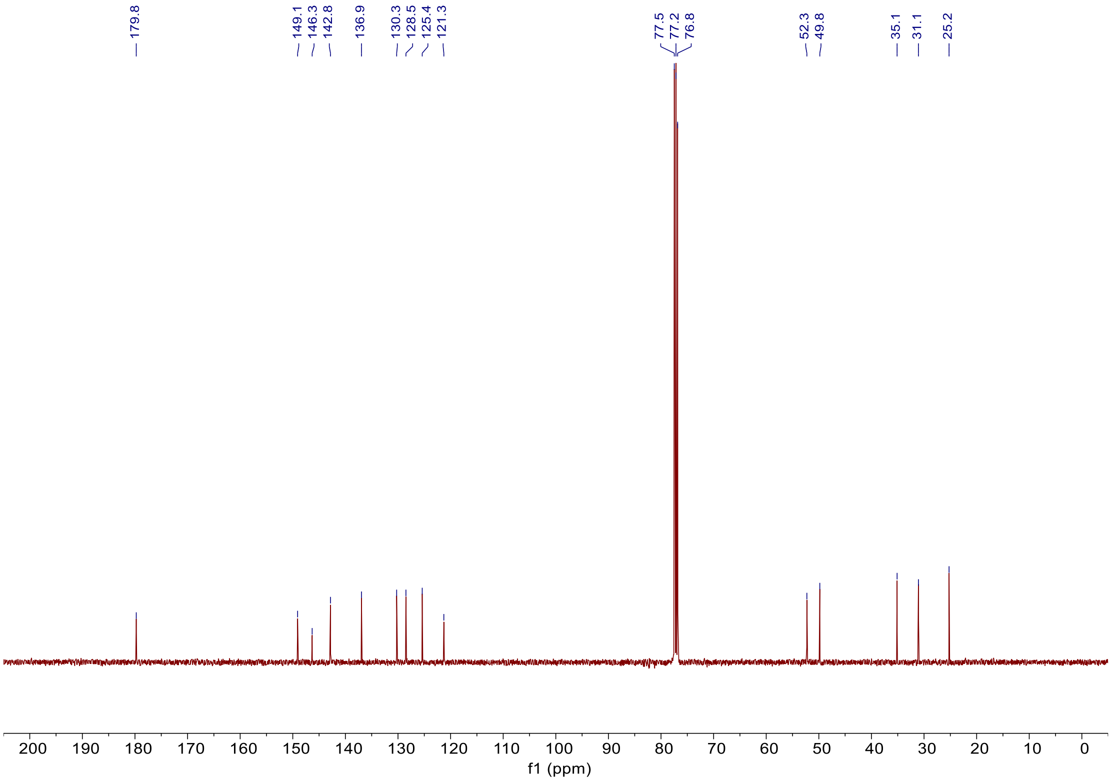

400 MHz ^{1}H NMR spectrum; 100.6 MHz ^{13}C NMR spectrum; $CDCl_3$ of *trans*-**17j**

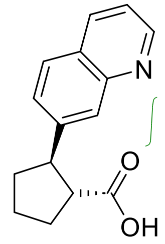

trans-**17j**

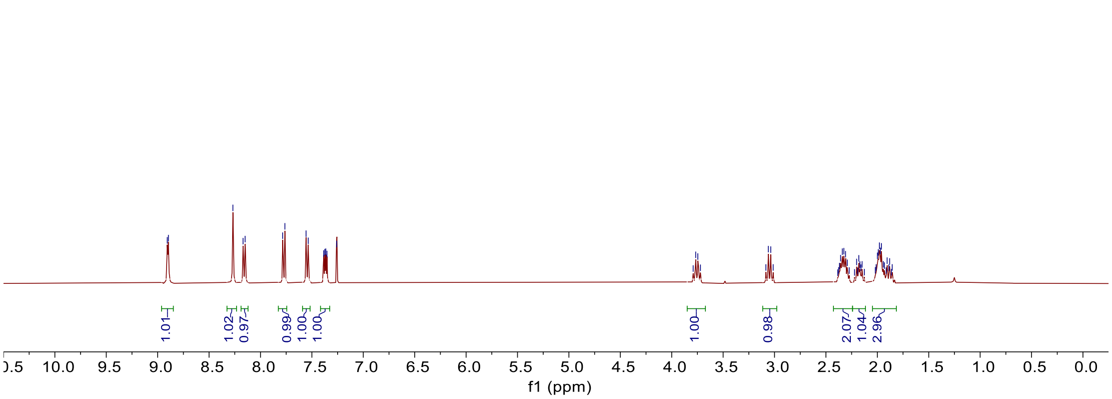

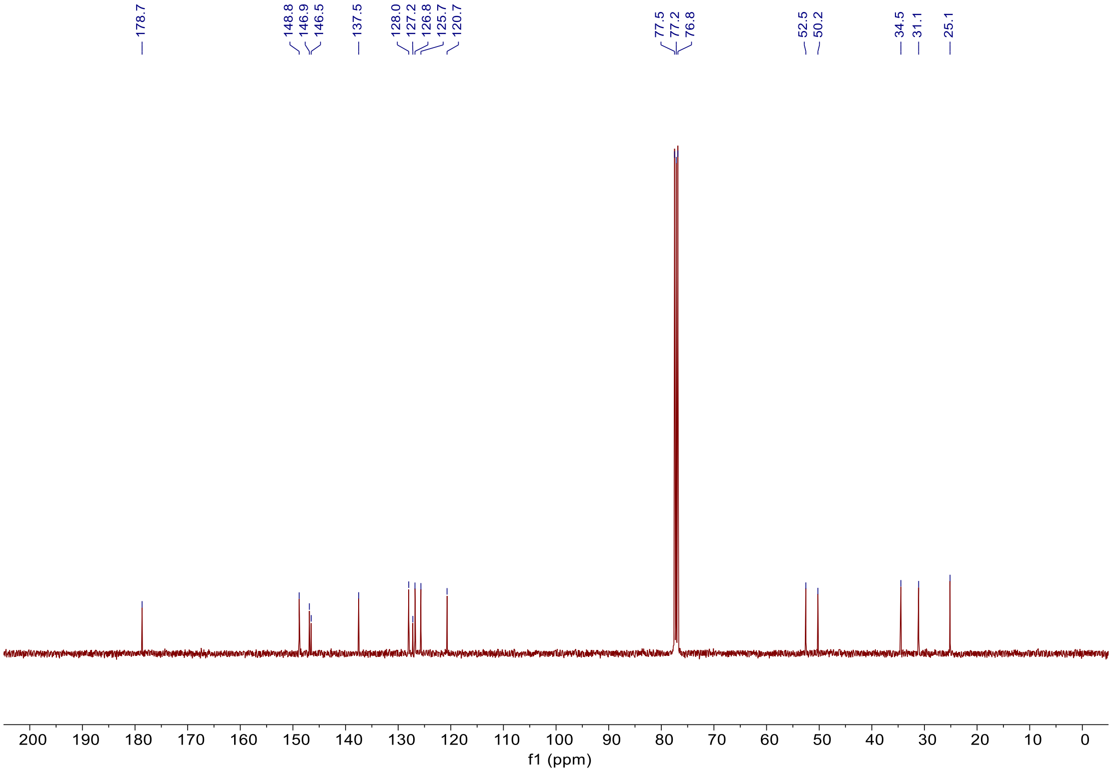

400 MHz ^{1}H NMR spectrum; 100.6 MHz ^{13}C NMR spectrum; $CDCl_3$ of **29**

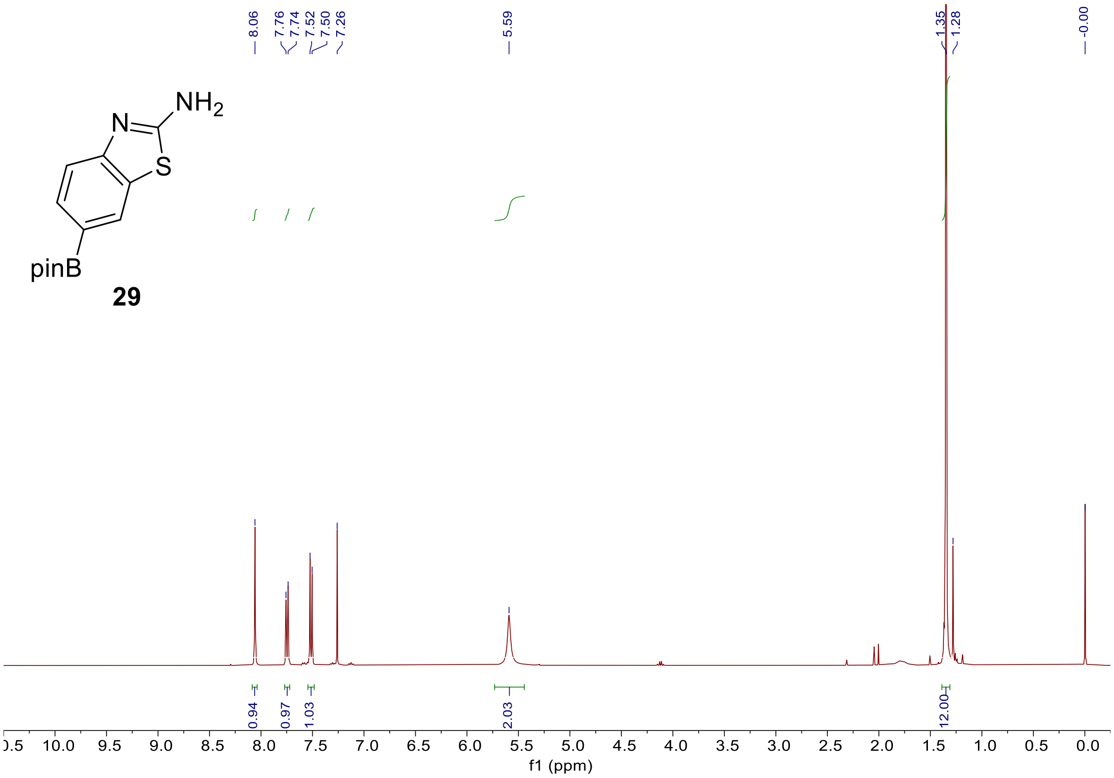

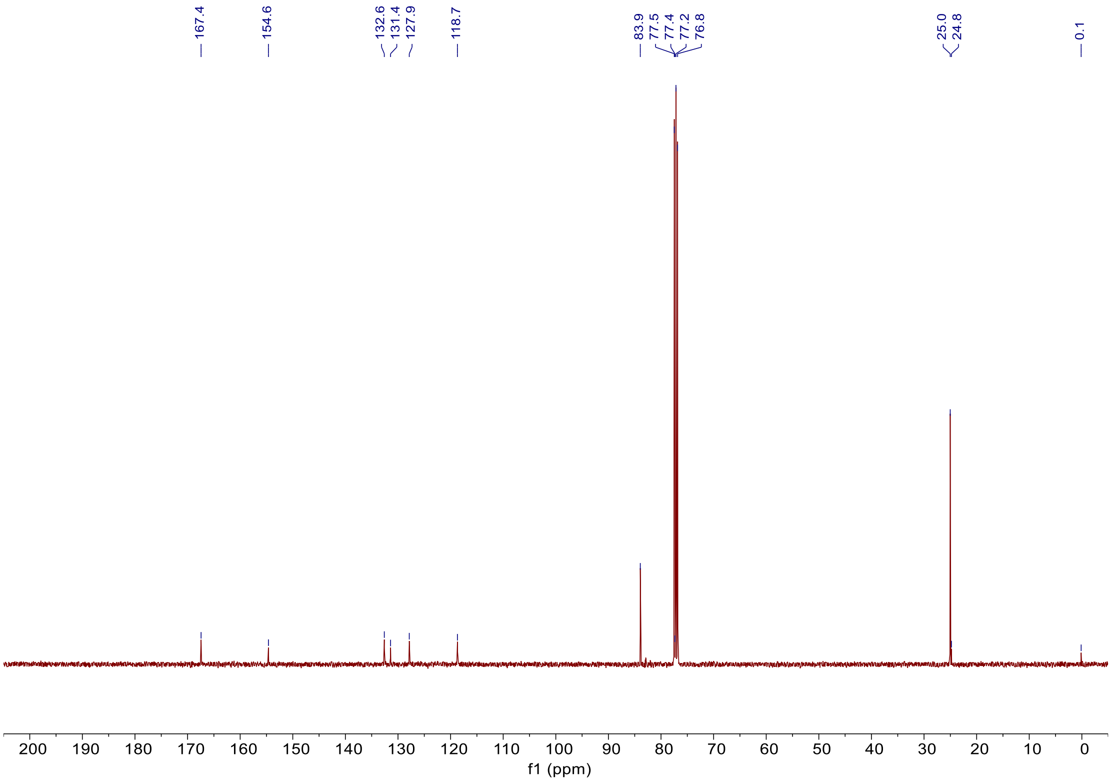

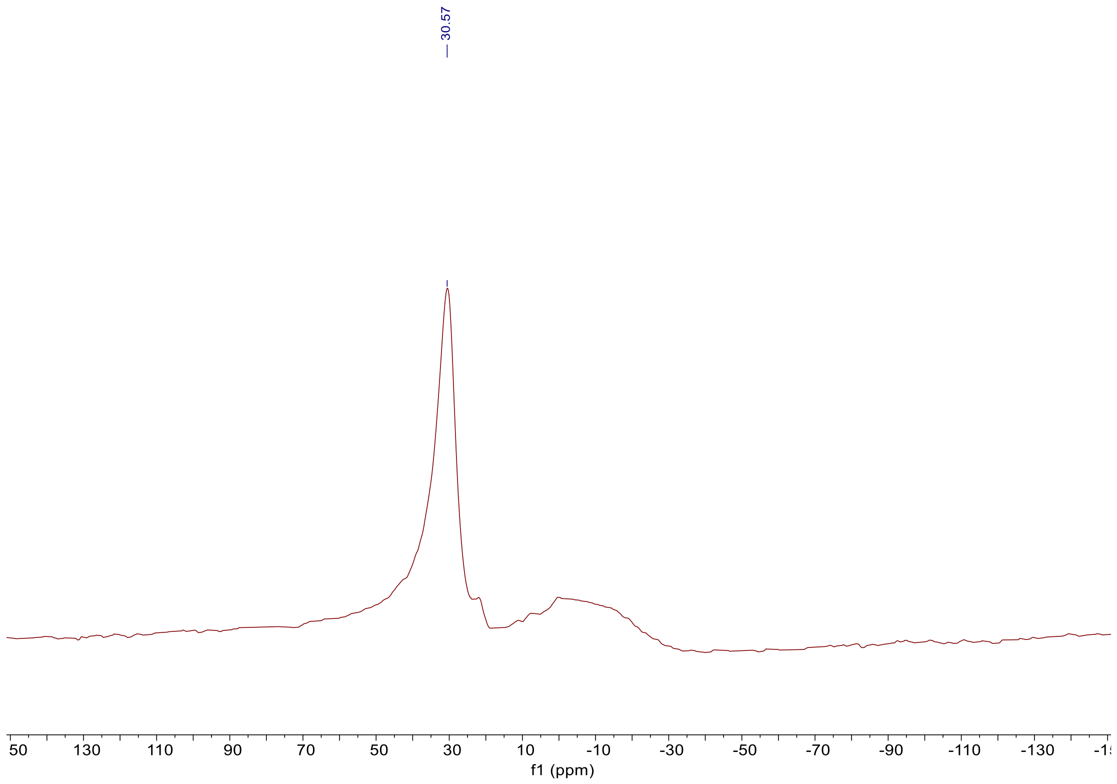
30.57
50
130
110
90
70
50
30
10
-10
-30
-50
-70
-90
-110
-130
f1 (ppm)

400 MHz ^{1}H NMR spectrum; 100.6 MHz ^{13}C NMR spectrum; $CDCl_3$ of **30a**

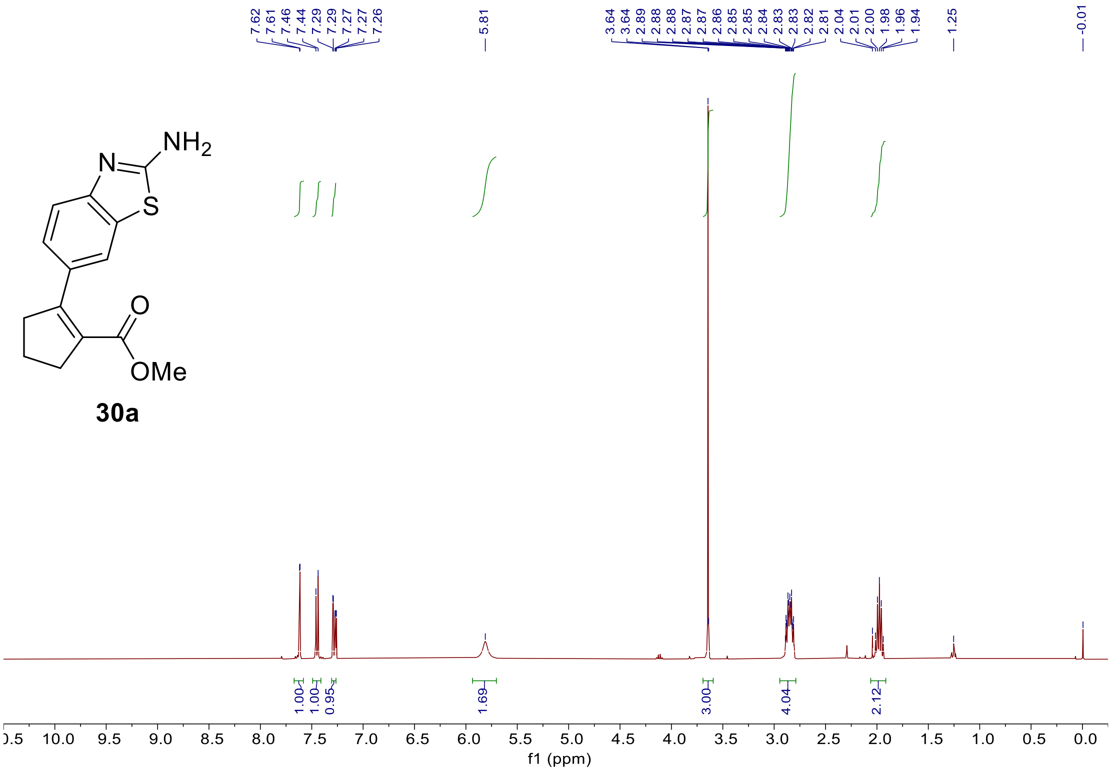

30a

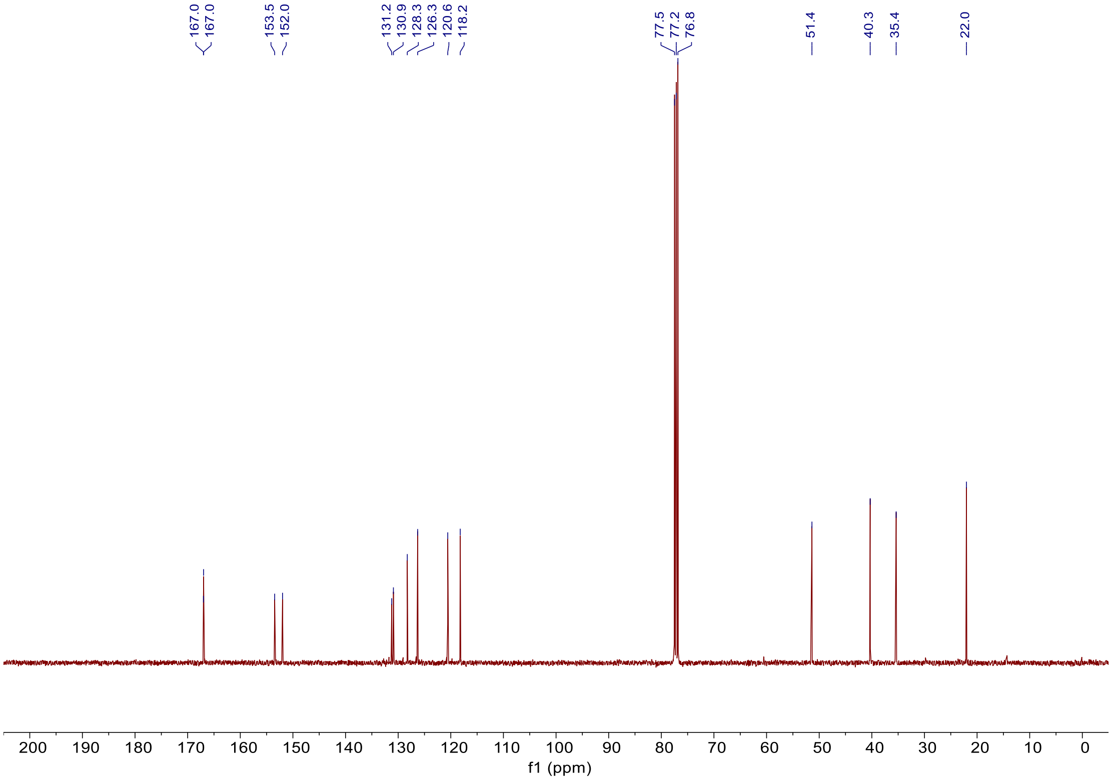

400 MHz ^{1}H NMR spectrum; 100.6 MHz ^{13}C NMR spectrum; $CDCl_3$ of **30b**

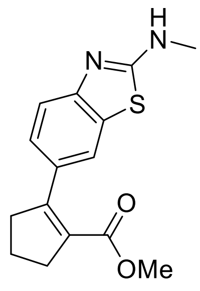

30b

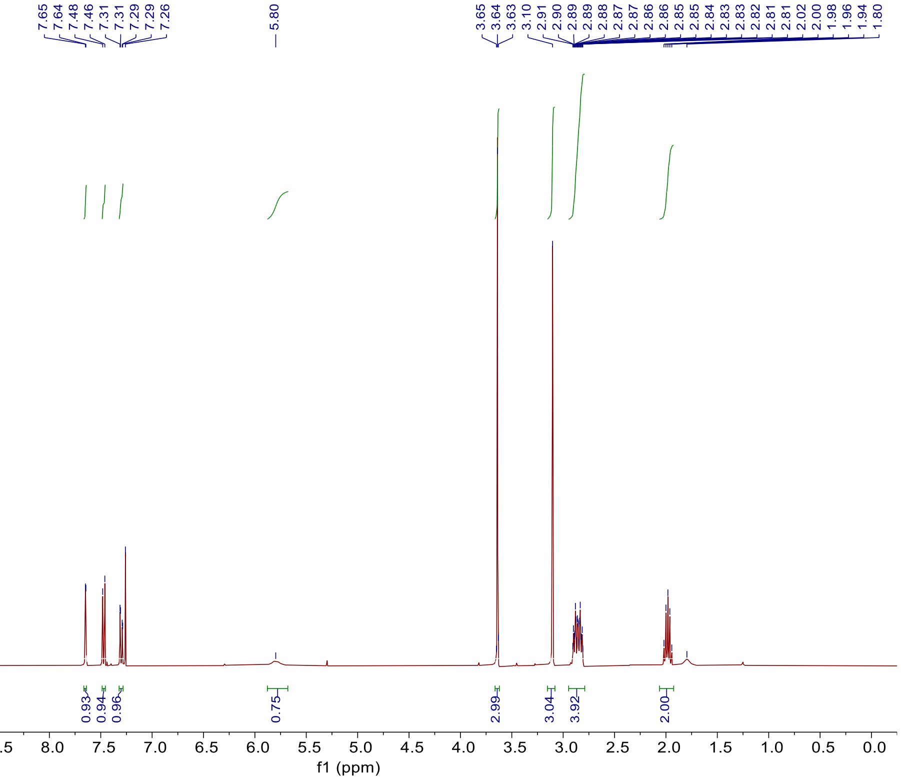

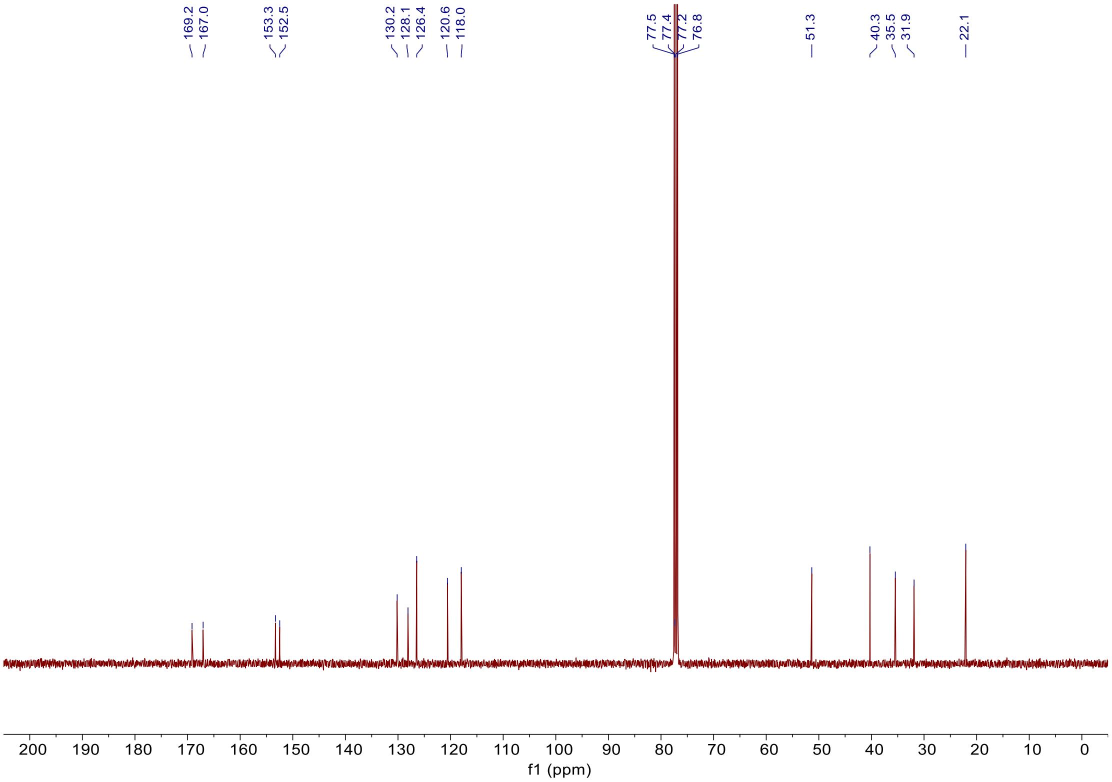

400 MHz ^{1}H NMR spectrum; 100.6 MHz ^{13}C NMR spectrum; $CDCl_3$ of **30c**

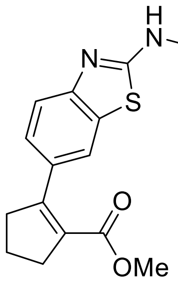

30c

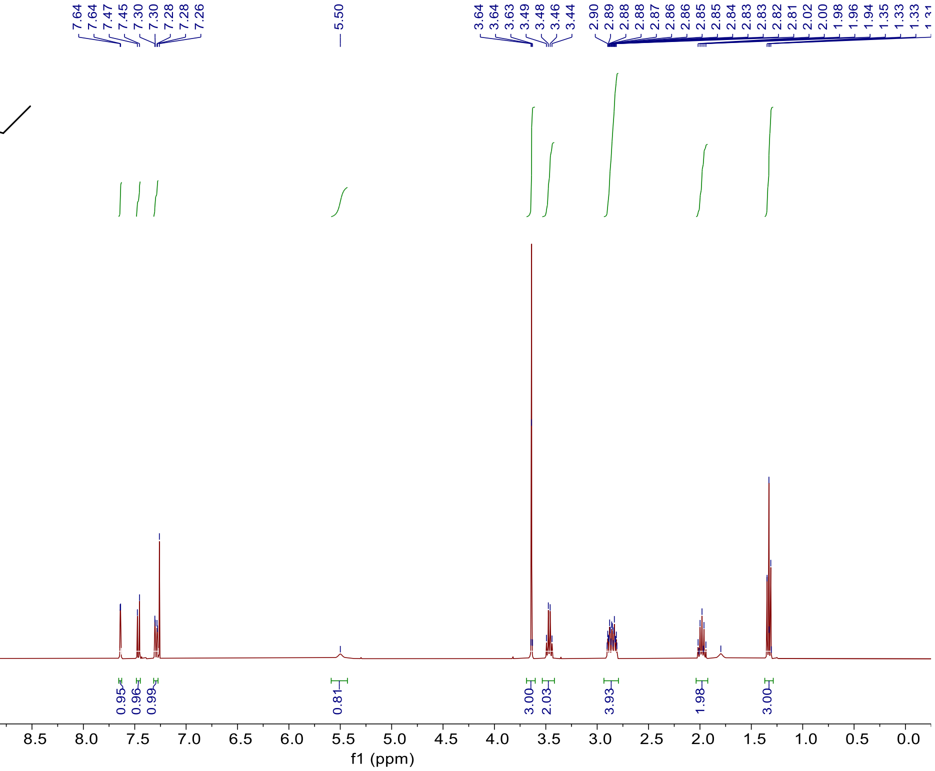

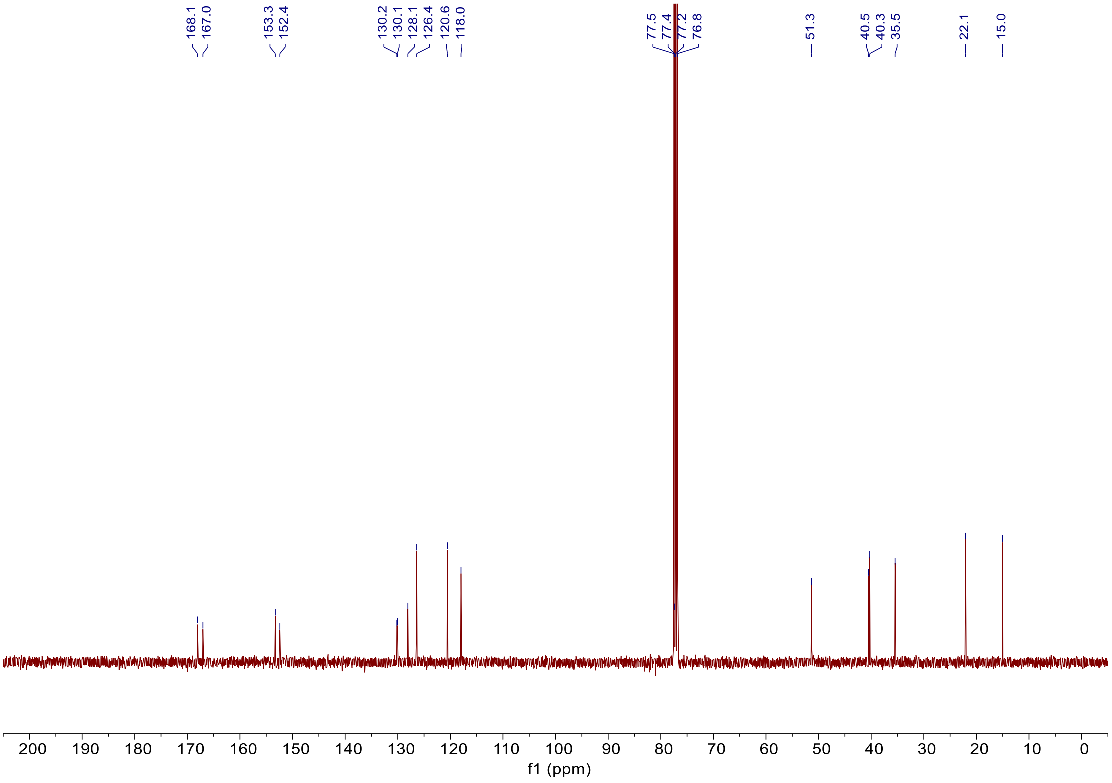

400 MHz ^{1}H NMR spectrum; 100.6 MHz ^{13}C NMR spectrum; DMSO-d_6 of **26a**

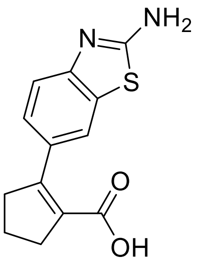

26a

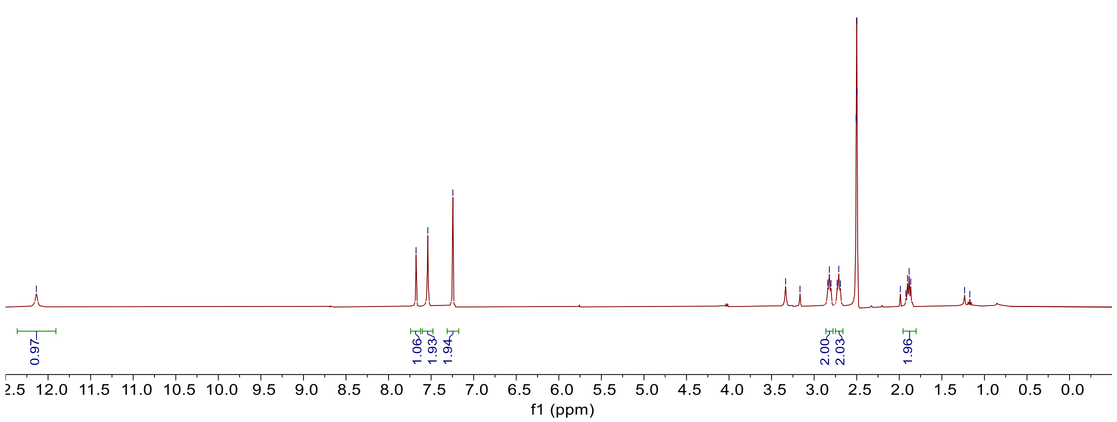

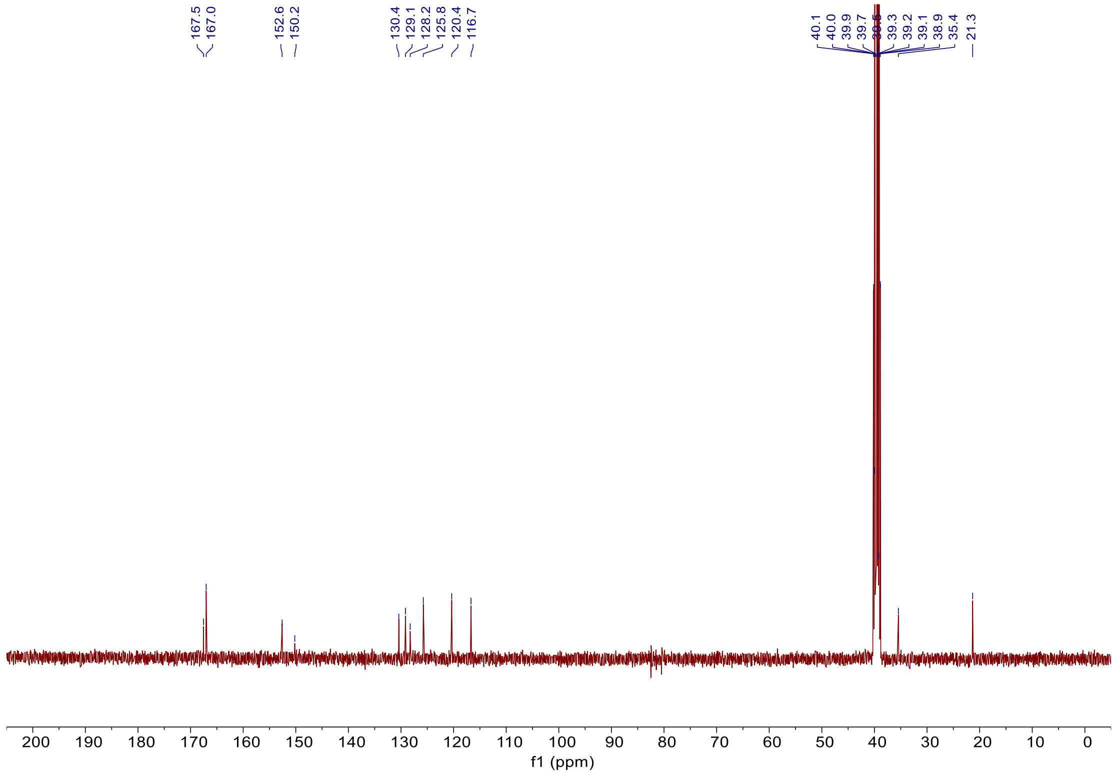

400 MHz ^{1}H NMR spectrum; 100.6 MHz ^{13}C NMR spectrum; DMSO-d_6 of **26b**

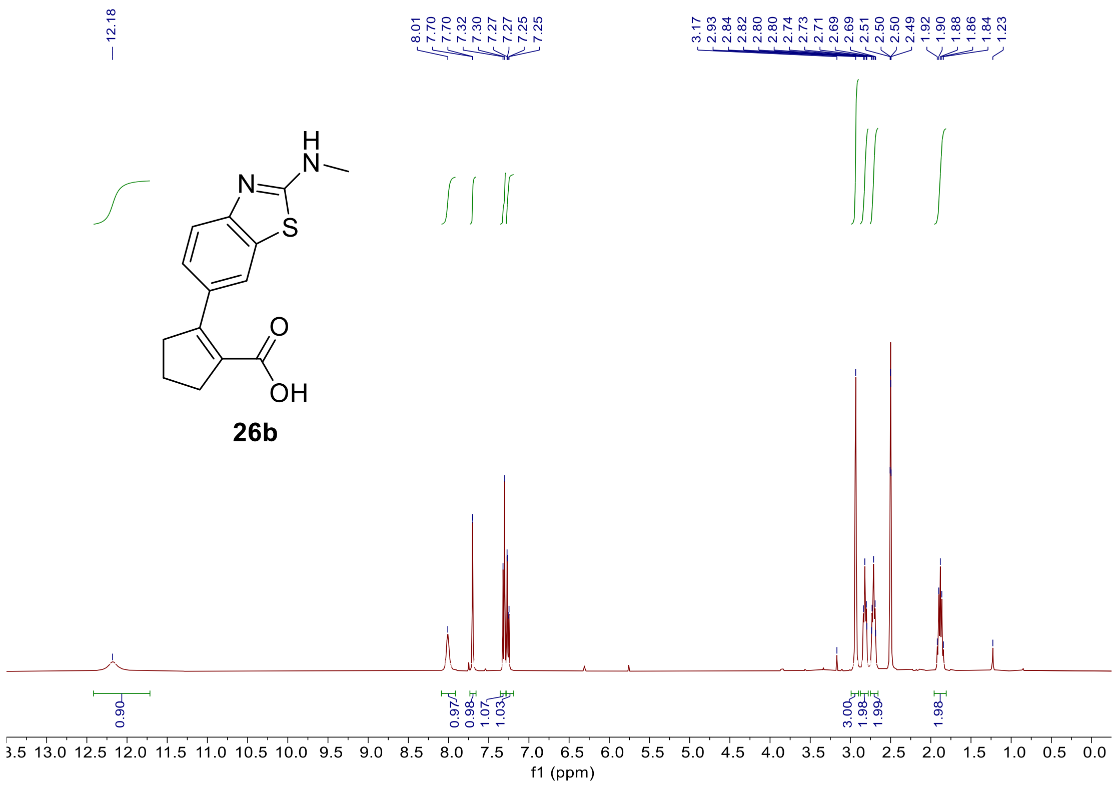

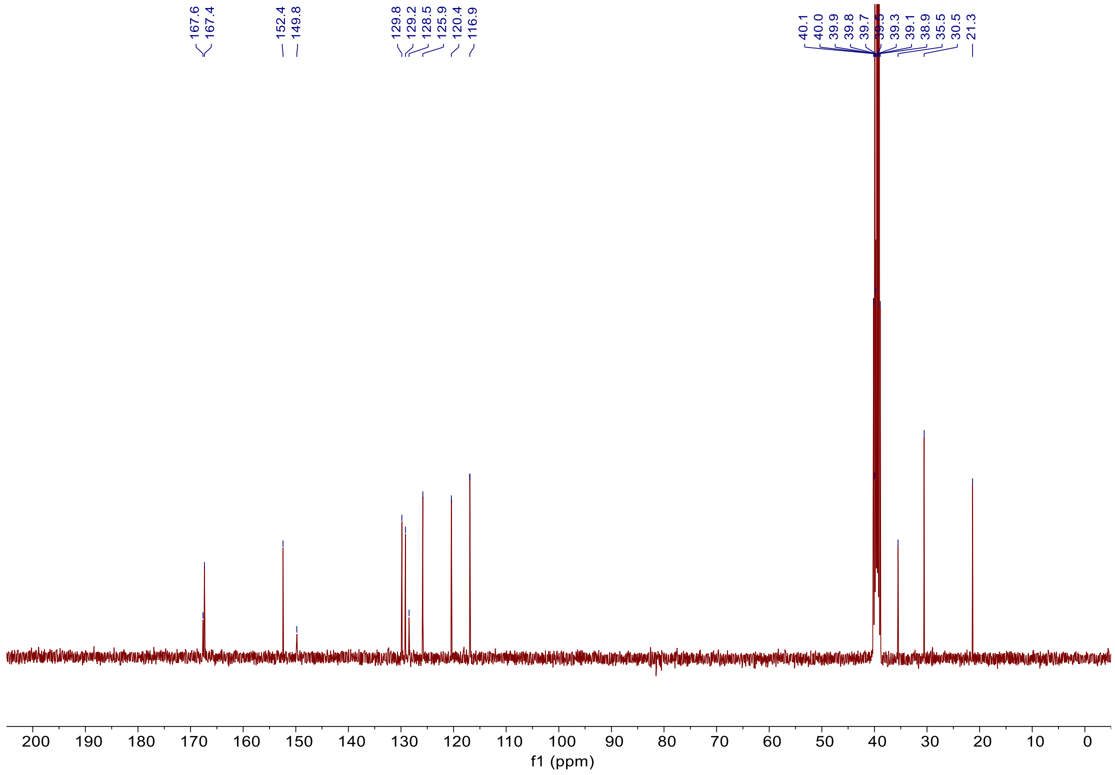

400 MHz ^{1}H NMR spectrum; 100.6 MHz ^{13}C NMR spectrum; DMSO-d_6 of **26c**

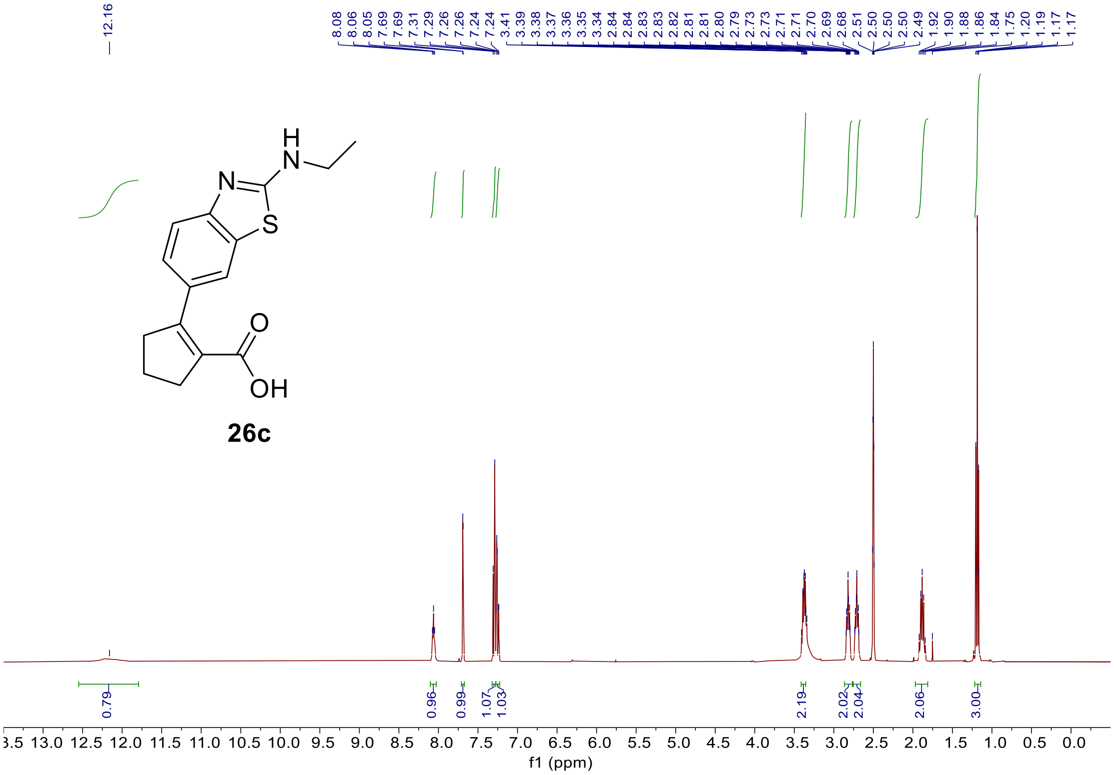

26c

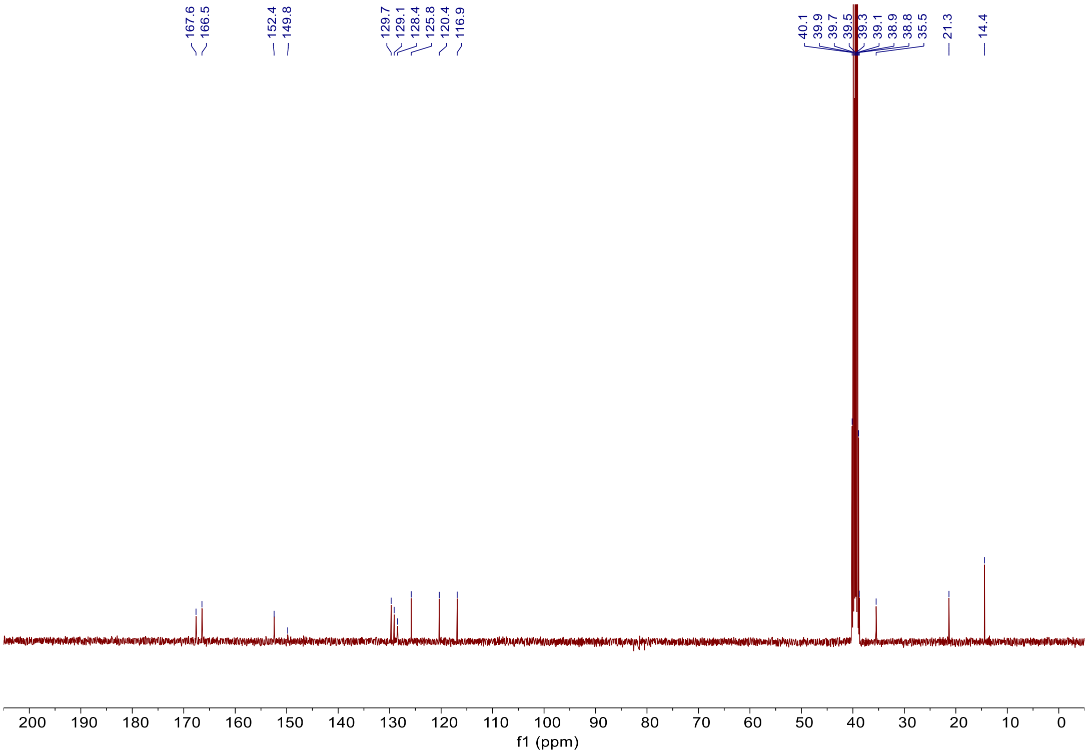

400 MHz ^{1}H NMR spectrum; 100.6 MHz ^{13}C NMR spectrum; DMSO-d_6 of **26d**

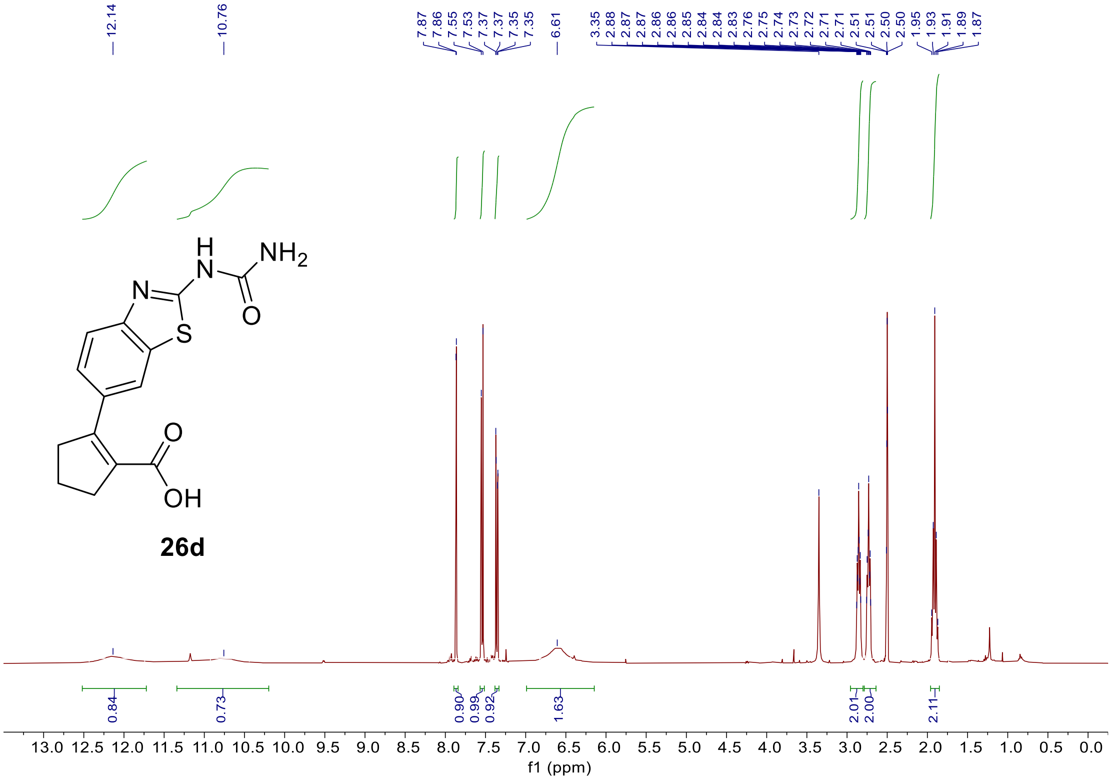

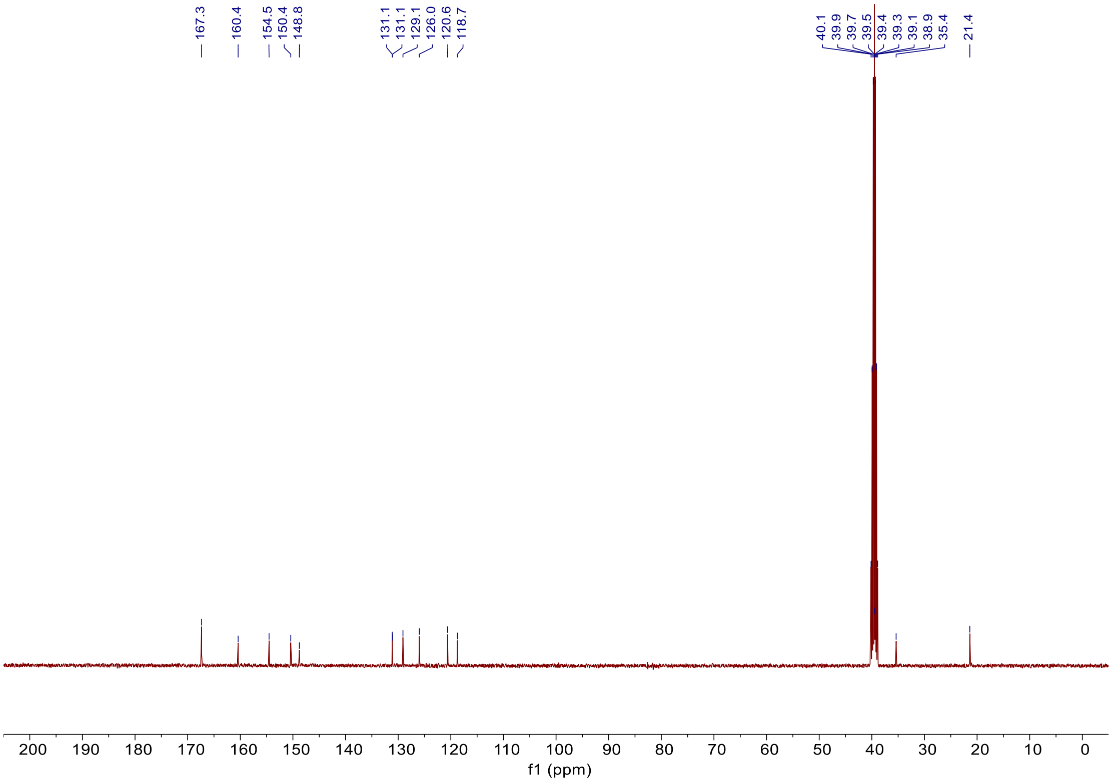

400 MHz ^{1}H NMR spectrum; 100.6 MHz ^{13}C NMR spectrum; CD_3OD of *trans*-**27**

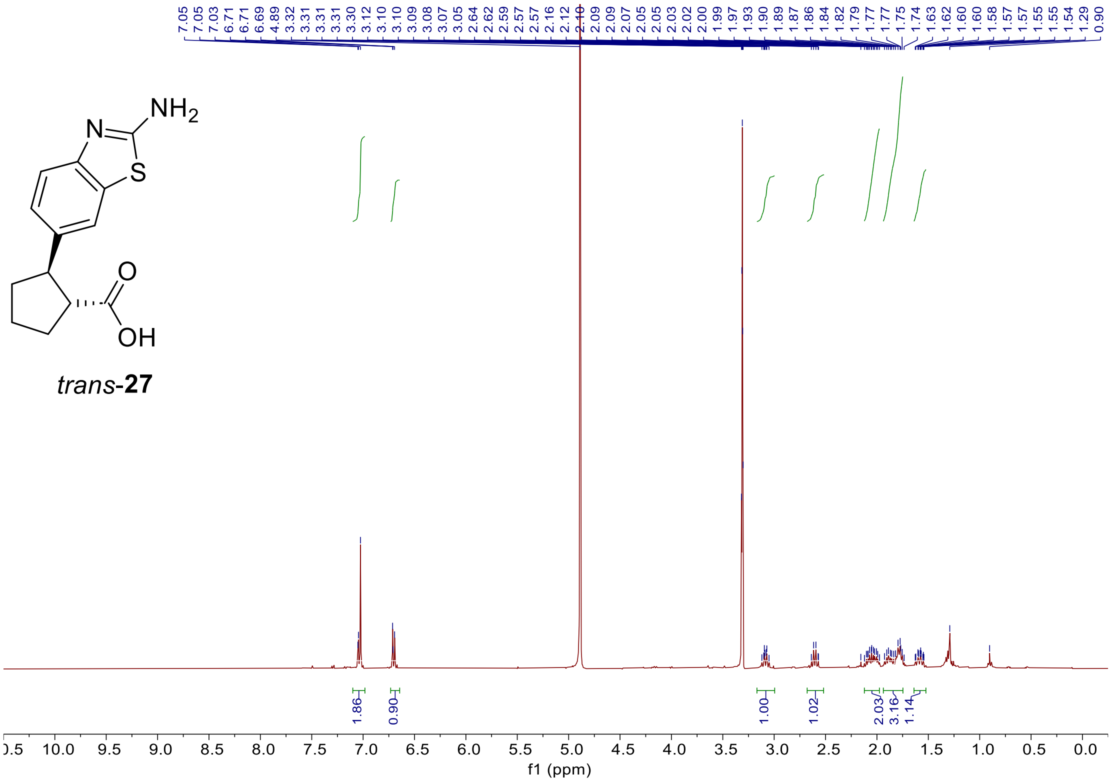

trans-**27**

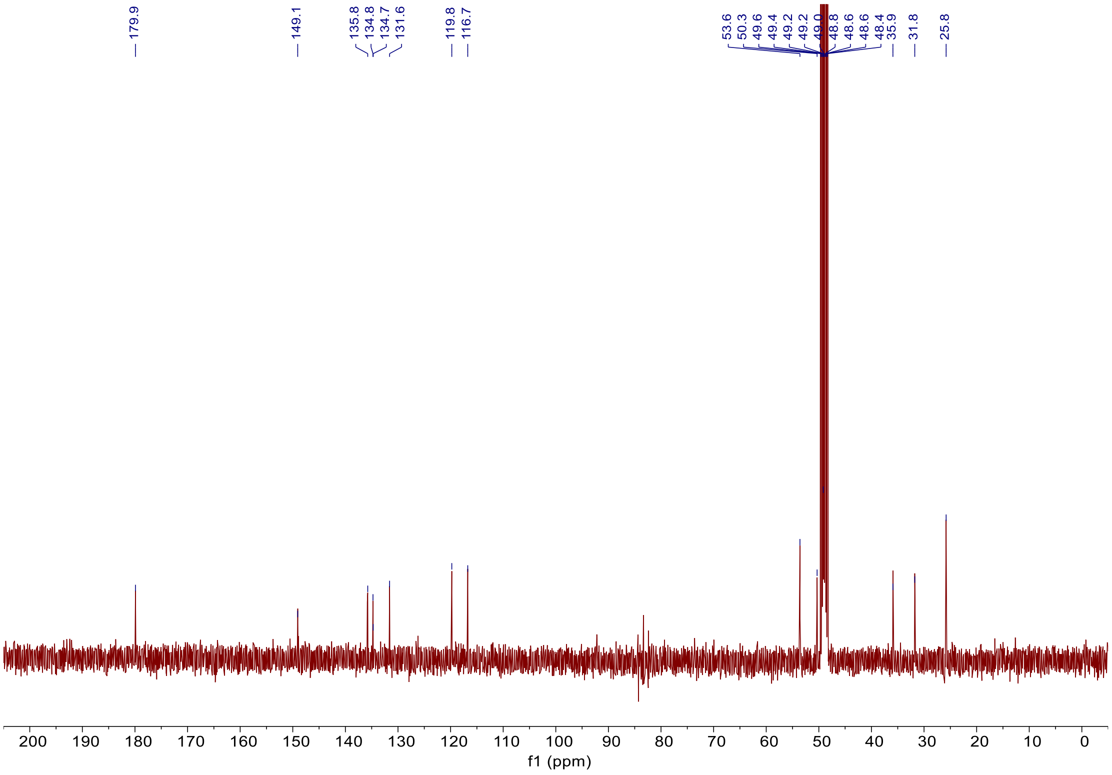

400 MHz ^{1}H NMR spectrum; 100.6 MHz ^{13}C NMR spectrum; $CDCl_3$ of *cis*-**22b**

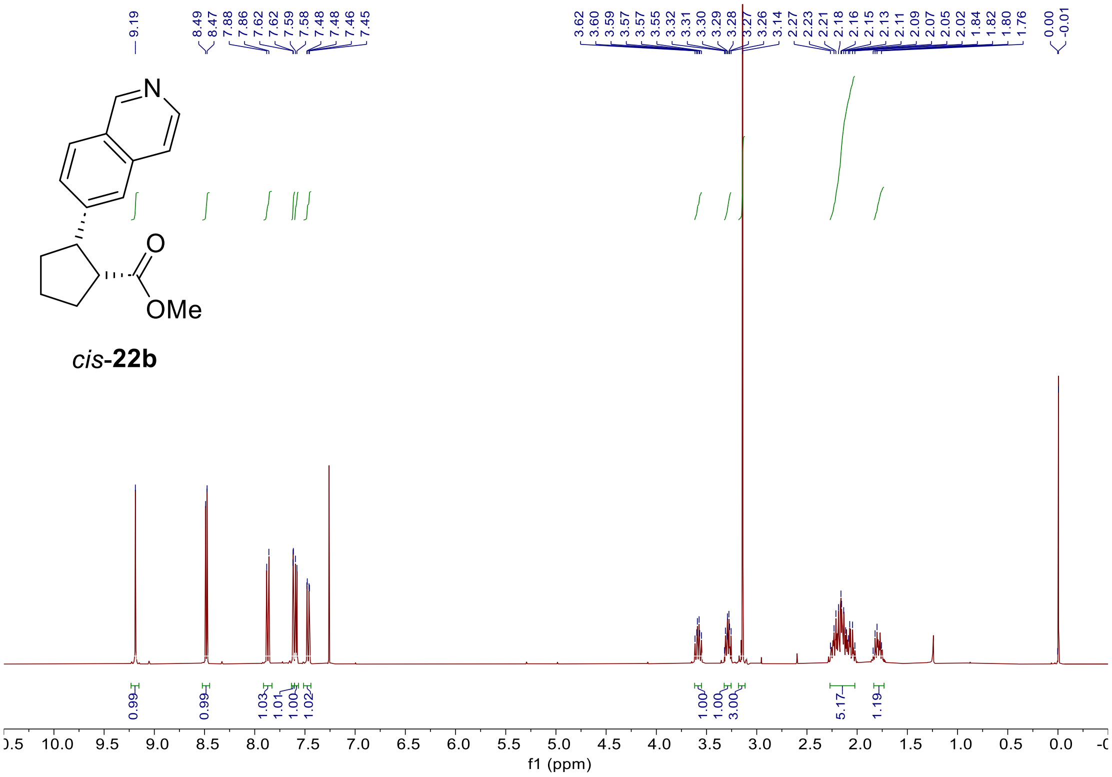

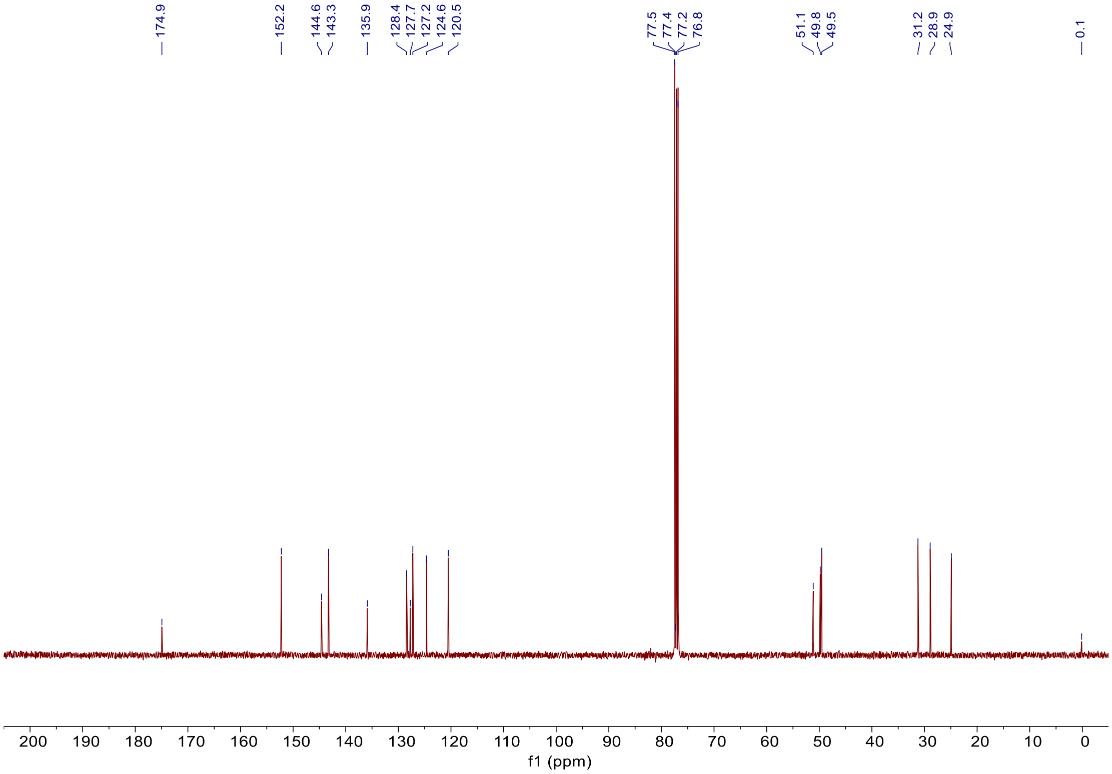

Supplement: Supplementary file 1 [file jm6c00236_si_001.pdf]
